# On the reticular construction concept of covalent organic frameworks

**DOI:** 10.3762/bjnano.1.8

**Published:** 2010-11-22

**Authors:** Binit Lukose, Agnieszka Kuc, Johannes Frenzel, Thomas Heine

**Affiliations:** 1 School of Engineering and Science, Jacobs University Bremen, Research III, Room 61, Campus Ring 1, Bremen 28759, Germany; 2 Lehrstuhl für Theoretische Chemie, Ruhr-Universität Bochum, Bochum 44780, Germany

**Keywords:** covalent organic frameworks, DFTB, energetic and electronic properties, layer stacking, XRD

## Abstract

The concept of reticular chemistry is investigated to explore the applicability of the formation of Covalent Organic Frameworks (COFs) from their defined individual building blocks. Thus, we have designed, optimized and investigated a set of reported and hypothetical 2D COFs using Density Functional Theory (DFT) and the related Density Functional based tight-binding (DFTB) method. Linear, trigonal and hexagonal building blocks have been selected for designing hexagonal COF layers. High-symmetry AA and AB stackings are considered, as well as low-symmetry serrated and inclined stackings of the layers. The latter ones are only slightly modified compared to the high-symmetry forms, but show higher energetic stability. Experimental XRD patterns found in literature also support stackings with highest formation energies. All stacking forms vary in their interlayer separations and band gaps; however, their electronic densities of states (DOS) are similar and not significantly different from that of a monolayer. The band gaps are found to be in the range of 1.7–4.0 eV. COFs built of building blocks with a greater number of aromatic rings have smaller band gaps.

## Introduction

In the past decade, considerable research efforts have been expended on nanoporous materials due to their excellent properties for many applications, such as gas storage and sieving, catalysis, selectivity, sensoring and filtration [1]. In 1994, Yaghi and co-workers introduced ways to synthesize extended structures by design. This new discipline is known as reticular chemistry [2–3], which uses well-defined building blocks to create extended crystalline structures. The feasibility of the building block approach and the geometry preservation throughout the assembly process are the key factors that lead to the design and synthesis of reticular structures.

One of the first families of materials synthesized using reticular chemistry were the so-called Metal-Organic Frameworks (MOFs) [4]. They are composed of metal-oxide connectors, which are covalently bound to organic linkers. The coordination versatility of constituent metal ions along with the functional diversity of organic linker molecules has created immense possibilities. The immense potential of MOFs is facilitated by the fact that all building blocks are inexpensive chemicals, and that the synthesis can be carried out solvothermally. MOFs are commercially available, and the scale up of production is continuing. Since the discovery of MOFs, many other crystalline frameworks have been synthesized using reticular chemistry, such as Metal-Organic Polyhedra (MOP) [5], Zeolite Imidazolate Frameworks (ZIFs) [6], and Covalent Organic Frameworks (COFs) [7].

In 2005, Coté and co-workers introduced COF materials [7–14], where organic linker molecules are stitched together by covalent entities including boron and oxygen atoms to form a regular framework. These materials have the distinct advantage that all framework bonds represent strong covalent interactions, and that they are composed of light-weight elements only, which lead to a very low mass density [7–9]. These materials can be synthesized by wet-chemical methods by condensation reactions and are composed of inexpensive and non-toxic building blocks, so their large-scale industrial application appears to be possible. From a topological viewpoint, we distinguish two- and three-dimensional COFs. In two-dimensional (2D) COFs, the covalently bound framework is restricted to layers. The crystal is then, similar as in graphite, composed of a stack of layers, which are not connected by covalent bonds.

COFs, compared with MOFs, have lower mass densities due to the absence of heavy atoms and therefore might be better for many applications. For example, the gravimetric uptake of gases is almost twice as large as that of MOFs with comparable surface areas [15–16]. Because COFs are fairly new materials, many of their properties and applications are still to be explored.

Recently, we have studied the structures of experimentally well-known 2D COFs [17]. We have found that commonly accepted 2D structures with AA and AB kinds of layering are energetically less stable than inclined and serrated forms. This is because AA stacking maximises the Coulomb repulsion due to the close vicinity of charge carrying atoms alike (O, B atoms) in neighbouring layers. The serrated and inclined forms are only slightly modified (layers are shifted with respect to each other by ≈1.4 Å) and experience less Coulomb forces between the layers, compared to AA stacking. This is equivalent to the energetic preference of graphite for an AB (Bernal) over an AA form (simple hexagonal), if we ignore the fact that interlayer ordering in serrated and inclined forms are not uniform everywhere. A known example of this is that in eclipsed hexagonal boron nitride (h-BN), boron atoms in one layer serve as nearest neighbours to nitrogen atoms in adjacent layers (AB stacking). The Coulomb interaction rules out possible interlayer eclipse between atoms with similar charges in this case.

In the present work, we aim to explore how far the concept of reticular chemistry is applicable to the individual building units, which define COFs. For this purpose, we have investigated a set of reported and hypothetical 2D COFs theoretically by exploring their structural, energetic and electronic properties. We have compared the properties of the isolated building blocks with those of the extended crystal structures, and have found that the properties of the building units are indeed maintained after their assembly to a network.

## Results and Discussion

### Structures and nomenclature

**Figure 1:**
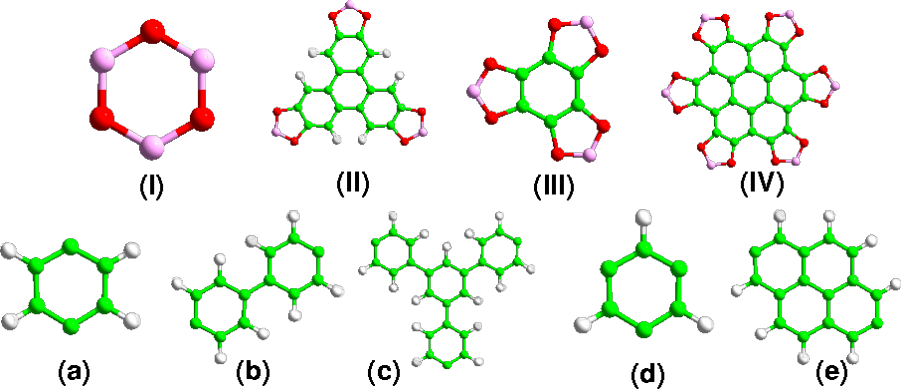
The connector (**I**–**IV**) and linker (**a**–**e**) units considered in this work. The same nomenclature is used in the text. Carbon – green; oxygen – red; boron – magenta; hydrogen – white.

We have considered four connectors (**I**–**IV**) and five linkers (**a**–**e**) for the systematic design of a number of 2D COFs (Figure 1). Each COF was built from one type of connector and one type of linker, thus resulting in the design of 20 different structures. Moreover, we have considered two hypothetical reference structures, which are only built from connectors **I** and **III** (no linker is present): REF-**I** and REF-**III**. Properties of other COFs were compared with the properties of these two structures. Some of the studied COFs are already well known in the literature [7–8,13–14] and we continue to use their traditional nomenclature, while hypothetical ones are labelled in the chronological order with an 'M' at the end which stands for 'modified'.

**Figure 2:**
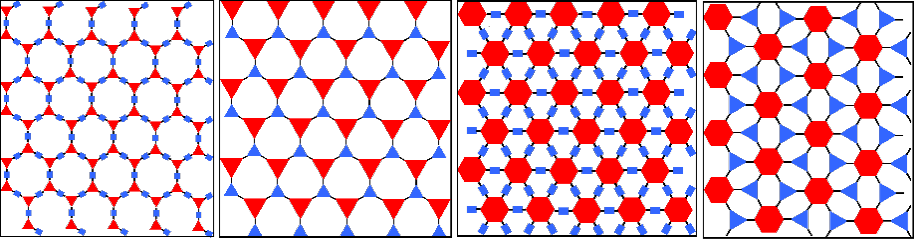
Topologies of 2D COFs considered in this work: (from the left) Tl, Tt, Hl, and Ht. Red and blue blocks are secondary building units corresponding to connectors and linkers, respectively.

Using the secondary building unit (SBU) approach, we can distinguish the connectors between trigonal [**T**] (connectors **I**, **II**, **III**) and hexagonal [**H**] (connector **IV**), and the linkers between linear [**l**] (linkers **a**, **b**, **e**) and trigonal [**t**] (linkers **c, d**). Topology of the layer is determined by the geometries of the connector and linker molecules, and typically a hexagonal pattern is formed due to the *D*_3_*_h_* symmetry of the connector moieties. Based on these topologies of the constituent building blocks, we have classified the studied COFs into four groups: **Tl**, **Tt, Hl** and **Ht** (Figure 2). Hereafter, we will use this nomenclature to describe the COF topologies.

**Figure 3:**
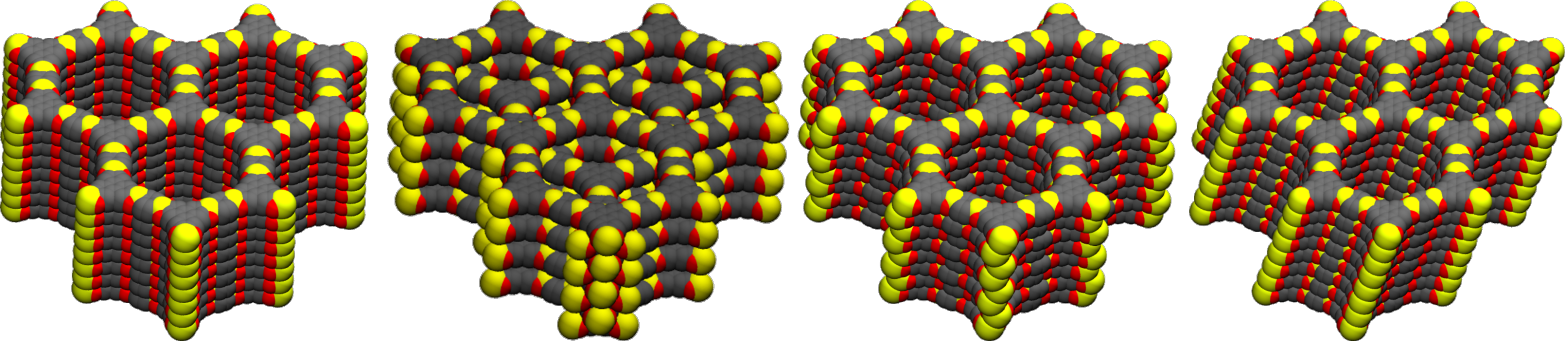
Layer stackings considered: AA, AB, serrated and inclined.

We have considered high-symmetry AA and AB kinds of stacking (hexagonal), and low-symmetry serrated (orthorhombic) and inclined (monoclinic) kinds of stacking of the layers. The latter two were discussed in a previous work on 2D COFs [17]. As an example, the structure of COF-5 [7] in different kinds of stacking of layers is shown in Figure 3. In eclipsed AA stacking, atoms of adjacent layers lie directly on top of each other, whilst in staggered AB stacking, three-connected vertices lie directly on top of the geometric centre of six-membered rings of neighbouring layers. In both serrated and inclined kinds of stacking, the layers are shifted with respect to each other by approximately 1.4 Å, resulting in hexagonal rings in the connector or linker being staggered with those in the adjacent layers. In serrated stacking, alternate layers are eclipsed. In inclined stacking, layers lie shifted along one direction and the lattice vector pointing out of the 2D plane is not rectangular. For COFs made of connector **I**, due to the absence of five-membered C_2_O_2_B rings, a zigzag shift leads to staggering in both connector and linker parts. For those made of other connectors, staggering at the connector or linker depends on whether the shift is armchair or zigzag, respectively [17].

### Structural properties

We have compared structural properties of isolated building blocks with those of the extended COF structures. Negligible differences have been found in the bond lengths and bond angles of the building blocks and the corresponding crystal structures. This indicates that the structural integrity of the building blocks remains unchanged while forming covalent organic frameworks, confirming the principle of reticular chemistry. In addition, the C–B, B–O and O–C bond lengths are almost the same when different COF structures are compared (see Table S1 in Supporting Information File 1). The experimental bond lengths are ≈1.54 Å for C–B, ≈1.38 Å for O–C and 1.37–1.48 Å for B–O. However, in the case of COF-1 the experimental values are slightly larger (1.60 Å for C–B and 1.51 Å for B–O). This could be the reason why our calculated bond lengths for COF-1 are much shorter than the experimental values, while all the other structures agree within 5% error. The calculated C–C bond lengths vary in the range from 1.36–1.47 Å (Figure S2 in Supporting Information File 1) and are the same for the COFs and their constituent building blocks at the respective positions of the carbon atoms. In addition, the reference structures, REF-**I** and REF-**III**, have direct B–B bond lengths of 1.67 Å and 1.66 Å, respectively, which is shorter by 0.14 Å than a typical B–B bond length. The calculated bond angles OBO in B_3_O_3_ and C_2_O_2_B rings are 120° and 113°, respectively.

Interlayer distances (*d*), which is the shortest distance between two layers (equivalent to *c* for AA; *c*/2 for AB and serrated), are different in all kinds of stacking: AB stacked 2D COFs have shorter interlayer distances than the corresponding AA, serrated, and inclined stacked structures. Among the latter three, AA stacked COFs have higher values for *d* because of the higher repulsive interlayer orbital interactions, resulting from the direct overlap of polarized alike atoms between the adjacent layers. This results in higher mass densities for AB stacked COF analogues. Serrated and inclined stacks have only slightly higher mass densities compared to AA. The differences in mass densities for all kinds of stacking are attributed to the differences in their interlayer separations. The *d* values of most of the COFs are larger than that of graphite in AA stacking but smaller in AB stacking.

Cell parameters (*a*) and mass densities (*ρ*) of all the COFs constructed from the considered connectors and linkers are shown in Table 1 (and in Table S3 in Supporting Information File 1). Mass densities of all the COFs are much lower than that of graphite (2.27 g·cm^−3^) and diamond (3.50 g·cm^−3^). AA/serrated/inclined stacked COF-10s have the lowest mass densities (0.45/0.46/0.46 g·cm^−3^), which is lower than that of MOF-5 (0.59 g·cm^−3^) [4], and comparable to that of highly porous MOF-177 (0.42 g·cm^−3^) [18].

**Table 1:** The calculated unit cell parameter *a* [Å], interlayer distance *d* [Å] and mass density *ρ* [g·cm^−3^] for AA and AB stacked COFs. Note that the cell parameter *a* is the same for all stacking types. Experimental data [7,19] is given in parentheses.

COF	Building Blocks	*a* [Å]	*d* [Å]		*ρ* [g·cm^−3^]	
			**AA**	**AB**	**AA**	**AB**

**COF-1**	**I-a**	15.02 (15.620)	3.51	3.13 (3.32)	0.94	1.06
**COF-1M**	**I-b**	22.41	3.49	3.04	0.68	0.78
**COF-2M**	**I-c**	14.92	3.47	3.12	0.95	1.06
**COF-3M**	**I-d**	07.47	3.49	3.27	1.53	1.64
**PPy-COF**	**I-e**	22.32 (22.163)	3.49 (3.421)	2.97	0.84	0.99
**COF-5**	**II-a**	30.14 (30.020)	3.47 (3.460)	3.26	0.56	0.60
**COF-10**	**II-b**	37.58 (37.810)	3.47 (3.476)	3.18	0.45 (0.45)	0.50
**COF-8**	**II-c**	22.51 (22.733)	3.46 (3.476)	3.20	0.71 (0.70)	0.77
**COF-6**	**II-d**	15.05 (15.091)	3.46 (3.599)	3.27	1.04	1.10
**TP COF**	**II-e**	37.50 (37.541)	3.48 (3.378)	3.20	0.51	0.56
**COF-4M**	**III-a**	21.71	3.50	3.18	0.73	0.80
**COF-5M**	**III-b**	29.15	3.50	3.18	0.55	0.61
**COF-6M**	**III-c**	18.33	3.45	3.18	0.83	0.90
**COF-7M**	**III-d**	10.83	3.50	3.30	1.29	1.36
**TP COF-1M**	**III-e**	29.05	3.49	3.10	0.65	0.74
**COF-8M**	**IV-a**	17.48	3.59	3.29	1.40	1.48
**COF-9M**	**IV-b**	21.76	3.49	3.30	1.17	1.74
**COF-10M**	**IV-c**	22.54	3.42	3.36	1.27	1.28
**COF-11M**	**IV-d**	15.12	3.46	3.38	1.68	1.72
**TP COF-2M**	**IV-e**	21.73	3.47	3.32	1.34	1.40
**REF-I**	**I**	07.73	3.59	3.36	1.44	1.48
**REF-III**	**III**	14.45	3.53	3.36	1.04	1.21
**Graphite**		2.47	3.43	3.35	2.20	2.27

**Figure 4:**
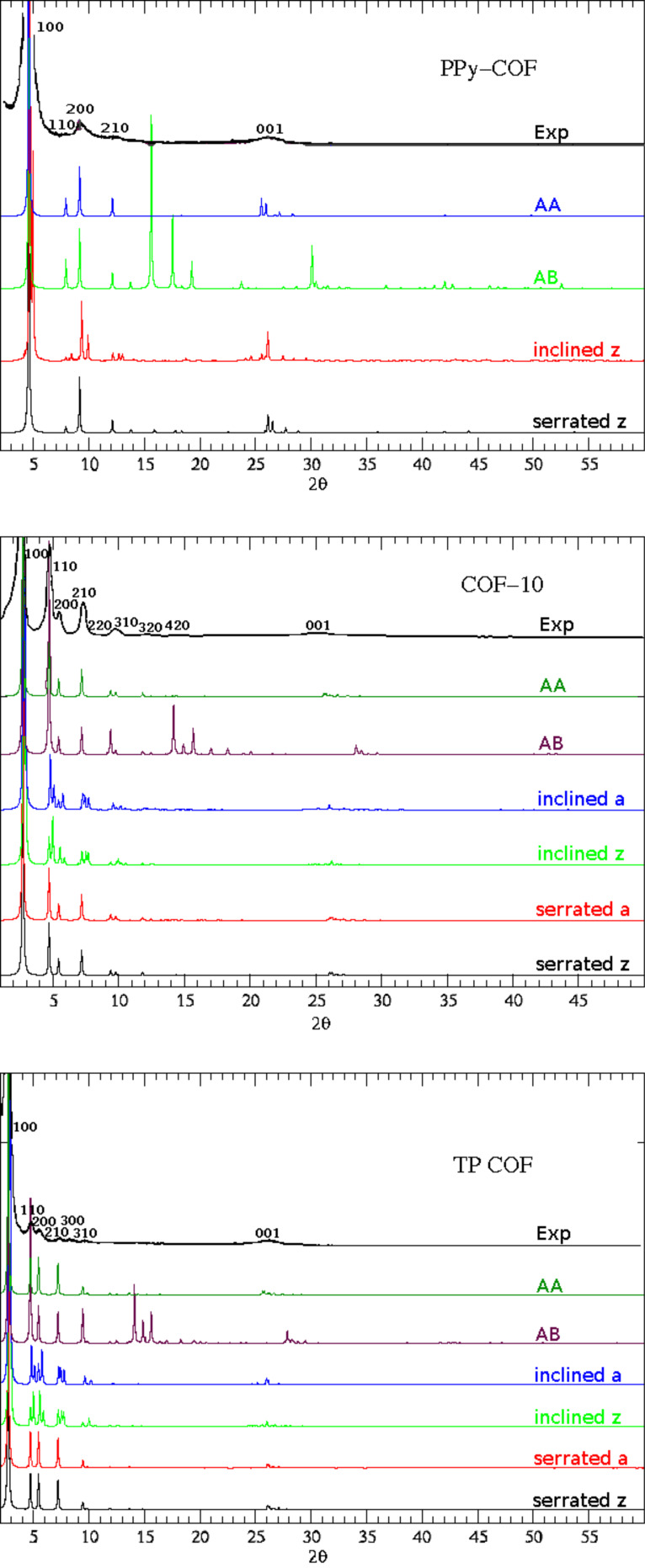
The calculated and experimental [8,13–14] XRD patterns of PPy-COF (top), COF-10 (middle) and TP COF (bottom).

In order to identify the stacking orders, we have analyzed X-ray diffraction (XRD) patterns of the well-known COFs (COF-10, TP COF, PPy-COF, see Figure 4) in all the above discussed stacking kinds. The change of stacking should be visible in XRDs because each space group has a distinct set of symmetry imposed reflection conditions. The XRD patterns of AA, serrated and inclined stacking kinds, which differ within a slight shift of adjacent layers to specific directions, are in agreement with the presently available experimental data [8,13–14]. Their peaks are at the same angles as in the experimental spectrum, whereas AB stacking clearly shows differences. The slight differences in the (001) angle between each stacking resemble the differences in their interlayer separations. The inclined stackings have more peaks; however, they are covered by the broad peaks in the experimental patterns. Similar results for COF-1, COF-5, COF-6 and COF-8 have been discussed in our previous work [17].

### Energetic stability

**Figure 5:**
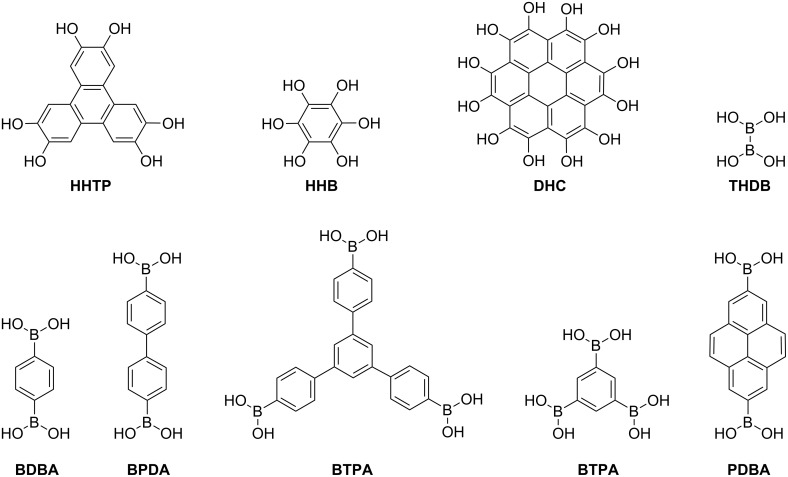
The reactants participating in the formation of 2D COFs.

We have considered dehydration reactions, the basis of experimental COF synthesis, to calculate formation energies of COFs in order to predict their energetic stability. Molecular units, 1,4-phenylenediboronic acid (BDBA), [1,1’-biphenyl]-4,4'-diylboronic acid (BPDA), 5’-(4-boronophenyl)-[1,1’:3’,1”-terphenyl]-4,4”-diboronic acid (BTPA), benzene-1,3,5-triyltriboronic acid (BTBA) and pyrene-2,7-diylboronic acid (PDBA) were considered as linkers **a**–**e**, respectively, with -B(OH)_2_ groups attached to each point of extension (Figure 5). Self-condensation of these building blocks result in the formation of B_3_O_3_ rings and the resultant COFs are those made of connector **I** and the corresponding linkers. This process requires a release of three or six water molecules in case of **t** or **l** topology, respectively.

Co-condensation of the above molecular units with compounds such as 2,3,6,7,10,11-hexahydroxytriphenylene (HHTP), hexahydroxybenzene (HHB) and dodecahydroxycoronene (DHC) (Figure 5) gives rise to COFs made of connectors **II**, **III** and **IV**, respectively, and the corresponding linkers. Self-condensation of tetrahydroxydiborane (THDB) and co-condensation of HHB with THDB result in the formation of the reference structures, REF-**I** and REF-**III**, respectively. In relation to the corresponding connector/linker topologies, these building blocks satisfy the following equations of the co-condensation reaction for COF formation:

[1]
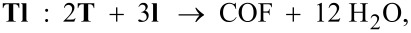


[2]
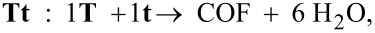


[3]
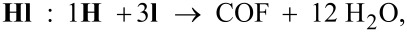


[4]
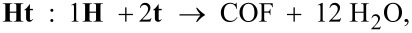


with a stochiometry appropriate for one unit cell. The number of covalent bonds formed between the building blocks is equivalent to the number of released water molecules, we refer to it as “formula unit” and will give all energies in the following in kJ·mol^−1^ per formula unit.

We have calculated the condensation energy of a single COF layer formed from monomers (building blocks, hereafter called bb) according to the above reactions as follows:

[5]
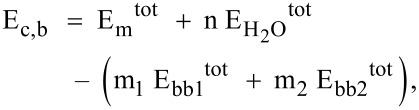


where E_m_^tot^ is the total energy of the monolayer; E_H2O_^tot^ is the total energy of the water molecule; E_bb1_^tot^ and E_bb2_^tot^ are the total energies of interacting building blocks; and n, m_1_, m_2_ are the corresponding stoichiometry numbers.

We have also calculated the stacking energy, E_s,b_, of layers:

[6]
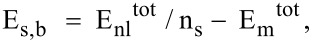


where E_nl_^tot^ is the total energy of n_s_ number of layers stacked in a COF. Finally, the COF formation energy can be given as a sum of E_c,b_ and E_s,b_. The calculated energies are given in Table 2 (and Table S4 in Supporting Information File 1) for all the studied stacking kinds.

**Table 2:** The calculated energies [kJ•mol^−1^] per bond formed between building blocks for AA and AB stacked COFs: E_c,b_ is the condensation energy, E_s,b_ is the stacking energy, and E_f,b_ is the COF formation energy (E_f,b_ = E_c,b_ + E_s,b_). The calculated band gaps, ∆ [eV], are given as well.

COF	Building Blocks	Mono- layer	AA			AB		
		**E_c,b_** **[kJ·mol^−1^]**	**E_s,b_** **[kJ·mol^−1^]**	**E_f,b_** **[kJ·mol^−1^]**	**∆** **[eV]**	**E_s,b_** **[kJ·mol^−1^]**	**E_f,b_** **[kJ·mol^−1^]**	**∆** **[eV]**

**COF-1**	**I-a**	9.06	−26.83	−17.77	3.3	−21.87	−12.80	3.6
**COF-1M**	**I-b**	9.49	−42.66	−33.66	2.7	−24.18	−14.69	3.1
**COF-2M**	**I-c**	9.56	−57.27	−47.71	2.8	−47.34	−37.78	3.0
**COF-3M**	**I-d**	7.63	−25.06	−17.42	3.8	−28.01	−20.37	4.0
**PPy-COF**	**I-e**	8.58	−57.23	−48.66	2.4	−38.55	−29.98	2.6
**COF-5**	**II-a**	2.11	−29.68	−27.56	2.4	−25.48	−23.37	2.8
**COF-10**	**II-b**	3.17	−37.66	−34.48	2.3	−13.44	−10.26	2.6
**COF-8**	**II-c**	2.63	−44.88	−42.24	2.5	−24.77	−22.13	2.8
**COF-6**	**II-d**	1.85	−28.81	−26.95	2.8	−21.27	−19.42	3.1
**TP COF**	**II-e**	2.31	−44.53	−42.22	2.4	−14.80	−12.50	2.7
**COF-4M**	**III-a**	−0.33	−17.30	−17.63	2.6	−8.80	−9.13	2.6
**COF-5M**	**III-b**	0.07	−25.33	−25.26	2.5	−9.72	−9.65	2.5
**COF-6M**	**III-c**	0.14	−32.31	−32.17	2.6	−21.34	−21.20	2.8
**COF-7M**	**III-d**	−1.70	−16.35	−18.05	3.0	−16.07	−17.77	3.2
**TP COF-1M**	**III-e**	−0.14	−32.26	−32.40	2.4	−12.77	−12.91	2.4
**COF-8M**	**IV-a**	−7.87	−27.56	−35.43	1.8	−26.80	−34.67	2.1
**COF-9M**	**IV-b**	−8.36	−35.77	−44.14	1.7	−30.03	−38.39	2.1
**COF-10M**	**IV-c**	−9.47	−42.97	−52.44	1.8	−41.92	−51.40	2.2
**COF-11M**	**IV-d**	−4.03	−26.84	−30.87	2.1	−28.33	−32.36	2.4
**TP COF-2M**	**IV-e**	0.30	−43.45	−43.15	1.8	−41.17	−40.87	2.1

All energies are given in terms of reaction energies, that is, more negative values indicate more stable products. Interestingly, the formation of monolayers is endothermic for the COFs made from connectors **I** and **II,** while exothermic for the others, except for COF-5M, COF-6M and TP COF-2M. However, all reactions leading to COFs in their bulk forms are exothermic. It is to be noted that COF-1 is the least stable in AB form, which is in contrast with experimental observations [7], however, is in support of our XRD simulation presented in another work [17]. Nevertheless, COF-3M made from connector **I** and linker **d** is more stable in AB stacking, compared with other stacking kinds. Two other linker-**d** made COFs (COF-11M and COF-7M) have AB stacking energies comparable to other stacking forms. For all other COFs, AB stacking is disfavoured.

AA stacking is always energetically disfavoured compared to the structurally close serrated and inclined stacking kinds. This is in contrary to experimental observations for most of the COFs. However, simple rationalisation, in terms of Coulomb energy as well as our XRD simulations presented in this and earlier work [17], supports these results. Hence, we suggest that all the reported 2D COF geometries should be re-examined carefully experimentally because the change in stacking redefines the pore geometry [17]. COFs made of connector-I, except COF-3M, are more stable in serrated forms. The majority of the other COFs is stable in inclined forms (see Table S4 in Supporting Information File 1).

We have calculated the condensation energies of COF-1, COF-5, and COF-8 using first-principles DFT (see “Methods” for computational details) to support our results quantitatively. For simplicity we have used a finite structure instead of a bulk crystal. Their calculated E_c,b_ energies are 7.71, 1.49 and 0.40 kJ•mol^−1^, respectively; hence supporting the endothermic nature of the condensation reaction and is in reasonable agreement with our DFTB results (Table 2).

### Electronic properties

All COFs, including the reference structures, are semiconductors with their band gaps lying between 1.7 eV and 4.0 eV (Table 2 and Table S4 in Supporting Information File 1). The largest band gaps are of the reference structures, while the lowest values are of COFs built from connector **IV**. The band gaps are different for different stacking kinds. This difference can be attributed to the different optimized interlayer distances. Generally, AB, serrated and inclined stacked COFs have band gaps comparable to, or larger than, that of their AA stacked analogues.

**Figure 6:**
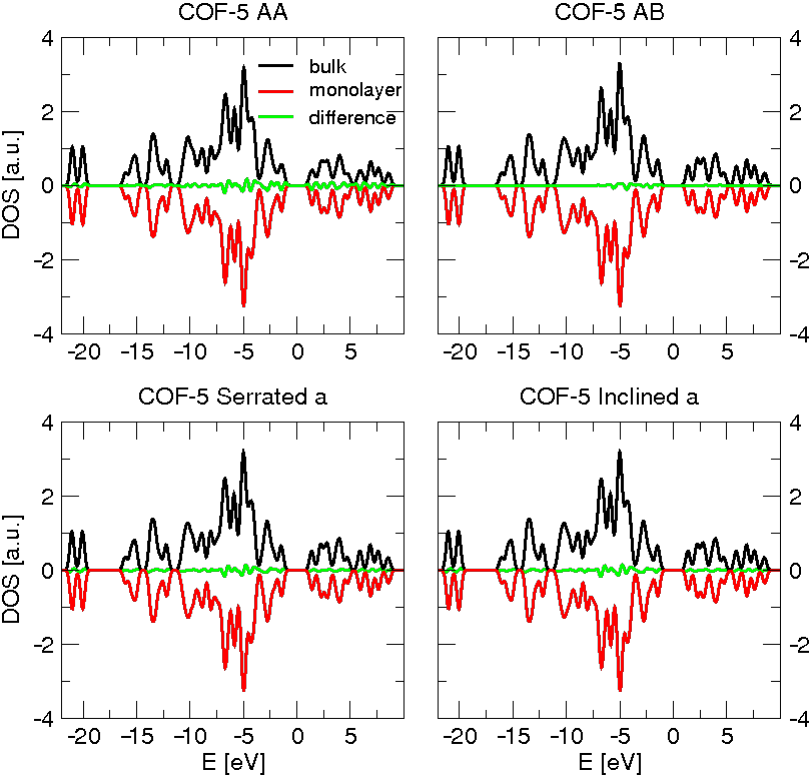
Total densities of states (DOS) (black) of AA (top left), AB (top right), serrated (bottom left), and inclined (bottom right), comparing stacked COF-5 with a monolayer (red) of COF-5. The differences between the TDOS of bulk and monolayer structures are indicated in green. The Fermi level *E*_F_ is shifted to zero.

We have calculated the electronic total density of states (TDOS) and the individual atomic contributions (partial density of states, PDOS). The energy state distributions of COFs and their monolayers are studied and a comparison for COF-5 is shown in Figure 6. In all stacking kinds, negligible differences are found for the densities at the top of valence band and the bottom of conduction band. These slight differences suggest that the weak interaction between the layers or the overlap of π-orbitals does not affect the electronic structure of COFs significantly. Hence, there is almost no difference between the TDOS of AA, AB, serrated and inclined stacking kinds. Therefore, in the following, we discuss only the AA stacked structures.

**Figure 7:**
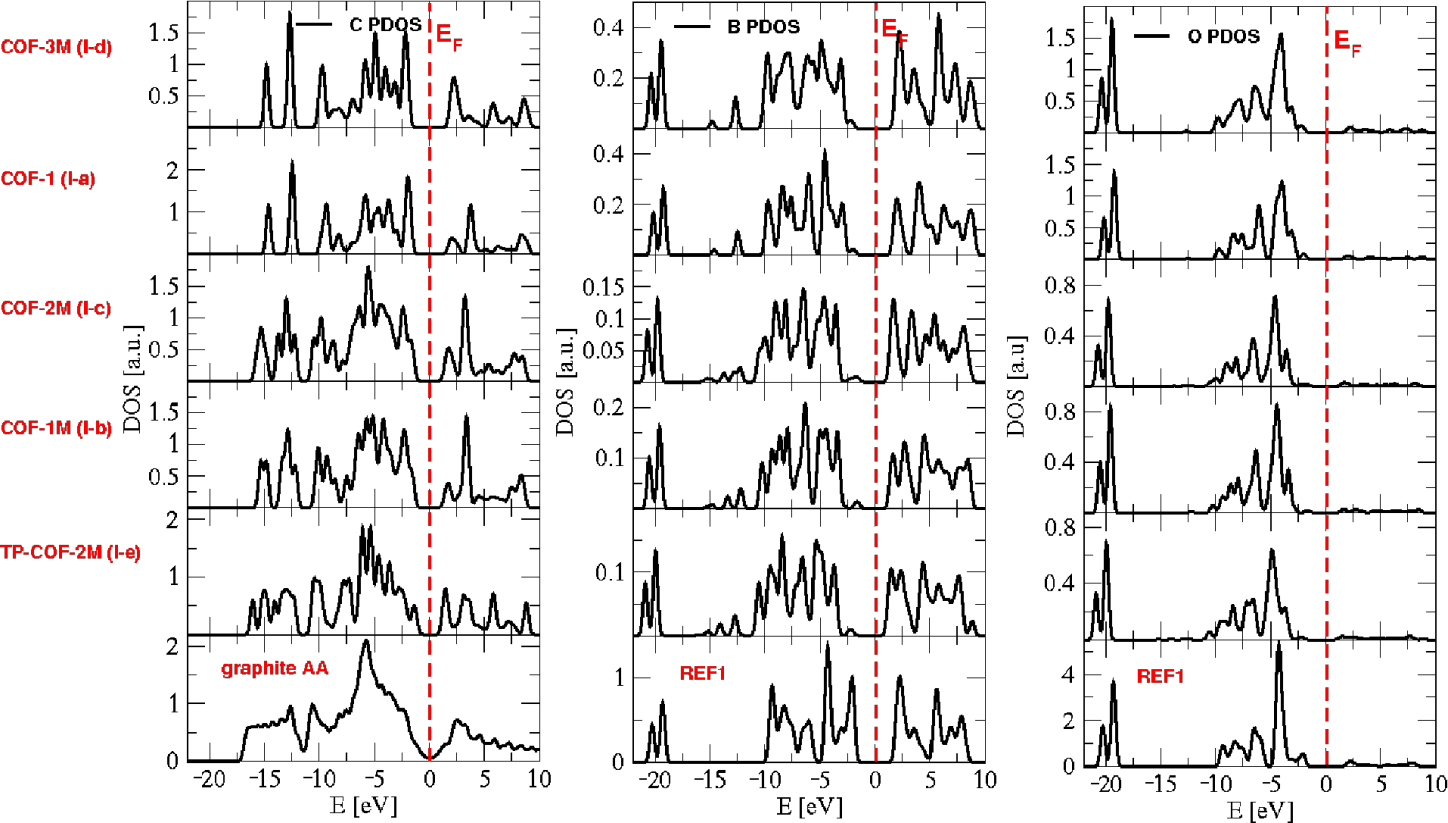
Partial density of states of carbon (left), boron (center), and oxygen (right) atoms of COFs built from type-I connectors and different linkers. The vertical dashed line in each figure indicates the Fermi level *E*_F._

**Figure 8:**
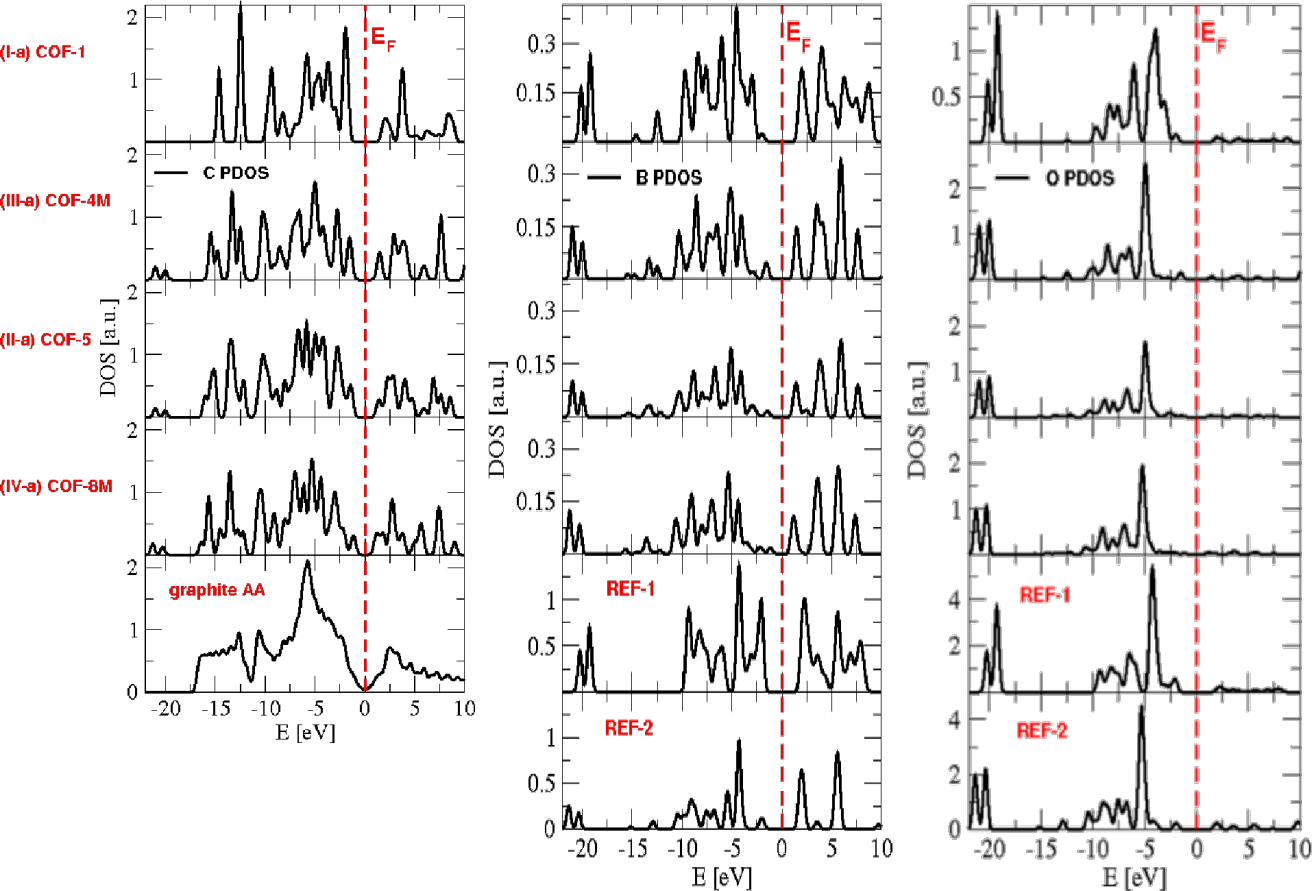
Partial density of states of carbon (left), boron (center), and oxygen (right) atoms of COFs built from type-**a** linkers. The vertical dashed line in each figure indicates the Fermi level *E*_F_.

It is of interest to see how the properties of COFs change depending on their composition of different secondary building units, that is, for different connectors and linkers. PDOS of COFs built from type-**I** connectors and different linkers are plotted in Figure 7. The PDOS of carbon atoms is compared with that of graphite (AA-stacking), while PDOS of boron and oxygen atoms are compared with that of REF-**I**, a structure which is composed solely of connector building blocks. Going from top to bottom of the plots, the number of carbon atoms per unit cell increases. It can be seen that this causes a decrease of the band gap. Figure 8 shows the PDOS of COFs built from type-**a** linkers and different connectors, where the COFs are arranged in the increasing number of carbon atoms in their unit cells from top to the bottom. Again, the C PDOS is compared with that of graphite, while both REF-**I** and REF- **III** are taken in comparison to O and B PDOS. The observed relation between number of carbon atoms and band gap is verified.

## Conclusion

In summary, we have designed 2D COFs of various topologies, by connecting building blocks of different connectivity and performed DFTB calculations to understand their structural, energetic and electronic properties. We have studied each COF in high-symmetry AA and AB as well as low-symmetry inclined and serrated stacking forms. The optimized COF structures exhibit different interlayer separations and band gaps in different kinds of layer stackings; however, the density of states of a single layer is not significantly different from that of a multilayer. The alternate shifted layers in AB, serrated and inclined stackings cause less repulsive orbital interactions within the layers, which result in shorter interlayer separation compared to AA stacking. All the studied COFs show semiconductor-like band gaps. We also have observed that larger number of carbon atoms in the unit cells in COFs causes smaller band gaps and vice versa. Energetic studies reveal that the studied structures are stable; however, notable difference in the layer stacking is observed from experimental observations. This result is also supported by simulated XRDs.

### Methods

We have optimized the atomic positions and the lattice parameters for all studied COFs. All calculations were carried out at the Density Functional Tight-Binding (DFTB) [20–21] level of theory. DFTB is based on a second-order expansion of the Kohn–Sham total energy in the Density-Functional Theory (DFT) with respect to charge density fluctuations. This can be considered as a non-orthogonal tight-binding method parameterized from DFT, which does not require large amounts of empirical parameters, however, maintains all the qualities of DFT. The main idea behind this method is to describe the Hamiltonian eigenstates with an atomic-like basis set and replace the Hamiltonian with a parameterized Hamiltonian matrix whose elements depend only on the internuclear distances and orbital symmetries [21]. While the Hamiltonian matrix elements are calculated using atomic reference densities, the remaining terms to the Kohn–Sham energy are parameterized from DFT reference calculations of a few reference molecules per atom pair. The accuracy is very much improved by the self-consistent charge (SCC) extension. Two computational codes were used: deMonNano code [22] and DFTB+ code [23]. The first code has dispersion correction [24] implemented to account for weak interactions and was used to obtain the layered bulk structure of COFs and their formation energies. The performance for interlayer interactions has been tested previously for graphite [24]. The second code, which can perform calculations using k-points, was used to calculate the electronic properties (band structure and density of states). Band gaps have been calculated as an additional stability indicator. While these quantities are typically strongly underestimated in standard LDA- and GGA-DFT calculations, they are typically in the correct range within the DFTB method. For validation of our method, we have calculated some of the structures using Density Functional Theory (DFT) as implemented in ADF code [25–26].

Periodic boundary conditions were used to represent frameworks of the crystalline solid state. The conjugate–gradient scheme was chosen for the geometry optimization. The atomic force tolerance of 3 × 10^−4^ eV/Å was applied. The optimization, using Γ-point approximation, was performed with the deMonNano code on 2×2×4 supercells. Some of the monolayers were also optimized using the DFTB+ code on elementary unit cells in order to validate the calculations within the Γ-point approximation. The number of k-points has been determined by reaching convergence for the total energy as a function of k-points according to the scheme proposed by Monkhorst and Pack [27]. Band structures were computed along lines between high symmetry points of the Brillouin zone with 50 k-points each along each line. XRD patterns have been simulated using Mercury software [28–29].

We have also performed first-principles DFT calculations at the PBE [30] /DZP [31] level to support our results quantitatively. For simplicity, we have used finite structures instead of bulk crystals.

## Supporting Information

Supporting Information supplies detailed data of calculated COF parameters and crystal information files (CIFs) of some of the studied COF structures.

File 1 Detailed data of calculated COF parameters.

File 2 CIFs for selected structures.

## References

[1] Mueller U, Schubert M, Teich F, Puetter H, Schierle-Arndt K, Pastre J (2006). J Mater Chem.

[2] Yaghi O M, O'Keeffe M, Ockwig N W, Chae H K, Eddaoudi M, Kim J (2003). Nature.

[3] Ockwig N W, Delgado-Friedrichs O, O'Keeffe M, Yaghi O M (2005). Acc Chem Res.

[4] Li H, Eddaoudi M, O'Keeffe M, Yaghi O M (1999). Nature.

[5] Sudik A C, Millward A R, Ockwig N W, Cote A P, Kim J, Yaghi O M (2005). J Am Chem Soc.

[6] Hayashi H, Cote A P, Furukawa H, O'Keeffe M, Yaghi O M (2007). Nat Mater.

[7] Cote A P, Benin A I, Ockwig N W, O'Keeffe M, Matzger A J, Yaghi O M (2005). Science.

[8] Cote A P, El-Kaderi H M, Furukawa H, Hunt J R, Yaghi O M (2007). J Am Chem Soc.

[9] El-Kaderi H M, Hunt J R, Mendoza-Cortes J L, Cote A P, Taylor R E, O'Keeffe M, Yaghi O M (2007). Science.

[10] Hunt J R, Doonan C J, LeVangie J D, Cote A P, Yaghi O M (2008). J Am Chem Soc.

[11] Tilford R W, Mugavero S J, Pellechia P J, Lavigne J J (2008). Adv Mater.

[12] Uribe-Romo F J, Hunt J R, Furukawa H, Klock C, O'Keeffe M, Yaghi O M (2009). J Am Chem Soc.

[13] Wan S, Guo J, Kim J, Ihee H, Jiang D L (2008). Angew Chem.

[14] Wan S, Guo J, Kim J, Ihee H, Jiang D L (2009). Angew Chem.

[15] Tylianakis E, Klontzas E, Froudakis G E (2009). Nanotechnology.

[16] Klontzas E, Tylianakis E, Froudakis G E (2008). J Phys Chem C.

[17] Lukose B, Kuc A, Heine T Chem–Eur J.

[18] Chae H K, Siberio-Perez D Y, Kim J, Go Y, Eddaoudi M, Matzger A J, O'Keeffe M, Yaghi O M (2004). Nature.

[19] Yang Q Y, Zhong C L (2009). Langmuir.

[20] Seifert G, Porezag D, Frauenheim T (1996). Int J Quantum Chem.

[21] Oliveira A, Seifert G, Heine T, Duarte H A (2009). J Braz Chem Soc.

[R22] 22Heine, T.; Rapacioli, M.; Patchkovskii, S.; Frenzel, J.; Koester, A. M.; Calaminici, P.; Escalante, S.; Duarte, H. A.; Flores, R.; Geudtner, G.; Goursot, A.; Reveles, J. U.; Vela, A.; Salahub, D. R. deMon, deMon-nano edn., deMon-nano, 2009.

[23] (2010). DFTB+ - Density Functional based Tight binding (and more).

[24] Zhechkov L, Heine T, Patchkovskii S, Seifert G, Duarte H A (2005). J Chem Theory Comput.

[25] (2009). ADF2009.01, SCM; Theoretical Chemistry.

[26] te Velde G, Bickelhaupt F M, van Gisbergen S J A, Fonseca Guerra C, Baerends E J, Snijders J G, Ziegler T (2001). J Comput Chem.

[27] Monkhorst H J, Pack J D (1976). Phys Rev B.

[28] (2010). Mercury - Crystal Structure Visualisation and Exploration Made Easy.

[29] Macrae C F, Edgington P R, McCabe P, Pidcock E, Shields G P, Taylor R, Towler M, Van de Streek J (2006). J Appl Crystallogr.

[30] Perdew J P, Burke K, Ernzerhof M (1996). Phys Rev Lett.

[31] Van Lenthe E, Baerends E J (2003). J Comput Chem.

