# Supplementary material for: On the reticular construction concept of covalent organic frameworks
Source: Beilstein J Nanotechnol. 2010 Nov 22;1:60–70. doi: 10.3762/bjnano.1.8 (PMC3045923; doi:10.3762/bjnano.1.8)
Supplement: File 1 — Detailed data of calculated COF parameters. [file Beilstein_J_Nanotechnol-01-60-s001.pdf]

**Supporting Information**

for

# On the reticular construction concept of covalent organic frameworks

Binit Lukose[1], Agnieszka Kuc[1], Johannes Frenzel[2], and Thomas Heine*[1]

Address: [1]School of Engineering and Science, Jacobs University Bremen, Research III, Room 61, Campus Ring 1, Bremen 28759, Germany and [2]Lehrstuhl für Theoretische Chemie, Ruhr-Universität Bochum, Bochum 44780, Germany

Email: Thomas Heine - t.heine@jacobs-university.de

* Corresponding author

# Detailed data of calculated COF parameters

# Contents

1. Table S1: Bond lengths and angles of studied COFs
2. Figure S2: C-C bond lengths in connectors and linkers
3. Table S3: Cell parameter *a* [Å], interlayer distance *d* [Å], and mass density *ρ* [g·cm$^{-3}$] for serrated and inclined stacked COFs.
4. Table S4: The calculated energies per bond formed between building blocks for serrated and inclined stacked COFs
5. Cartesian coordinates of all simulated COFs

**Table S1:** The calculated bond lengths and angles of all studied COFs. Corresponding experimental values found from the literature are shown in brackets.

| COF | Building Blocks | C-B | B-O | O-C | ∠OBO |
|---|---|---|---|---|---|
| **COF-1** | **I-a** | 1.498 (1.600) | 1.393 (1.509) | | 120.3* (120.0) |
| **COF-1M** | **I-b** | 1.497 | 1.393 | | 120.3* |
| **COF-2M** | **I-c** | 1.497 | 1.392 | | 120.3* |
| **COF-3M** | **I-d** | 1.496 | 1.392 | | 120.1* |
| **PPy-COF** | **I-e** | 1.498 | 1.393 | | 120.2* (119.0) |
| **COF-5** | **II-a** | 1.496 (1.530) | 1.399 (1.367) | 1.443 (1.384) | 113.5† (113.9) |
| **COF-10** | **II-b** | 1.495 (1.553) | 1.399 (1.465) | 1.443 (1.385) | 113.5† (112.0) |
| **COF-8** | **II-c** | 1.495 (1.548) | 1.399 (1.479) | 1.443 | 113.5† |
| **COF-6** | **II-d** | 1.496 | 1.399 | 1.443 | 113.5† |
| **TP COF** | **II-e** | 1.496 (1.542) | 1.399 (1.396) | 1.444 (1.377) | 113.5† (118.2) |
| **COF-4M** | **III-a** | 1.496 | 1.398 | 1.449 | 113.5† |
| **COF-5M** | **III-b** | 1.496 | 1.398 | 1.449 | 113.6† |
| **COF-6M** | **III-c** | 1.496 | 1.399 | 1.451 | 113.4† |
| **COF-7M** | **III-d** | 1.496 | 1.398 | 1.449 | 113.6† |
| **TP COF-1M** | **III-e** | 1.496 | 1.398 | 1.450 | 113.6† |
| **COF-8M** | **IV-a** | 1.496 | 1.398 | 1.445 | 113.1† |
| **COF-9M** | **IV-b** | 1.495 | 1.398 | 1.444 | 113.1† |
| **COF-10M** | **IV-c** | 1.495 | 1.391 | 1.418 | 112.6† |
| **COF-11M** | **IV-d** | 1.498 | 1.399 | 1.450 | 113.4† |
| **TP COF-2M** | **IV-e** | 1.499 | 1.399 | 1.447 | 113.4† |

*$B_3O_3$ connectivity
†$C_2B_2O$ connectivity

It should be noted that the bond lengths of experimentally known COF-1 are much larger compared to our optimized bond lengths as well as that of other synthesized COFs.

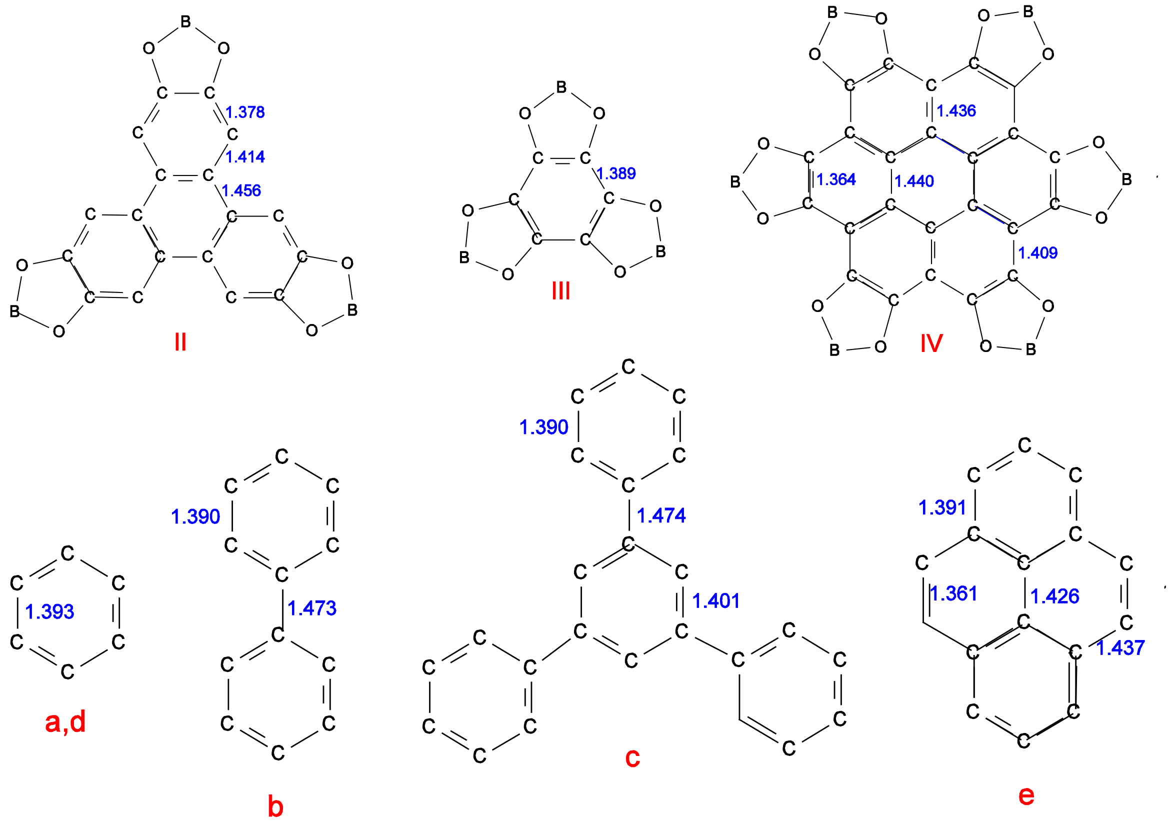


**Figure S2:** Calculated C-C bond lengths in connectors and linkers. Hydrogen atoms are omitted for clarity.

**Table S3:** The calculated unit cell parameters a [Å], interlayer distance d [Å] and mass density $\rho$ [g·cm$^{-3}$] for serrated ($S_a$: serrated armchair, $S_z$: serrated zigzag) and inclined ($I_a$: inclined armchair, $I_z$: inclined zigzag) stacked COFs.

| **COF** | **Building Blocks** | **a** | **d** | | | | **ρ** | | | |
|---|---|---|---|---|---|---|---|---|---|---|
| | | | **$S_a$** | **$S_z$** | **$I_a$** | **$I_z$** | **$S_a$** | **$S_z$** | **$I_a$** | **$I_z$** |
| **COF-1** | **I-a** | 15.02 | | 3.43 | | 3.43 | | 0.97 | | 0.97 |
| **COF-1M** | **I-b** | 22.41 | | 3.41 | | 3.42 | | 0.69 | | 0.69 |
| **COF-2M** | **I-c** | 14.92 | | 3.40 | | 3.39 | | 0.97 | | 0.97 |
| **COF-3M** | **I-d** | 07.47 | | 3.41 | | 3.42 | | 1.57 | | 1.56 |
| **PPy-COF** | **I-e** | 22.32 | | 3.41 | | 3.41 | | 0.86 | | 0.86 |
| **COF-5** | **II-a** | 30.14 | 3.42 | 3.42 | 3.41 | 3.40 | 0.57 | 0.57 | 0.58 | 0.58 |
| **COF-10** | **II-b** | 37.58 | 3.41 | 3.41 | 3.42 | 3.40 | 0.46 | 0.46 | 0.46 | 0.46 |
| **COF-8** | **II-c** | 22.51 | 3.41 | 3.41 | 3.42 | 3.42 | 0.73 | 0.73 | 0.72 | 0.72 |
| **COF-6** | **II-d** | 15.05 | 3.42 | 3.41 | 3.40 | 3.40 | 1.05 | 1.06 | 1.06 | 1.06 |
| **TP COF** | **II-e** | 37.50 | 3.42 | 3.41 | 3.42 | 3.42 | 0.52 | 0.52 | 0.52 | 0.52 |
| **COF-4M** | **III-a** | 21.71 | 3.44 | 3.44 | 3.45 | 3.44 | 0.74 | 0.74 | 0.74 | 0.74 |
| **COF-5M** | **III-b** | 29.15 | 3.43 | 3.42 | 3.43 | 3.43 | 0.56 | 0.56 | 0.56 | 0.56 |
| **COF-6M** | **III-c** | 18.33 | 3.41 | 3.41 | 3.42 | 3.41 | 0.84 | 0.84 | 0.84 | 0.84 |

| | | | | | | | | | | |
|---|---|---|---|---|---|---|---|---|---|---|
| **COF-7M** | **III-d** | 10.83 | 3.44 | 3.43 | 3.40 | 3.44 | 1.31 | 1.31 | 1.32 | 1.31 |
| **TP COF-1M** | **III-e** | 29.05 | 3.43 | 3.42 | 3.43 | 3.42 | 0.66 | 0.67 | 0.66 | 0.66 |
| **COF-8M** | **IV-a** | 17.48 | 3.41 | 3.41 | 3.42 | 3.42 | 1.42 | 1.42 | 1.42 | 1.42 |
| **COF-9M** | **IV-b** | 21.76 | 3.41 | 3.41 | 3.41 | 3.42 | 1.19 | 1.19 | 1.19 | 1.19 |
| **COF-10M** | **IV-c** | 22.54 | 3.40 | 3.40 | 3.40 | 3.40 | 1.28 | 1.28 | 1.28 | 1.28 |
| **COF-11M** | **IV-d** | 15.12 | 3.41 | 3.41 | 3.40 | 3.40 | 1.71 | 1.71 | 1.71 | 1.71 |
| **TP COF-2M** | **IV-e** | 21.73 | 3.40 | 3.40 | 3.40 | 3.40 | 1.37 | 1.37 | 1.37 | 1.37 |
| **REF-I** | **I** | 07.73 | | 3.49 | | 3.45 | | 1.48 | | 1.5 |
| **REF-III** | **III** | 14.45 | 3.48 | | 3.49 | | 1.06 | | 1.06 | |

**Table S4:** The calculated energies [kJ·mol$^{-1}$] per bond formed between building blocks for serrated ($S_a$: serrated armchair, $S_z$: serrated zigzag) and inclined ($I_a$: inclined armchair, $I_z$: inclined zigzag) stacked COFs: $E_{c,b}$ is the condensation energy, $E_{s,b}$ is the stacking energy, and $E_{f,b}$ is the COF formation energy ($E_{f,b} = E_{c,b} + E_{s,b}$). The calculated band gaps, Δ [eV], are given as well.

| **COF** | **$S_a$** | | | **$S_z$** | | | **$I_a$** | | | **$I_z$** | | |
|---|---|---|---|---|---|---|---|---|---|---|---|---|
| | **$E_{s,b}$** | **$E_{f,b}$** | **Δ** | **$E_{s,b}$** | **$E_{f,b}$** | **Δ** | **$E_{s,b}$** | **$E_{f,b}$** | **Δ** | **$E_{s,b}$** | **$E_{f,b}$** | **Δ** |
| **-1** | | | | -28.10 | -19.04 | 3.6 | | | | -27.86 | -18.80 | 3.6 |
| **-1M** | | | | -44.26 | -34.77 | 3.0 | | | | -43.89 | -34.40 | 3.0 |
| **-2M** | | | | -59.67 | -50.11 | 3.0 | | | | -58.33 | -48.77 | 3.0 |
| **-3M** | | | | -26.67 | -19.04 | 4.0 | | | | -25.91 | -18.28 | 4.0 |
| **PPy-** | | | | -59.16 | -50.58 | 2.6 | | | | -58.65 | -50.07 | 2.6 |
| **-5** | -30.52 | -28.41 | 2.7 | -30.51 | -28.40 | 2.6 | -32.75 | -30.64 | 2.6 | -32.97 | -30.86 | 2.6 |
| **-10** | -38.68 | -35.51 | 2.6 | -38.69 | -35.52 | 2.5 | -38.30 | -35.13 | 2.6 | -42.93 | -39.76 | 2.5 |
| **-8** | -46.15 | -43.52 | 2.7 | -46.14 | -43.51 | 2.7 | -45.71 | -43.08 | 2.7 | -45.73 | -43.10 | 2.7 |
| **-6** | -29.78 | -27.93 | 3.0 | -29.78 | -27.93 | 3.0 | -31.17 | -29.32 | 3.0 | -30.90 | -29.05 | 3.0 |
| **TP** | -45.57 | -43.26 | 2.6 | -45.62 | -43.31 | 2.6 | -45.11 | -42.80 | 2.6 | -45.11 | -42.80 | 2.6 |
| **-4M** | -18.11 | -18.44 | 2.8 | -18.13 | -18.46 | 2.8 | -17.69 | -18.02 | 2.8 | -18.80 | -19.13 | 2.8 |
| **-5M** | -26.26 | -26.19 | 2.7 | -26.30 | -26.23 | 2.6 | -25.97 | -25.90 | 2.6 | -26.00 | -25.93 | 2.6 |
| **-6M** | -33.75 | -33.61 | 2.8 | -33.81 | -33.67 | 2.8 | -34.87 | -34.73 | 2.8 | -33.49 | -33.35 | 2.8 |
| **-7M** | -17.24 | -18.94 | 3.2 | -17.26 | -18.96 | 3.1 | -23.33 | -25.03 | 3.2 | -18.66 | -20.36 | 3.1 |
| **TP -1M** | -33.27 | -33.41 | 2.6 | -33.34 | -33.48 | 2.6 | -33.40 | -33.54 | 2.6 | -33.53 | -33.67 | 2.6 |
| **-8M** | -28.97 | -36.84 | 2.1 | -28.95 | -36.82 | 2.1 | -29.71 | -37.58 | 2.1 | -29.83 | -37.70 | 2.1 |
| **-9M** | -37.32 | -45.68 | 2.0 | -37.33 | -45.69 | 2.0 | -36.84 | -45.20 | 2.0 | -37.06 | -45.42 | 2.0 |
| **-10M** | -45.12 | -54.59 | 2.1 | -45.13 | -54.60 | 2.1 | -44.91 | -54.38 | 2.1 | -44.95 | -54.42 | 2.1 |
| **-11M** | -28.31 | -32.34 | 2.4 | -28.34 | -32.37 | 2.4 | -28.16 | -32.19 | 2.4 | -28.30 | -32.33 | 2.4 |
| **TP -2M** | -45.00 | -44.70 | 2.0 | -45.05 | -44.75 | 2.0 | -44.95 | -44.65 | 2.0 | -44.96 | -44.66 | 2.0 |

# Cartesian coordinates of COF structures

## COF-1

### Layer I AA, AB, inclined zigzag, and serrated zigzag

| | | | | | | | |
|---|---|---|---|---|---|---|---|
| C | 9.619348 | 10.595180 | 0.794835 | O | 7.077028 | 12.043170 | 0.794935 |
| C | 9.578448 | 12.001080 | 0.795055 | B | 20.837220 | 12.738170 | 0.794775 |
| C | 10.816200 | 12.669210 | 0.795125 | O | 13.344850 | 1.168948 | 0.794905 |
| C | 12.018110 | 11.975350 | 0.794975 | C | 19.541290 | 11.986090 | 0.794485 |
| C | 12.058630 | 10.569420 | 0.794755 | C | 19.501730 | 10.580210 | 0.794805 |
| C | 10.821120 | 9.901537 | 0.794675 | C | 18.300440 | 9.885317 | 0.794835 |
| B | 13.354240 | 9.816797 | 0.794755 | C | 17.062330 | 10.552590 | 0.794485 |
| O | 14.561420 | 10.511340 | 0.794555 | C | 17.101900 | 11.958400 | 0.794135 |
| B | 15.762870 | 9.806707 | 0.794585 | C | 18.303200 | 12.653190 | 0.794105 |
| O | 15.760730 | 8.414117 | 0.794805 | H | 10.802260 | 0.793766 | 0.793905 |
| B | 14.549710 | 7.726126 | 0.794905 | H | 16.154750 | 12.522810 | 0.793925 |
| O | 13.344650 | 8.424098 | 0.794875 | H | 20.448893 | 10.015360 | 0.795205 |
| C | 14.545390 | 6.227821 | 0.795005 | H | 18.317090 | 8.782715 | 0.795225 |
| C | 15.742780 | 5.490328 | 0.795455 | H | 16.706014 | 3.565474 | 0.795925 |
| C | 15.743230 | 4.102874 | 0.795535 | H | 16.705240 | 6.028351 | 0.795805 |
| C | 14.546280 | 3.364655 | 0.795125 | H | 12.385610 | 3.564683 | 0.794525 |
| C | 13.348490 | 4.102057 | 0.794755 | H | 12.384980 | 6.026808 | 0.794405 |
| C | 13.348090 | 5.489797 | 0.794675 | H | 12.964940 | 12.540800 | 0.795055 |
| B | 14.550290 | 1.866363 | 0.795105 | H | 3.348885 | 0.809891 | 0.795325 |
| O | 0.793766 | 1.177789 | 0.795225 | H | 10.805050 | 8.798943 | 0.794505 |
| B | 8.279047 | 12.747040 | 0.795055 | H | 8.672658 | 10.029870 | 0.794755 |

Transformation Matrix

| | | | | |
|---|---|---|---|---|
| **a** | 14.967120 | 0.000000 | 0.000000 | |
| **b** | 7.483560 | 12.961900 | 0.000000 | |
| **c** | 0.000000 | 0.000000 | 3.505888 | AA stacking |
| **c** | 1.204480 | 0.695407 | 3.432429 | Inclined zigzag stacking |

### Layer II AB stacking

| | | | | | | | |
|---|---|---|---|---|---|---|---|
| C | 17.10291 | 14.91582 | 3.922080 | O | 14.56059 | 16.36381 | 3.922180 |
| C | 17.06201 | 16.32171 | 3.922300 | B | 28.32078 | 17.05881 | 3.922020 |
| C | 18.29976 | 16.98985 | 3.922370 | O | 20.82841 | 5.489583 | 3.922150 |
| C | 19.50167 | 16.29599 | 3.922220 | C | 27.02485 | 16.30672 | 3.921730 |
| C | 19.54219 | 14.89005 | 3.922000 | C | 26.98529 | 14.90084 | 3.922050 |
| C | 18.30468 | 14.22217 | 3.921920 | C | 25.78400 | 14.20595 | 3.922080 |
| B | 20.83780 | 14.13743 | 3.922000 | C | 24.54589 | 14.87323 | 3.921730 |
| O | 22.04498 | 14.83198 | 3.921800 | C | 24.58546 | 16.27904 | 3.921380 |
| B | 23.24643 | 14.12734 | 3.921830 | C | 25.78676 | 16.97383 | 3.921350 |
| O | 23.24429 | 12.73475 | 3.922050 | H | 18.28582 | 5.114401 | 3.921150 |
| B | 22.03327 | 12.04676 | 3.922150 | H | 23.63831 | 16.84344 | 3.921170 |
| O | 20.82821 | 12.74473 | 3.922120 | H | 27.93245 | 14.33599 | 3.922450 |
| C | 22.02895 | 10.54846 | 3.922250 | H | 25.80065 | 13.10335 | 3.922470 |
| C | 23.22634 | 9.810963 | 3.922700 | H | 24.18958 | 7.886109 | 3.923170 |
| C | 23.22679 | 8.423510 | 3.922780 | H | 24.18880 | 10.34899 | 3.923050 |
| C | 22.02984 | 7.685290 | 3.922370 | H | 19.86917 | 7.885318 | 3.921770 |
| C | 20.83205 | 8.422692 | 3.922000 | H | 19.86854 | 10.34744 | 3.921650 |
| C | 20.83165 | 9.810432 | 3.921920 | H | 20.44850 | 16.86143 | 3.922300 |

| | | | | | | | |
|---|---|---|---|---|---|---|---|
| B | 22.03385 | 6.186998 | 3.922350 | H | 10.83245 | 5.130527 | 3.922570 |
| O | 8.277327 | 5.498425 | 3.922470 | H | 18.28861 | 13.11958 | 3.921750 |
| B | 15.76261 | 17.06767 | 3.922300 | H | 16.15622 | 14.35051 | 3.922000 |

**c** 0.00000 0.00000 6.25449 AB stacking

### Layer II serrated zigzag

| | | | | | | | |
|---|---|---|---|---|---|---|---|
| C | 10.82383 | 11.29059 | 4.22064 | O | 8.28151 | 12.73858 | 4.220743 |
| C | 10.78293 | 12.69649 | 4.22086 | B | 14.55814 | 0.47168 | 4.220583 |
| C | 4.53712 | 0.40272 | 4.22093 | O | 14.54933 | 1.86436 | 4.220713 |
| C | 13.22259 | 12.67076 | 4.22078 | C | 20.74577 | 12.68150 | 4.220293 |
| C | 13.26311 | 11.26483 | 4.22056 | C | 20.70621 | 11.27562 | 4.220613 |
| C | 12.02560 | 10.59694 | 4.22048 | C | 19.50492 | 10.58072 | 4.220643 |
| B | 14.55872 | 10.51220 | 4.22056 | C | 18.26681 | 11.24800 | 4.220293 |
| O | 15.76590 | 11.20675 | 4.22036 | C | 18.30638 | 12.65381 | 4.219943 |
| B | 16.96735 | 10.50211 | 4.22039 | C | 12.02412 | 0.38670 | 4.219913 |
| O | 16.96521 | 9.109524 | 4.22061 | H | 12.00674 | 1.48917 | 4.219713 |
| B | 15.75419 | 8.421534 | 4.22071 | H | 9.87567 | 0.25632 | 4.219733 |
| O | 14.54913 | 9.119505 | 4.22068 | H | 6.68625 | 10.71077 | 4.221013 |
| C | 15.74987 | 6.923228 | 4.22081 | H | 19.52157 | 9.47812 | 4.221033 |
| C | 16.94726 | 6.185735 | 4.22126 | H | 2.94337 | 4.26088 | 4.221733 |
| C | 16.94771 | 4.798281 | 4.22134 | H | 17.90972 | 6.72376 | 4.221613 |
| C | 15.75076 | 4.060062 | 4.22093 | H | 13.59009 | 4.26009 | 4.220333 |
| C | 14.55297 | 4.797464 | 4.22056 | H | 13.58946 | 6.72222 | 4.220213 |
| C | 14.55257 | 6.185204 | 4.22048 | H | 6.68586 | 0.27431 | 4.220863 |
| B | 15.75477 | 2.561770 | 4.22091 | H | 4.55336 | 1.50530 | 4.221133 |
| O | 1.99824 | 1.873196 | 4.22103 | H | 12.00953 | 9.49435 | 4.220313 |
| B | 1.99996 | 0.480550 | 4.22086 | H | 9.87713 | 10.72528 | 4.220563 |

**c** 0.00000 0.00000 6.85162 serrated zigzag stacking

## COF-1M

### Layer 1 AA, AB, inclined zigzag, and serrated zigzag

| | | | | | | | |
|---|---|---|---|---|---|---|---|
| C | 13.308650 | 18.473020 | 0.793916 | C | 30.716500 | 18.434470 | 0.793956 |
| C | 13.353690 | 17.065480 | 0.793986 | C | 29.475080 | 19.099230 | 0.794056 |
| C | 14.555950 | 16.375820 | 0.794016 | C | 28.276480 | 18.403170 | 0.794086 |
| C | 15.780570 | 17.061520 | 0.793986 | C | 28.257560 | 16.999700 | 0.794016 |
| C | 15.750000 | 18.464710 | 0.793916 | C | 29.488090 | 16.324470 | 0.793886 |
| C | 14.544160 | 19.149250 | 0.793886 | C | 30.683890 | 17.026430 | 0.793856 |
| C | 17.052200 | 16.327260 | 0.794016 | C | 26.985690 | 16.265890 | 0.794036 |
| C | 17.070910 | 14.923690 | 0.793956 | C | 25.761130 | 16.951880 | 0.794016 |
| C | 18.269480 | 14.227490 | 0.793986 | C | 24.558700 | 16.262460 | 0.794036 |
| C | 19.510870 | 14.892130 | 0.794086 | C | 24.513430 | 14.854960 | 0.794086 |
| C | 19.478510 | 16.300090 | 0.794156 | C | 25.748740 | 14.178540 | 0.794116 |
| C | 18.282860 | 17.002340 | 0.794116 | C | 26.954810 | 14.862670 | 0.794086 |
| B | 20.802320 | 14.134430 | 0.794116 | H | 29.494620 | 15.220760 | 0.793836 |
| O | 22.012470 | 14.824880 | 0.794116 | H | 31.634540 | 16.467970 | 0.793766 |
| B | 23.211630 | 14.115310 | 0.794086 | H | 18.251610 | 0.7937660 | 0.794136 |
| O | 23.204290 | 12.722200 | 0.794056 | H | 27.317800 | 18.950490 | 0.794186 |
| B | 21.990370 | 12.038150 | 0.794056 | H | 25.756300 | 13.076080 | 0.794156 |
| O | 20.787410 | 12.741060 | 0.794086 | H | 27.907290 | 14.304970 | 0.794116 |
| C | 21.982510 | 10.540880 | 0.794056 | H | 25.767000 | 18.055760 | 0.793956 |
| C | 20.781510 | 9.8055030 | 0.794186 | H | 23.613390 | 16.829830 | 0.794016 |

| | | | | | | | |
|---|---|---|---|---|---|---|---|
| C | 20.775990 | 8.4189450 | 0.794216 | H | 24.137390 | 7.8620570 | 0.793916 |
| C | 21.977800 | 7.6939080 | 0.794116 | H | 24.143150 | 10.339540 | 0.793856 |
| C | 23.181970 | 8.4150640 | 0.793986 | H | 19.820850 | 10.346510 | 0.794286 |
| C | 23.180960 | 9.8012530 | 0.793956 | H | 19.818810 | 7.8691800 | 0.794286 |
| C | 21.977530 | 6.2254730 | 0.794116 | H | 24.142280 | 3.5794100 | 0.794116 |
| C | 20.775510 | 5.5006680 | 0.794086 | H | 24.137150 | 6.0568960 | 0.794136 |
| C | 20.780750 | 4.1141150 | 0.794056 | H | 19.818420 | 6.0505690 | 0.794086 |
| C | 21.981650 | 3.3785580 | 0.794056 | H | 19.819910 | 3.5732960 | 0.794036 |
| C | 23.180230 | 4.1179380 | 0.794116 | H | 16.702580 | 19.022280 | 0.793886 |
| C | 23.181600 | 5.5041230 | 0.794116 | H | 3.3468080 | 0.8438770 | 0.793806 |
| B | 21.989840 | 1.8812640 | 0.794016 | H | 12.408250 | 16.498420 | 0.794016 |
| O | 0.7937660 | 1.1976820 | 0.793986 | H | 14.561560 | 15.271920 | 0.794056 |
| B | 12.006670 | 19.212400 | 0.793936 | H | 20.429200 | 16.858450 | 0.794236 |
| O | 10.807840 | 18.502380 | 0.793886 | H | 18.289510 | 18.105990 | 0.794156 |
| B | 32.007720 | 19.192370 | 0.793956 | H | 16.112210 | 14.376520 | 0.793886 |
| O | 20.787130 | 1.1779250 | 0.794016 | H | 18.250840 | 13.125110 | 0.793936 |

Transformation Matrix

| | | | | |
|---|---|---|---|---|
| **a** | 22.410240 | 0.000000 | 0.000000 | |
| **b** | 11.205120 | 19.407830 | 0.000000 | |
| **c** | 0.000000 | 0.000000 | 3.490500 | AA |
| **c** | 1.199740 | 0.692670 | 3.41658 | inclined zigzag |

## Layer II AB stacking

| | | | | | | | |
|---|---|---|---|---|---|---|---|
| C | 24.51377 | 24.94230 | 3.830658 | C | 41.92162 | 24.90375 | 3.830698 |
| C | 24.55881 | 23.53476 | 3.830728 | C | 40.68020 | 25.56851 | 3.830798 |
| C | 25.76107 | 22.84510 | 3.830758 | C | 39.48160 | 24.87245 | 3.830828 |
| C | 26.98569 | 23.53080 | 3.830728 | C | 39.46268 | 23.46898 | 3.830758 |
| C | 26.95512 | 24.93399 | 3.830658 | C | 40.69321 | 22.79375 | 3.830628 |
| C | 25.74928 | 25.61853 | 3.830628 | C | 41.88901 | 23.49571 | 3.830598 |
| C | 28.25732 | 22.79654 | 3.830758 | C | 38.19081 | 22.73517 | 3.830778 |
| C | 28.27603 | 21.39297 | 3.830698 | C | 36.96625 | 23.42116 | 3.830758 |
| C | 29.47460 | 20.69677 | 3.830728 | C | 35.76382 | 22.73174 | 3.830778 |
| C | 30.71599 | 21.36141 | 3.830828 | C | 35.71855 | 21.32424 | 3.830828 |
| C | 30.68363 | 22.76937 | 3.830898 | C | 36.95386 | 20.64782 | 3.830858 |
| C | 29.48798 | 23.47162 | 3.830858 | C | 38.15993 | 21.33195 | 3.830828 |
| B | 32.00744 | 20.60371 | 3.830858 | H | 40.69974 | 21.69004 | 3.830578 |
| O | 33.21759 | 21.29416 | 3.830858 | H | 42.83966 | 22.93725 | 3.830508 |
| B | 34.41675 | 20.58459 | 3.830828 | H | 29.45673 | 7.263045 | 3.830878 |
| O | 34.40941 | 19.19148 | 3.830798 | H | 38.52292 | 25.41977 | 3.830928 |
| B | 33.19549 | 18.50743 | 3.830798 | H | 36.96142 | 19.54536 | 3.830898 |
| O | 31.99253 | 19.21034 | 3.830828 | H | 39.11241 | 20.77425 | 3.830858 |
| C | 33.18763 | 17.01016 | 3.830798 | H | 36.97212 | 24.52504 | 3.830698 |
| C | 31.98663 | 16.27478 | 3.830928 | H | 34.81851 | 23.29911 | 3.830758 |
| C | 31.98111 | 14.88822 | 3.830958 | H | 35.34251 | 14.33134 | 3.830658 |
| C | 33.18292 | 14.16319 | 3.830858 | H | 35.34827 | 16.80882 | 3.830598 |
| C | 34.38709 | 14.88434 | 3.830728 | H | 31.02597 | 16.81579 | 3.831028 |
| C | 34.38608 | 16.27053 | 3.830698 | H | 31.02393 | 14.33846 | 3.831028 |
| C | 33.18265 | 12.69475 | 3.830858 | H | 35.34740 | 10.04869 | 3.830858 |
| C | 31.98063 | 11.96995 | 3.830828 | H | 35.34227 | 12.52618 | 3.830878 |
| C | 31.98587 | 10.58340 | 3.830798 | H | 31.02354 | 12.51985 | 3.830828 |
| C | 33.18677 | 9.847837 | 3.830798 | H | 31.02503 | 10.04258 | 3.830778 |
| C | 34.38535 | 10.58722 | 3.830858 | H | 27.90770 | 25.49156 | 3.830628 |
| C | 34.38672 | 11.97340 | 3.830858 | H | 14.55193 | 7.313156 | 3.830548 |
| B | 33.19496 | 8.350543 | 3.830758 | H | 23.61337 | 22.96770 | 3.830758 |
| O | 11.99889 | 7.666961 | 3.830728 | H | 25.76668 | 21.74120 | 3.830798 |
| B | 23.21179 | 25.68168 | 3.830678 | H | 31.63432 | 23.32773 | 3.830978 |

| | | | | | | | |
|---|---|---|---|---|---|---|---|
| O | 22.01296 | 24.97166 | 3.830628 | H | 29.49463 | 24.57527 | 3.830898 |
| B | 43.21284 | 25.66165 | 3.830698 | H | 27.31733 | 20.84580 | 3.830628 |
| O | 31.99225 | 7.647204 | 3.830758 | H | 29.45596 | 19.59439 | 3.830678 |
| **c** | 0.00000 | 0.00000 | 6.073485 | AB | | | |

## Layer II serrated zigzag

| | | | | | | | |
|---|---|---|---|---|---|---|---|
| C | 14.50839 | 19.165690 | 4.207076 | C | 31.91624 | 19.127140 | 4.207116 |
| C | 14.55343 | 17.758150 | 4.207146 | C | 19.46970 | 0.384070 | 4.207216 |
| C | 15.75569 | 17.068490 | 4.207176 | C | 29.47622 | 19.095840 | 4.207246 |
| C | 16.98031 | 17.754190 | 4.207146 | C | 29.45730 | 17.692370 | 4.207176 |
| C | 16.94974 | 19.157380 | 4.207076 | C | 30.68783 | 17.017140 | 4.207046 |
| C | 4.53878 | 0.434090 | 4.207046 | C | 31.88363 | 17.719100 | 4.207016 |
| C | 18.25194 | 17.019930 | 4.207176 | C | 28.18543 | 16.958560 | 4.207196 |
| C | 18.27065 | 15.616360 | 4.207116 | C | 26.96087 | 17.644550 | 4.207176 |
| C | 19.46922 | 14.920160 | 4.207146 | C | 25.75844 | 16.955130 | 4.207196 |
| C | 20.71061 | 15.584800 | 4.207246 | C | 25.71317 | 15.547630 | 4.207246 |
| C | 20.67825 | 16.992760 | 4.207316 | C | 26.94848 | 14.871210 | 4.207276 |
| C | 19.48260 | 17.695010 | 4.207276 | C | 28.15455 | 15.555340 | 4.207246 |
| B | 22.00206 | 14.827100 | 4.207276 | H | 30.69436 | 15.913430 | 4.206996 |
| O | 23.21221 | 15.517550 | 4.207276 | H | 10.42404 | 17.160640 | 4.206926 |
| B | 24.41137 | 14.807980 | 4.207246 | H | 19.45135 | 1.486436 | 4.207296 |
| O | 24.40403 | 13.414870 | 4.207216 | H | 17.31242 | 0.235330 | 4.207346 |
| B | 23.19011 | 12.730820 | 4.207216 | H | 26.95604 | 13.768750 | 4.207316 |
| O | 21.98715 | 13.433730 | 4.207246 | H | 29.10703 | 14.997640 | 4.207276 |
| C | 23.18225 | 11.233550 | 4.207216 | H | 26.96674 | 18.748430 | 4.207116 |
| C | 21.98125 | 10.498170 | 4.207346 | H | 24.81313 | 17.522500 | 4.207176 |
| C | 21.97573 | 9.111615 | 4.207376 | H | 25.33713 | 8.554728 | 4.207076 |
| C | 23.17754 | 8.386579 | 4.207276 | H | 25.34289 | 11.032210 | 4.207016 |
| C | 24.38171 | 9.107734 | 4.207146 | H | 21.02059 | 11.039180 | 4.207446 |
| C | 24.38070 | 10.493920 | 4.207116 | H | 21.01855 | 8.561851 | 4.207446 |
| C | 23.17727 | 6.918143 | 4.207276 | H | 2.93178 | 4.272080 | 4.207276 |
| C | 21.97525 | 6.193338 | 4.207246 | H | 25.33689 | 6.749566 | 4.207296 |
| C | 21.98049 | 4.806785 | 4.207216 | H | 21.01816 | 6.743239 | 4.207246 |
| C | 23.18139 | 4.071228 | 4.207216 | H | 21.01965 | 4.265966 | 4.207196 |
| C | 24.37997 | 4.810608 | 4.207276 | H | 6.69720 | 0.307120 | 4.207046 |
| C | 24.38134 | 6.196793 | 4.207276 | H | 4.54654 | 1.536547 | 4.206966 |
| B | 23.18958 | 2.573934 | 4.207176 | H | 13.60799 | 17.191090 | 4.207176 |
| O | 1.99350 | 1.890352 | 4.207146 | H | 15.76130 | 15.964590 | 4.207216 |
| B | 2.00129 | 0.497240 | 4.207096 | H | 21.62894 | 17.551120 | 4.207396 |
| O | 12.00758 | 19.195050 | 4.207046 | H | 19.48925 | 18.798660 | 4.207316 |
| B | 22.00234 | 0.477210 | 4.207116 | H | 17.31195 | 15.069190 | 4.207046 |
| O | 21.98687 | 1.870595 | 4.207176 | H | 19.45058 | 13.817780 | 4.207096 |
| **c** | 0.00000 | 0.00000 | 6.82632 | serrated zigzag | | | |

# COF-2M

## Layer I AA, AB, inclined zigzag, and serrated zigzag

| | | | | | | | |
|---|---|---|---|---|---|---|---|
| C | 11.63233 | 6.210753 | 0.7964430 | C | 15.34072 | 12.66828 | 0.7999130 |
| C | 11.65635 | 7.613939 | 0.7969330 | B | 6.679040 | 3.364680 | 0.7965130 |
| C | 10.42911 | 8.294092 | 0.7959630 | O | 5.473729 | 4.063492 | 0.7958130 |
| C | 9.230199 | 7.598482 | 0.7947130 | B | 5.483906 | 5.456936 | 0.7949830 |

| | | | | | | | |
|---|---|---|---|---|---|---|---|
| C | 9.191589 | 6.190721 | 0.7944630 | O | 6.691520 | 6.151705 | 0.7942130 |
| C | 10.43006 | 5.520183 | 0.7952130 | B | 7.892957 | 5.445951 | 0.7944430 |
| C | 12.92817 | 8.352868 | 0.7981430 | O | 7.890639 | 4.052802 | 0.7953330 |
| C | 12.94053 | 9.753651 | 0.7989330 | H | 15.71660 | 5.673431 | 0.7978830 |
| C | 14.14200 | 10.47344 | 0.7995930 | H | 5.097457 | 8.180507 | 0.7954630 |
| C | 15.34868 | 9.762103 | 0.7995430 | H | 2.940189 | 4.434942 | 0.7955330 |
| C | 15.37087 | 8.361876 | 0.7987630 | H | 4.513570 | 1.667730 | 0.7984630 |
| C | 14.15196 | 7.672185 | 0.7981130 | H | 11.97939 | 12.11415 | 0.7994830 |
| C | 16.64636 | 7.629460 | 0.7979830 | H | 16.29746 | 12.11740 | 0.8004930 |
| C | 16.67207 | 6.226597 | 0.7973630 | H | 8.836130 | 1.671500 | 0.7994130 |
| C | 17.87467 | 5.537436 | 0.7961330 | H | 8.281958 | 8.161122 | 0.7939330 |
| C | 4.189492 | 6.208918 | 0.7954930 | H | 12.58784 | 5.657948 | 0.7971830 |
| C | 19.07268 | 7.616718 | 0.7960630 | H | 10.42946 | 9.397904 | 0.7962930 |
| C | 17.87288 | 8.311345 | 0.7973630 | H | 11.98042 | 10.30233 | 0.7988130 |
| C | 14.13856 | 11.94442 | 0.7997130 | H | 16.30454 | 10.31815 | 0.7999130 |
| C | 12.93522 | 12.66636 | 0.7993630 | H | 14.15703 | 6.566486 | 0.7973130 |
| C | 5.475680 | 1.129270 | 0.7987630 | H | 10.44329 | 4.417712 | 0.7948630 |
| C | 6.675010 | 1.867690 | 0.7983630 | H | 17.87189 | 9.415222 | 0.7978830 |
| C | 7.875200 | 1.130860 | 0.7992630 | | | | |

Transformation matrix

| | | | | |
|---|---|---|---|---|
| **a** | 14.922830 | 0.000000 | 0.000000 | |
| **b** | 7.461410 | 12.923540 | 0.000000 | |
| **c** | 0.000000 | 0.000000 | 3.474490 | AA |
| **c** | 1.221360 | 0.705152 | 3.392639 | inclined zigzag |

## Layer II AB stacking

| | | | | | | | |
|---|---|---|---|---|---|---|---|
| C | 19.09374 | 10.51860 | 3.913061 | C | 22.80214 | 16.97613 | 3.916531 |
| C | 19.11777 | 11.92179 | 3.913551 | B | 14.14046 | 7.672530 | 3.913131 |
| C | 17.89052 | 12.60194 | 3.912581 | O | 12.93514 | 8.371342 | 3.912431 |
| C | 16.69161 | 11.90633 | 3.911331 | B | 12.94532 | 9.764786 | 3.911601 |
| C | 16.65300 | 10.49857 | 3.911081 | O | 14.15294 | 10.45955 | 3.910831 |
| C | 17.89148 | 9.828033 | 3.911831 | B | 15.35437 | 9.753801 | 3.911061 |
| C | 20.38959 | 12.66072 | 3.914761 | O | 15.35205 | 8.360652 | 3.911951 |
| C | 20.40195 | 14.06150 | 3.915551 | H | 23.17801 | 9.981281 | 3.914501 |
| C | 21.60341 | 14.78129 | 3.916211 | H | 12.55887 | 12.48836 | 3.912081 |
| C | 22.81009 | 14.06995 | 3.916161 | H | 10.40160 | 8.742792 | 3.912151 |
| C | 22.83228 | 12.66973 | 3.915381 | H | 11.97499 | 5.975580 | 3.915081 |
| C | 21.61337 | 11.98003 | 3.914731 | H | 19.44081 | 16.42200 | 3.916101 |
| C | 24.10777 | 11.93731 | 3.914601 | H | 23.75887 | 16.42525 | 3.917111 |
| C | 24.13348 | 10.53445 | 3.913981 | H | 16.29754 | 5.979350 | 3.916031 |
| C | 25.33608 | 9.845285 | 3.912751 | H | 15.74337 | 12.46897 | 3.910551 |
| C | 11.65091 | 10.51677 | 3.912111 | H | 20.04926 | 9.965797 | 3.913801 |
| C | 26.53409 | 11.92457 | 3.912681 | H | 17.89087 | 13.70575 | 3.912911 |
| C | 25.33429 | 12.61919 | 3.913981 | H | 19.44184 | 14.61018 | 3.915431 |
| C | 21.59998 | 16.25227 | 3.916331 | H | 23.76595 | 14.62600 | 3.916531 |
| C | 20.39664 | 16.97421 | 3.915981 | H | 21.61844 | 10.87434 | 3.913931 |
| C | 12.93709 | 5.437120 | 3.915381 | H | 17.90471 | 8.725562 | 3.911481 |
| C | 14.13643 | 6.175540 | 3.914981 | H | 25.33331 | 13.72307 | 3.914501 |
| C | 15.33661 | 5.438710 | 3.915881 | | | | |

| | | | | |
|---|---|---|---|---|
| **c** | 0.00000 | 0.00000 | 6.23324 | AB |

## Layer II serrated zigzag

| | | | | | | | |
|---|---|---|---|---|---|---|---|
| C | 12.85369 | 6.915905 | 4.192503 | C | 9.10067 | 0.449890 | 4.195973 |
| C | 12.87771 | 8.319091 | 4.192993 | B | 7.90040 | 4.069832 | 4.192573 |
| C | 11.65047 | 8.999244 | 4.192023 | O | 6.69509 | 4.768644 | 4.191873 |

| | | | | | | | |
|---|---|---|---|---|---|---|---|
| C | 10.45156 | 8.303634 | 4.190773 | B | 6.70527 | 6.162088 | 4.191043 |
| C | 10.41295 | 6.895873 | 4.190523 | O | 7.91288 | 6.856857 | 4.190273 |
| C | 11.65142 | 6.225335 | 4.191273 | B | 9.11432 | 6.151103 | 4.190503 |
| C | 14.14953 | 9.058020 | 4.194203 | O | 9.11199 | 4.757954 | 4.191393 |
| C | 14.16189 | 10.458800 | 4.194993 | H | 16.93796 | 6.378583 | 4.193943 |
| C | 15.36336 | 11.178590 | 4.195653 | H | 6.31882 | 8.885658 | 4.191523 |
| C | 16.57004 | 10.467250 | 4.195603 | H | 4.16155 | 5.140094 | 4.191593 |
| C | 16.59223 | 9.067027 | 4.194823 | H | 5.73493 | 2.372882 | 4.194523 |
| C | 15.37332 | 8.377337 | 4.194173 | H | 13.20075 | 12.819300 | 4.195543 |
| C | 17.86772 | 8.334612 | 4.194043 | H | 17.51882 | 12.822550 | 4.196553 |
| C | 17.89343 | 6.931749 | 4.193423 | H | 10.05749 | 2.376652 | 4.195473 |
| C | 4.17320 | 6.242588 | 4.192193 | H | 9.50332 | 8.866274 | 4.189993 |
| C | 5.41085 | 6.914070 | 4.191553 | H | 13.80920 | 6.363100 | 4.193243 |
| C | 5.37121 | 8.321870 | 4.192123 | H | 11.65082 | 10.103060 | 4.192353 |
| C | 19.09424 | 9.016497 | 4.193423 | H | 13.20178 | 11.007480 | 4.194873 |
| C | 15.35992 | 12.649570 | 4.195773 | H | 17.52590 | 11.023300 | 4.195973 |
| C | 6.69517 | 0.447970 | 4.195423 | H | 15.37839 | 7.271638 | 4.193373 |
| C | 6.69704 | 1.834422 | 4.194823 | H | 11.66465 | 5.122864 | 4.190923 |
| C | 7.89637 | 2.572842 | 4.194423 | H | 19.09325 | 10.120370 | 4.193943 |
| C | 9.09656 | 1.836012 | 4.195323 | | | | |
| **c** | 0.00000 | 0.00000 | 6.79212 | serrated zigzag | | | |

# COF-3M

## Layer I AA, AB, inclined zigzag, and serrated zigzag

| | | | | |
|---|---|---|---|---|
| C | 5.821692 | 5.488254 | 0.793766 | |
| C | 5.859315 | 4.085139 | 0.793766 | |
| C | 7.057359 | 3.353438 | 0.793766 | |
| C | 8.253873 | 4.087584 | 0.793766 | |
| C | 8.288739 | 5.490777 | 0.793766 | |
| C | 7.054536 | 6.159733 | 0.793766 | |
| B | 7.057135 | 1.856436 | 0.793766 | |
| O | 5.849398 | 1.162547 | 0.793766 | |
| B | 9.585465 | 6.238942 | 0.793766 | |
| O | 3.319859 | 5.539076 | 0.793766 | |
| B | 4.525276 | 6.236943 | 0.793766 | |
| O | 0.7937660 | 1.160613 | 0.793766 | |
| H | 9.210704 | 3.538126 | 0.793766 | |
| H | 4.903924 | 3.533164 | 0.793766 | |
| H | 3.318080 | 0.7937660 | 0.793766 | |
| Transformation matrix | | | | |
| **a** | 7.469940 | 0.000000 | 0.000000 | |
| **b** | 3.734970 | 6.469160 | 0.000000 | |
| **c** | 0.000000 | 0.000000 | 3.488410 | AA |
| **c** | 1.197280 | 0.691250 | 3.420000 | Inclined zigzag |

## Layer II AB stacking

| | | | |
|---|---|---|---|
| C | 9.556662 | 7.644640 | 4.062411 |
| C | 9.594285 | 6.241525 | 4.062411 |
| C | 10.79233 | 5.509824 | 4.062411 |
| C | 11.98884 | 6.243970 | 4.062411 |
| C | 12.02371 | 7.647163 | 4.062411 |
| C | 10.78951 | 8.316119 | 4.062411 |

| | | | |
|---|---|---|---|
| B | 10.79210 | 4.012822 | 4.062411 |
| O | 9.584368 | 3.318933 | 4.062411 |
| B | 13.32044 | 8.395329 | 4.062411 |
| O | 7.054829 | 7.695462 | 4.062411 |
| B | 8.260246 | 8.393329 | 4.062411 |
| O | 4.528736 | 3.316999 | 4.062411 |
| H | 12.94567 | 5.694512 | 4.062411 |
| H | 8.638894 | 5.689550 | 4.062411 |
| H | 7.053050 | 2.950152 | 4.062411 |

**c** 0.00000 0.00000 6.53729 AB

### Layer II serrated zigzag

| | | | |
|---|---|---|---|
| C | 7.018972 | 6.179504 | 4.206926 |
| C | 7.056595 | 4.776389 | 4.206926 |
| C | 8.254640 | 4.044688 | 4.206926 |
| C | 9.451153 | 4.778834 | 4.206926 |
| C | 9.486019 | 6.182027 | 4.206926 |
| C | 4.516846 | 0.381823 | 4.206926 |
| B | 8.254416 | 2.547686 | 4.206926 |
| O | 7.046678 | 1.853797 | 4.206926 |
| B | 7.047780 | 0.461032 | 4.206926 |
| O | 4.517139 | 6.230326 | 4.206926 |
| B | 1.987586 | 0.459033 | 4.206926 |
| O | 1.991046 | 1.851863 | 4.206926 |
| H | 2.938040 | 4.229376 | 4.206926 |
| H | 6.101204 | 4.224414 | 4.206926 |
| H | 4.515360 | 1.485016 | 4.206926 |

**c** 0.00000 0.00000 6.82632 serrated zigzag

## PPy-COF

### Layer I AA, AB, inclined zigzag, and serrated zigzag

| | | | | | | | | | | | |
|---|---|---|---|---|---|---|---|---|---|---|---|
| C | 11.35865 | 16.45284 | 0.793925 | C | 21.18579 | 2.159241 | 0.7941000 | C | 22.44165 | 5.668580 | 0.7939750 |
| C | 11.38651 | 15.04819 | 0.793975 | C | 21.21311 | 3.560100 | 0.7941000 | H | 22.13766 | 8.368893 | 0.7938500 |
| C | 12.58565 | 14.32387 | 0.794000 | B | 31.15145 | 19.26523 | 0.7940250 | H | 17.82290 | 8.369067 | 0.7943000 |
| C | 13.82106 | 15.03222 | 0.793975 | O | 32.36685 | 18.58465 | 0.7939750 | C | 17.52082 | 5.666763 | 0.7942250 |
| C | 13.81621 | 16.45653 | 0.793925 | B | 10.05600 | 17.19214 | 0.7939250 | H | 22.13976 | 1.606842 | 0.7941250 |
| C | 12.58860 | 17.13289 | 0.793875 | O | 31.17973 | 16.47913 | 0.7939000 | C | 22.44209 | 4.307889 | 0.7940500 |
| C | 15.05371 | 14.31962 | 0.794025 | B | 29.96877 | 17.16670 | 0.7939500 | C | 17.52160 | 4.305840 | 0.7941750 |
| C | 15.05690 | 12.89544 | 0.793975 | O | 29.95041 | 18.55973 | 0.7940000 | H | 17.82505 | 1.604832 | 0.7940250 |
| C | 16.28348 | 12.21823 | 0.793975 | C | 28.67703 | 16.40861 | 0.7939500 | C | 15.07907 | 17.14633 | 0.7939750 |
| C | 17.51485 | 12.89589 | 0.794075 | C | 27.44604 | 17.08635 | 0.7940750 | H | 12.58726 | 18.23522 | 0.7938000 |
| C | 17.48922 | 14.30122 | 0.794150 | C | 26.21960 | 16.40935 | 0.7940750 | H | 10.43102 | 14.49862 | 0.7940500 |
| C | 16.29005 | 15.02674 | 0.794100 | C | 26.21594 | 14.98518 | 0.7940000 | C | 12.61698 | 12.88572 | 0.7939750 |
| B | 18.80745 | 12.13886 | 0.794100 | C | 27.45222 | 14.27783 | 0.7939250 | H | 18.44503 | 14.85062 | 0.7942500 |
| O | 20.01934 | 12.82580 | 0.794100 | C | 28.65134 | 15.00327 | 0.7938750 | C | 16.25751 | 16.46517 | 0.7940750 |
| B | 21.21724 | 12.11402 | 0.794075 | C | 24.98299 | 14.27328 | 0.7940250 | C | 13.79484 | 12.20472 | 0.7939500 |
| O | 21.20629 | 10.72118 | 0.794075 | C | 23.74797 | 14.98228 | 0.7940250 | H | 16.28412 | 11.11581 | 0.7939250 |
| B | 19.99100 | 10.03987 | 0.794075 | C | 22.54846 | 14.25844 | 0.7940250 | H | 28.37327 | 12.28811 | 0.7938500 |
| O | 18.79020 | 10.74570 | 0.794075 | C | 22.52000 | 12.85340 | 0.7940750 | H | 26.24156 | 11.05674 | 0.7940500 |
| C | 19.98093 | 8.542014 | 0.794075 | C | 23.75001 | 12.17288 | 0.7941250 | H | 24.95853 | 18.20265 | 0.7941500 |
| C | 18.77697 | 7.816687 | 0.794200 | C | 24.97778 | 12.84901 | 0.7940500 | H | 22.82538 | 16.97153 | 0.7940500 |
| C | 18.74956 | 6.415403 | 0.794175 | C | 27.41899 | 12.83925 | 0.7939250 | H | 23.39572 | 6.220187 | 0.7939000 |
| C | 19.98089 | 5.699238 | 0.794100 | H | 29.60688 | 14.45348 | 0.7937500 | H | 23.39716 | 3.757810 | 0.7940500 |
| C | 21.21208 | 6.415209 | 0.794000 | H | 27.44672 | 18.18865 | 0.7941750 | H | 16.56561 | 6.216244 | 0.7943000 |

| | | | | | | | | | | | |
|---|---|---|---|---|---|---|---|---|---|---|---|
| C | 21.18410 | 7.815895 | 0.793975 | C | 24.95805 | 17.10069 | 0.7941000 | H | 16.56737 | 3.754214 | 0.7941750 |
| C | 19.98162 | 4.275395 | 0.794100 | H | 23.74866 | 11.07031 | 0.7941750 | H | 17.21184 | 17.01626 | 0.7941250 |
| C | 18.75092 | 3.558709 | 0.794100 | C | 26.24024 | 12.15864 | 0.7940250 | H | 15.08064 | 18.24831 | 0.7939250 |
| C | 18.77876 | 2.157579 | 0.794050 | C | 23.77973 | 16.42044 | 0.7940500 | H | 13.79363 | 11.10274 | 0.7939250 |
| C | 19.98266 | 1.432851 | 0.794075 | H | 21.59317 | 14.80879 | 0.7939750 | H | 11.66185 | 12.33587 | 0.7940000 |

Transformation Matrix

| | | | | |
|---|---|---|---|---|
| **a** | 22.320600 | 0.000000 | 0.000000 | |
| **b** | 11.160300 | 19.330200 | 0.000000 | |
| **c** | 0.000000 | 0.000000 | 3.488000 | AA |
| **c** | 1.21997 | 0.70435 | 3.411452 | inclined zigzag |

## Layer II AB stacking

| | | | | | | | | | | | |
|---|---|---|---|---|---|---|---|---|---|---|---|
| C | 22.51895 | 22.89624 | 3.762205 | C | 32.34609 | 8.602643 | 3.762380 | C | 33.60195 | 12.11198 | 3.762255 |
| C | 22.54681 | 21.49159 | 3.762255 | C | 32.37341 | 10.00350 | 3.762380 | H | 33.29796 | 14.81229 | 3.762130 |
| C | 23.74595 | 20.76727 | 3.762280 | B | 42.31175 | 25.70863 | 3.762305 | H | 28.98320 | 14.81247 | 3.762580 |
| C | 24.98136 | 21.47562 | 3.762255 | O | 43.52715 | 25.02805 | 3.762255 | C | 28.68112 | 12.11016 | 3.762505 |
| C | 24.97651 | 22.89993 | 3.762205 | B | 21.21630 | 23.63554 | 3.762205 | H | 33.30006 | 8.050244 | 3.762405 |
| C | 23.74890 | 23.57629 | 3.762155 | O | 42.34003 | 22.92253 | 3.762180 | C | 33.60239 | 10.75129 | 3.762330 |
| C | 26.21401 | 20.76302 | 3.762305 | B | 41.12907 | 23.61010 | 3.762230 | C | 28.68190 | 10.74924 | 3.762455 |
| C | 26.21720 | 19.33884 | 3.762255 | O | 41.11071 | 25.00313 | 3.762280 | H | 28.98535 | 8.048234 | 3.762305 |
| C | 27.44378 | 18.66163 | 3.762255 | C | 39.83733 | 22.85201 | 3.762230 | C | 26.23937 | 23.58973 | 3.762255 |
| C | 28.67515 | 19.33929 | 3.762355 | C | 38.60634 | 23.52975 | 3.762355 | H | 23.74756 | 24.67862 | 3.762080 |
| C | 28.64952 | 20.74462 | 3.762430 | C | 37.37990 | 22.85275 | 3.762355 | H | 21.59132 | 20.94202 | 3.762330 |
| C | 27.45035 | 21.47014 | 3.762380 | C | 37.37624 | 21.42858 | 3.762280 | C | 23.77728 | 19.32912 | 3.762255 |
| B | 29.96775 | 18.58226 | 3.762380 | C | 38.61252 | 20.72123 | 3.762205 | H | 29.60533 | 21.29402 | 3.762530 |
| O | 31.17964 | 19.26920 | 3.762380 | C | 39.81164 | 21.44667 | 3.762155 | C | 27.41781 | 22.90857 | 3.762355 |
| B | 32.37754 | 18.55742 | 3.762355 | C | 36.14329 | 20.71668 | 3.762305 | C | 24.95514 | 18.64812 | 3.762230 |
| O | 32.36659 | 17.16458 | 3.762355 | C | 34.90827 | 21.42568 | 3.762305 | H | 27.44442 | 17.55921 | 3.762205 |
| B | 31.15130 | 16.48327 | 3.762355 | C | 33.70876 | 20.70184 | 3.762305 | H | 39.53357 | 18.73151 | 3.762130 |
| O | 29.95050 | 17.18910 | 3.762355 | C | 33.68030 | 19.29680 | 3.762355 | H | 37.40186 | 17.50014 | 3.762330 |
| C | 31.14123 | 14.98542 | 3.762355 | C | 34.91031 | 18.61628 | 3.762405 | H | 36.11883 | 24.64605 | 3.762430 |
| C | 29.93727 | 14.26009 | 3.762480 | C | 36.13808 | 19.29241 | 3.762330 | H | 33.98568 | 23.41493 | 3.762330 |
| C | 29.90986 | 12.85880 | 3.762455 | C | 38.57929 | 19.28265 | 3.762205 | H | 34.55602 | 12.66359 | 3.762180 |
| C | 31.14119 | 12.14264 | 3.762380 | H | 40.76718 | 20.89688 | 3.762030 | H | 34.55746 | 10.20121 | 3.762330 |
| C | 32.37238 | 12.85861 | 3.762280 | H | 38.60702 | 24.63205 | 3.762455 | H | 27.72591 | 12.65965 | 3.762580 |
| C | 32.34440 | 14.25930 | 3.762255 | C | 36.11835 | 23.54409 | 3.762380 | H | 27.72767 | 10.19762 | 3.762455 |
| C | 31.14192 | 10.71880 | 3.762380 | H | 34.90896 | 17.51371 | 3.762455 | H | 28.37214 | 23.45966 | 3.762405 |
| C | 29.91122 | 10.00211 | 3.762380 | C | 37.40054 | 18.60204 | 3.762305 | H | 26.24094 | 24.69171 | 3.762205 |
| C | 29.93906 | 8.600981 | 3.762330 | C | 34.94003 | 22.86384 | 3.762330 | H | 24.95393 | 17.54614 | 3.762205 |
| C | 31.14296 | 7.876253 | 3.762355 | H | 32.75347 | 21.25219 | 3.762255 | H | 22.82215 | 18.77927 | 3.762280 |

| | | | | |
|---|---|---|---|---|
| **c** | 0.00000 | 0.00000 | 5.93656 | AB |

## Layer II serrated zigzag

| | | | | | | | | | | | |
|---|---|---|---|---|---|---|---|---|---|---|---|
| C | 12.57862 | 17.157190 | 4.203665 | C | 22.40576 | 2.863591 | 4.203840 | C | 23.66162 | 6.37293 | 4.203715 |
| C | 12.60648 | 15.752540 | 4.203715 | C | 22.43308 | 4.264450 | 4.203840 | H | 23.35763 | 9.07324 | 4.203590 |
| C | 13.80562 | 15.028220 | 4.203740 | B | 21.21112 | 0.639380 | 4.203765 | H | 19.04287 | 9.07341 | 4.204040 |
| C | 15.04103 | 15.736570 | 4.203715 | O | 11.26622 | 19.289000 | 4.203715 | C | 18.74079 | 6.37111 | 4.203965 |
| C | 15.03618 | 17.160880 | 4.203665 | B | 11.27597 | 17.896490 | 4.203665 | H | 23.35973 | 2.31119 | 4.203865 |
| C | 13.80857 | 17.837240 | 4.203615 | O | 10.07910 | 17.183480 | 4.203640 | C | 23.66206 | 5.01223 | 4.203790 |
| C | 16.27368 | 15.023970 | 4.203765 | B | 31.18874 | 17.871050 | 4.203690 | C | 18.74157 | 5.01019 | 4.203915 |
| C | 16.27687 | 13.599790 | 4.203715 | O | 31.17038 | 19.264080 | 4.203740 | H | 19.04502 | 2.30918 | 4.203765 |
| C | 17.50345 | 12.922580 | 4.203715 | C | 29.89700 | 17.112960 | 4.203690 | C | 16.29904 | 17.85068 | 4.203715 |
| C | 18.73482 | 13.600240 | 4.203815 | C | 28.66601 | 17.790700 | 4.203815 | H | 13.80723 | 18.93957 | 4.203540 |
| C | 18.70919 | 15.005570 | 4.203890 | C | 27.43957 | 17.113700 | 4.203815 | H | 11.65099 | 15.20297 | 4.203790 |
| C | 17.51002 | 15.731090 | 4.203840 | C | 27.43591 | 15.689530 | 4.203740 | C | 13.83695 | 13.59007 | 4.203715 |
| B | 20.02742 | 12.843210 | 4.203840 | C | 28.67219 | 14.982180 | 4.203665 | H | 19.66500 | 15.55497 | 4.203990 |
| O | 21.23931 | 13.530150 | 4.203840 | C | 29.87131 | 15.707620 | 4.203615 | C | 17.47748 | 17.16952 | 4.203815 |
| B | 22.43721 | 12.818370 | 4.203815 | C | 26.20296 | 14.977630 | 4.203765 | C | 15.01481 | 12.90907 | 4.203690 |
| O | 22.42626 | 11.425530 | 4.203815 | C | 24.96794 | 15.686630 | 4.203765 | H | 17.50409 | 11.82016 | 4.203665 |

| | | | | | | | | | | | |
|---|---|---|---|---|---|---|---|---|---|---|---|
| B | 21.21097 | 10.744220 | 4.203815 | C | 23.76843 | 14.962790 | 4.203765 | H | 29.59324 | 12.99246 | 4.203590 |
| O | 20.01017 | 11.450050 | 4.203815 | C | 23.73997 | 13.557750 | 4.203815 | H | 27.46153 | 11.76109 | 4.203790 |
| C | 21.20090 | 9.246365 | 4.203815 | C | 24.96998 | 12.877230 | 4.203865 | H | 26.17850 | 18.90700 | 4.203890 |
| C | 19.99694 | 8.521037 | 4.203940 | C | 26.19775 | 13.553360 | 4.203790 | H | 24.04535 | 17.67588 | 4.203790 |
| C | 19.96953 | 7.119753 | 4.203915 | C | 28.63896 | 13.543600 | 4.203665 | H | 24.61569 | 6.92453 | 4.203640 |
| C | 21.20086 | 6.403588 | 4.203840 | H | 30.82685 | 15.157830 | 4.203490 | H | 24.61713 | 4.46216 | 4.203790 |
| C | 22.43205 | 7.119559 | 4.203740 | H | 28.66669 | 18.893000 | 4.203915 | H | 17.78558 | 6.92059 | 4.204040 |
| C | 22.40407 | 8.520245 | 4.203715 | C | 26.17802 | 17.805040 | 4.203840 | H | 17.78734 | 4.45856 | 4.203915 |
| C | 21.20159 | 4.979745 | 4.203840 | H | 24.96863 | 11.774660 | 4.203915 | H | 18.43181 | 17.72061 | 4.203865 |
| C | 19.97089 | 4.263059 | 4.203840 | C | 27.46021 | 12.862990 | 4.203765 | H | 16.30061 | 18.95266 | 4.203665 |
| C | 19.99873 | 2.861929 | 4.203790 | C | 24.99970 | 17.124790 | 4.203790 | H | 15.01360 | 11.80709 | 4.203665 |
| C | 21.20263 | 2.137201 | 4.203815 | H | 22.81314 | 15.513140 | 4.203715 | H | 12.88182 | 13.04022 | 4.203740 |

**c** 0.00000 0.00000 6.81948 serrated zigzag

# COF-5

## Layer I AA, AB, inclined zigzag & armchair, and serrated zigzag & armchair

| | | | | | | | | | | | |
|---|---|---|---|---|---|---|---|---|---|---|---|
| C | 39.38465 | 22.38862 | 0.8077360 | O | 20.47563 | 18.71826 | 0.8808860 | B | 35.64685 | 23.16284 | 0.8173060 |
| C | 39.41922 | 20.98273 | 0.8040060 | C | 15.45640 | 10.01228 | 0.8776360 | O | 34.40619 | 22.51739 | 0.8181660 |
| C | 38.18019 | 20.31742 | 0.7978060 | C | 16.65255 | 9.272400 | 0.8877860 | C | 24.35300 | 22.27500 | 0.8515860 |
| C | 36.98136 | 21.01507 | 0.7988360 | C | 16.64961 | 7.885280 | 0.8870660 | C | 24.30941 | 20.86914 | 0.8521860 |
| C | 36.94609 | 22.42116 | 0.8066160 | C | 15.45058 | 7.150020 | 0.8764060 | C | 23.10522 | 20.17951 | 0.8579360 |
| C | 38.18526 | 23.08650 | 0.8090860 | C | 14.25431 | 7.889580 | 0.8657860 | C | 21.87033 | 20.85244 | 0.8632060 |
| B | 40.70932 | 20.22534 | 0.8125860 | C | 14.25719 | 9.277330 | 0.8661560 | C | 21.91408 | 22.25825 | 0.8607860 |
| O | 41.96477 | 20.84153 | 0.8205360 | B | 15.45885 | 5.653904 | 0.8753560 | C | 23.11767 | 22.94780 | 0.8555060 |
| C | 12.75951 | 19.73973 | 0.8336660 | O | 16.63828 | 4.902127 | 0.8771360 | H | 36.03313 | 20.45280 | 0.7953560 |
| C | 14.13734 | 19.76794 | 0.8462360 | C | 16.18376 | 3.533013 | 0.8730860 | H | 38.16029 | 19.21536 | 0.7937660 |
| C | 14.82425 | 18.53141 | 0.8582860 | C | 16.92734 | 2.372618 | 0.8702360 | H | 40.33294 | 22.95052 | 0.8117860 |
| C | 14.10000 | 17.30940 | 0.8571560 | C | 16.23177 | 1.141180 | 0.8641860 | H | 38.20604 | 24.18863 | 0.8138360 |
| C | 12.68582 | 17.31851 | 0.8439560 | C | 14.81144 | 1.121423 | 0.8625060 | H | 13.29518 | 9.815740 | 0.8570360 |
| C | 12.04926 | 18.54070 | 0.8325360 | C | 14.08058 | 2.332607 | 0.8653360 | H | 13.29003 | 7.355373 | 0.8564160 |
| C | 14.81330 | 16.03980 | 0.8681360 | C | 14.79044 | 3.514143 | 0.8701060 | H | 17.61128 | 7.346499 | 0.8952160 |
| C | 16.23385 | 16.02310 | 0.8794360 | C | 29.16997 | 25.94961 | 0.8570560 | H | 17.61656 | 9.807080 | 0.8963160 |
| C | 16.97668 | 17.27594 | 0.8794860 | C | 29.89655 | 24.72903 | 0.8511660 | H | 12.98228 | 14.85730 | 0.8558360 |
| C | 16.28037 | 18.51419 | 0.8695360 | C | 31.35303 | 24.74986 | 0.8490860 | H | 18.91793 | 16.28417 | 0.8931860 |
| C | 16.93177 | 14.79286 | 0.8879560 | C | 32.04632 | 25.98983 | 0.8564860 | H | 16.43995 | 20.68804 | 0.8596360 |
| C | 16.19076 | 13.63059 | 0.8841660 | C | 29.21184 | 23.49104 | 0.8461360 | H | 18.03483 | 14.79542 | 0.8960660 |
| C | 14.79744 | 13.64734 | 0.8730660 | C | 27.83368 | 23.51634 | 0.8477060 | H | 14.71344 | 20.70860 | 0.8467560 |
| C | 14.08506 | 14.82721 | 0.8652860 | C | 27.12170 | 24.71423 | 0.8533060 | H | 12.13732 | 16.36148 | 0.8427860 |
| C | 16.99491 | 19.73474 | 0.8674060 | C | 27.75580 | 25.93760 | 0.8577160 | H | 23.10512 | 24.05012 | 0.8547660 |
| C | 18.37174 | 19.67534 | 0.8727360 | C | 33.46062 | 26.01705 | 0.8532360 | H | 12.13660 | 0.7937660 | 0.8618060 |
| C | 19.05533 | 18.46110 | 0.8813860 | C | 34.12878 | 24.81158 | 0.8402860 | H | 31.52002 | 22.57643 | 0.8325560 |
| C | 18.39107 | 17.25331 | 0.8862060 | C | 33.44818 | 23.59547 | 0.8326560 | H | 18.91479 | 0.8885610 | 0.8596660 |
| O | 14.30785 | 12.28935 | 0.8697560 | C | 32.07148 | 23.53168 | 0.8379660 | H | 29.78991 | 22.55151 | 0.8412660 |
| B | 15.46802 | 11.50839 | 0.8787360 | O | 25.70864 | 24.42541 | 0.8541060 | H | 18.03041 | 2.372697 | 0.8713860 |
| O | 16.64678 | 12.26132 | 0.8884360 | B | 25.64560 | 23.02835 | 0.8493860 | H | 12.97787 | 2.299852 | 0.8627160 |
| O | 40.77311 | 18.82814 | 0.8185360 | O | 26.90171 | 22.41331 | 0.8447660 | H | 20.96961 | 22.82688 | 0.8639060 |
| O | 19.32740 | 20.75623 | 0.8671860 | O | 14.29926 | 4.871904 | 0.8716060 | H | 23.11811 | 19.07719 | 0.8582860 |
| B | 20.56945 | 20.11355 | 0.8707860 | O | 35.55002 | 24.55802 | 0.8308860 | H | 25.25342 | 20.29993 | 0.8487160 |

Transformation Matrix

| | | | | |
|---|---|---|---|---|
| **a** | 30.137400 | 0.000000 | 0.000000 | |
| **b** | 15.068700 | 26.099740 | 0.000000 | |
| **c** | 0.000000 | 0.000000 | 3.487498 | AA |
| **c** | 1.197700 | 0.691492 | 3.405000 | inclined zigzag |
| **c** | 1.387445 | 0.000000 | 3.410000 | inclined armchair |

## Layer II AB stacking

| | | | | | | | | | | | |
|---|---|---|---|---|---|---|---|---|---|---|---|
| C | 54.45335 | 31.08854 | 4.065051 | O | 35.54433 | 27.41818 | 4.138201 | B | 50.71555 | 31.86276 | 4.074621 |
| C | 54.48792 | 29.68265 | 4.061321 | C | 30.52510 | 18.71220 | 4.134951 | O | 49.47488 | 31.21731 | 4.075481 |
| C | 53.24889 | 29.01734 | 4.055121 | C | 31.72125 | 17.97232 | 4.145101 | C | 39.42170 | 30.97492 | 4.108901 |
| C | 52.05006 | 29.71499 | 4.056151 | C | 31.71831 | 16.58520 | 4.144381 | C | 39.37811 | 29.56906 | 4.109501 |
| C | 52.01479 | 31.12108 | 4.063931 | C | 30.51928 | 15.84994 | 4.133721 | C | 38.17392 | 28.87943 | 4.115251 |
| C | 53.25396 | 31.78642 | 4.066401 | C | 29.32301 | 16.58950 | 4.123101 | C | 36.93903 | 29.55236 | 4.120521 |
| B | 55.77802 | 28.92526 | 4.069901 | C | 29.32589 | 17.97725 | 4.123471 | C | 36.98278 | 30.95817 | 4.118101 |
| O | 57.03347 | 29.54145 | 4.077851 | B | 30.52755 | 14.35382 | 4.132671 | C | 38.18637 | 31.64772 | 4.112821 |
| C | 27.82821 | 28.43965 | 4.090981 | O | 31.70698 | 13.60205 | 4.134451 | H | 51.10183 | 29.15272 | 4.052671 |
| C | 29.20604 | 28.46786 | 4.103551 | C | 31.25246 | 12.23293 | 4.130401 | H | 53.22898 | 27.91528 | 4.051081 |
| C | 29.89295 | 27.23133 | 4.115601 | C | 31.99604 | 11.07254 | 4.127551 | H | 55.40164 | 31.65044 | 4.069101 |
| C | 29.16870 | 26.00932 | 4.114471 | C | 31.30047 | 9.841098 | 4.121501 | H | 53.27474 | 32.88855 | 4.071151 |
| C | 27.75452 | 26.01843 | 4.101271 | C | 29.88014 | 9.821341 | 4.119821 | H | 28.36388 | 18.51566 | 4.114351 |
| C | 27.11796 | 27.24062 | 4.089851 | C | 29.14928 | 11.03253 | 4.122651 | H | 28.35873 | 16.05529 | 4.113731 |
| C | 29.88200 | 24.73972 | 4.125451 | C | 29.85914 | 12.21406 | 4.127421 | H | 32.67998 | 16.04642 | 4.152531 |
| C | 31.30255 | 24.72302 | 4.136751 | C | 44.23867 | 34.64953 | 4.114371 | H | 32.68526 | 18.50700 | 4.153631 |
| C | 32.04538 | 25.97586 | 4.136801 | C | 44.96525 | 33.42895 | 4.108481 | H | 28.05098 | 23.55722 | 4.113151 |
| C | 31.34907 | 27.21411 | 4.126851 | C | 46.42173 | 33.44978 | 4.106401 | H | 33.98663 | 24.98409 | 4.150501 |
| C | 32.00047 | 23.49278 | 4.145271 | C | 47.11502 | 34.68975 | 4.113801 | H | 31.50865 | 29.38796 | 4.116951 |
| C | 31.25946 | 22.33051 | 4.141481 | C | 44.28054 | 32.19096 | 4.103451 | H | 33.10353 | 23.49534 | 4.153381 |
| C | 29.86614 | 22.34726 | 4.130381 | C | 42.90238 | 32.21626 | 4.105021 | H | 29.78214 | 29.40852 | 4.104071 |
| C | 29.15376 | 23.52713 | 4.122601 | C | 42.19040 | 33.41415 | 4.110621 | H | 27.20602 | 25.06140 | 4.100101 |
| C | 32.06361 | 28.43466 | 4.124721 | C | 42.82450 | 34.63752 | 4.115031 | H | 38.17382 | 32.75004 | 4.112081 |
| C | 33.44044 | 28.37526 | 4.130051 | C | 48.52932 | 34.71697 | 4.110551 | H | 27.20530 | 9.493684 | 4.119121 |
| C | 34.12403 | 27.16102 | 4.138701 | C | 49.19748 | 33.51150 | 4.097601 | H | 46.58872 | 31.27635 | 4.089871 |
| C | 33.45977 | 25.95323 | 4.143521 | C | 48.51688 | 32.29539 | 4.089971 | H | 33.98349 | 9.588479 | 4.116981 |
| O | 29.37655 | 20.98927 | 4.127071 | C | 47.14018 | 32.23160 | 4.095281 | H | 44.85861 | 31.25143 | 4.098581 |
| B | 30.53672 | 20.20831 | 4.136051 | O | 40.77734 | 33.12533 | 4.111421 | H | 33.09911 | 11.07261 | 4.128701 |
| O | 31.71548 | 20.96124 | 4.145751 | B | 40.71430 | 31.72827 | 4.106701 | H | 28.04657 | 10.99977 | 4.120031 |
| O | 55.84181 | 27.52806 | 4.075851 | O | 41.97041 | 31.11323 | 4.102081 | H | 36.03831 | 31.52680 | 4.121221 |
| O | 34.39610 | 29.45615 | 4.124501 | O | 29.36796 | 13.57182 | 4.128921 | H | 38.18681 | 27.77711 | 4.115601 |
| B | 35.63815 | 28.81347 | 4.128101 | O | 50.61872 | 33.25794 | 4.088201 | H | 40.32212 | 28.99985 | 4.106031 |
| **c** | 0.00000 | 0.00000 | 6.51463 | AB | | | | | | | |

## Layer II serrated zigzag

| | | | | | | | | | | | |
|---|---|---|---|---|---|---|---|---|---|---|---|
| C | 40.58235 | 23.08011 | 4.22612 | O | 21.67333 | 19.409750 | 4.299271 | B | 36.84455 | 23.854330 | 4.23569100 |
| C | 40.61692 | 21.67422 | 4.22239 | C | 16.65410 | 10.703770 | 4.296021 | O | 35.60389 | 23.208880 | 4.23655100 |
| C | 39.37789 | 21.00891 | 4.21619 | C | 17.85025 | 9.963892 | 4.306171 | C | 25.55070 | 22.966490 | 4.26997100 |
| C | 38.17906 | 21.70656 | 4.21722 | C | 17.84731 | 8.576772 | 4.305451 | C | 25.50711 | 21.560630 | 4.27057100 |
| C | 38.14380 | 23.11265 | 4.22500 | C | 16.64828 | 7.841512 | 4.294791 | C | 24.30292 | 20.871000 | 4.27632100 |
| C | 39.38296 | 23.77799 | 4.22747 | C | 15.45201 | 8.581072 | 4.284171 | C | 23.06803 | 21.543930 | 4.28159100 |
| B | 41.90702 | 20.91683 | 4.23097 | C | 15.45489 | 9.968822 | 4.284541 | C | 23.11178 | 22.949740 | 4.27917100 |
| O | 13.02507 | 21.53302 | 4.23892 | B | 16.65655 | 6.345396 | 4.293741 | C | 24.31537 | 23.639290 | 4.27389100 |
| C | 13.95721 | 20.43122 | 4.25205 | O | 17.83598 | 5.593619 | 4.295521 | H | 37.23083 | 21.144290 | 4.21374100 |
| C | 15.33504 | 20.45943 | 4.26462 | C | 17.38146 | 4.224505 | 4.291471 | H | 39.35799 | 19.906850 | 4.21215100 |
| C | 16.02195 | 19.22290 | 4.27667 | C | 18.12504 | 3.064110 | 4.288621 | H | 41.53064 | 23.642010 | 4.23017100 |
| C | 15.29770 | 18.00089 | 4.27554 | C | 17.42947 | 1.832672 | 4.282571 | H | 39.40374 | 24.880120 | 4.23222100 |
| C | 13.88352 | 18.01000 | 4.26234 | C | 16.00914 | 1.812915 | 4.280891 | H | 14.49288 | 10.507230 | 4.27542100 |
| C | 13.24696 | 19.23219 | 4.25092 | C | 15.27828 | 3.024099 | 4.283721 | H | 14.48773 | 8.046865 | 4.27480100 |
| C | 16.01100 | 16.73129 | 4.28652 | C | 15.98814 | 4.205635 | 4.288491 | H | 18.80898 | 8.037991 | 4.31360100 |
| C | 17.43155 | 16.71459 | 4.29782 | C | 15.29897 | 0.541360 | 4.275441 | H | 18.81426 | 10.498570 | 4.31470100 |
| C | 18.17438 | 17.96743 | 4.29787 | C | 31.09425 | 25.420520 | 4.269551 | H | 14.17998 | 15.548790 | 4.27422100 |
| C | 17.47807 | 19.20568 | 4.28792 | C | 32.55073 | 25.441350 | 4.267471 | H | 20.11563 | 16.975660 | 4.31157100 |
| C | 18.12947 | 15.48435 | 4.30634 | C | 18.17532 | 0.581580 | 4.274871 | H | 17.63765 | 21.379530 | 4.27802100 |
| C | 17.38846 | 14.32208 | 4.30255 | C | 30.40954 | 24.182530 | 4.264521 | H | 19.23253 | 15.486910 | 4.31445100 |
| C | 15.99514 | 14.33883 | 4.29145 | C | 29.03138 | 24.207830 | 4.266091 | H | 15.91114 | 21.400090 | 4.26514100 |
| C | 15.28276 | 15.51870 | 4.28367 | C | 28.31940 | 25.405720 | 4.271691 | H | 13.33502 | 17.052970 | 4.26117100 |
| C | 18.19261 | 20.42623 | 4.28579 | C | 13.88480 | 0.529350 | 4.276101 | H | 24.30282 | 24.741610 | 4.27315100 |
| C | 19.56944 | 20.36683 | 4.29112 | C | 19.58962 | 0.608800 | 4.271621 | H | 13.33430 | 1.485258 | 4.28019100 |
| C | 20.25303 | 19.15259 | 4.29977 | C | 35.32648 | 25.503070 | 4.258671 | H | 32.71772 | 23.267920 | 4.25094100 |

| | | | | | | | | | | | |
|---|---|---|---|---|---|---|---|---|---|---|---|
| C | 19.58877 | 17.94480 | 4.30459 | C | 34.64588 | 24.286960 | 4.251041 | H | 20.11249 | 1.580053 | 4.27805100 |
| O | 15.50555 | 12.98084 | 4.28814 | C | 33.26918 | 24.223170 | 4.256351 | H | 30.98761 | 23.243000 | 4.25965100 |
| B | 16.66572 | 12.19988 | 4.29712 | O | 26.90634 | 25.116900 | 4.272491 | H | 19.22811 | 3.064189 | 4.28977100 |
| O | 17.84448 | 12.95281 | 4.30682 | B | 26.84330 | 23.719840 | 4.267771 | H | 14.17557 | 2.991344 | 4.28110100 |
| O | 11.83341 | 19.51963 | 4.23692 | O | 28.09941 | 23.104800 | 4.263151 | H | 22.16731 | 23.518370 | 4.28229100 |
| O | 20.52510 | 21.44772 | 4.28557 | O | 15.49696 | 5.563396 | 4.289991 | H | 24.31581 | 19.768680 | 4.27667100 |
| B | 21.76715 | 20.80504 | 4.28917 | O | 36.74772 | 25.249510 | 4.249271 | H | 26.45112 | 20.991420 | 4.26710100 |

**c** 0.00000 0.00000 6.83677 serrated zigzag

## Layer II serrated armchair

| | | | | | | | | | | | |
|---|---|---|---|---|---|---|---|---|---|---|---|
| C | 40.77209 | 22.38862 | 4.22813 | O | 21.86308 | 18.718260 | 4.301286 | B | 37.03430 | 23.162840 | 4.23770 |
| C | 40.80666 | 20.98273 | 4.22440 | C | 16.84385 | 10.012280 | 4.298036 | O | 35.79363 | 22.517390 | 4.23856 |
| C | 39.56763 | 20.31742 | 4.21820 | C | 18.04000 | 9.272400 | 4.308186 | C | 25.74045 | 22.275000 | 4.27198 |
| C | 38.36880 | 21.01507 | 4.21923 | C | 18.03706 | 7.885280 | 4.307466 | C | 25.69686 | 20.869140 | 4.27258 |
| C | 38.33354 | 22.42116 | 4.22701 | C | 16.83802 | 7.150020 | 4.296806 | C | 24.49267 | 20.179510 | 4.27833 |
| C | 39.57270 | 23.08650 | 4.22948 | C | 15.64175 | 7.889580 | 4.286186 | C | 23.25778 | 20.852440 | 4.28360 |
| B | 11.95936 | 20.22534 | 4.23298 | C | 15.64463 | 9.277330 | 4.286556 | C | 23.30153 | 22.258250 | 4.28118 |
| O | 13.21481 | 20.84153 | 4.24093 | B | 16.84629 | 5.653904 | 4.295756 | C | 24.50512 | 22.947800 | 4.27590 |
| C | 14.14696 | 19.73973 | 4.25406 | O | 18.02573 | 4.902127 | 4.297536 | H | 37.42057 | 20.452800 | 4.21575 |
| C | 15.52478 | 19.76794 | 4.26663 | C | 17.57121 | 3.533013 | 4.293486 | H | 39.54773 | 19.215360 | 4.21416 |
| C | 16.21169 | 18.53141 | 4.27868 | C | 18.31479 | 2.372618 | 4.290636 | H | 41.72038 | 22.950520 | 4.23218 |
| C | 15.48745 | 17.30940 | 4.27755 | C | 17.61922 | 1.141180 | 4.284586 | H | 39.59349 | 24.188630 | 4.23423 |
| C | 14.07327 | 17.31851 | 4.26435 | C | 16.19888 | 1.121423 | 4.282906 | H | 14.68262 | 9.815740 | 4.27743 |
| C | 13.43670 | 18.54070 | 4.25293 | C | 15.46803 | 2.332607 | 4.285736 | H | 14.67747 | 7.355373 | 4.27681 |
| C | 16.20074 | 16.03980 | 4.28853 | C | 16.17789 | 3.514143 | 4.290506 | H | 18.99873 | 7.346499 | 4.31561 |
| C | 17.62130 | 16.02310 | 4.29983 | C | 30.55742 | 25.949610 | 4.277456 | H | 19.00401 | 9.807080 | 4.31671 |
| C | 18.36413 | 17.27594 | 4.29988 | C | 31.28399 | 24.729030 | 4.271566 | H | 14.36972 | 14.857300 | 4.27623 |
| C | 17.66782 | 18.51419 | 4.28993 | C | 32.74047 | 24.749860 | 4.269486 | H | 20.30538 | 16.284170 | 4.31358 |
| C | 18.31922 | 14.79286 | 4.30835 | C | 33.43377 | 25.989830 | 4.276886 | H | 17.82740 | 20.688040 | 4.28003 |
| C | 17.57821 | 13.63059 | 4.30456 | C | 30.59929 | 23.491040 | 4.266536 | H | 19.42228 | 14.795420 | 4.31646 |
| C | 16.18489 | 13.64734 | 4.29346 | C | 29.22112 | 23.516340 | 4.268106 | H | 16.10089 | 20.708600 | 4.26715 |
| C | 15.47251 | 14.82721 | 4.28568 | C | 28.50915 | 24.714230 | 4.273706 | H | 13.52477 | 16.361480 | 4.26318 |
| C | 18.38235 | 19.73474 | 4.28780 | C | 29.14325 | 25.937600 | 4.278116 | H | 24.49257 | 24.050120 | 4.27516 |
| C | 19.75919 | 19.67534 | 4.29313 | C | 34.84806 | 26.017050 | 4.273636 | H | 13.52404 | 0.793766 | 4.28220 |
| C | 20.44278 | 18.46110 | 4.30178 | C | 35.51623 | 24.811580 | 4.260686 | H | 32.90746 | 22.576430 | 4.25295 |
| C | 19.77851 | 17.25331 | 4.30660 | C | 34.83562 | 23.595470 | 4.253056 | H | 20.30223 | 0.888561 | 4.28006 |
| O | 15.69530 | 12.28935 | 4.29015 | C | 33.45892 | 23.531680 | 4.258366 | H | 31.17735 | 22.551510 | 4.26166 |
| B | 16.85546 | 11.50839 | 4.29913 | O | 27.09608 | 24.425410 | 4.274506 | H | 19.41786 | 2.372697 | 4.29178 |
| O | 18.03423 | 12.26132 | 4.30883 | B | 27.03304 | 23.028350 | 4.269786 | H | 14.36531 | 2.299852 | 4.28311 |
| O | 12.02315 | 18.82814 | 4.23893 | O | 28.28916 | 22.413310 | 4.265166 | H | 22.35706 | 22.826880 | 4.28430 |
| O | 20.71485 | 20.75623 | 4.28758 | O | 15.68670 | 4.871904 | 4.292006 | H | 24.50556 | 19.077190 | 4.27868 |
| B | 21.95690 | 20.11355 | 4.29118 | O | 36.93746 | 24.558020 | 4.251286 | H | 26.64087 | 20.299930 | 4.26911 |

**c** 0.00000 0.00000 6.84080 serrated armchair

# COF-10

## Layer I AA, AB, inclined zigzag & armchair, and serrated zigzag & armchair

| | | | | | | | | | | | |
|---|---|---|---|---|---|---|---|---|---|---|---|
| B | 37.50977 | 27.53210 | 0.793766 | C | 19.43546 | 9.657603 | 0.793766 | C | 38.24199 | 20.51853 | 0.793766 |
| O | 36.34117 | 26.76319 | 0.793766 | C | 19.42151 | 7.265988 | 0.793766 | C | 40.32082 | 19.33411 | 0.793766 |
| C | 37.50737 | 29.02741 | 0.793766 | C | 20.14871 | 8.436213 | 0.793766 | C | 38.94325 | 19.28997 | 0.793766 |
| C | 36.30821 | 29.76566 | 0.793766 | H | 21.25162 | 8.448972 | 0.793766 | H | 38.37680 | 18.34322 | 0.793766 |
| C | 36.30917 | 31.15210 | 0.793766 | H | 20.89360 | 3.437906 | 0.793766 | H | 42.89956 | 16.15324 | 0.793766 |
| C | 37.51363 | 31.87336 | 0.793766 | H | 20.88455 | 0.961991 | 0.793766 | H | 45.04575 | 14.91717 | 0.793766 |
| C | 36.80304 | 23.00954 | 0.793766 | B | 13.72391 | 13.79473 | 0.793766 | O | 38.68137 | 26.76693 | 0.793766 |
| C | 36.81699 | 25.40116 | 0.793766 | O | 13.80313 | 12.39784 | 0.793766 | C | 38.70895 | 29.76133 | 0.793766 |
| C | 36.08978 | 24.23090 | 0.793766 | C | 12.42878 | 14.54264 | 0.793766 | C | 38.71519 | 31.14767 | 0.793766 |
| H | 34.98686 | 24.21814 | 0.793766 | C | 11.19060 | 13.87164 | 0.793766 | C | 38.22355 | 23.00970 | 0.793766 |
| H | 35.34490 | 29.22924 | 0.793766 | C | 9.988718 | 14.56286 | 0.793766 | C | 38.21038 | 25.40227 | 0.793766 |
| H | 35.35394 | 31.70512 | 0.793766 | C | 9.963801 | 15.96625 | 0.793766 | C | 38.93695 | 24.23109 | 0.793766 |

| | | | | | | | | | | | |
|---|---|---|---|---|---|---|---|---|---|---|---|
| B | 42.51460 | 18.87242 | 0.793766 | C | 17.28637 | 10.91834 | 0.793766 | H | 40.03979 | 24.21274 | 0.793766 |
| O | 42.43535 | 20.26927 | 0.793766 | C | 15.22095 | 12.12606 | 0.793766 | H | 39.66853 | 29.21889 | 0.793766 |
| C | 43.80972 | 18.12450 | 0.793766 | C | 15.87178 | 10.91112 | 0.793766 | H | 39.67292 | 31.69656 | 0.793766 |
| C | 45.04790 | 18.79551 | 0.793766 | H | 15.33533 | 9.947084 | 0.793766 | O | 23.64449 | 12.39598 | 0.793766 |
| C | 46.24976 | 18.10426 | 0.793766 | H | 11.17634 | 12.76915 | 0.793766 | C | 26.26091 | 13.86799 | 0.793766 |
| C | 46.27472 | 16.70086 | 0.793766 | H | 9.033188 | 14.01023 | 0.793766 | C | 27.46135 | 14.56172 | 0.793766 |
| C | 38.95214 | 21.74880 | 0.793766 | B | 23.72578 | 13.79248 | 0.793766 | C | 20.16314 | 10.91831 | 0.793766 |
| C | 41.01753 | 20.54105 | 0.793766 | O | 22.47722 | 14.42433 | 0.793766 | C | 22.22712 | 12.12662 | 0.793766 |
| C | 40.36674 | 21.75599 | 0.793766 | C | 25.02214 | 14.53776 | 0.793766 | C | 21.57750 | 10.91161 | 0.793766 |
| H | 40.90316 | 22.72006 | 0.793766 | C | 25.05735 | 15.94526 | 0.793766 | H | 22.11819 | 9.950241 | 0.793766 |
| H | 45.06214 | 19.89797 | 0.793766 | C | 26.25503 | 16.64345 | 0.793766 | H | 26.27779 | 12.76553 | 0.793766 |
| H | 47.20531 | 18.65692 | 0.793766 | C | 27.48411 | 15.96541 | 0.793766 | H | 28.41782 | 14.01078 | 0.793766 |
| B | 32.51270 | 18.87463 | 0.793766 | C | 19.45278 | 12.14849 | 0.793766 | O | 14.97309 | 14.42472 | 0.793766 |
| O | 33.76126 | 18.24278 | 0.793766 | C | 21.53118 | 13.33379 | 0.793766 | C | 12.39116 | 15.95047 | 0.793766 |
| C | 31.21635 | 18.12935 | 0.793766 | C | 20.15363 | 13.37717 | 0.793766 | C | 11.19181 | 16.64608 | 0.793766 |
| C | 31.18113 | 16.72185 | 0.793766 | H | 19.58600 | 14.32285 | 0.793766 | C | 17.99652 | 12.14862 | 0.793766 |
| C | 29.98345 | 16.02367 | 0.793766 | H | 24.10795 | 16.50531 | 0.793766 | C | 15.91767 | 13.33300 | 0.793766 |
| C | 28.75439 | 16.70174 | 0.793766 | H | 26.25166 | 17.74730 | 0.793766 | C | 17.29525 | 13.37717 | 0.793766 |
| C | 36.78572 | 20.51866 | 0.793766 | O | 32.59399 | 20.27113 | 0.793766 | H | 17.86168 | 14.32389 | 0.793766 |
| C | 34.70734 | 19.33333 | 0.793766 | C | 29.97759 | 18.79915 | 0.793766 | H | 13.33893 | 16.51387 | 0.793766 |
| C | 36.08487 | 19.28997 | 0.793766 | C | 28.77713 | 18.10540 | 0.793766 | H | 11.19275 | 17.74997 | 0.793766 |
| H | 36.65248 | 18.34426 | 0.793766 | C | 36.07534 | 21.74880 | 0.793766 | O | 17.55711 | 5.900183 | 0.793766 |
| H | 32.13055 | 16.16183 | 0.793766 | C | 34.01139 | 20.54050 | 0.793766 | C | 17.52954 | 2.905814 | 0.793766 |
| H | 29.98685 | 14.91981 | 0.793766 | C | 34.66100 | 21.75554 | 0.793766 | C | 17.52331 | 1.519471 | 0.793766 |
| B | 18.72871 | 5.135015 | 0.793766 | H | 34.12031 | 22.71691 | 0.793766 | C | 18.01493 | 9.657408 | 0.793766 |
| O | 19.89731 | 5.903926 | 0.793766 | H | 29.96070 | 19.90158 | 0.793766 | C | 18.02810 | 7.264849 | 0.793766 |
| C | 18.73113 | 3.639735 | 0.793766 | H | 27.82068 | 18.65637 | 0.793766 | C | 17.30153 | 8.436018 | 0.793766 |
| C | 19.93029 | 2.901485 | 0.793766 | O | 41.26539 | 18.24239 | 0.793766 | H | 16.19871 | 8.454407 | 0.793766 |
| C | 19.92932 | 1.515012 | 0.793766 | C | 43.84734 | 16.71668 | 0.793766 | H | 16.56996 | 3.448223 | 0.793766 |
| C | 18.72487 | 0.793784 | 0.793766 | C | 45.04671 | 16.02103 | 0.793766 | H | 16.56556 | 0.970551 | 0.793766 |

Transformation Matrix

| | | | | |
|---|---|---|---|---|
| **a** | 37.583030 | 0.000000 | 0.000000 | |
| **b** | 18.791520 | 32.547840 | 0.000000 | |
| **c** | 0.000000 | 0.000000 | 3.472000 | AA |
| **c** | 1.203000 | 0.694552 | 3.398119 | inclined zigzag |
| **c** | 1.387895 | 0.00000 | 3.420000 | inclined armchair |

## Layer II AB stacking

| | | | | | | | | | | | |
|---|---|---|---|---|---|---|---|---|---|---|---|
| B | 56.30128 | 38.38139 | 3.970638 | C | 38.22697 | 20.50689 | 3.970638 | C | 57.03350 | 31.36782 | 3.970638 |
| O | 55.13269 | 37.61248 | 3.970638 | C | 38.21303 | 18.11527 | 3.970638 | C | 59.11233 | 30.18340 | 3.970638 |
| C | 56.29889 | 39.87670 | 3.970638 | C | 38.94022 | 19.28550 | 3.970638 | C | 57.73476 | 30.13926 | 3.970638 |
| C | 55.09972 | 40.61494 | 3.970638 | H | 40.04314 | 19.29826 | 3.970638 | H | 57.16831 | 29.19251 | 3.970638 |
| C | 55.10069 | 42.00139 | 3.970638 | H | 39.68512 | 14.28719 | 3.970638 | H | 61.69107 | 27.00253 | 3.970638 |
| C | 56.30515 | 42.72265 | 3.970638 | H | 39.67606 | 11.81128 | 3.970638 | H | 63.83727 | 25.76646 | 3.970638 |
| C | 55.59455 | 33.85883 | 3.970638 | B | 32.51543 | 24.64402 | 3.970638 | O | 57.47289 | 37.61622 | 3.970638 |
| C | 55.60851 | 36.25045 | 3.970638 | O | 32.59465 | 23.24713 | 3.970638 | C | 57.50047 | 40.61062 | 3.970638 |
| C | 54.88129 | 35.08018 | 3.970638 | C | 31.22029 | 25.39193 | 3.970638 | C | 57.50671 | 41.99696 | 3.970638 |
| H | 53.77837 | 35.06743 | 3.970638 | C | 29.98212 | 24.72093 | 3.970638 | C | 57.01506 | 33.85899 | 3.970638 |
| H | 54.13641 | 40.07853 | 3.970638 | C | 28.78023 | 25.41215 | 3.970638 | C | 57.00190 | 36.25156 | 3.970638 |
| H | 54.14545 | 42.55441 | 3.970638 | C | 28.75532 | 26.81554 | 3.970638 | C | 57.72847 | 35.08038 | 3.970638 |
| B | 61.30611 | 29.72171 | 3.970638 | C | 36.07788 | 21.76763 | 3.970638 | H | 58.83131 | 35.06203 | 3.970638 |
| O | 61.22686 | 31.11856 | 3.970638 | C | 34.01247 | 22.97535 | 3.970638 | H | 58.46004 | 40.06818 | 3.970638 |
| C | 62.60124 | 28.97379 | 3.970638 | C | 34.66330 | 21.76041 | 3.970638 | H | 58.46444 | 42.54585 | 3.970638 |
| C | 63.83941 | 29.64480 | 3.970638 | H | 34.12685 | 20.79637 | 3.970638 | O | 42.43600 | 23.24527 | 3.970638 |
| C | 65.04128 | 28.95355 | 3.970638 | H | 29.96786 | 23.61844 | 3.970638 | C | 45.05243 | 24.71728 | 3.970638 |
| C | 65.06624 | 27.55015 | 3.970638 | H | 27.82470 | 24.85952 | 3.970638 | C | 46.25286 | 25.41101 | 3.970638 |
| C | 57.74366 | 32.59809 | 3.970638 | B | 42.51730 | 24.64177 | 3.970638 | C | 38.95465 | 21.76760 | 3.970638 |
| C | 59.80904 | 31.39034 | 3.970638 | O | 41.26874 | 25.27362 | 3.970638 | C | 41.01863 | 22.97591 | 3.970638 |
| C | 59.15826 | 32.60528 | 3.970638 | C | 43.81366 | 25.38705 | 3.970638 | C | 40.36901 | 21.76090 | 3.970638 |
| H | 59.69468 | 33.56935 | 3.970638 | C | 43.84887 | 26.79455 | 3.970638 | H | 40.90971 | 20.79953 | 3.970638 |
| H | 63.85366 | 30.74726 | 3.970638 | C | 45.04655 | 27.49274 | 3.970638 | H | 45.06931 | 23.61482 | 3.970638 |

| | | | | | | | | | | | |
|---|---|---|---|---|---|---|---|---|---|---|---|
| H | 65.99683 | 29.50621 | 3.970638 | C | 46.27563 | 26.81470 | 3.970638 | H | 47.20934 | 24.86007 | 3.970638 |
| B | 51.30421 | 29.72392 | 3.970638 | C | 38.24429 | 22.99778 | 3.970638 | O | 33.76461 | 25.27401 | 3.970638 |
| O | 52.55278 | 29.09207 | 3.970638 | C | 40.32269 | 24.18308 | 3.970638 | C | 31.18267 | 26.79976 | 3.970638 |
| C | 50.00787 | 28.97864 | 3.970638 | C | 38.94514 | 24.22646 | 3.970638 | C | 29.98333 | 27.49537 | 3.970638 |
| C | 49.97264 | 27.57114 | 3.970638 | H | 38.37752 | 25.17214 | 3.970638 | C | 36.78803 | 22.99791 | 3.970638 |
| C | 48.77496 | 26.87296 | 3.970638 | H | 42.89947 | 27.35460 | 3.970638 | C | 34.70919 | 24.18229 | 3.970638 |
| C | 47.54591 | 27.55103 | 3.970638 | H | 45.04317 | 28.59659 | 3.970638 | C | 36.08677 | 24.22646 | 3.970638 |
| C | 55.57724 | 31.36795 | 3.970638 | O | 51.38551 | 31.12042 | 3.970638 | H | 36.65319 | 25.17318 | 3.970638 |
| C | 53.49886 | 30.18262 | 3.970638 | C | 48.76910 | 29.64844 | 3.970638 | H | 32.13045 | 27.36316 | 3.970638 |
| C | 54.87638 | 30.13926 | 3.970638 | C | 47.56865 | 28.95469 | 3.970638 | H | 29.98426 | 28.59926 | 3.970638 |
| H | 55.44400 | 29.19355 | 3.970638 | C | 54.86686 | 32.59809 | 3.970638 | O | 36.34863 | 16.74947 | 3.970638 |
| H | 50.92207 | 27.01112 | 3.970638 | C | 52.80291 | 31.38979 | 3.970638 | C | 36.32105 | 13.75510 | 3.970638 |
| H | 48.77837 | 25.76910 | 3.970638 | C | 53.45251 | 32.60483 | 3.970638 | C | 36.31483 | 12.36876 | 3.970638 |
| B | 37.52023 | 15.98430 | 3.970638 | H | 52.91183 | 33.56620 | 3.970638 | C | 36.80645 | 20.50669 | 3.970638 |
| O | 38.68883 | 16.75321 | 3.970638 | H | 48.75221 | 30.75087 | 3.970638 | C | 36.81961 | 18.11414 | 3.970638 |
| C | 37.52264 | 14.48902 | 3.970638 | H | 46.61220 | 29.50566 | 3.970638 | C | 36.09305 | 19.28531 | 3.970638 |
| C | 38.72181 | 13.75077 | 3.970638 | O | 60.05690 | 29.09168 | 3.970638 | H | 34.99023 | 19.30369 | 3.970638 |
| C | 38.72083 | 12.36430 | 3.970638 | C | 62.63886 | 27.56597 | 3.970638 | H | 35.36147 | 14.29751 | 3.970638 |
| C | 37.51639 | 11.64307 | 3.970638 | C | 63.83823 | 26.87032 | 3.970638 | H | 35.35707 | 11.81984 | 3.970638 |

**c** 0.00000 0.00000 6.35374 AB

## Layer II serrated zigzag

| | | | | | | | | | | | |
|---|---|---|---|---|---|---|---|---|---|---|---|
| B | 38.71277 | 28.226650 | 4.206926 | C | 20.63846 | 10.35216 | 4.206926 | C | 39.44499 | 21.213080 | 4.206926 |
| O | 37.54417 | 27.457740 | 4.206926 | C | 20.62451 | 7.96054 | 4.206926 | C | 41.52382 | 20.028660 | 4.206926 |
| C | 38.71037 | 29.721960 | 4.206926 | C | 21.35171 | 9.13076 | 4.206926 | C | 40.14625 | 19.984520 | 4.206926 |
| C | 37.51121 | 30.460210 | 4.206926 | H | 22.45462 | 9.14352 | 4.206926 | H | 39.57980 | 19.037770 | 4.206926 |
| C | 37.51217 | 31.846650 | 4.206926 | H | 22.09660 | 4.13246 | 4.206926 | H | 44.10255 | 16.847790 | 4.206926 |
| C | 19.92511 | 0.020070 | 4.206926 | H | 22.08755 | 1.65654 | 4.206926 | H | 46.24875 | 15.611720 | 4.206926 |
| C | 38.00604 | 23.704090 | 4.206926 | B | 14.92691 | 14.48928 | 4.206926 | O | 39.88437 | 27.461480 | 4.206926 |
| C | 38.01999 | 26.095710 | 4.206926 | O | 15.00613 | 13.09239 | 4.206926 | C | 39.91195 | 30.455880 | 4.206926 |
| C | 37.29278 | 24.925450 | 4.206926 | C | 13.63178 | 15.23719 | 4.206926 | C | 39.91819 | 31.842220 | 4.206926 |
| H | 36.18986 | 24.912690 | 4.206926 | C | 12.39360 | 14.56619 | 4.206926 | C | 39.42655 | 23.704250 | 4.206926 |
| H | 36.54790 | 29.923790 | 4.206926 | C | 11.19172 | 15.25741 | 4.206926 | C | 39.41338 | 26.096820 | 4.206926 |
| H | 36.55694 | 32.399670 | 4.206926 | C | 11.16680 | 16.66080 | 4.206926 | C | 40.13995 | 24.925640 | 4.206926 |
| B | 43.71759 | 19.566970 | 4.206926 | C | 18.48937 | 11.61289 | 4.206926 | H | 41.24279 | 24.907290 | 4.206926 |
| O | 43.63835 | 20.963820 | 4.206926 | C | 16.42395 | 12.82061 | 4.206926 | H | 40.87153 | 29.913440 | 4.206926 |
| C | 45.01272 | 18.819050 | 4.206926 | C | 17.07478 | 11.60567 | 4.206926 | H | 40.87592 | 32.391110 | 4.206926 |
| C | 46.25090 | 19.490060 | 4.206926 | H | 16.53833 | 10.64164 | 4.206926 | O | 24.84749 | 13.090530 | 4.206926 |
| C | 47.45276 | 18.798810 | 4.206926 | H | 12.37934 | 13.46370 | 4.206926 | C | 27.46391 | 14.562540 | 4.206926 |
| C | 47.47772 | 17.395410 | 4.206926 | H | 10.23619 | 14.70478 | 4.206926 | C | 28.66435 | 15.256270 | 4.206926 |
| C | 40.15514 | 22.443350 | 4.206926 | B | 24.92878 | 14.48703 | 4.206926 | C | 21.36614 | 11.612860 | 4.206926 |
| C | 42.22053 | 21.235600 | 4.206926 | O | 23.68022 | 15.11888 | 4.206926 | C | 23.43012 | 12.821170 | 4.206926 |
| C | 41.56974 | 22.450540 | 4.206926 | C | 26.22514 | 15.23231 | 4.206926 | C | 22.78050 | 11.606160 | 4.206926 |
| H | 42.10616 | 23.414610 | 4.206926 | C | 26.26035 | 16.63981 | 4.206926 | H | 23.32119 | 10.644790 | 4.206926 |
| H | 46.26514 | 20.592520 | 4.206926 | C | 27.45803 | 17.33800 | 4.206926 | H | 27.48079 | 13.460080 | 4.206926 |
| H | 48.40831 | 19.351470 | 4.206926 | C | 28.68711 | 16.65996 | 4.206926 | H | 29.62082 | 14.705330 | 4.206926 |
| B | 33.71570 | 19.569180 | 4.206926 | C | 20.65578 | 12.84304 | 4.206926 | O | 16.17609 | 15.119270 | 4.206926 |
| O | 34.96426 | 18.937330 | 4.206926 | C | 22.73418 | 14.02834 | 4.206926 | C | 13.59416 | 16.645020 | 4.206926 |
| C | 32.41935 | 18.823900 | 4.206926 | C | 21.35663 | 14.07172 | 4.206926 | C | 12.39481 | 17.340630 | 4.206926 |
| C | 32.38413 | 17.416400 | 4.206926 | H | 20.78900 | 15.01740 | 4.206926 | C | 19.19952 | 12.843170 | 4.206926 |
| C | 31.18645 | 16.718220 | 4.206926 | H | 25.31095 | 17.19986 | 4.206926 | C | 17.12067 | 14.027550 | 4.206926 |
| C | 29.95739 | 17.396290 | 4.206926 | H | 27.45466 | 18.44185 | 4.206926 | C | 18.49825 | 14.071720 | 4.206926 |
| C | 37.98872 | 21.213210 | 4.206926 | O | 33.79699 | 20.96568 | 4.206926 | H | 19.06468 | 15.018440 | 4.206926 |
| C | 35.91034 | 20.027880 | 4.206926 | C | 31.18059 | 19.49370 | 4.206926 | H | 14.54193 | 17.208420 | 4.206926 |
| C | 37.28787 | 19.984520 | 4.206926 | C | 29.98013 | 18.79995 | 4.206926 | H | 12.39575 | 18.444520 | 4.206926 |
| H | 37.85548 | 19.038810 | 4.206926 | C | 37.27834 | 22.44335 | 4.206926 | O | 18.76011 | 6.594735 | 4.206926 |
| H | 33.33355 | 16.856380 | 4.206926 | C | 35.21439 | 21.23505 | 4.206926 | C | 18.73254 | 3.600366 | 4.206926 |
| H | 31.18985 | 15.614360 | 4.206926 | C | 35.86400 | 22.45009 | 4.206926 | C | 18.72631 | 2.214023 | 4.206926 |
| B | 19.93171 | 5.829567 | 4.206926 | H | 35.32331 | 23.41146 | 4.206926 | C | 19.21793 | 10.351960 | 4.206926 |
| O | 21.10031 | 6.598478 | 4.206926 | H | 31.16370 | 20.59613 | 4.206926 | C | 19.23110 | 7.959401 | 4.206926 |
| C | 19.93413 | 4.334287 | 4.206926 | H | 29.02368 | 19.35092 | 4.206926 | C | 18.50453 | 9.130570 | 4.206926 |
| C | 21.13329 | 3.596037 | 4.206926 | O | 42.46839 | 18.93694 | 4.206926 | H | 17.40171 | 9.148959 | 4.206926 |
| C | 21.13232 | 2.209564 | 4.206926 | C | 45.05034 | 17.41123 | 4.206926 | H | 17.77296 | 4.142775 | 4.206926 |

| | | | | | | | | | | | |
|---|---|---|---|---|---|---|---|---|---|---|---|
| C | 19.92787 | 1.488336 | 4.206926 | C | 46.24971 | 16.71558 | 4.206926 | H | 17.76856 | 1.665103 | 4.206926 |

**c** 0.00000 0.00000 6.82632 serrated zigzag

## Layer II serrated armchair

| | | | | | | | | | | | |
|---|---|---|---|---|---|---|---|---|---|---|---|
| B | 38.89766 | 27.532100 | 4.20692 | C | 20.82335 | 9.657603 | 4.20692 | C | 39.62988 | 20.518530 | 4.20692 |
| O | 37.72906 | 26.763190 | 4.20692 | C | 20.80941 | 7.265988 | 4.20692 | C | 41.70871 | 19.334110 | 4.20692 |
| C | 38.89526 | 29.027410 | 4.20692 | C | 21.53661 | 8.436213 | 4.20692 | C | 40.33114 | 19.289970 | 4.20692 |
| C | 37.69610 | 29.765660 | 4.20692 | H | 22.63951 | 8.448972 | 4.20692 | H | 39.76469 | 18.343220 | 4.20692 |
| C | 37.69706 | 31.152100 | 4.20692 | H | 22.28150 | 3.437906 | 4.20692 | H | 44.28745 | 16.153240 | 4.20692 |
| C | 38.90152 | 31.873360 | 4.20692 | H | 22.27245 | 0.961991 | 4.20692 | H | 8.850620 | 14.917170 | 4.20692 |
| C | 38.19093 | 23.009540 | 4.20692 | B | 15.11180 | 13.794730 | 4.20692 | O | 40.06926 | 26.766930 | 4.20692 |
| C | 38.20488 | 25.401160 | 4.20692 | O | 15.19102 | 12.397840 | 4.20692 | C | 40.09684 | 29.761330 | 4.20692 |
| C | 37.47767 | 24.230900 | 4.20692 | C | 13.81667 | 14.542640 | 4.20692 | C | 40.10308 | 31.147670 | 4.20692 |
| H | 36.37475 | 24.218140 | 4.20692 | C | 12.57850 | 13.871640 | 4.20692 | C | 39.61144 | 23.009700 | 4.20692 |
| H | 36.73279 | 29.229240 | 4.20692 | C | 11.37661 | 14.562860 | 4.20692 | C | 39.59827 | 25.402270 | 4.20692 |
| H | 36.74183 | 31.705120 | 4.20692 | C | 11.35170 | 15.966250 | 4.20692 | C | 40.32484 | 24.231090 | 4.20692 |
| B | 43.90249 | 18.872420 | 4.20692 | C | 18.67426 | 10.918340 | 4.20692 | H | 41.42768 | 24.212740 | 4.20692 |
| O | 43.82324 | 20.269270 | 4.20692 | C | 16.60884 | 12.126060 | 4.20692 | H | 41.05642 | 29.218890 | 4.20692 |
| C | 45.19762 | 18.124500 | 4.20692 | C | 17.25968 | 10.911120 | 4.20692 | H | 41.06081 | 31.696560 | 4.20692 |
| C | 46.43579 | 18.795510 | 4.20692 | H | 16.72322 | 9.947084 | 4.20692 | O | 25.03238 | 12.395980 | 4.20692 |
| C | 47.63766 | 18.104260 | 4.20692 | H | 12.56423 | 12.769150 | 4.20692 | C | 27.64881 | 13.867990 | 4.20692 |
| C | 10.07958 | 16.700860 | 4.20692 | H | 10.42108 | 14.010230 | 4.20692 | C | 28.84925 | 14.561720 | 4.20692 |
| C | 40.34003 | 21.748800 | 4.20692 | B | 25.11368 | 13.792480 | 4.20692 | C | 21.55103 | 10.918310 | 4.20692 |
| C | 42.40542 | 20.541050 | 4.20692 | O | 23.86512 | 14.424330 | 4.20692 | C | 23.61502 | 12.126620 | 4.20692 |
| C | 41.75463 | 21.755990 | 4.20692 | C | 26.41004 | 14.537760 | 4.20692 | C | 22.96539 | 10.911610 | 4.20692 |
| H | 42.29106 | 22.720060 | 4.20692 | C | 26.44525 | 15.945260 | 4.20692 | H | 23.50609 | 9.950241 | 4.20692 |
| H | 46.45004 | 19.897970 | 4.20692 | C | 27.64293 | 16.643450 | 4.20692 | H | 27.66569 | 12.765530 | 4.20692 |
| H | 11.01017 | 18.656920 | 4.20692 | C | 28.87201 | 15.965410 | 4.20692 | H | 29.80572 | 14.010780 | 4.20692 |
| B | 33.90059 | 18.874630 | 4.20692 | C | 20.84068 | 12.148490 | 4.20692 | O | 16.36098 | 14.424720 | 4.20692 |
| O | 35.14915 | 18.242780 | 4.20692 | C | 22.91908 | 13.333790 | 4.20692 | C | 13.77905 | 15.950470 | 4.20692 |
| C | 32.60424 | 18.129350 | 4.20692 | C | 21.54152 | 13.377170 | 4.20692 | C | 12.57970 | 16.646080 | 4.20692 |
| C | 32.56902 | 16.721850 | 4.20692 | H | 20.97390 | 14.322850 | 4.20692 | C | 19.38441 | 12.148620 | 4.20692 |
| C | 31.37135 | 16.023670 | 4.20692 | H | 25.49585 | 16.505310 | 4.20692 | C | 17.30556 | 13.333000 | 4.20692 |
| C | 30.14229 | 16.701740 | 4.20692 | H | 27.63955 | 17.747300 | 4.20692 | C | 18.68315 | 13.377170 | 4.20692 |
| C | 38.17361 | 20.518660 | 4.20692 | O | 33.98188 | 20.271130 | 4.20692 | H | 19.24957 | 14.323890 | 4.20692 |
| C | 36.09523 | 19.333330 | 4.20692 | C | 31.36549 | 18.799150 | 4.20692 | H | 14.72682 | 16.513870 | 4.20692 |
| C | 37.47276 | 19.289970 | 4.20692 | C | 30.16503 | 18.105400 | 4.20692 | H | 12.58064 | 17.749970 | 4.20692 |
| H | 38.04037 | 18.344260 | 4.20692 | C | 37.46323 | 21.748800 | 4.20692 | O | 18.94501 | 5.900183 | 4.20692 |
| H | 33.51844 | 16.161830 | 4.20692 | C | 35.39928 | 20.540500 | 4.20692 | C | 18.91743 | 2.905814 | 4.20692 |
| H | 31.37475 | 14.919810 | 4.20692 | C | 36.04889 | 21.755540 | 4.20692 | C | 18.91121 | 1.519471 | 4.20692 |
| B | 20.11661 | 5.135015 | 4.20692 | H | 35.50821 | 22.716910 | 4.20692 | C | 19.40283 | 9.657408 | 4.20692 |
| O | 21.28521 | 5.903926 | 4.20692 | H | 31.34859 | 19.901580 | 4.20692 | C | 19.41599 | 7.264849 | 4.20692 |
| C | 20.11903 | 3.639735 | 4.20692 | H | 29.20858 | 18.656370 | 4.20692 | C | 18.68943 | 8.436018 | 4.20692 |
| C | 21.31819 | 2.901485 | 4.20692 | O | 42.65328 | 18.242390 | 4.20692 | H | 17.58661 | 8.454407 | 4.20692 |
| C | 21.31721 | 1.515012 | 4.20692 | C | 45.23524 | 16.716680 | 4.20692 | H | 17.95786 | 3.448223 | 4.20692 |
| C | 20.11277 | 0.793784 | 4.20692 | C | 46.43460 | 16.021030 | 4.20692 | H | 17.95345 | 0.970551 | 4.20692 |

**c** 0.00000 0.00000 6.82632 serrated armchair

# COF-8

## Layer I AA, AB, inclined zigzag & armchair, and serrated zigzag & armchair

| | | | | | | | | | | | |
|---|---|---|---|---|---|---|---|---|---|---|---|
| C | 25.85511 | 6.863348 | 7.860975 | C | 16.93204 | 15.12143 | 7.789575 | C | 12.00871 | 9.051265 | 7.980750 |
| C | 4.564046 | 7.551417 | 7.906900 | C | 17.67002 | 16.28532 | 7.798175 | C | 10.76552 | 9.712545 | 7.984575 |
| C | 27.04152 | 8.953955 | 7.932275 | C | 19.76935 | 12.61187 | 7.842400 | C | 9.568823 | 9.011315 | 7.998150 |
| C | 25.83477 | 9.636429 | 7.915000 | C | 19.04838 | 11.38808 | 7.839901 | H | 4.968770 | 0.593880 | 7.773575 |
| C | 24.60197 | 8.957348 | 7.872050 | C | 17.59200 | 11.40208 | 7.842575 | H | 9.290070 | 0.621960 | 7.924825 |
| C | 24.65039 | 7.550442 | 7.845175 | C | 16.89313 | 12.63879 | 7.824075 | H | 15.82916 | 15.11616 | 7.771775 |
| C | 5.845753 | 6.828019 | 7.928650 | C | 19.73875 | 10.15314 | 7.840800 | H | 21.73021 | 13.56432 | 7.858100 |
| C | 5.881123 | 5.428016 | 7.906675 | C | 21.11665 | 10.18468 | 7.845775 | H | 19.16490 | 9.211218 | 7.837525 |

| | | | | | | | | | | | |
|---|---|---|---|---|---|---|---|---|---|---|---|
| C | 7.094083 | 4.727273 | 7.928900 | C | 21.82318 | 11.38555 | 7.855025 | H | 20.88093 | 15.06828 | 7.863150 |
| C | 8.288683 | 5.457203 | 7.971675 | C | 21.18377 | 12.60627 | 7.853175 | H | 17.43520 | 9.228512 | 7.890950 |
| C | 8.288533 | 6.858084 | 7.988900 | C | 15.47862 | 12.65933 | 7.834900 | H | 14.95035 | 13.62760 | 7.821225 |
| C | 7.058663 | 7.527767 | 7.969375 | C | 14.81638 | 11.45139 | 7.872100 | H | 9.268163 | 3.098184 | 7.972725 |
| C | 7.109643 | 3.255638 | 7.901625 | C | 15.50221 | 10.23842 | 7.891925 | H | 4.953673 | 3.068884 | 7.821875 |
| C | 5.913613 | 2.524094 | 7.844950 | C | 16.87893 | 10.18088 | 7.874175 | H | 4.931608 | 4.862563 | 7.870025 |
| C | 5.926420 | 1.138050 | 7.817825 | O | 22.05292 | 9.084995 | 7.845775 | H | 7.043953 | 8.633483 | 7.982450 |
| C | 7.130610 | 0.408700 | 7.844975 | B | 23.30700 | 9.705273 | 7.859050 | H | 9.253453 | 4.917077 | 7.986850 |
| C | 8.324480 | 1.153370 | 7.901100 | O | 23.23795 | 11.10286 | 7.864675 | H | 10.79771 | 5.834665 | 8.025425 |
| C | 8.316553 | 2.540254 | 7.928675 | O | 17.20934 | 17.65298 | 7.790775 | H | 12.93357 | 7.087914 | 8.002875 |
| B | 18.38553 | 18.41036 | 7.823425 | O | 13.39593 | 11.19266 | 7.904350 | H | 10.74185 | 10.81479 | 7.972126 |
| O | 19.54858 | 17.63286 | 7.843225 | B | 13.30443 | 9.797026 | 7.944225 | H | 8.609103 | 9.556862 | 7.996300 |
| C | 19.06316 | 16.27273 | 7.827525 | O | 14.54852 | 9.156354 | 7.937501 | H | 27.99298 | 9.512935 | 7.966525 |
| C | 19.77834 | 15.09475 | 7.841700 | C | 9.554843 | 7.608115 | 8.006650 | H | 25.84072 | 10.73869 | 7.936950 |
| C | 19.05345 | 13.87995 | 7.831375 | C | 10.78790 | 6.938528 | 8.014576 | H | 23.70758 | 6.980115 | 7.809900 |
| C | 17.63319 | 13.89307 | 7.810275 | C | 11.98140 | 7.643658 | 8.001800 | H | 3.348264 | 5.759680 | 7.837625 |

Transformation Matrix

| | | | | |
|---|---|---|---|---|
| **a** | 22.51323 | 0.00000 | 0.00000 | |
| **b** | 11.25662 | 19.49702 | 0.00000 | |
| **c** | 0.000000 | 0.000000 | 3.476572 | AA |
| **c** | 1.199774 | 0.69269 | 3.42000 | inclined zigzag |
| **c** | 1.388105 | 0.00000 | 3.42342 | inclined armchair |

## Layer II AB stacking

| | | | | | | | | | | | |
|---|---|---|---|---|---|---|---|---|---|---|---|
| C | 37.11172 | 13.36236 | 11.06289 | C | 28.18865 | 21.62044 | 10.99150 | C | 23.26532 | 15.55027 | 11.18267 |
| C | 15.82066 | 14.05043 | 11.10882 | C | 28.92663 | 22.78433 | 11.00010 | C | 22.02213 | 16.21156 | 11.18649 |
| C | 38.29814 | 15.45296 | 11.13420 | C | 31.02596 | 19.11088 | 11.04432 | C | 20.82544 | 15.51032 | 11.20007 |
| C | 37.09138 | 16.13544 | 11.11692 | C | 30.30499 | 17.88709 | 11.04182 | H | 16.22538 | 7.092890 | 10.97549 |
| C | 35.85858 | 15.45636 | 11.07397 | C | 28.84861 | 17.90109 | 11.04449 | H | 20.54668 | 7.120970 | 11.12675 |
| C | 35.90701 | 14.04945 | 11.04709 | C | 28.14974 | 19.13780 | 11.02600 | H | 27.08577 | 21.61517 | 10.97369 |
| C | 17.10237 | 13.32703 | 11.13057 | C | 30.99537 | 16.65215 | 11.04272 | H | 32.98682 | 20.06333 | 11.06002 |
| C | 17.13774 | 11.92703 | 11.10859 | C | 32.37326 | 16.68369 | 11.04770 | H | 30.42151 | 15.71023 | 11.03944 |
| C | 18.35070 | 11.22628 | 11.13082 | C | 33.07980 | 17.88456 | 11.05694 | H | 32.13754 | 21.56729 | 11.06507 |
| C | 19.54530 | 11.95621 | 11.17360 | C | 32.44038 | 19.10528 | 11.05510 | H | 28.69181 | 15.72752 | 11.09287 |
| C | 19.54515 | 13.35709 | 11.19082 | C | 26.73523 | 19.15834 | 11.03682 | H | 26.20696 | 20.12661 | 11.02315 |
| C | 18.31528 | 14.02678 | 11.17130 | C | 26.07299 | 17.95040 | 11.07402 | H | 20.52478 | 9.597194 | 11.17464 |
| C | 18.36626 | 9.754647 | 11.10355 | C | 26.75882 | 16.73743 | 11.09385 | H | 16.21029 | 9.567894 | 11.02380 |
| C | 17.17023 | 9.023104 | 11.04687 | C | 28.13554 | 16.67989 | 11.07610 | H | 16.18822 | 11.36157 | 11.07195 |
| C | 17.18303 | 7.637060 | 11.01974 | O | 33.30953 | 15.58401 | 11.04770 | H | 18.30057 | 15.13249 | 11.18437 |
| C | 18.38722 | 6.907710 | 11.04690 | B | 34.56361 | 16.20428 | 11.06097 | H | 20.51007 | 11.41609 | 11.18877 |
| C | 19.58109 | 7.652380 | 11.10302 | O | 34.49456 | 17.60187 | 11.06660 | H | 22.05433 | 12.33367 | 11.22734 |
| C | 19.57317 | 9.039264 | 11.13060 | O | 28.46595 | 24.15199 | 10.99269 | H | 24.19019 | 13.58692 | 11.20480 |
| B | 29.64215 | 24.90937 | 11.02534 | O | 24.65255 | 17.69167 | 11.10627 | H | 21.99846 | 17.31380 | 11.17405 |
| O | 30.80519 | 24.13187 | 11.04515 | B | 24.56104 | 16.29604 | 11.14614 | H | 19.86572 | 16.05587 | 11.19822 |
| C | 30.31977 | 22.77174 | 11.02945 | O | 25.80513 | 15.65536 | 11.13942 | H | 39.24960 | 16.01194 | 11.16845 |
| C | 31.03495 | 21.59376 | 11.04362 | C | 20.81146 | 14.10712 | 11.20857 | H | 37.09734 | 17.23770 | 11.13887 |
| C | 30.31006 | 20.37896 | 11.03329 | C | 22.04451 | 13.43754 | 11.21650 | H | 34.96420 | 13.47912 | 11.01182 |
| C | 28.88980 | 20.39208 | 11.01220 | C | 23.23801 | 14.14267 | 11.20372 | H | 14.60488 | 12.25869 | 11.03955 |

| | | | | |
|---|---|---|---|---|
| **c** | 0.00000 | 0.00000 | 6.40384 | AB |

## Layer II serrated zigzag

| | | | | | | | | | | | |
|---|---|---|---|---|---|---|---|---|---|---|---|
| C | 27.07536 | 7.567857 | 11.26808 | C | 18.15228 | 15.82594 | 11.19668 | C | 13.22896 | 9.755774 | 11.38786 |
| C | 5.784291 | 8.255926 | 11.31401 | C | 18.89026 | 16.98983 | 11.20529 | C | 11.98577 | 10.41705 | 11.39168 |
| C | 28.26176 | 9.658463 | 11.33939 | C | 20.98960 | 13.31638 | 11.24951 | C | 10.78907 | 9.715824 | 11.40526 |
| C | 27.05502 | 10.34094 | 11.32211 | C | 20.26863 | 12.09259 | 11.24701 | H | 6.189015 | 1.298389 | 11.18069 |
| C | 25.82222 | 9.661857 | 11.27916 | C | 18.81224 | 12.10659 | 11.24969 | H | 10.51031 | 1.326469 | 11.33193 |
| C | 25.87064 | 8.254951 | 11.25229 | C | 18.11337 | 13.34330 | 11.23118 | H | 17.04940 | 15.82067 | 11.17888 |
| C | 7.065998 | 7.532528 | 11.33576 | C | 20.95900 | 10.85765 | 11.24791 | H | 22.95045 | 14.26883 | 11.26521 |
| C | 7.101368 | 6.132525 | 11.31378 | C | 22.33689 | 10.88919 | 11.25288 | H | 20.38515 | 9.915727 | 11.24463 |
| C | 8.314328 | 5.431782 | 11.33601 | C | 23.04342 | 12.09006 | 11.26213 | H | 22.10118 | 15.77279 | 11.27026 |
| C | 9.508928 | 6.161712 | 11.37879 | C | 22.40401 | 13.31078 | 11.26029 | H | 18.65545 | 9.933021 | 11.29806 |

| | | | | | | | | | | | |
|---|---|---|---|---|---|---|---|---|---|---|---|
| C | 9.508779 | 7.562593 | 11.39601 | C | 16.69886 | 13.36384 | 11.24201 | H | 16.17060 | 14.33211 | 11.22834 |
| C | 8.278908 | 8.232276 | 11.37648 | C | 16.03662 | 12.15590 | 11.27921 | H | 10.48841 | 3.802693 | 11.37984 |
| C | 8.329888 | 3.960147 | 11.30873 | C | 16.72245 | 10.94293 | 11.29904 | H | 6.173918 | 3.773393 | 11.22898 |
| C | 7.133858 | 3.228603 | 11.25206 | C | 18.09917 | 10.88539 | 11.28129 | H | 6.151853 | 5.567072 | 11.27713 |
| C | 7.146665 | 1.842559 | 11.22493 | O | 23.27316 | 9.789504 | 11.25288 | H | 8.264198 | 9.337992 | 11.38956 |
| C | 8.350855 | 1.113209 | 11.25208 | B | 24.52724 | 10.40978 | 11.26616 | H | 10.47370 | 5.621586 | 11.39396 |
| C | 9.544725 | 1.857879 | 11.30821 | O | 24.45819 | 11.80737 | 11.27178 | H | 12.01796 | 6.539174 | 11.43254 |
| C | 9.536798 | 3.244763 | 11.33578 | O | 18.42958 | 18.35749 | 11.19788 | H | 14.15382 | 7.792423 | 11.40999 |
| B | 19.60578 | 19.11487 | 11.23053 | O | 14.61618 | 11.89717 | 11.31146 | H | 11.96210 | 11.51930 | 11.37924 |
| O | 20.76883 | 18.33737 | 11.25033 | B | 14.52468 | 10.50153 | 11.35133 | H | 9.829349 | 10.26137 | 11.40341 |
| C | 20.28341 | 16.97724 | 11.23464 | O | 15.76877 | 9.860863 | 11.34461 | H | 29.21322 | 10.21744 | 11.37364 |
| C | 20.99858 | 15.79926 | 11.24881 | C | 10.77509 | 8.312624 | 11.41376 | H | 27.06096 | 11.44320 | 11.34406 |
| C | 20.27369 | 14.58446 | 11.23849 | C | 12.00815 | 7.643037 | 11.42169 | H | 24.92783 | 7.684624 | 11.21701 |
| C | 18.85344 | 14.59758 | 11.21739 | C | 13.20164 | 8.348167 | 11.40891 | H | 4.568509 | 6.464189 | 11.24473 |

**c** 0.00000 0.00000 6.81422 serrated zigzag

## Layer II serrated armchair

| | | | | | | | | | | | |
|---|---|---|---|---|---|---|---|---|---|---|---|
| C | 27.24322 | 6.863348 | 11.26808 | C | 18.32014 | 15.12143 | 11.19668 | C | 13.39682 | 9.051265 | 11.38786 |
| C | 5.952151 | 7.551417 | 11.31401 | C | 19.05812 | 16.28532 | 11.20529 | C | 12.15363 | 9.712545 | 11.39168 |
| C | 28.42962 | 8.953955 | 11.33939 | C | 21.15746 | 12.61187 | 11.24951 | C | 10.95693 | 9.011315 | 11.40526 |
| C | 27.22288 | 9.636429 | 11.32211 | C | 20.43649 | 11.38808 | 11.24701 | H | 6.356875 | 0.593880 | 11.18069 |
| C | 25.99008 | 8.957348 | 11.27916 | C | 18.98010 | 11.40208 | 11.24969 | H | 10.67817 | 0.621960 | 11.33193 |
| C | 26.03850 | 7.550442 | 11.25229 | C | 18.28123 | 12.63879 | 11.23118 | H | 17.21726 | 15.11616 | 11.17888 |
| C | 7.233858 | 6.828019 | 11.33576 | C | 21.12686 | 10.15314 | 11.24791 | H | 23.11831 | 13.56432 | 11.26521 |
| C | 7.269228 | 5.428016 | 11.31378 | C | 22.50476 | 10.18468 | 11.25288 | H | 20.55301 | 9.211218 | 11.24463 |
| C | 8.482188 | 4.727273 | 11.33601 | C | 23.21128 | 11.38555 | 11.26213 | H | 22.26904 | 15.06828 | 11.27026 |
| C | 9.676788 | 5.457203 | 11.37879 | C | 22.57187 | 12.60627 | 11.26029 | H | 18.82331 | 9.228512 | 11.29806 |
| C | 9.676639 | 6.858084 | 11.39601 | C | 16.86672 | 12.65933 | 11.24201 | H | 16.33846 | 13.62760 | 11.22834 |
| C | 8.446768 | 7.527767 | 11.37648 | C | 16.20448 | 11.45139 | 11.27921 | H | 10.65627 | 3.098184 | 11.37984 |
| C | 8.497748 | 3.255638 | 11.30873 | C | 16.89031 | 10.23842 | 11.29904 | H | 6.341778 | 3.068884 | 11.22898 |
| C | 7.301718 | 2.524094 | 11.25206 | C | 18.26703 | 10.18088 | 11.28129 | H | 6.319713 | 4.862563 | 11.27713 |
| C | 7.314525 | 1.138050 | 11.22493 | O | 23.44102 | 9.084995 | 11.25288 | H | 8.432058 | 8.633483 | 11.38956 |
| C | 8.518715 | 0.408700 | 11.25208 | B | 24.69510 | 9.705273 | 11.26616 | H | 10.64156 | 4.917077 | 11.39396 |
| C | 9.712585 | 1.153370 | 11.30821 | O | 24.62605 | 11.10286 | 11.27178 | H | 12.18582 | 5.834665 | 11.43254 |
| C | 9.704659 | 2.540254 | 11.33578 | O | 18.59744 | 17.65298 | 11.19788 | H | 14.32168 | 7.087914 | 11.40999 |
| B | 19.77364 | 18.41036 | 11.23053 | O | 14.78404 | 11.19266 | 11.31146 | H | 12.12996 | 10.81479 | 11.37924 |
| O | 20.93669 | 17.63286 | 11.25033 | B | 14.69254 | 9.797026 | 11.35133 | H | 9.997209 | 9.556862 | 11.40341 |
| C | 20.45127 | 16.27273 | 11.23464 | O | 15.93663 | 9.156354 | 11.34461 | H | 29.38108 | 9.512935 | 11.37364 |
| C | 21.16644 | 15.09475 | 11.24881 | C | 10.94295 | 7.608115 | 11.41376 | H | 27.22882 | 10.73869 | 11.34406 |
| C | 20.44156 | 13.87995 | 11.23849 | C | 12.17601 | 6.938528 | 11.42169 | H | 25.09569 | 6.980115 | 11.21701 |
| C | 19.02130 | 13.89307 | 11.21739 | C | 13.36950 | 7.643658 | 11.40891 | H | 4.736369 | 5.759680 | 11.24473 |

**c** 0.00000 0.00000 6.81422 serrated armchair

# COF-6

## Layer I AA, AB, inclined zigzag & armchair, and serrated zigzag & armchair

| | | | | | | | |
|---|---|---|---|---|---|---|---|
| C | 15.89563 | 8.845316 | 0.86161 | O | 9.288773 | 5.341016 | 0.82716 |
| C | 14.48142 | 8.839945 | 0.84999 | B | 8.040427 | 5.971807 | 0.82076 |
| C | 13.76977 | 7.610588 | 0.84166 | O | 8.120573 | 7.368169 | 0.81091 |
| C | 14.46919 | 6.381114 | 0.84331 | O | 16.79032 | 5.331043 | 0.84878 |
| C | 15.84657 | 6.423560 | 0.85196 | B | 18.03967 | 5.960022 | 0.85468 |
| C | 16.54457 | 7.629647 | 0.86141 | O | 17.96170 | 7.356371 | 0.86463 |
| C | 12.31386 | 7.612648 | 0.82996 | C | 5.521790 | 3.085190 | 0.82963 |
| C | 11.60519 | 8.843752 | 0.81941 | C | 4.320793 | 3.811040 | 0.84141 |
| C | 12.33496 | 10.10368 | 0.82261 | C | 4.283533 | 5.213800 | 0.84601 |
| C | 13.75529 | 10.10189 | 0.84169 | C | 5.512527 | 5.891126 | 0.83848 |
| C | 14.47044 | 11.32227 | 0.85003 | C | 6.745853 | 5.221990 | 0.82658 |
| C | 13.74534 | 12.49412 | 0.83789 | C | 6.717893 | 3.818930 | 0.82261 |

| | | | | | | | |
|---|---|---|---|---|---|---|---|
| C | 12.35196 | 12.49558 | 0.81744 | H | 16.43449 | 9.807772 | 0.86739 |
| C | 11.62342 | 11.32585 | 0.80941 | H | 12.17483 | 5.437313 | 0.84011 |
| C | 10.19080 | 8.853007 | 0.80986 | H | 9.654883 | 9.817066 | 0.80283 |
| C | 9.538543 | 7.639046 | 0.81301 | H | 13.90248 | 5.434823 | 0.83509 |
| C | 10.23402 | 6.431499 | 0.82308 | H | 10.52053 | 11.31154 | 0.79376 |
| C | 11.61116 | 6.385260 | 0.83086 | H | 15.57334 | 11.30425 | 0.86594 |
| O | 4.353760 | 0.823440 | 0.80971 | H | 5.508854 | 6.994033 | 0.84209 |
| B | 5.523970 | 1.589210 | 0.82696 | H | 3.367370 | 3.256520 | 0.84836 |
| O | 6.693240 | 0.821640 | 0.84359 | H | 7.675120 | 3.271100 | 0.81209 |

Transformation Matrix

| | | | | |
|---|---|---|---|---|
| **a** | 15.052800 | 0.000000 | 0.000000 | |
| **b** | 7.526400 | 13.036110 | 0.000000 | |
| **c** | 0.000000 | 0.000000 | 3.480887 | AA |
| **c** | 1.198595 | 0.692009 | 3.396059 | inclined zigzag |
| **c** | 1.387745 | 0.000000 | 3.402900 | inclined armchair |

## Layer II AB stacking

| | | | | | | | |
|---|---|---|---|---|---|---|---|
| C | 23.42203 | 13.19069 | 4.13016 | O | 16.81517 | 9.686385 | 4.09571 |
| C | 22.00782 | 13.18531 | 4.11854 | B | 15.56683 | 10.31718 | 4.08931 |
| C | 21.29617 | 11.95596 | 4.11021 | O | 15.64697 | 11.71354 | 4.07946 |
| C | 21.99559 | 10.72648 | 4.11186 | O | 24.31672 | 9.676413 | 4.11733 |
| C | 23.37297 | 10.76893 | 4.12051 | B | 25.56607 | 10.30539 | 4.12323 |
| C | 24.07097 | 11.97502 | 4.12996 | O | 25.48810 | 11.70174 | 4.13318 |
| C | 19.84026 | 11.95802 | 4.09851 | C | 13.04819 | 7.430559 | 4.09818 |
| C | 19.13159 | 13.18912 | 4.08796 | C | 11.84719 | 8.156409 | 4.10996 |
| C | 19.86136 | 14.44905 | 4.09116 | C | 11.80993 | 9.559170 | 4.11456 |
| C | 21.28169 | 14.44726 | 4.11024 | C | 13.03893 | 10.23650 | 4.10703 |
| C | 21.99684 | 15.66764 | 4.11858 | C | 14.27225 | 9.567360 | 4.09513 |
| C | 21.27174 | 16.83949 | 4.10644 | C | 14.24429 | 8.164299 | 4.09116 |
| C | 19.87836 | 16.84095 | 4.08599 | H | 23.96089 | 14.15314 | 4.13594 |
| C | 19.14982 | 15.67122 | 4.07796 | H | 19.70123 | 9.782682 | 4.10866 |
| C | 17.71720 | 13.19838 | 4.07841 | H | 17.18128 | 14.16244 | 4.07138 |
| C | 17.06494 | 11.98442 | 4.08156 | H | 21.42888 | 9.780192 | 4.10364 |
| C | 17.76042 | 10.77687 | 4.09163 | H | 18.04693 | 15.65691 | 4.06231 |
| C | 19.13756 | 10.73063 | 4.09941 | H | 23.09974 | 15.64962 | 4.13449 |
| O | 11.88016 | 5.168809 | 4.07826 | H | 13.03525 | 11.33940 | 4.11064 |
| B | 13.05037 | 5.934579 | 4.09551 | H | 10.89377 | 7.601890 | 4.11691 |
| O | 14.21964 | 5.167009 | 4.11214 | H | 15.20152 | 7.616469 | 4.08064 |
| **c** | 0.00000 | 0.00000 | 6.537105 | AB | | | |

## Layer II serrated zigzag

| | | | | | | | |
|---|---|---|---|---|---|---|---|
| C | 17.094220 | 9.537325 | 4.27655 | O | 10.487370 | 6.033025 | 4.24210 |
| C | 15.680010 | 9.531954 | 4.26493 | B | 9.239022 | 6.663816 | 4.23570 |
| C | 14.968360 | 8.302597 | 4.25660 | O | 9.319168 | 8.060178 | 4.22585 |
| C | 15.667780 | 7.073123 | 4.25825 | O | 17.988920 | 6.023052 | 4.26372 |
| C | 17.045160 | 7.115569 | 4.26690 | B | 4.185460 | 6.652031 | 4.26962 |
| C | 17.743170 | 8.321655 | 4.27635 | O | 19.160290 | 8.048380 | 4.27957 |
| C | 13.512450 | 8.304657 | 4.24490 | C | 6.720385 | 3.777199 | 4.24457 |
| C | 12.803790 | 9.535761 | 4.23435 | C | 5.519388 | 4.503049 | 4.25635 |
| C | 13.533560 | 10.795690 | 4.23755 | C | 5.482128 | 5.905809 | 4.26095 |
| C | 14.953890 | 10.793900 | 4.25663 | C | 6.711122 | 6.583135 | 4.25342 |
| C | 15.669030 | 12.014280 | 4.26497 | C | 7.944448 | 5.913999 | 4.24152 |
| C | 7.417540 | 0.150020 | 4.25283 | C | 7.916488 | 4.510939 | 4.23755 |
| C | 6.024160 | 0.151480 | 4.23238 | H | 17.633090 | 10.499780 | 4.28233 |
| C | 12.822010 | 12.017860 | 4.22435 | H | 13.373430 | 6.129322 | 4.25505 |
| C | 11.389390 | 9.545016 | 4.22480 | H | 10.853480 | 10.509080 | 4.21777 |

| | | | | | | | |
|---|---|---|---|---|---|---|---|
| C | 10.737140 | 8.331055 | 4.22795 | H | 15.101080 | 6.126832 | 4.25003 |
| C | 11.432620 | 7.123508 | 4.23802 | H | 11.719120 | 12.003550 | 4.20870 |
| C | 12.809760 | 7.077269 | 4.24580 | H | 16.771930 | 11.996260 | 4.28088 |
| O | 5.552355 | 1.515449 | 4.22465 | H | 6.707449 | 7.686042 | 4.25703 |
| B | 6.722565 | 2.281219 | 4.24190 | H | 4.565965 | 3.948529 | 4.26330 |
| O | 7.891835 | 1.513649 | 4.25853 | H | 8.873714 | 3.963109 | 4.22703 |
| **c** | 0.00000 | 0.00000 | 6.82988 | serrated zigzag | | | |

## Layer II serrated armchair

| | | | | | | | |
|---|---|---|---|---|---|---|---|
| C | 17.283370 | 8.845316 | 4.27996 | O | 10.676520 | 5.341016 | 4.24551 |
| C | 15.869160 | 8.839945 | 4.26834 | B | 9.428172 | 5.971807 | 4.23911 |
| C | 15.157510 | 7.610588 | 4.26001 | O | 9.508318 | 7.368169 | 4.22926 |
| C | 15.856930 | 6.381114 | 4.26166 | O | 3.125270 | 5.331043 | 4.26713 |
| C | 17.234310 | 6.423560 | 4.27031 | B | 4.374610 | 5.960022 | 4.27303 |
| C | 17.932320 | 7.629647 | 4.27976 | O | 4.296640 | 7.356371 | 4.28298 |
| C | 13.701600 | 7.612648 | 4.24831 | C | 6.909535 | 3.085190 | 4.24798 |
| C | 12.992940 | 8.843752 | 4.23776 | C | 5.708538 | 3.811040 | 4.25976 |
| C | 13.722700 | 10.103680 | 4.24096 | C | 5.671278 | 5.213800 | 4.26436 |
| C | 15.143030 | 10.101890 | 4.26004 | C | 6.900272 | 5.891126 | 4.25683 |
| C | 15.858180 | 11.322270 | 4.26838 | C | 8.133598 | 5.221990 | 4.24493 |
| C | 15.133090 | 12.494120 | 4.25624 | C | 8.105639 | 3.818930 | 4.24096 |
| C | 13.739710 | 12.495580 | 4.23579 | H | 17.822240 | 9.807772 | 4.28574 |
| C | 13.011160 | 11.325850 | 4.22776 | H | 13.562580 | 5.437313 | 4.25846 |
| C | 11.578540 | 8.853007 | 4.22821 | H | 11.042630 | 9.817066 | 4.22118 |
| C | 10.926290 | 7.639046 | 4.23136 | H | 15.290230 | 5.434823 | 4.25344 |
| C | 11.621770 | 6.431499 | 4.24143 | H | 11.908270 | 11.311540 | 4.21211 |
| C | 12.998910 | 6.385260 | 4.24921 | H | 16.961090 | 11.304250 | 4.28429 |
| O | 5.741505 | 0.823440 | 4.22806 | H | 6.896599 | 6.994033 | 4.26044 |
| B | 6.911715 | 1.589210 | 4.24531 | H | 4.755115 | 3.256520 | 4.26671 |
| O | 8.080985 | 0.821640 | 4.26194 | H | 9.062865 | 3.271100 | 4.23044 |
| **c** | 0.00000 | 0.00000 | 6.83669 | serrated armchair | | | |

# TP COF

## Layer I AA, AB, inclined zigzag & armchair, and serrated zigzag & armchair

| | | | | | | | | | | | |
|---|---|---|---|---|---|---|---|---|---|---|---|
| C | 34.39868 | 29.87646 | 0.8851000 | C | 24.37890 | 16.82335 | 1.032600 | B | 11.84997 | 13.94665 | 0.9876250 |
| C | 36.83124 | 31.28005 | 0.9077250 | C | 25.61187 | 16.11050 | 1.034250 | C | 16.85226 | 0.9500470 | 0.8756500 |
| C | 36.80484 | 29.87837 | 0.9170500 | C | 25.60210 | 14.68645 | 1.042625 | C | 18.08207 | 1.668191 | 0.8882250 |
| C | 35.60217 | 29.15006 | 0.9062250 | C | 24.37281 | 14.01387 | 1.039150 | C | 18.05348 | 3.069575 | 0.8809500 |
| H | 33.44363 | 29.32625 | 0.8758000 | C | 23.14563 | 14.69843 | 1.027075 | C | 16.84923 | 3.794051 | 0.8623500 |
| C | 36.31152 | 23.12550 | 0.9099250 | H | 24.36958 | 12.91163 | 1.040475 | C | 15.64649 | 3.067074 | 0.8480500 |
| C | 37.02438 | 24.34742 | 0.9158250 | H | 22.22370 | 16.65756 | 1.022275 | C | 15.62094 | 1.665626 | 0.8540750 |
| C | 38.45328 | 21.86251 | 0.9374750 | C | 28.04238 | 14.66603 | 1.033025 | H | 19.00718 | 3.622121 | 0.8918250 |
| C | 36.29857 | 25.51970 | 0.9156250 | C | 26.86112 | 13.99045 | 1.046875 | H | 14.69127 | 3.617120 | 0.8324500 |
| C | 34.89022 | 23.12622 | 0.9052000 | H | 26.85656 | 12.88845 | 1.058825 | C | 19.31136 | 0.9209210 | 0.9099000 |
| C | 34.16021 | 21.86543 | 0.9001500 | H | 28.99457 | 14.11092 | 1.033850 | H | 20.26561 | 1.472396 | 0.9195500 |
| C | 34.87082 | 20.63552 | 0.8890250 | H | 37.76038 | 29.32940 | 0.9335250 | H | 20.24120 | 10.10662 | 0.9359750 |
| C | 36.32595 | 20.63432 | 0.8965750 | H | 38.99637 | 22.82239 | 0.9526250 | H | 19.37732 | 8.607212 | 0.8973250 |
| C | 37.03899 | 21.86257 | 0.9123750 | H | 38.12735 | 24.33161 | 0.9198250 | H | 14.32416 | 8.609713 | 0.8674250 |
| C | 34.17387 | 19.40526 | 0.8849000 | H | 36.45034 | 18.45755 | 0.8907000 | H | 13.46097 | 10.10351 | 0.9084250 |
| C | 37.02020 | 19.40224 | 0.9024250 | H | 34.74218 | 18.45969 | 0.8769500 | H | 17.71272 | 14.48212 | 0.9219500 |
| C | 32.79719 | 19.44526 | 0.8987000 | H | 33.07544 | 24.33291 | 0.9043250 | H | 15.98495 | 14.48153 | 0.9239750 |
| C | 32.74545 | 21.86793 | 0.9114750 | H | 32.20335 | 22.82837 | 0.9240000 | C | 41.87809 | 18.21613 | 1.031250 |
| C | 34.17826 | 24.34885 | 0.9069750 | C | 14.39298 | 0.9160510 | 0.8412250 | C | 43.10454 | 18.90112 | 1.055425 |
| C | 32.09827 | 20.65075 | 0.9128750 | H | 13.43777 | 1.465609 | 0.8237000 | C | 44.33380 | 18.22957 | 1.074325 |
| C | 34.90507 | 25.52051 | 0.9113000 | C | 17.57832 | 12.30619 | 0.9074750 | C | 44.34391 | 16.80553 | 1.069575 |

| | | | | | | | | | | | |
|---|---|---|---|---|---|---|---|---|---|---|---|
| O | 30.68187 | 20.37381 | 0.9355250 | C | 18.27864 | 13.53516 | 0.9240000 | C | 43.11116 | 16.09209 | 1.056575 |
| O | 31.85747 | 18.35059 | 0.9119000 | C | 19.65576 | 13.49148 | 0.9479500 | C | 41.90845 | 16.81111 | 1.037300 |
| O | 36.77170 | 26.88481 | 0.9179000 | C | 20.35256 | 12.28453 | 0.9534500 | H | 43.09948 | 20.00329 | 1.053900 |
| O | 34.43290 | 26.88552 | 0.9100750 | C | 19.70287 | 11.06943 | 0.9320000 | H | 40.95639 | 16.25577 | 1.021600 |
| B | 30.60740 | 18.97701 | 0.9375750 | O | 20.60011 | 14.58300 | 0.9765000 | C | 39.09807 | 20.64413 | 0.9491000 |
| B | 35.60246 | 27.65364 | 0.9126750 | O | 21.76957 | 12.55647 | 0.9865500 | C | 38.39683 | 19.43987 | 0.9309250 |
| C | 38.05931 | 32.03014 | 0.9193000 | C | 16.12204 | 12.30593 | 0.9025000 | O | 40.51356 | 20.36410 | 0.9872000 |
| H | 39.01498 | 31.48159 | 0.9364750 | C | 15.41295 | 11.07515 | 0.8962750 | O | 39.33394 | 18.34341 | 0.9565000 |
| C | 24.41733 | 18.26152 | 1.027250 | C | 16.14113 | 9.814229 | 0.8848000 | B | 40.58493 | 18.96701 | 0.9929000 |
| C | 34.37049 | 31.27794 | 0.8740250 | C | 17.56149 | 9.813969 | 0.8926750 | C | 45.59208 | 18.92632 | 1.090825 |
| C | 35.60035 | 31.99605 | 0.8855750 | C | 18.28873 | 11.07605 | 0.9091000 | C | 45.57991 | 16.09949 | 1.068700 |
| C | 33.14102 | 32.02530 | 0.8509750 | C | 15.42718 | 8.593088 | 0.8720250 | C | 43.15001 | 14.65392 | 1.056175 |
| C | 26.84758 | 16.81718 | 1.020575 | C | 16.15344 | 7.421854 | 0.8680250 | H | 42.19825 | 14.09806 | 1.053025 |
| C | 28.08053 | 16.10404 | 1.014725 | C | 17.54702 | 7.421431 | 0.8769250 | C | 46.81231 | 16.81342 | 1.072000 |
| C | 29.28315 | 16.82283 | 0.9879500 | C | 18.27422 | 8.592146 | 0.8890250 | C | 46.77369 | 18.25139 | 1.089550 |
| C | 29.31319 | 18.22769 | 0.9686000 | O | 15.67979 | 6.058103 | 0.8582000 | H | 47.72544 | 18.80706 | 1.098950 |
| C | 28.08663 | 18.91288 | 0.9813000 | O | 18.01930 | 6.056999 | 0.8738000 | H | 45.58649 | 20.02830 | 1.101200 |
| C | 26.85737 | 18.24126 | 1.006300 | C | 13.99864 | 11.06667 | 0.9098500 | C | 45.59021 | 14.67545 | 1.055400 |
| H | 30.23537 | 16.26740 | 0.9804000 | C | 13.34772 | 12.28106 | 0.9324750 | C | 44.33161 | 13.97873 | 1.055775 |
| H | 28.09240 | 20.01511 | 0.9679500 | C | 14.04313 | 13.48859 | 0.9356250 | H | 44.33682 | 12.87656 | 1.052275 |
| C | 25.59852 | 18.93723 | 1.014650 | C | 15.42030 | 13.53392 | 0.9186500 | C | 9.326377 | 14.00396 | 1.035200 |
| H | 25.60300 | 20.03930 | 1.008300 | O | 11.93065 | 12.55056 | 0.9637250 | C | 10.55260 | 14.69006 | 1.027125 |
| H | 23.46556 | 18.81709 | 1.031025 | O | 13.09771 | 14.57885 | 0.9686750 | C | 10.52161 | 16.09469 | 1.050875 |
| H | 32.18653 | 31.47438 | 0.8413000 | B | 21.84881 | 13.95298 | 0.9997500 | H | 9.331083 | 12.90176 | 1.019800 |
| C | 23.17641 | 16.10342 | 1.029025 | B | 16.84910 | 5.290247 | 0.8633000 | H | 11.47402 | 16.64954 | 1.048075 |

Transformation Matrix

| | | | | |
|---|---|---|---|---|
| **a** | **3**7.493430 | 0.000000 | 0.000000 | |
| **b** | 18.746710 | 32.470250 | 0.000000 | |
| **c** | 0.000000 | 0.000000 | 3.472000 | AA |
| **c** | 1.203185 | 0.69466 | 3.42000 | inclined zigzag |
| **c** | 1.386345 | 0.000000 | 3.425000 | inclined armchair |

## Layer II AB stacking

| | | | | | | | | | | | |
|---|---|---|---|---|---|---|---|---|---|---|---|
| C | 53.14540 | 40.69988 | 4.086278 | C | 43.12562 | 27.64677 | 4.233778 | B | 30.59669 | 24.77007 | 4.188803 |
| C | 55.57796 | 42.10347 | 4.108903 | C | 44.35859 | 26.93392 | 4.235428 | C | 35.59898 | 11.77347 | 4.076828 |
| C | 55.55156 | 40.70179 | 4.118228 | C | 44.34882 | 25.50987 | 4.243803 | C | 36.82878 | 12.49161 | 4.089403 |
| C | 54.34888 | 39.97348 | 4.107403 | C | 43.11953 | 24.83729 | 4.240328 | C | 36.80019 | 13.89300 | 4.082128 |
| H | 52.19035 | 40.14967 | 4.076978 | C | 41.89235 | 25.52185 | 4.228253 | C | 35.59595 | 14.61747 | 4.063528 |
| C | 55.05824 | 33.94892 | 4.111103 | H | 43.11629 | 23.73505 | 4.241653 | C | 34.39320 | 13.89050 | 4.049228 |
| C | 55.77110 | 35.17084 | 4.117003 | H | 40.97041 | 27.48098 | 4.223453 | C | 34.36766 | 12.48905 | 4.055253 |
| C | 57.20000 | 32.68593 | 4.138653 | C | 46.78909 | 25.48945 | 4.234203 | H | 37.75389 | 14.44554 | 4.093003 |
| C | 55.04528 | 36.34312 | 4.116803 | C | 45.60783 | 24.81387 | 4.248053 | H | 33.43798 | 14.44054 | 4.033628 |
| C | 53.63694 | 33.94964 | 4.106378 | H | 45.60328 | 23.71187 | 4.260003 | C | 38.05807 | 11.74434 | 4.111078 |
| C | 52.90693 | 32.68885 | 4.101328 | H | 47.74129 | 24.93434 | 4.235028 | H | 39.01233 | 12.29582 | 4.120728 |
| C | 53.61753 | 31.45894 | 4.090203 | H | 56.50710 | 40.15282 | 4.134703 | H | 38.98792 | 20.93004 | 4.137153 |
| C | 55.07267 | 31.45774 | 4.097753 | H | 57.74308 | 33.64581 | 4.153803 | H | 38.12403 | 19.43063 | 4.098503 |
| C | 55.78571 | 32.68599 | 4.113553 | H | 56.87407 | 35.15503 | 4.121003 | H | 33.07088 | 19.43313 | 4.068603 |
| C | 52.92059 | 30.22868 | 4.086078 | H | 55.19706 | 29.28097 | 4.091878 | H | 32.20769 | 20.92693 | 4.109603 |
| C | 55.76691 | 30.22566 | 4.103603 | H | 53.48890 | 29.28311 | 4.078128 | H | 36.45943 | 25.30554 | 4.123128 |
| C | 51.54391 | 30.26868 | 4.099878 | H | 51.82215 | 35.15633 | 4.105503 | H | 34.73167 | 25.30495 | 4.125153 |
| C | 51.49216 | 32.69135 | 4.112653 | H | 50.95007 | 33.65179 | 4.125178 | C | 60.62481 | 29.03955 | 4.232428 |
| C | 52.92498 | 35.17227 | 4.108153 | C | 33.13969 | 11.73947 | 4.042403 | C | 61.85126 | 29.72454 | 4.256603 |
| C | 50.84499 | 31.47417 | 4.114053 | H | 32.18449 | 12.28903 | 4.024878 | C | 63.08052 | 29.05299 | 4.275503 |
| C | 53.65179 | 36.34393 | 4.112478 | C | 36.32504 | 23.12961 | 4.108653 | C | 63.09063 | 27.62895 | 4.270753 |
| O | 49.42859 | 31.19723 | 4.136703 | C | 37.02536 | 24.35858 | 4.125178 | C | 61.85788 | 26.91551 | 4.257753 |
| O | 50.60419 | 29.17401 | 4.113078 | C | 38.40247 | 24.31490 | 4.149128 | C | 60.65517 | 27.63453 | 4.238478 |
| O | 55.51842 | 37.70823 | 4.119078 | C | 39.09927 | 23.10795 | 4.154628 | H | 61.84620 | 30.82671 | 4.255078 |
| O | 53.17962 | 37.70894 | 4.111253 | C | 38.44958 | 21.89285 | 4.133178 | H | 59.70311 | 27.07919 | 4.222778 |
| B | 49.35412 | 29.80043 | 4.138753 | O | 39.34682 | 25.40642 | 4.177678 | C | 57.84478 | 31.46755 | 4.150278 |
| B | 54.34917 | 38.47706 | 4.113853 | O | 40.51628 | 23.37989 | 4.187728 | C | 57.14355 | 30.26329 | 4.132103 |
| C | 56.80603 | 42.85356 | 4.120478 | C | 34.86876 | 23.12935 | 4.103678 | O | 59.26028 | 31.18752 | 4.188378 |

| | | | | | | | | | | | |
|---|---|---|---|---|---|---|---|---|---|---|---|
| H | 57.76170 | 42.30501 | 4.137653 | C | 34.15966 | 21.89857 | 4.097453 | O | 58.08065 | 29.16683 | 4.157678 |
| C | 43.16405 | 29.08494 | 4.228428 | C | 34.88785 | 20.63765 | 4.085978 | B | 59.33165 | 29.79043 | 4.194078 |
| C | 53.11721 | 42.10136 | 4.075203 | C | 36.30820 | 20.63739 | 4.093853 | C | 64.33879 | 29.74974 | 4.292003 |
| C | 54.34706 | 42.81947 | 4.086753 | C | 37.03545 | 21.89947 | 4.110278 | C | 64.32663 | 26.92291 | 4.269878 |
| C | 51.88774 | 42.84872 | 4.052153 | C | 34.17390 | 19.41651 | 4.073203 | C | 61.89672 | 25.47734 | 4.257353 |
| C | 45.59430 | 27.64060 | 4.221753 | C | 34.90015 | 18.24528 | 4.069203 | H | 60.94497 | 24.92148 | 4.254203 |
| C | 46.82725 | 26.92746 | 4.215903 | C | 36.29374 | 18.24485 | 4.078103 | C | 65.55902 | 27.63684 | 4.273178 |
| C | 48.02987 | 27.64625 | 4.189128 | C | 37.02094 | 19.41557 | 4.090203 | C | 65.52040 | 29.07481 | 4.290728 |
| C | 48.05991 | 29.05111 | 4.169778 | O | 34.42651 | 16.88153 | 4.059378 | H | 66.47215 | 29.63048 | 4.300128 |
| C | 46.83334 | 29.73630 | 4.182478 | O | 36.76601 | 16.88042 | 4.074978 | H | 64.33321 | 30.85172 | 4.302378 |
| C | 45.60409 | 29.06468 | 4.207478 | C | 32.74535 | 21.89009 | 4.111028 | C | 64.33693 | 25.49887 | 4.256578 |
| H | 48.98209 | 27.09082 | 4.181578 | C | 32.09444 | 23.10448 | 4.133653 | C | 63.07833 | 24.80215 | 4.256953 |
| H | 46.83912 | 30.83853 | 4.169128 | C | 32.78984 | 24.31201 | 4.136803 | H | 63.08353 | 23.69998 | 4.253453 |
| C | 44.34524 | 29.76065 | 4.215828 | C | 34.16702 | 24.35734 | 4.119828 | C | 28.07309 | 24.82738 | 4.236378 |
| H | 44.34972 | 30.86272 | 4.209478 | O | 30.67736 | 23.37398 | 4.164903 | C | 29.29932 | 25.51348 | 4.228303 |
| H | 42.21228 | 29.64051 | 4.232203 | O | 31.84443 | 25.40227 | 4.169853 | C | 29.26833 | 26.91811 | 4.252053 |
| H | 50.93325 | 42.29780 | 4.042478 | B | 40.59553 | 24.77640 | 4.200928 | H | 28.07780 | 23.72518 | 4.220978 |
| C | 41.92313 | 26.92684 | 4.230203 | B | 35.59582 | 16.11367 | 4.064478 | H | 30.22074 | 27.47296 | 4.249253 |

**c** 0.00000 0.00000 6.402355 AB

## Layer II serrated zigzag

| | | | | | | | | | | | |
|---|---|---|---|---|---|---|---|---|---|---|---|
| C | 35.60187 | 30.57112 | 4.297788 | C | 25.58208 | 17.51801 | 4.445288 | B | 13.05315 | 14.64131 | 4.400313 |
| C | 38.03443 | 31.97471 | 4.320413 | C | 26.81505 | 16.80516 | 4.446938 | C | 18.05544 | 1.64470 | 4.288338 |
| C | 38.00803 | 30.57303 | 4.329738 | C | 26.80528 | 15.38111 | 4.455313 | C | 19.28525 | 2.36285 | 4.300913 |
| C | 36.80536 | 29.84472 | 4.318913 | C | 25.57599 | 14.70853 | 4.451838 | C | 19.25666 | 3.76423 | 4.293638 |
| H | 34.64682 | 30.02091 | 4.288488 | C | 24.34881 | 15.39309 | 4.439763 | C | 18.05241 | 4.48871 | 4.275038 |
| C | 37.51471 | 23.82016 | 4.322613 | H | 25.57276 | 13.60629 | 4.453163 | C | 16.84967 | 3.76173 | 4.260738 |
| C | 38.22757 | 25.04208 | 4.328513 | H | 23.42688 | 17.35222 | 4.434963 | C | 16.82413 | 2.36028 | 4.266763 |
| C | 39.65647 | 22.55717 | 4.350163 | C | 29.24556 | 15.36069 | 4.445713 | H | 20.21036 | 4.31678 | 4.304513 |
| C | 37.50175 | 26.21436 | 4.328313 | C | 28.06430 | 14.68511 | 4.459563 | H | 15.89445 | 4.31178 | 4.245138 |
| C | 36.09341 | 23.82088 | 4.317888 | H | 28.05974 | 13.58311 | 4.471513 | C | 20.51454 | 1.61558 | 4.322588 |
| C | 35.36340 | 22.56009 | 4.312838 | H | 30.19775 | 14.80558 | 4.446538 | H | 21.46879 | 2.16705 | 4.332238 |
| C | 36.07401 | 21.33018 | 4.301713 | H | 38.96357 | 30.02406 | 4.346213 | H | 21.44438 | 10.80128 | 4.348663 |
| C | 37.52914 | 21.32898 | 4.309263 | H | 40.19955 | 23.51705 | 4.365313 | H | 20.58050 | 9.30187 | 4.310013 |
| C | 38.24218 | 22.55723 | 4.325063 | H | 39.33054 | 25.02627 | 4.332513 | H | 15.52734 | 9.30437 | 4.280113 |
| C | 35.37706 | 20.09992 | 4.297588 | H | 37.65353 | 19.15221 | 4.303388 | H | 14.66416 | 10.79817 | 4.321113 |
| C | 38.22338 | 20.09690 | 4.315113 | H | 35.94537 | 19.15435 | 4.289638 | H | 18.91590 | 15.17678 | 4.334638 |
| C | 34.00038 | 20.13992 | 4.311388 | H | 34.27863 | 25.02757 | 4.317013 | H | 17.18814 | 15.17619 | 4.336663 |
| C | 33.94864 | 22.56259 | 4.324163 | H | 33.40654 | 23.52303 | 4.336688 | C | 43.08128 | 18.91079 | 4.443938 |
| C | 35.38145 | 25.04351 | 4.319663 | C | 15.59616 | 1.61071 | 4.253913 | C | 44.30773 | 19.59578 | 4.468113 |
| C | 33.30146 | 21.34541 | 4.325563 | H | 14.64095 | 2.16026 | 4.236388 | C | 45.53699 | 18.92423 | 4.487013 |
| C | 36.10826 | 26.21517 | 4.323988 | C | 18.78150 | 13.00085 | 4.320163 | C | 45.54710 | 17.50019 | 4.482263 |
| O | 31.88505 | 21.06847 | 4.348213 | C | 19.48182 | 14.22982 | 4.336688 | C | 44.31435 | 16.78675 | 4.469263 |
| O | 33.06065 | 19.04525 | 4.324588 | C | 20.85894 | 14.18614 | 4.360638 | C | 43.11164 | 17.50577 | 4.449988 |
| O | 37.97489 | 27.57947 | 4.330588 | C | 21.55574 | 12.97919 | 4.366138 | H | 44.30267 | 20.69795 | 4.466588 |
| O | 35.63609 | 27.58018 | 4.322763 | C | 20.90605 | 11.76409 | 4.344688 | H | 42.15958 | 16.95043 | 4.434288 |
| B | 31.81059 | 19.67167 | 4.350263 | O | 21.80329 | 15.27766 | 4.389188 | C | 40.30125 | 21.33879 | 4.361788 |
| B | 36.80564 | 28.34830 | 4.325363 | O | 22.97275 | 13.25113 | 4.399238 | C | 39.60002 | 20.13453 | 4.343613 |
| C | 20.51579 | 0.25455 | 4.331988 | C | 17.32522 | 13.00059 | 4.315188 | O | 41.71675 | 21.05876 | 4.399888 |
| H | 40.21817 | 32.17625 | 4.349163 | C | 16.61613 | 11.76981 | 4.308963 | O | 40.53712 | 19.03807 | 4.369188 |
| C | 25.62051 | 18.95618 | 4.439938 | C | 17.34431 | 10.50889 | 4.297488 | B | 41.78812 | 19.66167 | 4.405588 |
| C | 35.57368 | 31.97260 | 4.286713 | C | 18.76467 | 10.50863 | 4.305363 | C | 46.79527 | 19.62098 | 4.503513 |
| C | 18.05683 | 0.22046 | 4.298263 | C | 19.49191 | 11.77071 | 4.321788 | C | 46.78310 | 16.79415 | 4.481388 |
| C | 15.59750 | 0.24971 | 4.263663 | C | 16.63037 | 9.28774 | 4.284713 | C | 44.35320 | 15.34858 | 4.468863 |
| C | 28.05076 | 17.51184 | 4.433263 | C | 17.35662 | 8.11651 | 4.280713 | H | 43.40144 | 14.79272 | 4.465713 |
| C | 29.28371 | 16.79870 | 4.427413 | C | 18.75020 | 8.11609 | 4.289613 | C | 10.52207 | 17.50808 | 4.484688 |
| C | 30.48633 | 17.51749 | 4.400638 | C | 19.47740 | 9.28680 | 4.301713 | C | 47.97688 | 18.94605 | 4.502238 |
| C | 30.51637 | 18.92235 | 4.381288 | O | 16.88297 | 6.75276 | 4.270888 | H | 11.43520 | 19.50172 | 4.511638 |
| C | 29.28981 | 19.60754 | 4.393988 | O | 19.22248 | 6.75165 | 4.286488 | H | 46.78968 | 20.72296 | 4.513888 |
| C | 28.06055 | 18.93592 | 4.418988 | C | 15.20183 | 11.76133 | 4.322538 | C | 9.29997 | 15.37011 | 4.468088 |
| H | 31.43855 | 16.96206 | 4.393088 | C | 14.55091 | 12.97572 | 4.345163 | C | 45.53480 | 14.67339 | 4.468463 |
| H | 29.29558 | 20.70977 | 4.380638 | C | 15.24632 | 14.18325 | 4.348313 | H | 8.04657 | 13.57122 | 4.464963 |

| | | | | | | | | | | | |
|---|---|---|---|---|---|---|---|---|---|---|---|
| C | 26.80170 | 19.63189 | 4.427338 | C | 16.62348 | 14.22858 | 4.331338 | C | 10.52956 | 14.69862 | 4.447888 |
| H | 26.80618 | 20.73396 | 4.420988 | O | 13.13383 | 13.24522 | 4.376413 | C | 11.75578 | 15.38472 | 4.439813 |
| H | 24.66875 | 19.51175 | 4.443713 | O | 14.30089 | 15.27351 | 4.381363 | C | 11.72480 | 16.78935 | 4.463563 |
| H | 33.38972 | 32.16904 | 4.253988 | B | 23.05199 | 14.64764 | 4.412438 | H | 10.53427 | 13.59642 | 4.432488 |
| C | 24.37959 | 16.79808 | 4.441713 | B | 18.05228 | 5.98490 | 4.275988 | H | 12.67721 | 17.34420 | 4.460763 |

**c** 0.00000 0.00000 6.82538 serrated zigzag

## Layer II serrated armchair

| | | | | | | | | | | | |
|---|---|---|---|---|---|---|---|---|---|---|---|
| C | 35.78503 | 29.876460 | 4.301190 | C | 25.76525 | 16.823350 | 4.448690 | B | 13.23631 | 13.946650 | 4.403715 |
| C | 38.21759 | 31.280050 | 4.323815 | C | 26.99821 | 16.110500 | 4.450340 | C | 18.23861 | 0.950047 | 4.291740 |
| C | 38.19118 | 29.878370 | 4.333140 | C | 26.98845 | 14.686450 | 4.458715 | C | 19.46841 | 1.668191 | 4.304315 |
| C | 36.98851 | 29.150060 | 4.322315 | C | 25.75916 | 14.013870 | 4.455240 | C | 19.43983 | 3.069575 | 4.297040 |
| H | 34.82998 | 29.326250 | 4.291890 | C | 24.53197 | 14.698430 | 4.443165 | C | 18.23557 | 3.794051 | 4.278440 |
| C | 37.69786 | 23.125500 | 4.326015 | H | 25.75592 | 12.911630 | 4.456565 | C | 17.03284 | 3.067074 | 4.264140 |
| C | 38.41072 | 24.347420 | 4.331915 | H | 23.61004 | 16.657560 | 4.438365 | C | 17.00729 | 1.665626 | 4.270165 |
| C | 39.83963 | 21.862510 | 4.353565 | C | 29.42872 | 14.666030 | 4.449115 | H | 20.39352 | 3.622121 | 4.307915 |
| C | 37.68491 | 25.519700 | 4.331715 | C | 28.24747 | 13.990450 | 4.462965 | H | 16.07762 | 3.617120 | 4.248540 |
| C | 36.27657 | 23.126220 | 4.321290 | H | 28.24290 | 12.888450 | 4.474915 | C | 20.69770 | 0.920921 | 4.325990 |
| C | 35.54655 | 21.865430 | 4.316240 | H | 30.38091 | 14.110920 | 4.449940 | H | 21.65195 | 1.472396 | 4.335640 |
| C | 36.25716 | 20.635520 | 4.305115 | H | 39.14672 | 29.329400 | 4.349615 | H | 21.62754 | 10.106620 | 4.352065 |
| C | 37.71230 | 20.634320 | 4.312665 | H | 40.38271 | 22.822390 | 4.368715 | H | 20.76366 | 8.607212 | 4.313415 |
| C | 38.42533 | 21.862570 | 4.328465 | H | 39.51369 | 24.331610 | 4.335915 | H | 15.71050 | 8.609713 | 4.283515 |
| C | 35.56021 | 19.405260 | 4.300990 | H | 37.83669 | 18.457550 | 4.306790 | H | 14.84731 | 10.103510 | 4.324515 |
| C | 38.40654 | 19.402240 | 4.318515 | H | 36.12852 | 18.459690 | 4.293040 | H | 19.09907 | 14.482120 | 4.338040 |
| C | 34.18354 | 19.445260 | 4.314790 | H | 34.46178 | 24.332910 | 4.320415 | H | 17.37130 | 14.481530 | 4.340065 |
| C | 34.13179 | 21.867930 | 4.327565 | H | 33.58969 | 22.828370 | 4.340090 | C | 43.26443 | 18.216130 | 4.447340 |
| C | 35.56461 | 24.348850 | 4.323065 | C | 15.77932 | 0.916051 | 4.257315 | C | 44.49089 | 18.901120 | 4.471515 |
| C | 33.48462 | 20.650750 | 4.328965 | H | 14.82411 | 1.465609 | 4.239790 | C | 45.72015 | 18.229570 | 4.490415 |
| C | 36.29142 | 25.520510 | 4.327390 | C | 18.96466 | 12.306190 | 4.323565 | C | 45.73026 | 16.805530 | 4.485665 |
| O | 32.06821 | 20.373810 | 4.351615 | C | 19.66499 | 13.535160 | 4.340090 | C | 44.49751 | 16.092090 | 4.472665 |
| O | 33.24382 | 18.350590 | 4.327990 | C | 21.04210 | 13.491480 | 4.364040 | C | 43.29480 | 16.811110 | 4.453390 |
| O | 38.15805 | 26.884810 | 4.333990 | C | 21.73890 | 12.284530 | 4.369540 | H | 44.48582 | 20.003290 | 4.469990 |
| O | 35.81924 | 26.885520 | 4.326165 | C | 21.08921 | 11.069430 | 4.348090 | H | 42.34274 | 16.255770 | 4.437690 |
| B | 31.99375 | 18.977010 | 4.353665 | O | 21.98645 | 14.583000 | 4.392590 | C | 40.48441 | 20.644130 | 4.365190 |
| B | 36.98880 | 27.653640 | 4.328765 | O | 23.15591 | 12.556470 | 4.402640 | C | 39.78318 | 19.439870 | 4.347015 |
| C | 39.44566 | 32.030140 | 4.335390 | C | 17.50838 | 12.305930 | 4.318590 | O | 41.89991 | 20.364100 | 4.403290 |
| H | 40.40133 | 31.481590 | 4.352565 | C | 16.79930 | 11.075150 | 4.312365 | O | 40.72028 | 18.343410 | 4.372590 |
| C | 25.80367 | 18.261520 | 4.443340 | C | 17.52748 | 9.814229 | 4.300890 | B | 41.97128 | 18.967010 | 4.408990 |
| C | 35.75684 | 31.277940 | 4.290115 | C | 18.94783 | 9.813969 | 4.308765 | C | 46.97842 | 18.926320 | 4.506915 |
| C | 36.98669 | 31.996050 | 4.301665 | C | 19.67508 | 11.076050 | 4.325190 | C | 9.47283 | 16.099490 | 4.484790 |
| C | 34.52737 | 32.025300 | 4.267065 | C | 16.81353 | 8.593088 | 4.288115 | C | 44.53635 | 14.653920 | 4.472265 |
| C | 28.23392 | 16.817180 | 4.436665 | C | 17.53979 | 7.421854 | 4.284115 | H | 43.58459 | 14.098060 | 4.469115 |
| C | 29.46688 | 16.104040 | 4.430815 | C | 18.93336 | 7.421431 | 4.293015 | C | 10.70522 | 16.813420 | 4.488090 |
| C | 30.66949 | 16.822830 | 4.404040 | C | 19.66056 | 8.592146 | 4.305115 | C | 10.66660 | 18.251390 | 4.505640 |
| C | 30.69954 | 18.227690 | 4.384690 | O | 17.06614 | 6.058103 | 4.274290 | H | 11.61836 | 18.807060 | 4.515040 |
| C | 29.47297 | 18.912880 | 4.397390 | O | 19.40565 | 6.056999 | 4.289890 | H | 46.97284 | 20.028300 | 4.517290 |
| C | 28.24372 | 18.241260 | 4.422390 | C | 15.38498 | 11.066670 | 4.325940 | C | 9.48312 | 14.675450 | 4.471490 |
| H | 31.62172 | 16.267400 | 4.396490 | C | 14.73407 | 12.281060 | 4.348565 | C | 8.22453 | 13.978730 | 4.471865 |
| H | 29.47874 | 20.015110 | 4.384040 | C | 15.42947 | 13.488590 | 4.351715 | H | 8.22973 | 12.876560 | 4.468365 |
| C | 26.98487 | 18.937230 | 4.430740 | C | 16.80664 | 13.533920 | 4.334740 | C | 10.71272 | 14.003960 | 4.451290 |
| H | 26.98935 | 20.039300 | 4.424390 | O | 13.31699 | 12.550560 | 4.379815 | C | 11.93894 | 14.690060 | 4.443215 |
| H | 24.85191 | 18.817090 | 4.447115 | O | 14.48405 | 14.578850 | 4.384765 | C | 11.90796 | 16.094690 | 4.466965 |
| H | 33.57288 | 31.474380 | 4.257390 | B | 23.23516 | 13.952980 | 4.415840 | H | 10.71743 | 12.901760 | 4.435890 |
| C | 24.56276 | 16.103420 | 4.445115 | B | 18.23545 | 5.290247 | 4.279390 | H | 12.86036 | 16.649540 | 4.464165 |

**c** 0.00000 0.00000 6.83218 serrated armchair

# COF-4M

## Layer I AA, AB, inclined zigzag & armchair, serrated zigzag & armchair

| | | | | | | | |
|---|---|---|---|---|---|---|---|
| C | 16.65795 | 14.57413 | 0.8662960 | O | 11.20571 | 9.554735 | 0.8149160 |
| C | 16.69869 | 15.98028 | 0.8642160 | O | 8.386591 | 14.42251 | 0.9112960 |
| C | 15.46072 | 16.64832 | 0.8602260 | B | 28.85009 | 13.79134 | 0.9119460 |
| C | 14.25893 | 15.95477 | 0.8594960 | B | 12.92274 | 13.79939 | 0.8610660 |
| C | 14.21803 | 14.54852 | 0.8606960 | B | 10.03714 | 8.786865 | 0.8151160 |
| C | 15.45619 | 13.88052 | 0.8633260 | C | 26.31219 | 16.63906 | 0.9244460 |
| H | 15.44348 | 17.75057 | 0.8584760 | C | 25.07378 | 15.97165 | 0.9219960 |
| H | 13.31323 | 16.52131 | 0.8566460 | C | 25.11413 | 14.56548 | 0.9248460 |
| H | 15.47330 | 12.77820 | 0.8640760 | C | 26.31559 | 13.87148 | 0.9253160 |
| H | 17.60369 | 14.00774 | 0.8695460 | C | 27.55387 | 14.53887 | 0.9241960 |
| C | 11.23588 | 6.552347 | 0.7979460 | C | 27.51373 | 15.94491 | 0.9261160 |
| C | 10.03838 | 7.290568 | 0.8001260 | H | 24.16820 | 13.99940 | 0.9243960 |
| C | 8.840971 | 6.552197 | 0.7960960 | H | 26.29782 | 12.76925 | 0.9250460 |
| C | 8.840961 | 5.164634 | 0.7951960 | H | 28.45984 | 16.51109 | 0.9265760 |
| C | 10.03848 | 4.426489 | 0.7995760 | H | 26.33001 | 17.74158 | 0.9237460 |
| C | 11.23590 | 5.164728 | 0.7984960 | C | 9.340211 | 0.795383 | 0.8400960 |
| H | 7.877671 | 7.088386 | 0.7953460 | C | 19.49931 | 18.39517 | 0.8532960 |
| H | 7.877801 | 4.628389 | 0.7937660 | C | 20.19245 | 17.19146 | 0.8764660 |
| H | 12.19921 | 4.628671 | 0.8001960 | C | 21.58226 | 17.18971 | 0.8876260 |
| H | 12.19940 | 7.088141 | 0.7988760 | C | 22.27837 | 18.39192 | 0.8747960 |
| C | 10.72633 | 13.33004 | 0.8750660 | C | 10.72945 | 0.793766 | 0.8504260 |
| C | 9.336611 | 13.32796 | 0.8885460 | O | 19.24258 | 16.09653 | 0.8840160 |
| C | 8.643501 | 12.12411 | 0.8777660 | O | 22.52935 | 16.09253 | 0.9102460 |
| C | 9.340001 | 10.92161 | 0.8529160 | O | 11.20607 | 2.162620 | 0.8326760 |
| C | 10.72909 | 10.92367 | 0.8395160 | O | 8.867001 | 2.165328 | 0.8159160 |
| C | 11.42252 | 12.12811 | 0.8508760 | O | 23.70144 | 18.11697 | 0.8890960 |
| O | 7.219531 | 12.39550 | 0.8935960 | O | 18.07539 | 18.12346 | 0.8460460 |
| O | 8.866661 | 9.551425 | 0.8371960 | B | 17.99500 | 16.72763 | 0.8650460 |
| O | 12.84555 | 12.40333 | 0.8417960 | B | 10.03749 | 2.930173 | 0.8134960 |
| O | 11.67349 | 14.42733 | 0.8815960 | B | 23.77861 | 16.72083 | 0.9089960 |

Transformation Matrix

| | | | | |
|---|---|---|---|---|
| **a** | 21.711180 | 0.000000 | 0.000000 | |
| **b** | 10.855590 | 18.802430 | 0.000000 | |
| **c** | 0.000000 | 0.000000 | 3.505078 | AA |
| **c** | 1.19747 | 0.691360 | 3.437101 | inclined zigzag |
| **c** | 1.38958 | 0.00000 | 3.45078 | inclined armchair |

## Layer II AB stacking

| | | | | | | | |
|---|---|---|---|---|---|---|---|
| C | 27.51354 | 20.84161 | 4.048914 | O | 22.06130 | 15.82221 | 3.997534 |
| C | 27.55428 | 22.24776 | 4.046834 | O | 19.24218 | 20.68999 | 4.093914 |
| C | 26.31631 | 22.91580 | 4.042844 | B | 39.70568 | 20.05882 | 4.094564 |
| C | 25.11452 | 22.22225 | 4.042114 | B | 23.77833 | 20.06687 | 4.043684 |
| C | 25.07362 | 20.81600 | 4.043314 | B | 20.89273 | 15.05434 | 3.997734 |
| C | 26.31178 | 20.14800 | 4.045944 | C | 37.16778 | 22.90654 | 4.107064 |
| H | 26.29907 | 24.01805 | 4.041094 | C | 35.92937 | 22.23913 | 4.104614 |
| H | 24.16882 | 22.78879 | 4.039264 | C | 35.96972 | 20.83296 | 4.107464 |
| H | 26.32889 | 19.04568 | 4.046694 | C | 37.17118 | 20.13896 | 4.107934 |
| H | 28.45928 | 20.27522 | 4.052164 | C | 38.40946 | 20.80635 | 4.106814 |
| C | 22.09147 | 12.81983 | 3.980564 | C | 38.36932 | 22.21239 | 4.108734 |
| C | 20.89397 | 13.55805 | 3.982744 | H | 35.02379 | 20.26688 | 4.107014 |
| C | 19.69656 | 12.81968 | 3.978714 | H | 37.15341 | 19.03673 | 4.107664 |
| C | 19.69655 | 11.43211 | 3.977814 | H | 39.31543 | 22.77857 | 4.109194 |

| | | | | | | | |
|---|---|---|---|---|---|---|---|
| C | 20.89407 | 10.69397 | 3.982194 | H | 37.18560 | 24.00906 | 4.106364 |
| C | 22.09149 | 11.43221 | 3.981114 | C | 20.19580 | 7.062861 | 4.022714 |
| H | 18.73326 | 13.35586 | 3.977964 | C | 30.35490 | 24.66265 | 4.035914 |
| H | 18.73339 | 10.89587 | 3.976384 | C | 31.04804 | 23.45894 | 4.059084 |
| H | 23.05480 | 10.89615 | 3.982814 | C | 32.43785 | 23.45719 | 4.070244 |
| H | 23.05499 | 13.35562 | 3.981494 | C | 33.13396 | 24.65940 | 4.057414 |
| C | 21.58192 | 19.59752 | 4.057684 | C | 21.58504 | 7.061244 | 4.033044 |
| C | 20.19220 | 19.59544 | 4.071164 | O | 30.09817 | 22.36401 | 4.066634 |
| C | 19.49909 | 18.39159 | 4.060384 | O | 33.38494 | 22.36001 | 4.092864 |
| C | 20.19559 | 17.18909 | 4.035534 | O | 22.06166 | 8.430098 | 4.015294 |
| C | 21.58468 | 17.19115 | 4.022134 | O | 19.72259 | 8.432806 | 3.998534 |
| C | 22.27811 | 18.39559 | 4.033494 | O | 34.55703 | 24.38445 | 4.071714 |
| O | 18.07512 | 18.66298 | 4.076214 | O | 28.93098 | 24.39094 | 4.028664 |
| O | 19.72225 | 15.81890 | 4.019814 | B | 28.85059 | 22.99511 | 4.047664 |
| O | 23.70114 | 18.67081 | 4.024414 | B | 20.89308 | 9.197651 | 3.996114 |
| O | 22.52908 | 20.69481 | 4.064214 | B | 34.63420 | 22.98831 | 4.091614 |
| **c** | 0.00000 | 0.00000 | 6.36524 | AB | | | |

## Layer II serrated zigzag

| | | | | | | | |
|---|---|---|---|---|---|---|---|
| C | 17.85542 | 15.265490 | 4.306816 | O | 12.40318 | 10.246100 | 4.255436 |
| C | 17.89616 | 16.671640 | 4.304736 | O | 9.58406 | 15.113870 | 4.351816 |
| C | 16.65819 | 17.339680 | 4.300746 | B | 30.04756 | 14.482700 | 4.352466 |
| C | 15.45640 | 16.646130 | 4.300016 | B | 14.12021 | 14.490750 | 4.301586 |
| C | 15.41550 | 15.239880 | 4.301216 | B | 11.23461 | 9.478226 | 4.255636 |
| C | 16.65366 | 14.571880 | 4.303846 | C | 27.50966 | 17.330420 | 4.364966 |
| H | 16.64095 | 18.441930 | 4.298996 | C | 26.27125 | 16.663010 | 4.362516 |
| H | 14.51070 | 17.212670 | 4.297166 | C | 26.31160 | 15.256840 | 4.365366 |
| H | 16.67077 | 13.469560 | 4.304596 | C | 27.51306 | 14.562840 | 4.365836 |
| H | 18.80116 | 14.699100 | 4.310066 | C | 28.75134 | 15.230230 | 4.364716 |
| C | 12.43335 | 7.243707 | 4.238466 | C | 28.71120 | 16.636270 | 4.366636 |
| C | 11.23585 | 7.981928 | 4.240646 | H | 25.36567 | 14.690760 | 4.364916 |
| C | 10.03844 | 7.243557 | 4.236616 | H | 27.49529 | 13.460610 | 4.365566 |
| C | 10.03843 | 5.855994 | 4.235716 | H | 29.65731 | 17.202450 | 4.367096 |
| C | 11.23595 | 5.117849 | 4.240096 | H | 27.52748 | 18.432940 | 4.364266 |
| C | 12.43337 | 5.856088 | 4.239016 | C | 10.53768 | 1.486743 | 4.280616 |
| H | 9.07514 | 7.779746 | 4.235866 | C | 9.84119 | 0.284100 | 4.293816 |
| H | 9.07527 | 5.319749 | 4.234286 | C | 21.38992 | 17.882820 | 4.316986 |
| H | 13.39668 | 5.320031 | 4.240716 | C | 22.77973 | 17.881070 | 4.328146 |
| H | 13.39687 | 7.779501 | 4.239396 | C | 12.62025 | 0.280850 | 4.315316 |
| C | 11.92380 | 14.021400 | 4.315586 | C | 11.92692 | 1.485126 | 4.290946 |
| C | 10.53408 | 14.019320 | 4.329066 | O | 20.44005 | 16.787890 | 4.324536 |
| C | 9.84097 | 12.815470 | 4.318286 | O | 23.72682 | 16.783890 | 4.350766 |
| C | 10.53747 | 11.612970 | 4.293436 | O | 12.40354 | 2.853980 | 4.273196 |
| C | 11.92656 | 11.615030 | 4.280036 | O | 10.06447 | 2.856688 | 4.256436 |
| C | 12.61999 | 12.819470 | 4.291396 | O | 14.04332 | 0.005900 | 4.329616 |
| O | 8.41700 | 13.086860 | 4.334116 | O | 8.41727 | 0.012390 | 4.286566 |
| O | 10.06413 | 10.242780 | 4.277716 | B | 19.19247 | 17.418990 | 4.305566 |
| O | 14.04302 | 13.094690 | 4.282316 | B | 11.23496 | 3.621533 | 4.254016 |
| O | 12.87096 | 15.118690 | 4.322116 | B | 24.97608 | 17.412190 | 4.349516 |
| **c** | 0.00000 | 0.00000 | 6.88104 | serrated zigzag | | | |

## Layer II serrated armchair

| | | | | | | | |
|---|---|---|---|---|---|---|---|
| C | 18.047530 | 14.574130 | 4.310236 | O | 12.595290 | 9.554735 | 4.258856 |
| C | 18.088270 | 15.980280 | 4.308156 | O | 9.776171 | 14.422510 | 4.355236 |
| C | 16.850300 | 16.648320 | 4.304166 | B | 8.528490 | 13.791340 | 4.355886 |
| C | 15.648510 | 15.954770 | 4.303436 | B | 14.312320 | 13.799390 | 4.305006 |

| | | | | | | | |
|---|---|---|---|---|---|---|---|
| C | 15.607610 | 14.548520 | 4.304636 | B | 11.426720 | 8.786865 | 4.259056 |
| C | 16.845770 | 13.880520 | 4.307266 | C | 27.701770 | 16.639060 | 4.368386 |
| H | 16.833060 | 17.750570 | 4.302416 | C | 26.463360 | 15.971650 | 4.365936 |
| H | 14.702810 | 16.521310 | 4.300586 | C | 26.503710 | 14.565480 | 4.368786 |
| H | 16.862880 | 12.778200 | 4.308016 | C | 27.705170 | 13.871480 | 4.369256 |
| H | 18.993270 | 14.007740 | 4.313486 | C | 28.943450 | 14.538870 | 4.368136 |
| C | 12.625460 | 6.552347 | 4.241886 | C | 28.903310 | 15.944910 | 4.370056 |
| C | 11.427960 | 7.290568 | 4.244066 | H | 25.557780 | 13.999400 | 4.368336 |
| C | 10.230550 | 6.552197 | 4.240036 | H | 27.687400 | 12.769250 | 4.368986 |
| C | 10.230540 | 5.164634 | 4.239136 | H | 29.849420 | 16.511090 | 4.370516 |
| C | 11.428060 | 4.426489 | 4.243516 | H | 27.719590 | 17.741580 | 4.367686 |
| C | 12.625480 | 5.164728 | 4.242436 | C | 10.729790 | 0.795383 | 4.284036 |
| H | 9.267251 | 7.088386 | 4.239286 | C | 20.888890 | 18.395170 | 4.297236 |
| H | 9.267381 | 4.628389 | 4.237706 | C | 21.582030 | 17.191460 | 4.320406 |
| H | 13.588790 | 4.628671 | 4.244136 | C | 22.971840 | 17.189710 | 4.331566 |
| H | 13.588980 | 7.088141 | 4.242816 | C | 23.667950 | 18.391920 | 4.318736 |
| C | 12.115910 | 13.330040 | 4.319006 | C | 12.119030 | 0.793766 | 4.294366 |
| C | 10.726190 | 13.327960 | 4.332486 | O | 20.632160 | 16.096530 | 4.327956 |
| C | 10.033080 | 12.124110 | 4.321706 | O | 23.918930 | 16.092530 | 4.354186 |
| C | 10.729580 | 10.921610 | 4.296856 | O | 12.595650 | 2.162620 | 4.276616 |
| C | 12.118670 | 10.923670 | 4.283456 | O | 10.256580 | 2.165328 | 4.259856 |
| C | 12.812100 | 12.128110 | 4.294816 | O | 25.091020 | 18.116970 | 4.333036 |
| O | 8.609111 | 12.395500 | 4.337536 | O | 19.464970 | 18.123460 | 4.289986 |
| O | 10.256240 | 9.551425 | 4.281136 | B | 19.384580 | 16.727630 | 4.308986 |
| O | 14.235130 | 12.403330 | 4.285736 | B | 11.427070 | 2.930173 | 4.257436 |
| O | 13.063070 | 14.427330 | 4.325536 | B | 25.168190 | 16.720830 | 4.352936 |
| **c** | 0.00000 | 0.00000 | 6.88788 | serrated armchair | | | |

# COF-5M

## Layer I AA, AB, inclined zigzag & armchair, and serrated zigzag & armchair

| | | | | | | | | | | | |
|---|---|---|---|---|---|---|---|---|---|---|---|
| C | 13.01181 | 22.34097 | 0.9135870 | C | 27.58970 | 2.790600 | 0.8839620 | C | 22.23723 | 10.86297 | 1.022137 |
| C | 15.41276 | 23.73070 | 0.9347870 | C | 27.58465 | 1.403850 | 0.8886370 | H | 25.59842 | 12.79208 | 1.050612 |
| C | 15.41148 | 22.34430 | 0.9440870 | H | 30.95196 | 3.323317 | 0.9137120 | H | 23.41300 | 9.062314 | 1.004562 |
| C | 14.21256 | 21.60599 | 0.9334870 | H | 26.62894 | 3.330941 | 0.8722620 | C | 10.05809 | 12.90808 | 0.8579870 |
| H | 41.20082 | 21.80104 | 0.9047870 | O | 31.64852 | 8.606116 | 0.8449620 | C | 8.858518 | 12.21246 | 0.8419870 |
| C | 14.96698 | 15.57696 | 0.9486620 | O | 30.49775 | 10.64400 | 0.8876870 | C | 7.630246 | 12.89205 | 0.8480120 |
| C | 15.64707 | 16.78833 | 0.9593370 | B | 31.73979 | 10.00195 | 0.8538870 | C | 36.80516 | 14.29538 | 0.8685620 |
| C | 14.93871 | 17.98314 | 0.9511120 | O | 26.02376 | 8.666956 | 0.9843370 | C | 8.856944 | 14.98615 | 0.8834870 |
| C | 13.57766 | 15.56085 | 0.9302120 | B | 25.96297 | 10.06458 | 0.9972620 | H | 11.00543 | 12.34449 | 0.8538120 |
| C | 12.86939 | 16.75619 | 0.9236620 | O | 27.21853 | 10.67924 | 0.9731370 | C | 35.50769 | 12.15967 | 0.8380370 |
| C | 13.54972 | 17.96746 | 0.9337370 | O | 17.07419 | 16.53399 | 0.9789870 | H | 37.99348 | 16.08834 | 0.9006370 |
| O | 15.39925 | 19.35711 | 0.9559620 | O | 15.92897 | 14.49269 | 0.9607870 | C | 34.28146 | 12.84275 | 0.8498120 |
| O | 13.05913 | 19.33217 | 0.9269120 | O | 12.64145 | 14.45389 | 0.9142620 | C | 33.08038 | 12.15033 | 0.8502120 |
| B | 14.22106 | 20.11042 | 0.9397870 | O | 11.44886 | 16.46757 | 0.9046370 | C | 33.03878 | 10.74288 | 0.8382120 |
| C | 42.15765 | 23.72782 | 0.9043120 | B | 17.16970 | 15.13879 | 0.9795870 | C | 34.27464 | 10.06875 | 0.8206120 |
| C | 14.20938 | 24.45350 | 0.9143370 | B | 11.38620 | 15.07040 | 0.8991620 | C | 6.328531 | 10.75647 | 0.8206620 |
| H | 16.37365 | 21.80646 | 0.9598370 | C | 18.47296 | 14.40545 | 0.9982620 | H | 32.13431 | 12.71617 | 0.8616120 |
| C | 28.15192 | 9.571300 | 0.9397620 | C | 10.09526 | 14.31548 | 0.8796370 | H | 34.28576 | 8.966738 | 0.8086620 |
| C | 29.54024 | 9.556532 | 0.9030870 | C | 18.52074 | 12.99846 | 0.9926370 | H | 23.46207 | 14.04228 | 1.061062 |
| C | 27.44410 | 8.375884 | 0.9472370 | C | 19.72498 | 12.31243 | 1.006762 | H | 21.27807 | 10.31708 | 1.013437 |
| C | 28.12549 | 7.164942 | 0.9204370 | C | 20.94826 | 13.00050 | 1.027462 | H | 21.86387 | 14.96439 | 1.051237 |
| C | 29.51459 | 7.150704 | 0.8873120 | C | 20.91346 | 14.40361 | 1.035062 | H | 19.73294 | 11.20832 | 1.000262 |
| C | 30.22199 | 8.346423 | 0.8773870 | C | 19.70584 | 15.08559 | 1.020637 | H | 16.36911 | 24.28176 | 0.9433620 |
| O | 27.63679 | 5.798896 | 0.9228870 | H | 17.57731 | 12.42820 | 0.9751370 | H | 1.794930 | 0.846800 | 0.9256120 |
| O | 29.97603 | 5.776882 | 0.8704870 | C | 22.22023 | 12.26659 | 1.034562 | H | 26.62709 | 0.855000 | 0.8810370 |

| | | | | | | | | | | | |
|---|---|---|---|---|---|---|---|---|---|---|---|
| B | 28.79922 | 5.021281 | 0.8938120 | H | 19.71152 | 16.18803 | 1.025862 | H | 41.20013 | 24.27656 | 0.8885120 |
| C | 28.78563 | 0.677480 | 0.9047870 | C | 23.45246 | 12.93888 | 1.048287 | H | 35.84981 | 14.84786 | 0.8738370 |
| C | 0.839300 | 1.399460 | 0.9128370 | C | 24.64747 | 12.23470 | 1.042037 | H | 8.859699 | 11.10883 | 0.8255620 |
| C | 29.98927 | 2.786030 | 0.9059120 | C | 24.67650 | 10.82685 | 1.023087 | H | 34.28357 | 13.94647 | 0.8612370 |
| C | 28.79080 | 3.525527 | 0.8927620 | C | 23.43431 | 10.16420 | 1.017012 | H | 7.281887 | 10.20058 | 0.8091370 |

Transformation Matrix

| | | | | |
|---|---|---|---|---|
| **a** | 29.150180 | 0.000000 | 0.000000 | |
| **b** | 14.575090 | 25.244790 | 0.000000 | |
| **c** | 0.000000 | 0.000000 | 3.504000 | AA |
| **c** | 1.19993 | 0.69278 | 3.433682 | inclined zigzag |
| **c** | 1.38905 | 0.00000 | 3.43368 | inclined armchair |

## Layer II AB stacking

| | | | | | | | | | | | |
|---|---|---|---|---|---|---|---|---|---|---|---|
| C | 27.58690 | 30.75590 | 4.091712 | C | 42.16479 | 11.20553 | 4.062087 | C | 36.81232 | 19.27790 | 4.200262 |
| C | 29.98785 | 32.14563 | 4.112912 | C | 42.15974 | 9.818782 | 4.066762 | H | 40.17351 | 21.20701 | 4.228737 |
| C | 29.98657 | 30.75923 | 4.122212 | H | 45.52705 | 11.73825 | 4.091837 | H | 37.98809 | 17.47725 | 4.182687 |
| C | 28.78765 | 30.02092 | 4.111612 | H | 41.20403 | 11.74587 | 4.050387 | C | 24.63318 | 21.32301 | 4.036112 |
| H | 55.77591 | 30.21597 | 4.082912 | O | 46.22361 | 17.02105 | 4.023087 | C | 23.43361 | 20.62739 | 4.020112 |
| C | 29.54207 | 23.99189 | 4.126787 | O | 45.07284 | 19.05893 | 4.065812 | C | 22.20534 | 21.30698 | 4.026137 |
| C | 30.22216 | 25.20326 | 4.137462 | B | 46.31488 | 18.41688 | 4.032012 | C | 51.38025 | 22.71031 | 4.046687 |
| C | 29.51380 | 26.39807 | 4.129237 | O | 40.59885 | 17.08189 | 4.162462 | C | 23.43203 | 23.40108 | 4.061612 |
| C | 28.15275 | 23.97578 | 4.108337 | B | 40.53806 | 18.47951 | 4.175387 | H | 25.58052 | 20.75942 | 4.031937 |
| C | 27.44448 | 25.17112 | 4.101787 | O | 41.79362 | 19.09417 | 4.151262 | C | 50.08278 | 20.57460 | 4.016162 |
| C | 28.12481 | 26.38239 | 4.111862 | O | 31.64928 | 24.94892 | 4.157112 | H | 52.56857 | 24.50327 | 4.078762 |
| O | 29.97434 | 27.77204 | 4.134087 | O | 30.50406 | 22.90762 | 4.138912 | C | 48.85655 | 21.25768 | 4.027937 |
| O | 27.63422 | 27.74710 | 4.105037 | O | 27.21654 | 22.86882 | 4.092387 | C | 47.65547 | 20.56526 | 4.028337 |
| B | 28.79615 | 28.52535 | 4.117912 | O | 26.02395 | 24.88250 | 4.082762 | C | 47.61387 | 19.15781 | 4.016337 |
| C | 56.73274 | 32.14275 | 4.082437 | B | 31.74479 | 23.55372 | 4.157712 | C | 48.84973 | 18.48368 | 3.998737 |
| C | 28.78447 | 32.86843 | 4.092462 | B | 25.96129 | 23.48533 | 4.077287 | C | 20.90362 | 19.17140 | 3.998787 |
| H | 30.94874 | 30.22139 | 4.137962 | C | 33.04805 | 22.82038 | 4.176387 | H | 46.70940 | 21.13110 | 4.039737 |
| C | 42.72701 | 17.98623 | 4.117887 | C | 24.67035 | 22.73041 | 4.057762 | H | 48.86085 | 17.38167 | 3.986787 |
| C | 44.11533 | 17.97146 | 4.081212 | C | 33.09583 | 21.41339 | 4.170762 | H | 38.03716 | 22.45721 | 4.239187 |
| C | 42.01919 | 16.79082 | 4.125362 | C | 34.30007 | 20.72736 | 4.184887 | H | 35.85316 | 18.73201 | 4.191562 |
| C | 42.70058 | 15.57987 | 4.098562 | C | 35.52335 | 21.41543 | 4.205587 | H | 36.43896 | 23.37932 | 4.229362 |
| C | 44.08968 | 15.56564 | 4.065437 | C | 35.48855 | 22.81854 | 4.213187 | H | 34.30803 | 19.62325 | 4.178387 |
| C | 44.79708 | 16.76136 | 4.055512 | C | 34.28093 | 23.50052 | 4.198762 | H | 30.94420 | 32.69669 | 4.121487 |
| O | 42.21188 | 14.21383 | 4.101012 | H | 32.15240 | 20.84313 | 4.153262 | H | 16.37002 | 9.261732 | 4.103737 |
| O | 44.55112 | 14.19181 | 4.048612 | C | 36.79532 | 20.68152 | 4.212687 | H | 41.20218 | 9.269932 | 4.059162 |
| B | 43.37431 | 13.43621 | 4.071937 | H | 34.28661 | 24.60296 | 4.203987 | H | 55.77522 | 32.69149 | 4.066637 |
| C | 43.36072 | 9.092412 | 4.082912 | C | 38.02755 | 21.35381 | 4.226412 | H | 50.42490 | 23.26279 | 4.051962 |
| C | 15.41439 | 9.814392 | 4.090962 | C | 39.22256 | 20.64963 | 4.220162 | H | 23.43479 | 19.52376 | 4.003687 |
| C | 44.56436 | 11.20096 | 4.084037 | C | 39.25159 | 19.24178 | 4.201212 | H | 48.85866 | 22.36140 | 4.039362 |
| C | 43.36589 | 11.94046 | 4.070887 | C | 38.00940 | 18.57913 | 4.195137 | H | 21.85698 | 18.61551 | 3.987262 |

**c** 0.00000 0.00000 6.35625 AB

## Layer II serrated zigzag

| | | | | | | | | | | | |
|---|---|---|---|---|---|---|---|---|---|---|---|
| C | 14.21174 | 23.033750 | 4.337007 | C | 28.78963 | 3.483380 | 4.307382 | C | 23.43716 | 11.555750 | 4.445557 |
| C | 16.61269 | 24.423480 | 4.358207 | C | 28.78458 | 2.096630 | 4.312057 | H | 26.79835 | 13.484860 | 4.474032 |
| C | 16.61141 | 23.037080 | 4.367507 | H | 3.00171 | 4.016097 | 4.337132 | H | 24.61293 | 9.755095 | 4.427982 |
| C | 15.41249 | 22.298770 | 4.356907 | H | 27.82887 | 4.023721 | 4.295682 | C | 11.25802 | 13.600860 | 4.281407 |
| H | 13.25057 | 22.493820 | 4.328207 | O | 32.84845 | 9.298897 | 4.268382 | C | 10.05845 | 12.905240 | 4.265407 |
| C | 16.16691 | 16.269740 | 4.372082 | O | 31.69768 | 11.336780 | 4.311107 | C | 8.83017 | 13.584830 | 4.271432 |
| C | 16.84700 | 17.481110 | 4.382757 | B | 32.93972 | 10.694730 | 4.277307 | C | 8.85491 | 14.988160 | 4.291982 |
| C | 16.13864 | 18.675920 | 4.374532 | O | 27.22369 | 9.359735 | 4.407757 | C | 10.05687 | 15.678930 | 4.306907 |
| C | 14.77759 | 16.253630 | 4.353632 | B | 27.16290 | 10.757360 | 4.420682 | H | 12.20536 | 13.037270 | 4.277232 |
| C | 14.06932 | 17.448970 | 4.347082 | O | 28.41846 | 11.372020 | 4.396557 | C | 7.55744 | 12.852450 | 4.261457 |
| C | 14.74965 | 18.660240 | 4.357157 | O | 18.27412 | 17.226770 | 4.402407 | H | 10.04323 | 16.781120 | 4.324057 |
| O | 16.59918 | 20.049890 | 4.379382 | O | 17.12890 | 15.185470 | 4.384207 | C | 35.48139 | 13.535530 | 4.273232 |
| O | 14.25906 | 20.024950 | 4.350332 | O | 13.84138 | 15.146670 | 4.337682 | C | 34.28031 | 12.843110 | 4.273632 |
| B | 15.42099 | 20.803200 | 4.363207 | O | 12.64879 | 17.160350 | 4.328057 | C | 34.23871 | 11.435660 | 4.261632 |

| | | | | | | | | | | | |
|---|---|---|---|---|---|---|---|---|---|---|---|
| C | 14.20740 | 24.420600 | 4.327732 | B | 18.36963 | 15.831570 | 4.403007 | C | 6.32439 | 10.761530 | 4.244032 |
| C | 15.40931 | 25.146280 | 4.337757 | B | 12.58613 | 15.763180 | 4.322582 | C | 7.52846 | 11.449250 | 4.244082 |
| H | 17.57358 | 22.499240 | 4.383257 | C | 19.67289 | 15.098230 | 4.421682 | H | 33.33424 | 13.408950 | 4.285032 |
| C | 29.35185 | 10.264080 | 4.363182 | C | 11.29519 | 15.008260 | 4.303057 | H | 6.33550 | 9.659517 | 4.232082 |
| C | 30.74017 | 10.249310 | 4.326507 | C | 19.72067 | 13.691240 | 4.416057 | H | 24.66200 | 14.735060 | 4.484482 |
| C | 28.64403 | 9.068665 | 4.370657 | C | 20.92491 | 13.005210 | 4.430182 | H | 22.47800 | 11.009860 | 4.436857 |
| C | 29.32542 | 7.857722 | 4.343857 | C | 22.14819 | 13.693280 | 4.450882 | H | 23.06380 | 15.657170 | 4.474657 |
| C | 30.71452 | 7.843484 | 4.310732 | C | 22.11339 | 15.096390 | 4.458482 | H | 20.93287 | 11.901100 | 4.423682 |
| C | 31.42192 | 9.039204 | 4.300807 | C | 20.90577 | 15.778370 | 4.444057 | H | 17.56904 | 24.974540 | 4.366782 |
| O | 28.83672 | 6.491676 | 4.346307 | H | 18.77724 | 13.120980 | 4.398557 | H | 2.99486 | 1.539580 | 4.349032 |
| O | 31.17596 | 6.469662 | 4.293907 | C | 23.42016 | 12.959370 | 4.457982 | H | 27.82702 | 1.547780 | 4.304457 |
| B | 29.99915 | 5.714061 | 4.317232 | H | 20.91145 | 16.880810 | 4.449282 | H | 42.40006 | 24.969340 | 4.311932 |
| C | 0.83538 | 1.370260 | 4.328207 | C | 24.65239 | 13.631660 | 4.471707 | H | 37.04974 | 15.540640 | 4.297257 |
| C | 2.03923 | 2.092240 | 4.336257 | C | 25.84740 | 12.927480 | 4.465457 | H | 10.05963 | 11.801610 | 4.248982 |
| C | 2.03902 | 3.478810 | 4.329332 | C | 25.87643 | 11.519630 | 4.446507 | H | 35.48350 | 14.639250 | 4.284657 |
| C | 29.99073 | 4.218307 | 4.316182 | C | 24.63424 | 10.856980 | 4.440432 | H | 8.48181 | 10.893360 | 4.232557 |

**c** 0.00000 0.00000 6.84684 serrated zigzag

## Layer II serrated armchair

| | | | | | | | | | | | |
|---|---|---|---|---|---|---|---|---|---|---|---|
| C | 14.400860 | 22.340970 | 4.340427 | C | 28.978750 | 2.790600 | 4.310802 | C | 23.626280 | 10.862970 | 4.448977 |
| C | 16.801810 | 23.730700 | 4.361627 | C | 28.973700 | 1.403850 | 4.315477 | H | 26.987470 | 12.792080 | 4.477452 |
| C | 16.800530 | 22.344300 | 4.370927 | H | 3.190830 | 3.323317 | 4.340552 | H | 24.802050 | 9.062314 | 4.431402 |
| C | 15.601610 | 21.605990 | 4.360327 | H | 28.017990 | 3.330941 | 4.299102 | C | 11.447140 | 12.908080 | 4.284827 |
| H | 13.439690 | 21.801040 | 4.331627 | O | 33.037570 | 8.606116 | 4.271802 | C | 10.247570 | 12.212460 | 4.268827 |
| C | 16.356030 | 15.576960 | 4.375502 | O | 31.886800 | 10.644000 | 4.314527 | C | 9.019297 | 12.892050 | 4.274852 |
| C | 17.036120 | 16.788330 | 4.386177 | B | 33.128840 | 10.001950 | 4.280727 | C | 9.044030 | 14.295380 | 4.295402 |
| C | 16.327760 | 17.983140 | 4.377952 | O | 27.412810 | 8.666956 | 4.411177 | C | 10.245990 | 14.986150 | 4.310327 |
| C | 14.966710 | 15.560850 | 4.357052 | B | 27.352020 | 10.064580 | 4.424102 | H | 12.394480 | 12.344490 | 4.280652 |
| C | 14.258440 | 16.756190 | 4.350502 | O | 28.607580 | 10.679240 | 4.399977 | C | 7.746560 | 12.159670 | 4.264877 |
| C | 14.938770 | 17.967460 | 4.360577 | O | 18.463240 | 16.533990 | 4.405827 | H | 10.232350 | 16.088340 | 4.327477 |
| O | 16.788300 | 19.357110 | 4.382802 | O | 17.318020 | 14.492690 | 4.387627 | C | 35.670510 | 12.842750 | 4.276652 |
| O | 14.448180 | 19.332170 | 4.353752 | O | 14.030500 | 14.453890 | 4.341102 | C | 34.469430 | 12.150330 | 4.277052 |
| B | 15.610110 | 20.110420 | 4.366627 | O | 12.837910 | 16.467570 | 4.331477 | C | 34.427830 | 10.742880 | 4.265052 |
| C | 14.396520 | 23.727820 | 4.331152 | B | 18.558750 | 15.138790 | 4.406427 | C | 6.513510 | 10.068750 | 4.247452 |
| C | 15.598430 | 24.453500 | 4.341177 | B | 12.775250 | 15.070400 | 4.326002 | C | 7.717581 | 10.756470 | 4.247502 |
| H | 17.762700 | 21.806460 | 4.386677 | C | 19.862010 | 14.405450 | 4.425102 | H | 33.523360 | 12.716170 | 4.288452 |
| C | 29.540970 | 9.571300 | 4.366602 | C | 11.484310 | 14.315480 | 4.306477 | H | 6.524620 | 8.966738 | 4.235502 |
| C | 30.929290 | 9.556532 | 4.329927 | C | 19.909790 | 12.998460 | 4.419477 | H | 24.851120 | 14.042280 | 4.487902 |
| C | 28.833150 | 8.375884 | 4.374077 | C | 21.114030 | 12.312430 | 4.433602 | H | 22.667120 | 10.317080 | 4.440277 |
| C | 29.514540 | 7.164942 | 4.347277 | C | 22.337310 | 13.000500 | 4.454302 | H | 23.252920 | 14.964390 | 4.478077 |
| C | 30.903640 | 7.150704 | 4.314152 | C | 22.302510 | 14.403610 | 4.461902 | H | 21.121990 | 11.208320 | 4.427102 |
| C | 31.611040 | 8.346423 | 4.304227 | C | 21.094890 | 15.085590 | 4.447477 | H | 17.758160 | 24.281760 | 4.370202 |
| O | 29.025840 | 5.798896 | 4.349727 | H | 18.966360 | 12.428200 | 4.401977 | H | 3.183980 | 0.846800 | 4.352452 |
| O | 31.365080 | 5.776882 | 4.297327 | C | 23.609280 | 12.266590 | 4.461402 | H | 28.016140 | 0.855000 | 4.307877 |
| B | 30.188270 | 5.021281 | 4.320652 | H | 21.100570 | 16.188030 | 4.452702 | H | 42.589180 | 24.276560 | 4.315352 |
| C | 1.024500 | 0.677480 | 4.331627 | C | 24.841510 | 12.938880 | 4.475127 | H | 37.238860 | 14.847860 | 4.300677 |
| C | 2.228350 | 1.399460 | 4.339677 | C | 26.036520 | 12.234700 | 4.468877 | H | 10.248750 | 11.108830 | 4.252402 |
| C | 2.228140 | 2.786030 | 4.332752 | C | 26.065550 | 10.826850 | 4.449927 | H | 35.672620 | 13.946470 | 4.288077 |
| C | 30.179850 | 3.525527 | 4.319602 | C | 24.823360 | 10.164200 | 4.443852 | H | 8.670937 | 10.200580 | 4.235977 |

**c** 0.00000 0.00000 6.85368 serrated armchair

# COF-6M

## Layer I AA, AB, inclined zigzag & armchair, serrated zigzag & armchair

| | | | | | | | |
|---|---|---|---|---|---|---|---|
| C | 2.069789 | 2.222231 | 0.9642750 | B | 24.51850 | 15.19163 | 0.9585000 |
| C | 4.503057 | 3.615478 | 0.9920000 | O | 18.81151 | 13.76917 | 0.9918500 |
| C | 4.516872 | 2.213752 | 1.004500 | B | 18.72369 | 15.16558 | 1.007625 |
| C | 3.290919 | 1.533999 | 0.9900750 | O | 19.97116 | 15.79992 | 1.001375 |

| | | | | | | | |
|---|---|---|---|---|---|---|---|
| C | 19.12395 | 1.488225 | 0.9549250 | H | 5.462789 | 4.164466 | 1.002400 |
| C | 2.094722 | 3.623877 | 0.9524000 | H | 5.465576 | 5.986796 | 0.9831000 |
| C | 3.301635 | 4.337306 | 0.9658750 | H | 19.48176 | 5.994481 | 0.9224500 |
| C | 5.791527 | 1.472347 | 1.023550 | H | 19.47330 | 4.180899 | 0.9322500 |
| C | 20.92758 | 14.70809 | 0.9810750 | H | 3.287207 | 0.428162 | 0.9989750 |
| C | 22.31848 | 14.71442 | 0.9700000 | C | 5.816369 | 0.068796 | 1.027350 |
| C | 20.23823 | 13.50168 | 0.9754750 | C | 16.18345 | 15.24957 | 1.026825 |
| C | 20.93885 | 12.30068 | 0.9595250 | C | 8.260023 | 0.034787 | 1.023850 |
| C | 22.32830 | 12.30689 | 0.9511500 | C | 8.221853 | 1.443006 | 1.029450 |
| C | 23.01829 | 13.51413 | 0.9560750 | C | 7.024327 | 2.143892 | 1.029575 |
| O | 20.47047 | 10.92726 | 0.9532000 | H | 14.02684 | 15.39445 | 1.026575 |
| O | 22.80925 | 10.93757 | 0.9409500 | H | 16.16085 | 14.14730 | 1.025400 |
| B | 21.64331 | 10.16396 | 0.9437500 | H | 9.170374 | 2.004966 | 1.030050 |
| C | 21.63993 | 5.811987 | 0.9546500 | H | 7.033475 | 3.247776 | 1.030375 |
| C | 4.508557 | 6.536959 | 0.9669000 | C | 17.89443 | 2.165613 | 0.9377000 |
| C | 22.84239 | 7.924728 | 0.9602750 | C | 16.69275 | 1.471379 | 0.9388750 |
| C | 21.64565 | 8.667626 | 0.9422000 | C | 16.64719 | 0.063382 | 0.9549250 |
| C | 20.44625 | 7.929031 | 0.9281750 | C | 27.05415 | 15.27235 | 0.9677500 |
| C | 20.44070 | 6.541469 | 0.9334750 | C | 0.757990 | 0.084705 | 0.9681500 |
| H | 5.472964 | 8.459459 | 0.9717250 | H | 17.89104 | 3.269416 | 0.9252500 |
| H | 19.48475 | 8.468255 | 0.9127250 | H | 15.74778 | 2.039150 | 0.9272750 |
| O | 24.44228 | 13.79426 | 0.9496500 | H | 8.736521 | 14.17002 | 0.9806750 |
| O | 23.26568 | 15.81503 | 0.9714500 | H | 10.87745 | 15.40432 | 0.9807750 |

Transformation Matrix

| | | | | |
|---|---|---|---|---|
| **a** | 18.333510 | 0.000000 | 0.000000 | |
| **b** | 9.166750 | 15.877270 | 0.000000 | |
| **c** | 0.000000 | 0.000000 | 3.446880 | AA |
| **c** | 1.22371 | 0.70651 | 3.41316 | inclined zigzag |
| **c** | 1.390080 | 0.000000 | 3.423420 | inclined armchair |

## Layer II AB stacking

| | | | | | | | |
|---|---|---|---|---|---|---|---|
| C | 11.23654 | 7.514659 | 4.145750 | B | 33.68525 | 20.48406 | 4.139975 |
| C | 13.66981 | 8.907907 | 4.173475 | O | 27.97826 | 19.06160 | 4.173325 |
| C | 13.68363 | 7.506181 | 4.185975 | B | 27.89045 | 20.45801 | 4.189100 |
| C | 12.45767 | 6.826427 | 4.171550 | O | 29.13792 | 21.09235 | 4.182850 |
| C | 28.29070 | 6.780653 | 4.136400 | H | 14.62954 | 9.456894 | 4.183875 |
| C | 11.26148 | 8.916306 | 4.133875 | H | 14.63233 | 11.27922 | 4.164575 |
| C | 12.46839 | 9.629734 | 4.147350 | H | 28.64851 | 11.28691 | 4.103925 |
| C | 14.95828 | 6.764775 | 4.205025 | H | 28.64006 | 9.473328 | 4.113725 |
| C | 30.09433 | 20.00052 | 4.162550 | H | 12.45396 | 5.720591 | 4.180450 |
| C | 31.48523 | 20.00685 | 4.151475 | C | 14.98312 | 5.361225 | 4.208825 |
| C | 29.40498 | 18.79411 | 4.156950 | C | 25.35020 | 20.54200 | 4.208300 |
| C | 30.10561 | 17.59311 | 4.141000 | C | 17.42678 | 5.327216 | 4.205325 |
| C | 31.49506 | 17.59932 | 4.132625 | C | 17.38861 | 6.735435 | 4.210925 |
| C | 32.18504 | 18.80656 | 4.137550 | C | 16.19108 | 7.436320 | 4.211050 |
| O | 29.63723 | 16.21969 | 4.134675 | H | 23.19360 | 20.68688 | 4.208050 |
| O | 31.97601 | 16.23000 | 4.122425 | H | 25.32761 | 19.43973 | 4.206875 |
| B | 30.81007 | 15.45639 | 4.125225 | H | 18.33713 | 7.297395 | 4.211525 |
| C | 30.80669 | 11.10442 | 4.136125 | H | 16.20023 | 8.540205 | 4.211850 |
| C | 13.67531 | 11.82939 | 4.148375 | C | 27.06118 | 7.458041 | 4.119175 |
| C | 32.00914 | 13.21716 | 4.141750 | C | 25.85950 | 6.763807 | 4.120350 |
| C | 30.81240 | 13.96005 | 4.123675 | C | 25.81395 | 5.355811 | 4.136400 |
| C | 29.61301 | 13.22146 | 4.109650 | C | 36.22091 | 20.56478 | 4.149225 |
| C | 29.60746 | 11.83390 | 4.114950 | C | 9.924745 | 5.377133 | 4.149625 |
| H | 14.63972 | 13.75189 | 4.153200 | H | 27.05780 | 8.561845 | 4.106725 |
| H | 28.65150 | 13.76068 | 4.094200 | H | 24.91454 | 7.331578 | 4.108750 |

| | | | | | | | |
|---|---|---|---|---|---|---|---|
| O | 33.60904 | 19.08669 | 4.131125 | H | 17.90327 | 19.46245 | 4.162150 |
| O | 32.43243 | 21.10746 | 4.152925 | H | 20.04420 | 20.69675 | 4.162250 |
| **c** | 0.00000 | 0.00000 | 6.36295 | AB | | | |

## Layer II    serrated zigzag

| | | | | | | | |
|---|---|---|---|---|---|---|---|
| C | 3.293499 | 2.928741 | 4.374015 | B | 16.575460 | 0.020870 | 4.368240 |
| C | 5.726767 | 4.321988 | 4.401740 | O | 20.035220 | 14.475680 | 4.401590 |
| C | 5.740582 | 2.920262 | 4.414240 | B | 19.947400 | 15.872090 | 4.417365 |
| C | 4.514629 | 2.240509 | 4.399815 | O | 12.028120 | 0.629160 | 4.411115 |
| C | 2.014150 | 2.194735 | 4.364665 | H | 6.686499 | 4.870976 | 4.412140 |
| C | 3.318432 | 4.330387 | 4.362140 | H | 6.689286 | 6.693306 | 4.392840 |
| C | 4.525345 | 5.043816 | 4.375615 | H | 20.705470 | 6.700991 | 4.332190 |
| C | 7.015237 | 2.178857 | 4.433290 | H | 20.697010 | 4.887409 | 4.341990 |
| C | 22.151290 | 15.414600 | 4.390815 | H | 4.510917 | 1.134672 | 4.408715 |
| C | 23.542190 | 15.420930 | 4.379740 | C | 7.040079 | 0.775306 | 4.437090 |
| C | 21.461940 | 14.208190 | 4.385215 | C | 8.240410 | 0.078810 | 4.436565 |
| C | 22.162560 | 13.007190 | 4.369265 | C | 9.483733 | 0.741297 | 4.433590 |
| C | 23.552010 | 13.013400 | 4.360890 | C | 9.445563 | 2.149516 | 4.439190 |
| C | 24.242000 | 14.220640 | 4.365815 | C | 8.248036 | 2.850402 | 4.439315 |
| O | 21.694180 | 11.633770 | 4.362940 | H | 6.083800 | 0.223690 | 4.436315 |
| O | 24.032960 | 11.644080 | 4.350690 | H | 17.384560 | 14.853810 | 4.435140 |
| B | 22.867020 | 10.870470 | 4.353490 | H | 10.394080 | 2.711476 | 4.439790 |
| C | 4.530130 | 6.518497 | 4.364390 | H | 8.257185 | 3.954286 | 4.440115 |
| C | 5.732267 | 7.243469 | 4.376640 | C | 19.118140 | 2.872123 | 4.347440 |
| C | 5.732590 | 8.631238 | 4.370015 | C | 17.916460 | 2.177889 | 4.348615 |
| C | 22.869360 | 9.374136 | 4.351940 | C | 17.870900 | 0.769892 | 4.364665 |
| C | 21.669960 | 8.635541 | 4.337915 | C | 0.777600 | 0.101590 | 4.377490 |
| C | 21.664410 | 7.247979 | 4.343215 | C | 1.981700 | 0.791215 | 4.377890 |
| H | 6.696674 | 9.165969 | 4.381465 | H | 19.114750 | 3.975926 | 4.334990 |
| H | 20.708460 | 9.174765 | 4.322465 | H | 16.971490 | 2.745660 | 4.337015 |
| O | 25.665990 | 14.500770 | 4.359390 | H | 9.960231 | 14.876530 | 4.390415 |
| O | 15.322640 | 0.644270 | 4.381190 | H | 2.934410 | 0.233560 | 4.390515 |
| **c** | 0.00000 | 0.00000 | 6.81948 | serrated zigzag | | | |

## Layer II    serrated armchair

| | | | | | | | |
|---|---|---|---|---|---|---|---|
| C | 3.459869 | 2.222231 | 4.377435 | B | 25.908580 | 15.191630 | 4.371660 |
| C | 5.893137 | 3.615478 | 4.405160 | O | 20.201590 | 13.769170 | 4.405010 |
| C | 5.906952 | 2.213752 | 4.417660 | B | 20.113770 | 15.165580 | 4.420785 |
| C | 4.680999 | 1.533999 | 4.403235 | O | 21.361240 | 15.799920 | 4.414535 |
| C | 2.180520 | 1.488225 | 4.368085 | H | 6.852869 | 4.164466 | 4.415560 |
| C | 3.484802 | 3.623877 | 4.365560 | H | 6.855656 | 5.986796 | 4.396260 |
| C | 4.691715 | 4.337306 | 4.379035 | H | 20.871840 | 5.994481 | 4.335610 |
| C | 7.181607 | 1.472347 | 4.436710 | H | 2.529870 | 4.180899 | 4.345410 |
| C | 22.317660 | 14.708090 | 4.394235 | H | 4.677287 | 0.428162 | 4.412135 |
| C | 23.708560 | 14.714420 | 4.383160 | C | 7.206449 | 0.068796 | 4.440510 |
| C | 21.628310 | 13.501680 | 4.388635 | C | 17.573530 | 15.249570 | 4.439985 |
| C | 22.328930 | 12.300680 | 4.372685 | C | 9.650103 | 0.034787 | 4.437010 |
| C | 23.718380 | 12.306890 | 4.364310 | C | 9.611933 | 1.443006 | 4.442610 |
| C | 24.408370 | 13.514130 | 4.369235 | C | 8.414407 | 2.143892 | 4.442735 |
| O | 21.860550 | 10.927260 | 4.366360 | H | 15.416920 | 15.394450 | 4.439735 |
| O | 24.199330 | 10.937570 | 4.354110 | H | 17.550930 | 14.147300 | 4.438560 |
| B | 23.033390 | 10.163960 | 4.356910 | H | 10.560450 | 2.004966 | 4.443210 |
| C | 4.696500 | 5.811987 | 4.367810 | H | 8.423555 | 3.247776 | 4.443535 |
| C | 5.898637 | 6.536959 | 4.380060 | C | 19.284510 | 2.165613 | 4.350860 |
| C | 5.898960 | 7.924728 | 4.373435 | C | 18.082830 | 1.471379 | 4.352035 |
| C | 23.035730 | 8.667626 | 4.355360 | C | 18.037270 | 0.063382 | 4.368085 |

| | | | | | | | |
|---|---|---|---|---|---|---|---|
| C | 21.836330 | 7.929031 | 4.341335 | C | 10.110720 | 15.272350 | 4.380910 |
| C | 21.830780 | 6.541469 | 4.346635 | C | 2.148070 | 0.084705 | 4.381310 |
| H | 6.863044 | 8.459459 | 4.384885 | H | 19.281120 | 3.269416 | 4.338410 |
| H | 20.874830 | 8.468255 | 4.325885 | H | 17.137860 | 2.039150 | 4.340435 |
| O | 25.832360 | 13.794260 | 4.362810 | H | 10.126600 | 14.170020 | 4.393835 |
| O | 24.655760 | 15.815030 | 4.384610 | H | 12.267530 | 15.404320 | 4.393935 |

**c** 0.00000 0.00000 6.82632 serrated armchair

## COF-7M

### Layer I AA, AB, inclined zigzag & armchair, and serrated zigzag & armchair

| | | | | | | | |
|---|---|---|---|---|---|---|---|
| C | 2.089298 | 3.482910 | 0.8291060 | C | 9.432521 | 4.695974 | 0.8271560 |
| C | 3.321002 | 4.155495 | 0.8423260 | C | 8.043071 | 4.695796 | 0.8329760 |
| C | 4.552149 | 3.481775 | 0.8406360 | O | 9.906851 | 8.471457 | 0.8017060 |
| C | 4.518664 | 2.078683 | 0.8270560 | O | 11.54991 | 5.626999 | 0.8141760 |
| C | 3.319577 | 1.349452 | 0.8149560 | O | 7.095810 | 3.599552 | 0.8441360 |
| C | 2.121119 | 2.079809 | 0.8155560 | O | 5.925208 | 5.625911 | 0.8349760 |
| H | 5.473324 | 1.526728 | 0.8256260 | O | 10.38063 | 3.600256 | 0.8340060 |
| H | 1.165733 | 1.529205 | 0.8037860 | O | 7.566726 | 8.470697 | 0.8124060 |
| C | 7.348151 | 5.898857 | 0.8271560 | B | 8.736531 | 9.236634 | 0.8072560 |
| C | 8.042571 | 7.102210 | 0.8149560 | B | 5.847724 | 4.229781 | 0.8428760 |
| C | 9.432001 | 7.102548 | 0.8085560 | B | 11.62851 | 4.231011 | 0.8263060 |
| C | 10.12681 | 5.899374 | 0.8149560 | H | 3.321609 | 5.258204 | 0.8550860 |

Transformation Matrix

| | | | | |
|---|---|---|---|---|
| **a** | 10.834740 | 0.000000 | 0.000000 | |
| **b** | 5.417370 | 9.383160 | 0.000000 | |
| **c** | 0.000000 | 0.000000 | 3.495390 | AA |
| **c** | 1.198785 | 0.692119 | 3.443941 | inclined zigzag |
| **c** | 1.389345 | 0.000000 | 3.402900 | inclined armchair |

### Layer II AB stacking

| | | | | | | | |
|---|---|---|---|---|---|---|---|
| C | 7.506668 | 6.610630 | 4.132246 | C | 14.84989 | 7.823694 | 4.130296 |
| C | 8.738372 | 7.283215 | 4.145466 | C | 13.46044 | 7.823516 | 4.136116 |
| C | 9.969519 | 6.609495 | 4.143776 | O | 15.32422 | 11.59918 | 4.104846 |
| C | 9.936033 | 5.206403 | 4.130196 | O | 16.96728 | 8.754719 | 4.117316 |
| C | 8.736947 | 4.477172 | 4.118096 | O | 12.51318 | 6.727272 | 4.147276 |
| C | 7.538489 | 5.207529 | 4.118696 | O | 11.34258 | 8.753632 | 4.138116 |
| H | 10.89069 | 4.654448 | 4.128766 | O | 15.79800 | 6.727976 | 4.137146 |
| H | 6.583103 | 4.656925 | 4.106926 | O | 12.98410 | 11.59842 | 4.115546 |
| C | 12.76552 | 9.026577 | 4.130296 | B | 14.15390 | 12.36435 | 4.110396 |
| C | 13.45994 | 10.22993 | 4.118096 | B | 11.26509 | 7.357501 | 4.146016 |
| C | 14.84937 | 10.23027 | 4.111696 | B | 17.04588 | 7.358731 | 4.129446 |
| C | 15.54418 | 9.027094 | 4.118096 | H | 8.738979 | 8.385923 | 4.158226 |

**c** 0.00000 0.00000 6.60628 AB

### Layer II serrated zigzag

| | | | | | | | |
|---|---|---|---|---|---|---|---|
| C | 3.288083 | 4.175029 | 4.26278 | C | 10.631310 | 5.388093 | 4.26083 |
| C | 4.519787 | 4.847614 | 4.27600 | C | 9.241856 | 5.387915 | 4.26665 |
| C | 5.750934 | 4.173894 | 4.27431 | O | 11.105640 | 9.163575 | 4.23538 |
| C | 5.717449 | 2.770802 | 4.26073 | O | 12.748690 | 6.319118 | 4.24785 |
| C | 4.518362 | 2.041571 | 4.24863 | O | 8.294595 | 4.291671 | 4.27781 |
| C | 3.319904 | 2.771928 | 4.24923 | O | 7.123993 | 6.318030 | 4.26865 |
| H | 6.672109 | 2.218847 | 4.25930 | O | 11.579410 | 4.292375 | 4.26768 |

| | | | | | | | |
|---|---|---|---|---|---|---|---|
| H | 2.364518 | 2.221324 | 4.23746 | O | 8.765512 | 9.162816 | 4.24608 |
| C | 8.546936 | 6.590976 | 4.26083 | B | 4.517946 | 0.545593 | 4.24093 |
| C | 9.241356 | 7.794329 | 4.24863 | B | 7.046509 | 4.921900 | 4.27655 |
| C | 10.630790 | 7.794667 | 4.24223 | B | 12.827290 | 4.923130 | 4.25998 |
| C | 11.325590 | 6.591493 | 4.24863 | H | 4.520394 | 5.950323 | 4.28876 |
| **c** | 0.00000 | 0.00000 | 6.86736 | serrated zigzag | | | |

**Layer II serrated armchair**

| | | | | | | | |
|---|---|---|---|---|---|---|---|
| C | 3.478643 | 3.482910 | 4.26620 | C | 10.821870 | 4.695974 | 4.26425 |
| C | 4.710347 | 4.155495 | 4.27942 | C | 9.432416 | 4.695796 | 4.27007 |
| C | 5.941494 | 3.481775 | 4.27773 | O | 11.296200 | 8.471457 | 4.23880 |
| C | 5.908009 | 2.078683 | 4.26415 | O | 12.939250 | 5.626999 | 4.25127 |
| C | 4.708922 | 1.349452 | 4.25205 | O | 8.485155 | 3.599552 | 4.28123 |
| C | 3.510464 | 2.079809 | 4.25265 | O | 7.314553 | 5.625911 | 4.27207 |
| H | 6.862669 | 1.526728 | 4.26272 | O | 11.769970 | 3.600256 | 4.27110 |
| H | 2.555078 | 1.529205 | 4.24088 | O | 8.956071 | 8.470697 | 4.24950 |
| C | 8.737496 | 5.898857 | 4.26425 | B | 10.125880 | 9.236634 | 4.24435 |
| C | 9.431916 | 7.102210 | 4.25205 | B | 7.237069 | 4.229781 | 4.27997 |
| C | 10.821350 | 7.102548 | 4.24565 | B | 13.017850 | 4.231011 | 4.26340 |
| C | 11.516160 | 5.899374 | 4.25205 | H | 4.710954 | 5.258204 | 4.29218 |
| **c** | 0.00000 | 0.00000 | 6.87420 | serrated armchair | | | |

## TP COF-1M

### Layer I AA, AB, inclined zigzag & armchair, and serrated zigzag & armchair

| | | | | | | | | | | | |
|---|---|---|---|---|---|---|---|---|---|---|---|
| C | 41.93071 | 22.44135 | 0.9456780 | C | 29.81555 | 2.941544 | 0.9388030 | C | 24.50818 | 10.96620 | 1.062253 |
| C | 15.31735 | 23.84314 | 0.9692280 | C | 28.61193 | 3.666409 | 0.9219280 | C | 23.27786 | 10.28848 | 1.054703 |
| C | 15.29007 | 22.44251 | 0.9782280 | C | 27.40732 | 2.943154 | 0.9112030 | C | 22.05271 | 10.96706 | 1.057953 |
| C | 14.08640 | 21.71777 | 0.9667030 | C | 27.37994 | 1.542002 | 0.9168030 | C | 23.25423 | 14.53541 | 1.105878 |
| H | 40.97625 | 21.88984 | 0.9363030 | H | 30.76978 | 3.493206 | 0.9481530 | H | 25.44198 | 12.91995 | 1.090878 |
| C | 14.84988 | 15.69144 | 0.9781780 | H | 26.45344 | 3.495369 | 0.8969780 | H | 23.27742 | 9.186367 | 1.040328 |
| C | 15.52798 | 16.90401 | 0.9862280 | C | 2.022967 | 0.793766 | 0.9708530 | C | 20.79098 | 10.27623 | 1.043678 |
| C | 14.81742 | 18.09719 | 0.9812280 | H | 2.977441 | 1.344597 | 0.9803780 | H | 24.20850 | 15.08624 | 1.125428 |
| C | 13.46063 | 15.67280 | 0.9653030 | O | 31.47836 | 8.738125 | 0.8700030 | H | 22.07697 | 16.31894 | 1.110603 |
| C | 12.75065 | 16.86703 | 0.9621030 | O | 30.33164 | 10.77838 | 0.9177030 | H | 20.79141 | 9.174468 | 1.038578 |
| C | 13.42875 | 18.07931 | 0.9697780 | B | 31.57160 | 10.13326 | 0.8815030 | H | 18.65771 | 10.40866 | 1.022203 |
| O | 15.27568 | 19.47153 | 0.9842780 | O | 25.85447 | 8.808589 | 1.021228 | C | 9.952588 | 13.02103 | 0.8913030 |
| O | 12.93524 | 19.44270 | 0.9651780 | B | 25.79604 | 10.20572 | 1.035003 | C | 8.752552 | 12.29745 | 0.8716030 |
| B | 14.09600 | 20.22198 | 0.9731280 | O | 27.05160 | 10.81994 | 1.008928 | C | 7.516801 | 13.00546 | 0.8795530 |
| C | 16.54692 | 24.58981 | 0.9810280 | O | 16.95549 | 16.65313 | 1.001678 | C | 36.56987 | 14.42960 | 0.9020530 |
| H | 17.50093 | 24.03838 | 0.9986280 | O | 15.81476 | 14.60929 | 0.9879030 | C | 8.748195 | 15.10579 | 0.9199530 |
| C | 41.90369 | 23.84248 | 0.9354780 | O | 12.52497 | 14.56424 | 0.9505280 | H | 10.90689 | 12.46984 | 0.8863780 |
| C | 43.13467 | 24.55867 | 0.9472530 | O | 11.33105 | 16.57718 | 0.9465530 | C | 8.718841 | 10.85926 | 0.8442280 |
| C | 40.67483 | 24.59019 | 0.9128780 | B | 17.05346 | 15.25841 | 1.003378 | C | 35.33230 | 12.29438 | 0.8677530 |
| H | 39.72037 | 24.03938 | 0.9025780 | B | 11.26967 | 15.18058 | 0.9388280 | C | 35.30775 | 15.12076 | 0.9061530 |
| H | 16.24449 | 21.89103 | 0.9948280 | C | 18.35656 | 14.52386 | 1.023653 | H | 37.79769 | 16.20793 | 0.9384780 |
| C | 26.15078 | 0.794345 | 0.9041030 | C | 9.978078 | 14.42570 | 0.9171530 | C | 34.09654 | 13.00241 | 0.8798280 |
| H | 25.19615 | 1.344748 | 0.8867780 | C | 18.38367 | 13.11909 | 1.020153 | C | 32.89714 | 12.27868 | 0.8793780 |
| C | 27.98384 | 9.710557 | 0.9723030 | C | 19.58266 | 12.39556 | 1.038228 | C | 32.87125 | 10.87385 | 0.8659280 |
| C | 29.37176 | 9.693224 | 0.9327780 | C | 20.81815 | 13.10377 | 1.060628 | C | 34.10009 | 10.19311 | 0.8474030 |
| C | 27.27371 | 8.516394 | 0.9804280 | C | 20.81289 | 14.52804 | 1.069503 | C | 6.278407 | 10.87041 | 0.8480030 |
| C | 27.95286 | 7.304898 | 0.9506780 | C | 19.58573 | 15.20523 | 1.050303 | C | 34.12928 | 14.44057 | 0.8957780 |
| C | 29.34174 | 7.288068 | 0.9140030 | H | 17.42910 | 12.56838 | 1.001678 | H | 31.94200 | 12.82916 | 0.8929530 |
| C | 30.05164 | 8.482030 | 0.9040530 | C | 19.61335 | 10.95752 | 1.034203 | H | 34.09911 | 9.090973 | 0.8358030 |
| O | 27.46063 | 5.940952 | 0.9541280 | C | 22.05026 | 12.39115 | 1.070653 | C | 7.539982 | 10.17945 | 0.8329030 |
| O | 29.80042 | 5.913657 | 0.8952280 | C | 22.07599 | 15.21701 | 1.097228 | H | 9.672662 | 10.30770 | 0.8328530 |
| B | 28.62153 | 5.162302 | 0.9218530 | H | 19.58529 | 16.30750 | 1.056078 | H | 7.538297 | 9.077640 | 0.8129530 |

| | | | | | | | | | | | |
|---|---|---|---|---|---|---|---|---|---|---|---|
| C | 28.61072 | 0.825489 | 0.9373280 | C | 23.28708 | 13.09705 | 1.088128 | H | 35.30721 | 16.22285 | 0.9187030 |
| C | 29.84224 | 1.540845 | 0.9485530 | C | 24.48596 | 12.37125 | 1.082478 | H | 33.17422 | 14.99055 | 0.9004530 |

Transformation Matrix

**a** 29.048470 0.000000 0.000000

**b** 14.524230 25.156700 0.000000

**c** 0.000000 0.000000 3.488000 AA

**c** 1.21743 0.70288 3.42821 inclined zigzag

**c** 1.38884 0.00000 3.431975 inclined armchair

## Layer II AB stacking

| | | | | | | | | | | | |
|---|---|---|---|---|---|---|---|---|---|---|---|
| C | 56.45494 | 30.82692 | 4.043016 | C | 44.33978 | 11.32712 | 4.036141 | C | 39.03242 | 19.35177 | 4.159591 |
| C | 29.84159 | 32.22871 | 4.066566 | C | 43.13617 | 12.05198 | 4.019266 | C | 37.80209 | 18.67405 | 4.152041 |
| C | 29.81430 | 30.82808 | 4.075566 | C | 41.93156 | 11.32873 | 4.008541 | C | 36.57694 | 19.35263 | 4.155291 |
| C | 28.61063 | 30.10334 | 4.064041 | C | 41.90417 | 9.927573 | 4.014141 | C | 37.77847 | 22.92098 | 4.203216 |
| H | 55.50048 | 30.27541 | 4.033641 | H | 45.29401 | 11.87878 | 4.045491 | H | 39.96622 | 21.30552 | 4.188216 |
| C | 29.37411 | 24.07701 | 4.075516 | H | 40.97768 | 11.88094 | 3.994316 | H | 37.80165 | 17.57194 | 4.137666 |
| C | 30.05222 | 25.28958 | 4.083566 | C | 16.54720 | 9.179338 | 4.068191 | C | 35.31522 | 18.66180 | 4.141016 |
| C | 29.34166 | 26.48276 | 4.078566 | H | 17.50168 | 9.730168 | 4.077716 | H | 38.73273 | 23.47181 | 4.222766 |
| C | 27.98487 | 24.05837 | 4.062641 | O | 46.00259 | 17.12370 | 3.967341 | H | 36.60120 | 24.70451 | 4.207941 |
| C | 27.27489 | 25.25260 | 4.059441 | O | 44.85587 | 19.16395 | 4.015041 | H | 35.31564 | 17.56004 | 4.135916 |
| C | 27.95298 | 26.46488 | 4.067116 | B | 46.09583 | 18.51883 | 3.978841 | H | 33.18195 | 18.79423 | 4.119541 |
| O | 29.79992 | 27.85710 | 4.081616 | O | 40.37870 | 17.19416 | 4.118566 | C | 24.47682 | 21.40660 | 3.988641 |
| O | 27.45947 | 27.82827 | 4.062516 | B | 40.32027 | 18.59129 | 4.132341 | C | 23.27679 | 20.68302 | 3.968941 |
| B | 28.62024 | 28.60755 | 4.070466 | O | 41.57584 | 19.20551 | 4.106266 | C | 22.04103 | 21.39103 | 3.976891 |
| C | 31.07116 | 32.97538 | 4.078366 | O | 31.47972 | 25.03870 | 4.099016 | C | 51.09410 | 22.81517 | 3.999391 |
| H | 32.02517 | 32.42395 | 4.095966 | O | 30.33899 | 22.99486 | 4.085241 | C | 23.27243 | 23.49136 | 4.017291 |
| C | 56.42793 | 32.22805 | 4.032816 | O | 27.04921 | 22.94981 | 4.047866 | H | 25.43113 | 20.85541 | 3.983716 |
| C | 57.65891 | 32.94424 | 4.044591 | O | 25.85529 | 24.96275 | 4.043891 | C | 23.24308 | 19.24483 | 3.941566 |
| C | 55.19907 | 32.97576 | 4.010216 | B | 31.57769 | 23.64398 | 4.100716 | C | 49.85653 | 20.67995 | 3.965091 |
| H | 54.24461 | 32.42495 | 3.999916 | B | 25.79390 | 23.56615 | 4.036166 | C | 49.83199 | 23.50633 | 4.003491 |
| H | 30.76872 | 30.27660 | 4.092166 | C | 32.88079 | 22.90943 | 4.120991 | H | 52.32193 | 24.59350 | 4.035816 |
| C | 40.67501 | 9.179916 | 4.001441 | C | 24.50231 | 22.81127 | 4.014491 | C | 48.62077 | 21.38798 | 3.977166 |
| H | 39.72038 | 9.730320 | 3.984116 | C | 32.90791 | 21.50466 | 4.117491 | C | 47.42138 | 20.66425 | 3.976716 |
| C | 42.50808 | 18.09613 | 4.069641 | C | 34.10690 | 20.78113 | 4.135566 | C | 47.39548 | 19.25942 | 3.963266 |
| C | 43.89600 | 18.07880 | 4.030116 | C | 35.34238 | 21.48934 | 4.157966 | C | 48.62432 | 18.57868 | 3.944741 |
| C | 41.79794 | 16.90197 | 4.077766 | C | 35.33712 | 22.91361 | 4.166841 | C | 20.80264 | 19.25598 | 3.945341 |
| C | 42.47710 | 15.69047 | 4.048016 | C | 34.10996 | 23.59080 | 4.147641 | C | 48.65351 | 22.82614 | 3.993116 |
| C | 43.86597 | 15.67364 | 4.011341 | H | 31.95333 | 20.95395 | 4.099016 | H | 46.46623 | 21.21473 | 3.990291 |
| C | 44.57587 | 16.86760 | 4.001391 | C | 34.13758 | 19.34309 | 4.131541 | H | 48.62334 | 17.47654 | 3.933141 |
| O | 41.98486 | 14.32652 | 4.051466 | C | 36.57449 | 20.77672 | 4.167991 | C | 22.06422 | 18.56502 | 3.930241 |
| O | 44.32465 | 14.29923 | 3.992566 | C | 36.60023 | 23.60258 | 4.194566 | H | 24.19690 | 18.69327 | 3.930191 |
| B | 43.14577 | 13.54787 | 4.019191 | H | 34.10953 | 24.69307 | 4.153416 | H | 22.06253 | 17.46321 | 3.910291 |
| C | 43.13496 | 9.211061 | 4.034666 | C | 37.81132 | 21.48262 | 4.185466 | H | 49.83144 | 24.60842 | 4.016041 |
| C | 44.36647 | 9.926416 | 4.045891 | C | 39.01019 | 20.75682 | 4.179816 | H | 47.69846 | 23.37612 | 3.997791 |

**c** 0.00000 0.00000 6.194675 AB

## Layer II serrated zigzag

| | | | | | | | | | | | |
|---|---|---|---|---|---|---|---|---|---|---|---|
| C | 14.099670 | 23.144230 | 4.365678 | C | 31.032980 | 3.644424 | 4.358803 | C | 25.725610 | 11.669080 | 4.482253 |
| C | 16.534780 | 24.546020 | 4.389228 | C | 29.829360 | 4.369289 | 4.341928 | C | 24.495290 | 10.991360 | 4.474703 |
| C | 16.507500 | 23.145390 | 4.398228 | C | 28.624750 | 3.646034 | 4.331203 | C | 23.270140 | 11.669940 | 4.477953 |
| C | 15.303830 | 22.420650 | 4.386703 | C | 28.597370 | 2.244882 | 4.336803 | C | 24.471660 | 15.238290 | 4.525878 |
| H | 13.145210 | 22.592720 | 4.356303 | H | 2.938740 | 4.196086 | 4.368153 | H | 26.659410 | 13.622830 | 4.510878 |
| C | 16.067310 | 16.394320 | 4.398178 | H | 27.670870 | 4.198249 | 4.316978 | H | 24.494850 | 9.889247 | 4.460328 |
| C | 16.745410 | 17.606890 | 4.406228 | C | 3.240397 | 1.496646 | 4.390853 | C | 22.008410 | 10.979110 | 4.463678 |
| C | 16.034850 | 18.800070 | 4.401228 | H | 4.194871 | 2.047477 | 4.400378 | H | 25.425930 | 15.789120 | 4.545428 |
| C | 14.678060 | 16.375680 | 4.385303 | O | 32.695790 | 9.441005 | 4.290003 | H | 23.294400 | 17.021820 | 4.530603 |
| C | 13.968080 | 17.569910 | 4.382103 | O | 31.549070 | 11.481260 | 4.337703 | H | 22.008840 | 9.877348 | 4.458578 |
| C | 14.646180 | 18.782190 | 4.389778 | B | 32.789030 | 10.836140 | 4.301503 | H | 19.875140 | 11.111540 | 4.442203 |
| O | 16.493110 | 20.174410 | 4.404278 | O | 27.071900 | 9.511469 | 4.441228 | C | 11.170020 | 13.723910 | 4.311303 |
| O | 14.152670 | 20.145580 | 4.385178 | B | 27.013470 | 10.908600 | 4.455003 | C | 9.969982 | 13.000330 | 4.291603 |
| B | 15.313430 | 20.924860 | 4.393128 | O | 28.269030 | 11.522820 | 4.428928 | C | 8.734231 | 13.708340 | 4.299553 |

| | | | | | | | | | | | |
|---|---|---|---|---|---|---|---|---|---|---|---|
| C | 3.240120 | 0.135990 | 4.401028 | O | 18.172920 | 17.356010 | 4.421678 | C | 8.738830 | 15.132480 | 4.322053 |
| H | 18.718360 | 24.741260 | 4.418628 | O | 17.032190 | 15.312170 | 4.407903 | C | 9.965625 | 15.808670 | 4.339953 |
| C | 43.121120 | 24.545360 | 4.355478 | O | 13.742400 | 15.267120 | 4.370528 | H | 12.124320 | 13.172720 | 4.306378 |
| C | 0.779400 | 0.104850 | 4.367253 | O | 12.548480 | 17.280060 | 4.366553 | C | 9.936271 | 11.562140 | 4.264228 |
| C | 27.368030 | 0.136370 | 4.332878 | B | 18.270890 | 15.961290 | 4.423378 | C | 36.549730 | 12.997260 | 4.287753 |
| H | 40.937800 | 24.742260 | 4.322578 | B | 12.487100 | 15.883460 | 4.358828 | C | 36.525180 | 15.823640 | 4.326153 |
| H | 17.461920 | 22.593910 | 4.414828 | C | 19.573990 | 15.226740 | 4.443653 | H | 9.966650 | 16.910810 | 4.358478 |
| C | 27.368210 | 1.497225 | 4.324103 | C | 11.195510 | 15.128580 | 4.337153 | C | 35.313970 | 13.705290 | 4.299828 |
| H | 26.413580 | 2.047628 | 4.306778 | C | 19.601100 | 13.821970 | 4.440153 | C | 34.114570 | 12.981560 | 4.299378 |
| C | 29.201270 | 10.413440 | 4.392303 | C | 20.800090 | 13.098440 | 4.458228 | C | 34.088680 | 11.576730 | 4.285928 |
| C | 30.589190 | 10.396100 | 4.352778 | C | 22.035580 | 13.806650 | 4.480628 | C | 35.317520 | 10.895990 | 4.267403 |
| C | 28.491140 | 9.219274 | 4.400428 | C | 22.030320 | 15.230920 | 4.489503 | C | 7.495837 | 11.573290 | 4.268003 |
| C | 29.170290 | 8.007778 | 4.370678 | C | 20.803160 | 15.908110 | 4.470303 | C | 35.346710 | 15.143450 | 4.315778 |
| C | 30.559170 | 7.990948 | 4.334003 | H | 18.646530 | 13.271260 | 4.421678 | H | 33.159430 | 13.532040 | 4.312953 |
| C | 31.269070 | 9.184910 | 4.324053 | C | 20.830780 | 11.660400 | 4.454203 | H | 6.268070 | 9.793853 | 4.255803 |
| O | 28.678060 | 6.643832 | 4.374128 | C | 23.267690 | 13.094030 | 4.490653 | C | 8.757412 | 10.882330 | 4.252903 |
| O | 31.017850 | 6.616537 | 4.315228 | C | 23.293420 | 15.919890 | 4.517228 | H | 10.890090 | 11.010580 | 4.252853 |
| B | 29.838960 | 5.865182 | 4.341853 | H | 20.802720 | 17.010380 | 4.476078 | H | 8.755727 | 9.780519 | 4.232953 |
| C | 29.828150 | 1.528369 | 4.357328 | C | 24.504510 | 13.799930 | 4.508128 | H | 36.524640 | 16.925730 | 4.338703 |
| C | 2.011200 | 2.243725 | 4.368553 | C | 25.703390 | 13.074130 | 4.502478 | H | 34.391650 | 15.693430 | 4.320453 |

**c** 0.00000 0.00000 6.84000 serrated zigzag

## Layer II serrated armchair

| | | | | | | | | | | | |
|---|---|---|---|---|---|---|---|---|---|---|---|
| C | 14.271080 | 22.441350 | 4.372518 | C | 2.155920 | 2.941544 | 4.365643 | C | 25.897020 | 10.966200 | 4.489093 |
| C | 16.706190 | 23.843140 | 4.396068 | C | 30.000770 | 3.666409 | 4.348768 | C | 24.666700 | 10.288480 | 4.481543 |
| C | 16.678910 | 22.442510 | 4.405068 | C | 28.796160 | 2.943154 | 4.338043 | C | 23.441550 | 10.967060 | 4.484793 |
| C | 15.475240 | 21.717770 | 4.393543 | C | 28.768780 | 1.542002 | 4.343643 | C | 24.643070 | 14.535410 | 4.532718 |
| H | 13.316620 | 21.889840 | 4.363143 | H | 3.110150 | 3.493206 | 4.374993 | H | 26.830820 | 12.919950 | 4.517718 |
| C | 16.238720 | 15.691440 | 4.405018 | H | 27.842280 | 3.495369 | 4.323818 | H | 24.666260 | 9.186367 | 4.467168 |
| C | 16.916820 | 16.904010 | 4.413068 | C | 3.411807 | 0.793766 | 4.397693 | C | 22.179820 | 10.276230 | 4.470518 |
| C | 16.206260 | 18.097190 | 4.408068 | H | 4.366281 | 1.344597 | 4.407218 | H | 25.597340 | 15.086240 | 4.552268 |
| C | 14.849470 | 15.672800 | 4.392143 | O | 32.867200 | 8.738125 | 4.296843 | H | 23.465810 | 16.318940 | 4.537443 |
| C | 14.139490 | 16.867030 | 4.388943 | O | 31.720480 | 10.778380 | 4.344543 | H | 22.180250 | 9.174468 | 4.465418 |
| C | 14.817590 | 18.079310 | 4.396618 | B | 32.960440 | 10.133260 | 4.308343 | H | 20.046550 | 10.408660 | 4.449043 |
| O | 16.664520 | 19.471530 | 4.411118 | O | 27.243310 | 8.808589 | 4.448068 | C | 11.341430 | 13.021030 | 4.318143 |
| O | 14.324080 | 19.442700 | 4.392018 | B | 27.184880 | 10.205720 | 4.461843 | C | 10.141390 | 12.297450 | 4.298443 |
| B | 15.484840 | 20.221980 | 4.399968 | O | 28.440440 | 10.819940 | 4.435768 | C | 8.905641 | 13.005460 | 4.306393 |
| C | 17.935760 | 24.589810 | 4.407868 | O | 18.344330 | 16.653130 | 4.428518 | C | 8.910240 | 14.429600 | 4.328893 |
| H | 18.889770 | 24.038380 | 4.425468 | O | 17.203600 | 14.609290 | 4.414743 | C | 10.137030 | 15.105790 | 4.346793 |
| C | 14.244060 | 23.842480 | 4.362318 | O | 13.913810 | 14.564240 | 4.377368 | H | 12.295730 | 12.469840 | 4.313218 |
| C | 15.475040 | 24.558670 | 4.374093 | O | 12.719890 | 16.577180 | 4.373393 | C | 10.107680 | 10.859260 | 4.271068 |
| C | 42.063670 | 24.590190 | 4.339718 | B | 18.442300 | 15.258410 | 4.430218 | C | 7.672670 | 12.294380 | 4.294593 |
| H | 41.109210 | 24.039380 | 4.329418 | B | 12.658510 | 15.180580 | 4.365668 | C | 36.696590 | 15.120760 | 4.332993 |
| H | 17.633330 | 21.891030 | 4.421668 | C | 19.745400 | 14.523860 | 4.450493 | H | 10.138060 | 16.207930 | 4.365318 |
| C | 27.539620 | 0.794345 | 4.330943 | C | 11.366920 | 14.425700 | 4.343993 | C | 35.485380 | 13.002410 | 4.306668 |
| H | 26.584990 | 1.344748 | 4.313618 | C | 19.772510 | 13.119090 | 4.446993 | C | 34.285980 | 12.278680 | 4.306218 |
| C | 29.372680 | 9.710557 | 4.399143 | C | 20.971500 | 12.395560 | 4.465068 | C | 34.260090 | 10.873850 | 4.292768 |
| C | 30.760600 | 9.693224 | 4.359618 | C | 22.206990 | 13.103770 | 4.487468 | C | 6.440460 | 10.193110 | 4.274243 |
| C | 28.662550 | 8.516394 | 4.407268 | C | 22.201730 | 14.528040 | 4.496343 | C | 7.667247 | 10.870410 | 4.274843 |
| C | 29.341700 | 7.304898 | 4.377518 | C | 20.974570 | 15.205230 | 4.477143 | C | 35.518120 | 14.440570 | 4.322618 |
| C | 30.730580 | 7.288068 | 4.340843 | H | 18.817940 | 12.568380 | 4.428518 | H | 33.330840 | 12.829160 | 4.319793 |
| C | 31.440480 | 8.482030 | 4.330893 | C | 21.002190 | 10.957520 | 4.461043 | H | 6.439480 | 9.090973 | 4.262643 |
| O | 28.849470 | 5.940952 | 4.380968 | C | 23.439100 | 12.391150 | 4.497493 | C | 8.928822 | 10.179450 | 4.259743 |
| O | 31.189260 | 5.913657 | 4.322068 | C | 23.464830 | 15.217010 | 4.524068 | H | 11.061500 | 10.307700 | 4.259693 |
| B | 30.010370 | 5.162302 | 4.348693 | H | 20.974130 | 16.307500 | 4.482918 | H | 8.927137 | 9.077640 | 4.239793 |
| C | 0.951090 | 0.825489 | 4.364168 | C | 24.675920 | 13.097050 | 4.514968 | H | 36.696050 | 16.222850 | 4.345543 |
| C | 2.182610 | 1.540845 | 4.375393 | C | 25.874800 | 12.371250 | 4.509318 | H | 34.563060 | 14.990550 | 4.327293 |

**c** 0.00000 0.00000 6.85368 serrated armchair

# COF-8M

## Layer I    AA, AB, inclined zigzag & armchair, and serrated zigzag & armchair

| | | | | | | | | | | | |
|---|---|---|---|---|---|---|---|---|---|---|---|
| C | 14.87100 | 13.10092 | 12.53652 | C | 9.994664 | 10.25464 | 12.48675 | B | 4.657629 | 8.066248 | 12.49980 |
| C | 15.60762 | 14.29931 | 12.54615 | C | 10.05393 | 11.66229 | 12.50093 | B | 13.39624 | 2.998720 | 12.47470 |
| C | 16.99475 | 14.29966 | 12.54198 | C | 6.314614 | 9.571654 | 12.48913 | H | 17.53230 | 10.94041 | 12.51272 |
| C | 17.73172 | 13.10140 | 12.52777 | O | 9.075429 | 8.150852 | 12.46467 | H | 8.793394 | 0.127528 | 12.54862 |
| C | 16.99497 | 11.90296 | 12.52248 | O | 11.09507 | 9.318142 | 12.47698 | C | 19.28621 | 5.530660 | 12.52065 |
| C | 15.60790 | 11.90277 | 12.52615 | C | 11.24319 | 12.41787 | 12.51398 | C | 19.97979 | 6.731958 | 12.51320 |
| H | 6.332169 | 0.126983 | 12.55585 | C | 22.54893 | 11.66186 | 12.49982 | C | 3.909664 | 6.770794 | 12.50930 |
| H | 15.07095 | 10.94000 | 12.51947 | C | 21.35955 | 12.41766 | 12.50475 | C | 4.579064 | 5.533536 | 12.51765 |
| C | 25.03966 | 14.53985 | 12.50580 | C | 21.35980 | 13.78255 | 12.50263 | C | 3.885240 | 4.332313 | 12.52560 |
| C | 16.30157 | 0.840782 | 12.50650 | C | 22.54915 | 14.53802 | 12.49840 | C | 2.479138 | 4.293537 | 12.52580 |
| C | 17.55042 | 1.493011 | 12.51538 | C | 22.60887 | 10.25408 | 12.49687 | H | 19.41463 | 7.678579 | 12.50865 |
| C | 1.255848 | 0.810617 | 12.52420 | O | 19.99767 | 11.93435 | 12.51305 | H | 5.681329 | 5.517084 | 12.51720 |
| C | 10.05366 | 14.53797 | 12.52137 | O | 19.99862 | 14.26709 | 12.50978 | H | 4.450455 | 3.385723 | 12.53092 |
| C | 15.05264 | 1.493086 | 12.49675 | C | 13.87068 | 0.810799 | 12.49273 | H | 18.18408 | 5.547112 | 12.52210 |
| O | 17.81232 | 2.914312 | 12.51670 | B | 1.731129 | 2.998160 | 12.52627 | C | 11.24289 | 4.333766 | 12.44150 |
| O | 2.355929 | 1.747764 | 12.53140 | B | 10.47093 | 8.067413 | 12.46348 | C | 10.54974 | 5.535216 | 12.43835 |
| C | 11.24303 | 13.78254 | 12.52532 | B | 19.22758 | 13.10107 | 12.51723 | C | 11.21933 | 6.772308 | 12.45425 |
| C | 23.79268 | 13.81988 | 12.50110 | O | 12.60461 | 14.26672 | 12.53745 | C | 12.62550 | 6.732987 | 12.46800 |
| C | 23.79267 | 12.37991 | 12.49920 | O | 12.60503 | 11.93408 | 12.51793 | C | 13.31859 | 5.531432 | 12.47097 |
| C | 7.563324 | 11.65993 | 12.49578 | O | 21.50914 | 9.316689 | 12.50257 | C | 12.64889 | 4.294324 | 12.46085 |
| C | 8.810241 | 12.38004 | 12.50228 | O | 6.052922 | 8.150474 | 12.48980 | H | 9.447520 | 5.519505 | 12.42425 |
| C | 8.810172 | 13.81989 | 12.51038 | O | 14.79147 | 2.914312 | 12.48668 | H | 13.19118 | 7.679184 | 12.47850 |
| C | 7.563577 | 10.22387 | 12.48703 | O | 12.77095 | 1.748475 | 12.47910 | H | 14.42101 | 5.547339 | 12.48378 |
| C | 8.812889 | 9.571866 | 12.47962 | B | 13.37519 | 13.10052 | 12.53230 | H | 10.67745 | 3.387569 | 12.42988 |

Transformation Matrix

| | | | | |
|---|---|---|---|---|
| **a** | 17.476440 | 0.000000 | 0.000000 | |
| **b** | 8.738220 | 15.135040 | 0.000000 | |
| **c** | 0.000000 | 0.000000 | 3.472735 | AA stacking |
| **c** | 1.384650 | 0.000000 | 3.416581 | inclined zigzag |
| **c** | 1.219300 | 0.703963 | 3.419316 | inclined armchair |

## Layer II   AB stacking

| | | | | | | | | | | | |
|---|---|---|---|---|---|---|---|---|---|---|---|
| C | 23.60922 | 18.14593 | 15.82520 | C | 18.73288 | 15.29965 | 15.77542 | B | 13.39585 | 13.11126 | 15.78848 |
| C | 24.34584 | 19.34432 | 15.83483 | C | 18.79215 | 16.70730 | 15.78961 | B | 22.13446 | 8.043734 | 15.76338 |
| C | 25.73297 | 19.34467 | 15.83066 | C | 15.05283 | 14.61667 | 15.77781 | H | 26.27052 | 15.98542 | 15.80140 |
| C | 26.46994 | 18.14641 | 15.81645 | O | 17.81365 | 13.19587 | 15.75335 | H | 17.53161 | 5.172542 | 15.83730 |
| C | 25.73319 | 16.94797 | 15.81116 | O | 19.83329 | 14.36316 | 15.76566 | C | 28.02443 | 10.57567 | 15.80933 |
| C | 24.34612 | 16.94778 | 15.81483 | C | 19.98141 | 17.46288 | 15.80266 | C | 28.71801 | 11.77697 | 15.80188 |
| H | 15.07039 | 5.171997 | 15.84453 | C | 31.28715 | 16.70687 | 15.78850 | C | 12.64788 | 11.81581 | 15.79798 |
| H | 23.80917 | 15.98501 | 15.80815 | C | 30.09777 | 17.46267 | 15.79343 | C | 13.31728 | 10.57855 | 15.80632 |
| C | 33.77788 | 19.58486 | 15.79448 | C | 30.09802 | 18.82756 | 15.79131 | C | 12.62346 | 9.377327 | 15.81428 |
| C | 25.03979 | 5.885796 | 15.79518 | C | 31.28737 | 19.58303 | 15.78708 | C | 11.21736 | 9.338551 | 15.81448 |
| C | 26.28864 | 6.538025 | 15.80406 | C | 31.34709 | 15.29909 | 15.78555 | H | 28.15285 | 12.72359 | 15.79733 |
| C | 9.994068 | 5.855631 | 15.81288 | O | 28.73589 | 16.97936 | 15.80173 | H | 14.41955 | 10.56210 | 15.80588 |
| C | 18.79188 | 19.58298 | 15.81005 | O | 28.73684 | 19.31210 | 15.79846 | H | 13.18867 | 8.430737 | 15.81960 |
| C | 23.79086 | 6.538100 | 15.78543 | C | 22.60890 | 5.855813 | 15.78141 | H | 26.92230 | 10.59213 | 15.81078 |
| O | 26.55054 | 7.959326 | 15.80538 | B | 10.46935 | 8.043174 | 15.81495 | C | 19.98111 | 9.378780 | 15.73018 |
| O | 11.09415 | 6.792778 | 15.82008 | B | 19.20915 | 13.11243 | 15.75216 | C | 19.28796 | 10.58023 | 15.72703 |
| C | 19.98125 | 18.82755 | 15.81400 | B | 27.96580 | 18.14608 | 15.80591 | C | 19.95755 | 11.81732 | 15.74293 |
| C | 32.53090 | 18.86489 | 15.78977 | O | 21.34283 | 19.31173 | 15.82613 | C | 21.36372 | 11.77800 | 15.75668 |
| C | 32.53089 | 17.42492 | 15.78788 | O | 21.34325 | 16.97909 | 15.80661 | C | 22.05681 | 10.57645 | 15.75965 |
| C | 16.30154 | 16.70494 | 15.78446 | O | 30.24736 | 14.36170 | 15.79125 | C | 21.38711 | 9.339338 | 15.74953 |
| C | 17.54846 | 17.42505 | 15.79096 | O | 14.79114 | 13.19549 | 15.77848 | H | 18.18574 | 10.56452 | 15.71292 |
| C | 17.54839 | 18.86490 | 15.79906 | O | 23.52969 | 7.959326 | 15.77536 | H | 21.92940 | 12.72420 | 15.76718 |
| C | 16.30180 | 15.26888 | 15.77571 | O | 21.50917 | 6.793489 | 15.76778 | H | 23.15923 | 10.59235 | 15.77246 |
| C | 17.55111 | 14.61688 | 15.76830 | B | 22.11341 | 18.14553 | 15.82098 | H | 19.41567 | 8.432583 | 15.71856 |

| | | | | |
|---|---|---|---|---|
| **c** | 0.00000 | 0.00000 | 13.15470 | AB |

## Layer II serrated zigzag

| | | | | | | | | | | | |
|---|---|---|---|---|---|---|---|---|---|---|---|
| C | 16.09030 | 13.80488 | 15.94626 | C | 11.21396 | 10.95860 | 15.89649 | B | 5.876929 | 8.770211 | 15.90954 |
| C | 16.82692 | 15.00327 | 15.95589 | C | 11.27323 | 12.36625 | 15.91067 | B | 14.61554 | 3.702683 | 15.88444 |
| C | 18.21405 | 15.00362 | 15.95172 | C | 7.533914 | 10.27562 | 15.89887 | H | 18.75160 | 11.64437 | 15.92246 |
| C | 18.95102 | 13.80536 | 15.93751 | O | 10.29473 | 8.854815 | 15.87441 | H | 10.01269 | 0.831491 | 15.95836 |
| C | 18.21427 | 12.60692 | 15.93222 | O | 12.31437 | 10.02211 | 15.88672 | C | 20.50551 | 6.234623 | 15.93039 |
| C | 16.82720 | 12.60673 | 15.93589 | C | 12.46249 | 13.12183 | 15.92372 | C | 21.19909 | 7.435921 | 15.92294 |
| H | 7.551469 | 0.830946 | 15.96559 | C | 23.76823 | 12.36582 | 15.90956 | C | 5.128964 | 7.474757 | 15.91904 |
| H | 16.29025 | 11.64396 | 15.92921 | C | 22.57885 | 13.12162 | 15.91449 | C | 5.798364 | 6.237499 | 15.92739 |
| C | 26.25896 | 15.24381 | 15.91554 | C | 22.57910 | 14.48651 | 15.91237 | C | 5.104540 | 5.036276 | 15.93534 |
| C | 17.52087 | 1.544745 | 15.91624 | C | 23.76845 | 15.24198 | 15.90814 | C | 3.698438 | 4.997500 | 15.93554 |
| C | 18.76972 | 2.196974 | 15.92512 | C | 23.82817 | 10.95804 | 15.90661 | H | 20.63393 | 8.382542 | 15.91839 |
| C | 2.475148 | 1.514580 | 15.93394 | O | 21.21697 | 12.63831 | 15.92279 | H | 6.900629 | 6.221047 | 15.92694 |
| C | 11.27296 | 15.24193 | 15.93111 | O | 21.21792 | 14.97105 | 15.91952 | H | 5.669755 | 4.089686 | 15.94066 |
| C | 16.27194 | 2.197049 | 15.90649 | C | 15.08998 | 1.514762 | 15.90247 | H | 19.40338 | 6.251075 | 15.93184 |
| O | 19.03162 | 3.618275 | 15.92644 | B | 2.950429 | 3.702123 | 15.93601 | C | 12.46219 | 5.037729 | 15.85124 |
| O | 3.575229 | 2.451727 | 15.94114 | B | 11.69023 | 8.771377 | 15.87322 | C | 11.76904 | 6.239179 | 15.84809 |
| C | 12.46233 | 14.48650 | 15.93506 | B | 20.44688 | 13.80503 | 15.92697 | C | 12.43863 | 7.476271 | 15.86399 |
| C | 25.01198 | 14.52384 | 15.91084 | O | 13.82391 | 14.97068 | 15.94719 | C | 13.84480 | 7.436950 | 15.87774 |
| C | 25.01197 | 13.08387 | 15.90894 | O | 13.82433 | 12.63804 | 15.92767 | C | 14.53789 | 6.235395 | 15.88071 |
| C | 8.782624 | 12.36389 | 15.90552 | O | 22.72844 | 10.02065 | 15.91231 | C | 13.86819 | 4.998287 | 15.87059 |
| C | 10.02954 | 13.08400 | 15.91202 | O | 7.272222 | 8.854437 | 15.89954 | H | 10.66682 | 6.223468 | 15.83399 |
| C | 10.02947 | 14.52385 | 15.92012 | O | 16.01077 | 3.618275 | 15.89642 | H | 14.41048 | 8.383147 | 15.88824 |
| C | 8.782877 | 10.92783 | 15.89677 | O | 13.99025 | 2.452438 | 15.88884 | H | 15.64031 | 6.251302 | 15.89352 |
| C | 10.03219 | 10.27583 | 15.88936 | B | 14.59449 | 13.80448 | 15.94204 | H | 11.89675 | 4.091532 | 15.83962 |

**c** 0.00000 0.00000 6.81948 serrated zigzag

## Layer II serrated armchair

| | | | | | | | | | | | |
|---|---|---|---|---|---|---|---|---|---|---|---|
| C | 16.25565 | 13.10092 | 15.94626 | C | 11.37931 | 10.25464 | 15.89649 | B | 6.042279 | 8.066248 | 15.90954 |
| C | 16.99227 | 14.29931 | 15.95589 | C | 11.43858 | 11.66229 | 15.91067 | B | 14.78089 | 2.998720 | 15.88444 |
| C | 18.37940 | 14.29966 | 15.95172 | C | 7.699264 | 9.571654 | 15.89887 | H | 18.91695 | 10.94041 | 15.92246 |
| C | 19.11637 | 13.10140 | 15.93751 | O | 10.46008 | 8.150852 | 15.87441 | H | 10.17804 | 0.127528 | 15.95836 |
| C | 18.37962 | 11.90296 | 15.93222 | O | 12.47972 | 9.318142 | 15.88672 | C | 20.67086 | 5.530660 | 15.93039 |
| C | 16.99255 | 11.90277 | 15.93589 | C | 12.62784 | 12.41787 | 15.92372 | C | 21.36444 | 6.731958 | 15.92294 |
| H | 7.716819 | 0.126983 | 15.96559 | C | 23.93358 | 11.66186 | 15.90956 | C | 5.294314 | 6.770794 | 15.91904 |
| H | 16.45560 | 10.94000 | 15.92921 | C | 22.74420 | 12.41766 | 15.91449 | C | 5.963714 | 5.533536 | 15.92739 |
| C | 26.42431 | 14.53985 | 15.91554 | C | 22.74445 | 13.78255 | 15.91237 | C | 5.269890 | 4.332313 | 15.93534 |
| C | 17.68622 | 0.840782 | 15.91624 | C | 23.93380 | 14.53802 | 15.90814 | C | 3.863788 | 4.293537 | 15.93554 |
| C | 18.93507 | 1.493011 | 15.92512 | C | 23.99352 | 10.25408 | 15.90661 | H | 20.79928 | 7.678579 | 15.91839 |
| C | 2.640498 | 0.810617 | 15.93394 | O | 21.38232 | 11.93435 | 15.92279 | H | 7.065979 | 5.517084 | 15.92694 |
| C | 11.43831 | 14.53797 | 15.93111 | O | 21.38327 | 14.26709 | 15.91952 | H | 5.835105 | 3.385723 | 15.94066 |
| C | 16.43729 | 1.493086 | 15.90649 | C | 15.25533 | 0.810799 | 15.90247 | H | 19.56873 | 5.547112 | 15.93184 |
| O | 19.19697 | 2.914312 | 15.92644 | B | 3.115779 | 2.998160 | 15.93601 | C | 12.62754 | 4.333766 | 15.85124 |
| O | 3.740579 | 1.747764 | 15.94114 | B | 11.85558 | 8.067413 | 15.87322 | C | 11.93439 | 5.535216 | 15.84809 |
| C | 12.62768 | 13.78254 | 15.93506 | B | 20.61223 | 13.10107 | 15.92697 | C | 12.60398 | 6.772308 | 15.86399 |
| C | 25.17733 | 13.81988 | 15.91084 | O | 13.98926 | 14.26672 | 15.94719 | C | 14.01015 | 6.732987 | 15.87774 |
| C | 25.17732 | 12.37991 | 15.90894 | O | 13.98968 | 11.93408 | 15.92767 | C | 14.70324 | 5.531432 | 15.88071 |
| C | 8.947974 | 11.65993 | 15.90552 | O | 22.89379 | 9.316689 | 15.91231 | C | 14.03354 | 4.294324 | 15.87059 |
| C | 10.19489 | 12.38004 | 15.91202 | O | 7.437572 | 8.150474 | 15.89954 | H | 10.83217 | 5.519505 | 15.83399 |
| C | 10.19482 | 13.81989 | 15.92012 | O | 16.17612 | 2.914312 | 15.89642 | H | 14.57583 | 7.679184 | 15.88824 |
| C | 8.948227 | 10.22387 | 15.89677 | O | 14.15560 | 1.748475 | 15.88884 | H | 15.80566 | 5.547339 | 15.89352 |
| C | 10.19754 | 9.571866 | 15.88936 | B | 14.75984 | 13.10052 | 15.94204 | H | 12.06210 | 3.387569 | 15.83962 |

**c** 0.00000 0.00000 6.81948 serrated armchair

## COF-9M

### Layer I AA, AB, inclined zigzag & armchair, and serrated zigzag & armchair

| | | | | | | | | | | | |
|---|---|---|---|---|---|---|---|---|---|---|---|
| C | 14.59178 | 3.372689 | 0.8041910 | C | 11.20542 | 13.01756 | 0.9207410 | H | 22.76201 | 6.737653 | 0.9634910 |
| C | 13.89448 | 4.570645 | 0.8000910 | C | 11.26492 | 14.42487 | 0.9335410 | H | 4.741983 | 4.584498 | 1.050616 |
| C | 14.57390 | 5.799003 | 0.8085410 | C | 7.526185 | 12.33444 | 0.9405160 | H | 3.509206 | 2.435057 | 1.030366 |
| C | 15.97742 | 5.776763 | 0.8152160 | O | 10.28352 | 10.91457 | 0.8990910 | H | 21.52552 | 4.591133 | 0.9420910 |
| C | 16.67005 | 4.576300 | 0.8188910 | O | 12.30498 | 12.08092 | 0.9052410 | C | 12.43730 | 7.096361 | 0.8189410 |
| C | 15.99938 | 3.338292 | 0.8165410 | C | 12.45361 | 15.18074 | 0.9523410 | C | 11.74939 | 8.299501 | 0.8403160 |
| H | 12.79071 | 4.568421 | 0.7937660 | C | 28.04780 | 14.42404 | 0.9369160 | C | 12.42443 | 9.535039 | 0.8623910 |
| H | 16.52786 | 6.733695 | 0.8211410 | C | 26.85874 | 15.17908 | 0.9367160 | C | 13.83190 | 9.496081 | 0.8580660 |
| H | 17.77229 | 4.590982 | 0.8270910 | C | 26.85885 | 16.54380 | 0.9158660 | C | 14.52433 | 8.295693 | 0.8370410 |
| H | 14.03087 | 2.423710 | 0.8002660 | C | 28.04751 | 17.29885 | 0.8990160 | C | 13.84081 | 7.069730 | 0.8189160 |
| C | 16.07924 | 15.86807 | 1.017216 | C | 28.10753 | 13.01686 | 0.9479160 | H | 10.64704 | 8.289003 | 0.8411660 |
| C | 16.81173 | 17.07067 | 1.014266 | O | 25.49746 | 14.69534 | 0.9585160 | H | 14.39634 | 10.44282 | 0.8734910 |
| C | 18.19785 | 17.07721 | 1.019991 | O | 25.49861 | 17.02901 | 0.9232410 | H | 15.62817 | 8.292979 | 0.8365410 |
| C | 18.92305 | 15.87533 | 1.030216 | C | 28.10630 | 18.70596 | 0.8757660 | H | 11.88279 | 6.142143 | 0.8037910 |
| C | 18.20316 | 14.67029 | 1.041591 | B | 22.55716 | 2.047775 | 0.9600160 | C | 24.77636 | 8.307850 | 0.9863910 |
| C | 16.81724 | 14.66902 | 1.034516 | B | 11.67911 | 10.83068 | 0.8893910 | C | 25.47579 | 9.504525 | 0.9763160 |
| H | 16.26928 | 18.03031 | 1.003291 | B | 24.72792 | 15.86281 | 0.9517160 | C | 26.88352 | 9.536773 | 0.9833910 |
| H | 16.28000 | 13.70649 | 1.040341 | O | 13.81292 | 17.03118 | 0.9851660 | C | 5.788726 | 8.297616 | 1.004091 |
| C | 30.53716 | 17.30121 | 0.9112910 | O | 13.81525 | 14.69754 | 0.9736160 | C | 5.093962 | 7.098472 | 1.015991 |
| C | 30.53635 | 18.73719 | 0.8999910 | O | 27.00774 | 12.08085 | 0.9642660 | C | 25.45391 | 7.078588 | 1.005641 |
| C | 20.90273 | 0.541890 | 0.9192160 | O | 7.265470 | 10.91354 | 0.9530160 | H | 24.91649 | 10.45439 | 0.9597660 |
| C | 11.20333 | 18.70792 | 0.9433910 | O | 18.14427 | 1.962037 | 0.8520410 | H | 6.891042 | 8.280691 | 1.010316 |
| C | 11.26375 | 17.30048 | 0.9436160 | O | 16.12396 | 0.793766 | 0.8513410 | H | 5.642541 | 6.140729 | 1.031316 |
| C | 18.40620 | 0.541360 | 0.8760160 | B | 14.58440 | 15.86510 | 0.9939160 | H | 23.67278 | 8.312147 | 0.9775910 |
| O | 21.16193 | 1.962659 | 0.9276160 | B | 27.63308 | 10.83017 | 0.9673660 | C | 21.11555 | 17.07632 | 0.9937410 |
| O | 1.420584 | 0.797856 | 0.9689660 | B | 16.74851 | 2.044647 | 0.8373160 | C | 22.50151 | 17.06893 | 0.9722910 |
| C | 12.45272 | 16.54561 | 0.9598660 | H | 18.75806 | 13.71599 | 1.052166 | C | 23.23318 | 15.86591 | 0.9759910 |
| C | 29.29102 | 16.58125 | 0.9105410 | H | 18.74669 | 18.03484 | 1.012941 | C | 22.49501 | 14.66727 | 1.004991 |
| C | 29.29134 | 15.14210 | 0.9249160 | C | 22.62760 | 4.580050 | 0.9666410 | C | 21.10926 | 14.66940 | 1.026141 |
| C | 8.774544 | 14.42274 | 0.9261410 | C | 23.31602 | 5.783171 | 0.9787660 | C | 20.39008 | 15.87488 | 1.019666 |
| C | 10.02105 | 15.14259 | 0.9282160 | C | 24.71927 | 5.808954 | 1.006491 | H | 23.04468 | 18.02798 | 0.9507410 |
| C | 10.02046 | 16.58204 | 0.9277410 | C | 3.638383 | 4.582425 | 1.027216 | H | 23.03190 | 13.70448 | 1.009716 |
| C | 8.774642 | 12.98680 | 0.9277660 | C | 2.945480 | 3.382245 | 1.015341 | H | 20.55391 | 13.71558 | 1.046541 |
| C | 10.02321 | 12.33531 | 0.9173660 | C | 23.30188 | 3.343890 | 0.9819910 | H | 20.56711 | 18.03412 | 0.9883660 |

Transformation Matrix

| | | | | |
|---|---|---|---|---|
| **a** | 21.763390 | 0.000000 | 0.000000 | |
| **b** | 10.881700 | 18.847640 | 0.000000 | |
| **c** | 0.000000 | 0.000000 | 3.472735 | AA |
| **c** | 1.38742 | 0.000000 | 3.415897 | inclined zigzag |
| **c** | 1.21549 | 0.70176 | 3.41316 | inclined armchair |

### Layer II AB stacking

| | | | | | | | | | | | |
|---|---|---|---|---|---|---|---|---|---|---|---|
| C | 25.47347 | 9.655238 | 4.144956 | C | 22.08711 | 19.30011 | 4.261506 | H | 33.64371 | 13.02020 | 4.304256 |
| C | 24.77617 | 10.85319 | 4.140856 | C | 22.14661 | 20.70742 | 4.274306 | H | 15.62368 | 10.86705 | 4.391381 |
| C | 25.45560 | 12.08155 | 4.149306 | C | 18.40788 | 18.61699 | 4.281281 | H | 14.39090 | 8.717607 | 4.371131 |
| C | 26.85912 | 12.05931 | 4.155981 | O | 21.16521 | 17.19712 | 4.239856 | H | 32.40722 | 10.87368 | 4.282856 |
| C | 27.55174 | 10.85885 | 4.159656 | O | 23.18668 | 18.36347 | 4.246006 | C | 23.31899 | 13.37891 | 4.159706 |
| C | 26.88107 | 9.620841 | 4.157306 | C | 23.33530 | 21.46329 | 4.293106 | C | 22.63108 | 14.58205 | 4.181081 |
| H | 23.67241 | 10.85097 | 4.134531 | C | 38.92950 | 20.70659 | 4.277681 | C | 23.30613 | 15.81759 | 4.203156 |
| H | 27.40956 | 13.01624 | 4.161906 | C | 37.74043 | 21.46163 | 4.277481 | C | 24.71359 | 15.77863 | 4.198831 |
| H | 28.65398 | 10.87353 | 4.167856 | C | 37.74055 | 22.82635 | 4.256631 | C | 25.40602 | 14.57824 | 4.177806 |
| H | 24.91257 | 8.706260 | 4.141031 | C | 38.92921 | 23.58140 | 4.239781 | C | 24.72250 | 13.35228 | 4.159681 |
| C | 26.96094 | 22.15062 | 4.357981 | C | 38.98923 | 19.29941 | 4.288681 | H | 21.52874 | 14.57155 | 4.181931 |
| C | 27.69342 | 23.35322 | 4.355031 | O | 36.37915 | 20.97789 | 4.299281 | H | 25.27803 | 16.72537 | 4.214256 |
| C | 29.07954 | 23.35976 | 4.360756 | O | 36.38030 | 23.31156 | 4.264006 | H | 26.50986 | 14.57553 | 4.177306 |
| C | 29.80474 | 22.15788 | 4.370981 | C | 38.98800 | 24.98851 | 4.216531 | H | 22.76448 | 12.42469 | 4.144556 |
| C | 29.08485 | 20.95284 | 4.382356 | B | 33.43885 | 8.330324 | 4.300781 | C | 35.65805 | 14.59040 | 4.327156 |

| | | | | | | | | | | | |
|---|---|---|---|---|---|---|---|---|---|---|---|
| C | 27.69893 | 20.95157 | 4.375281 | B | 22.56080 | 17.11323 | 4.230156 | C | 36.35748 | 15.78708 | 4.317081 |
| H | 27.15097 | 24.31286 | 4.344056 | B | 35.60962 | 22.14536 | 4.292481 | C | 37.76522 | 15.81932 | 4.324156 |
| H | 27.16170 | 19.98904 | 4.381106 | O | 24.69461 | 23.31373 | 4.325931 | C | 16.67042 | 14.58017 | 4.344856 |
| C | 41.41885 | 23.58376 | 4.252056 | O | 24.69695 | 20.98009 | 4.314381 | C | 15.97566 | 13.38102 | 4.356756 |
| C | 41.41805 | 25.01974 | 4.240756 | O | 37.88943 | 18.36340 | 4.305031 | C | 36.33560 | 13.36114 | 4.346406 |
| C | 31.78443 | 6.824440 | 4.259981 | O | 18.14717 | 17.19609 | 4.293781 | H | 35.79819 | 16.73694 | 4.300531 |
| C | 22.08503 | 24.99047 | 4.284156 | O | 29.02596 | 8.244586 | 4.192806 | H | 17.77274 | 14.56324 | 4.351081 |
| C | 22.14544 | 23.58303 | 4.284381 | O | 27.00566 | 7.076315 | 4.192106 | H | 16.52423 | 12.42328 | 4.372081 |
| C | 29.28790 | 6.823909 | 4.216781 | B | 25.46609 | 22.14765 | 4.334681 | H | 34.55447 | 14.59470 | 4.318356 |
| O | 32.04362 | 8.245209 | 4.268381 | B | 38.51477 | 17.11272 | 4.308131 | C | 31.99724 | 23.35887 | 4.334506 |
| O | 12.30228 | 7.080405 | 4.309731 | B | 27.63021 | 8.327196 | 4.178081 | C | 33.38321 | 23.35148 | 4.313056 |
| C | 23.33442 | 22.82816 | 4.300631 | H | 29.63976 | 19.99854 | 4.392931 | C | 34.11488 | 22.14846 | 4.316756 |
| C | 40.17271 | 22.86380 | 4.251306 | H | 29.62839 | 24.31739 | 4.353706 | C | 33.37671 | 20.94982 | 4.345756 |
| C | 40.17303 | 21.42465 | 4.265681 | C | 33.50929 | 10.86260 | 4.307406 | C | 31.99096 | 20.95195 | 4.366906 |
| C | 19.65624 | 20.70529 | 4.266906 | C | 34.19772 | 12.06572 | 4.319531 | C | 31.27177 | 22.15743 | 4.360431 |
| C | 20.90274 | 21.42514 | 4.268981 | C | 35.60097 | 12.09150 | 4.347256 | H | 33.92638 | 24.31053 | 4.291506 |
| C | 20.90215 | 22.86459 | 4.268506 | C | 14.52008 | 10.86497 | 4.367981 | H | 33.91360 | 19.98703 | 4.350481 |
| C | 19.65634 | 19.26935 | 4.268531 | C | 13.82718 | 9.664795 | 4.356106 | H | 31.43560 | 19.99813 | 4.387306 |
| C | 20.90490 | 18.61786 | 4.258131 | C | 34.18357 | 9.626439 | 4.322756 | H | 31.44880 | 24.31667 | 4.329131 |

**c** 0.00000 0.00000 6.68153 AB

## Layer II serrated zigzag

| | | | | | | | | | | | |
|---|---|---|---|---|---|---|---|---|---|---|---|
| C | 15.97920 | 3.372689 | 4.210511 | C | 12.59284 | 13.017560 | 4.327061 | H | 24.14943 | 6.737653 | 4.369811 |
| C | 15.28190 | 4.570645 | 4.206411 | C | 12.65234 | 14.424870 | 4.339861 | H | 6.12940 | 4.584498 | 4.456936 |
| C | 15.96132 | 5.799003 | 4.214861 | C | 8.91360 | 12.334440 | 4.346836 | H | 4.89662 | 2.435057 | 4.436686 |
| C | 17.36484 | 5.776763 | 4.221536 | O | 11.67094 | 10.914570 | 4.305411 | H | 22.91294 | 4.591133 | 4.348411 |
| C | 18.05747 | 4.576300 | 4.225211 | O | 13.69240 | 12.080920 | 4.311561 | C | 13.82472 | 7.096361 | 4.225261 |
| C | 17.38680 | 3.338292 | 4.222861 | C | 13.84103 | 15.180740 | 4.358661 | C | 13.13681 | 8.299501 | 4.246636 |
| H | 14.17813 | 4.568421 | 4.200086 | C | 29.43522 | 14.424040 | 4.343236 | C | 13.81185 | 9.535039 | 4.268711 |
| H | 17.91528 | 6.733695 | 4.227461 | C | 28.24616 | 15.179080 | 4.343036 | C | 15.21932 | 9.496081 | 4.264386 |
| H | 19.15971 | 4.590982 | 4.233411 | C | 28.24627 | 16.543800 | 4.322186 | C | 15.91175 | 8.295693 | 4.243361 |
| H | 15.41829 | 2.423710 | 4.206586 | C | 29.43493 | 17.298850 | 4.305336 | C | 15.22823 | 7.069730 | 4.225236 |
| C | 17.46666 | 15.868070 | 4.423536 | C | 7.73156 | 13.016860 | 4.354236 | H | 12.03446 | 8.289003 | 4.247486 |
| C | 18.19915 | 17.070670 | 4.420586 | O | 26.88488 | 14.695340 | 4.364836 | H | 15.78376 | 10.442820 | 4.279811 |
| C | 19.58527 | 17.077210 | 4.426311 | O | 26.88603 | 17.029010 | 4.329561 | H | 17.01559 | 8.292979 | 4.242861 |
| C | 20.31047 | 15.875330 | 4.436536 | C | 29.49372 | 18.705960 | 4.282086 | H | 13.27021 | 6.142143 | 4.210111 |
| C | 19.59058 | 14.670290 | 4.447911 | B | 2.18119 | 2.047775 | 4.366336 | C | 26.16378 | 8.307850 | 4.392711 |
| C | 18.20466 | 14.669020 | 4.440836 | B | 13.06653 | 10.830680 | 4.295711 | C | 26.86321 | 9.504525 | 4.382636 |
| H | 17.65670 | 18.030310 | 4.409611 | B | 26.11534 | 15.862810 | 4.358036 | C | 6.50755 | 9.536773 | 4.389711 |
| H | 17.66742 | 13.706490 | 4.446661 | O | 15.20034 | 17.031180 | 4.391486 | C | 7.17614 | 8.297616 | 4.410411 |
| C | 10.16119 | 17.301210 | 4.317611 | O | 15.20267 | 14.697540 | 4.379936 | C | 6.48138 | 7.098472 | 4.422311 |
| C | 31.92377 | 18.737190 | 4.306311 | O | 28.39516 | 12.080850 | 4.370586 | C | 5.07794 | 7.078588 | 4.411961 |
| C | 0.52676 | 0.541890 | 4.325536 | O | 8.65289 | 10.913540 | 4.359336 | H | 26.30391 | 10.454390 | 4.366086 |
| C | 12.59075 | 18.707920 | 4.349711 | O | 19.53169 | 1.962037 | 4.258361 | H | 8.27846 | 8.280691 | 4.416636 |
| C | 12.65117 | 17.300480 | 4.349936 | O | 17.51138 | 0.793766 | 4.257661 | H | 7.02996 | 6.140729 | 4.437636 |
| C | 19.79362 | 0.541360 | 4.282336 | B | 15.97182 | 15.865100 | 4.400236 | H | 25.06020 | 8.312147 | 4.383911 |
| O | 22.54935 | 1.962659 | 4.333936 | B | 7.25711 | 10.830170 | 4.373686 | C | 22.50297 | 17.076320 | 4.400061 |
| O | 2.80800 | 0.797856 | 4.375286 | B | 18.13593 | 2.044647 | 4.243636 | C | 23.88893 | 17.068930 | 4.378611 |
| C | 13.84014 | 16.545610 | 4.366186 | H | 20.14548 | 13.715990 | 4.458486 | C | 24.62060 | 15.865910 | 4.382311 |
| C | 30.67844 | 16.581250 | 4.316861 | H | 20.13411 | 18.034840 | 4.419261 | C | 23.88243 | 14.667270 | 4.411311 |
| C | 8.91537 | 15.142100 | 4.331236 | C | 24.01502 | 4.580050 | 4.372961 | C | 22.49668 | 14.669400 | 4.432461 |
| C | 10.16196 | 14.422740 | 4.332461 | C | 24.70344 | 5.783171 | 4.385086 | C | 21.77750 | 15.874880 | 4.425986 |
| C | 11.40847 | 15.142590 | 4.334536 | C | 4.34330 | 5.808954 | 4.412811 | H | 24.43210 | 18.027980 | 4.357061 |
| C | 11.40788 | 16.582040 | 4.334061 | C | 5.02580 | 4.582425 | 4.433536 | H | 24.41932 | 13.704480 | 4.416036 |
| C | 10.16206 | 12.986800 | 4.334086 | C | 4.33290 | 3.382245 | 4.421661 | H | 21.94133 | 13.715580 | 4.452861 |
| C | 11.41063 | 12.335310 | 4.323686 | C | 2.92591 | 3.343890 | 4.388311 | H | 21.95453 | 18.034120 | 4.394686 |

**c** 0.00000 0.00000 6.81264 serrated zigzag

## Layer II serrated armchair

| | | | | | | | | | | | |
|---|---|---|---|---|---|---|---|---|---|---|---|
| C | 15.807270 | 4.074449 | 4.210511 | C | 12.420910 | 13.719320 | 4.327061 | H | 23.977500 | 7.439413 | 4.369811 |
| C | 15.109970 | 5.272405 | 4.206411 | C | 12.480410 | 15.126630 | 4.339861 | H | 5.957473 | 5.286258 | 4.456936 |

| | | | | | | | | | | | |
|---|---|---|---|---|---|---|---|---|---|---|---|
| C | 15.789390 | 6.500763 | 4.214861 | C | 8.741675 | 13.036200 | 4.346836 | H | 4.724696 | 3.136817 | 4.436686 |
| C | 17.192910 | 6.478523 | 4.221536 | O | 11.499010 | 11.616330 | 4.305411 | H | 22.741010 | 5.292893 | 4.348411 |
| C | 17.885540 | 5.278060 | 4.225211 | O | 13.520470 | 12.782680 | 4.311561 | C | 13.652790 | 7.798121 | 4.225261 |
| C | 17.214870 | 4.040052 | 4.222861 | C | 13.669100 | 15.882500 | 4.358661 | C | 12.964880 | 9.001262 | 4.246636 |
| H | 14.006200 | 5.270181 | 4.200086 | C | 29.263290 | 15.125800 | 4.343236 | C | 13.639920 | 10.236800 | 4.268711 |
| H | 17.743350 | 7.435455 | 4.227461 | C | 28.074230 | 15.880840 | 4.343036 | C | 15.047390 | 10.197840 | 4.264386 |
| H | 18.987780 | 5.292742 | 4.233411 | C | 28.074340 | 17.245560 | 4.322186 | C | 15.739820 | 8.997454 | 4.243361 |
| H | 15.246360 | 3.125470 | 4.206586 | C | 29.263000 | 18.000610 | 4.305336 | C | 15.056300 | 7.771490 | 4.225236 |
| C | 17.294730 | 16.569830 | 4.423536 | C | 29.323020 | 13.718620 | 4.354236 | H | 11.862530 | 8.990764 | 4.247486 |
| C | 18.027220 | 17.772430 | 4.420586 | O | 26.712950 | 15.397100 | 4.364836 | H | 15.611830 | 11.144580 | 4.279811 |
| C | 19.413340 | 17.778970 | 4.426311 | O | 26.714100 | 17.730770 | 4.329561 | H | 16.843660 | 8.994740 | 4.242861 |
| C | 20.138540 | 16.577090 | 4.436536 | C | 18.440090 | 0.560080 | 4.282086 | H | 13.098280 | 6.843903 | 4.210111 |
| C | 19.418650 | 15.372050 | 4.447911 | B | 2.009260 | 2.749535 | 4.366336 | C | 25.991850 | 9.009610 | 4.392711 |
| C | 18.032730 | 15.370780 | 4.440836 | B | 12.894600 | 11.532440 | 4.295711 | C | 26.691280 | 10.206290 | 4.382636 |
| H | 17.484770 | 18.732070 | 4.409611 | B | 25.943410 | 16.564570 | 4.358036 | C | 6.335620 | 10.238530 | 4.389711 |
| H | 17.495490 | 14.408250 | 4.446661 | O | 15.028410 | 17.732940 | 4.391486 | C | 7.004216 | 8.999376 | 4.410411 |
| C | 31.752650 | 18.002970 | 4.317611 | O | 15.030740 | 15.399300 | 4.379936 | C | 6.309452 | 7.800232 | 4.422311 |
| C | 20.870140 | 0.591310 | 4.306311 | O | 28.223230 | 12.782610 | 4.370586 | C | 4.906010 | 7.780348 | 4.411961 |
| C | 22.118220 | 1.243650 | 4.325536 | O | 8.480960 | 11.615300 | 4.359336 | H | 26.131980 | 11.156150 | 4.366086 |
| C | 1.537120 | 0.562040 | 4.349711 | O | 19.359760 | 2.663797 | 4.258361 | H | 8.106532 | 8.982451 | 4.416636 |
| C | 12.479240 | 18.002240 | 4.349936 | O | 17.339450 | 1.495526 | 4.257661 | H | 6.858031 | 6.842489 | 4.437636 |
| C | 19.621690 | 1.243120 | 4.282336 | B | 15.799890 | 16.566860 | 4.400236 | H | 24.888270 | 9.013907 | 4.383911 |
| O | 22.377420 | 2.664419 | 4.333936 | B | 7.085180 | 11.531930 | 4.373686 | C | 22.331040 | 17.778080 | 4.400061 |
| O | 2.636074 | 1.499616 | 4.375286 | B | 17.964000 | 2.746407 | 4.243636 | C | 23.717000 | 17.770690 | 4.378611 |
| C | 13.668210 | 17.247370 | 4.366186 | H | 19.973550 | 14.417750 | 4.458486 | C | 24.448670 | 16.567670 | 4.382311 |
| C | 30.506510 | 17.283010 | 4.316861 | H | 19.962180 | 18.736600 | 4.419261 | C | 23.710500 | 15.369030 | 4.411311 |
| C | 30.506830 | 15.843860 | 4.331236 | C | 23.843090 | 5.281810 | 4.372961 | C | 22.324750 | 15.371160 | 4.432461 |
| C | 9.990034 | 15.124500 | 4.332461 | C | 24.531510 | 6.484931 | 4.385086 | C | 21.605570 | 16.576640 | 4.425986 |
| C | 11.236540 | 15.844350 | 4.334536 | C | 4.171370 | 6.510714 | 4.412811 | H | 24.260170 | 18.729740 | 4.357061 |
| C | 11.235950 | 17.283800 | 4.334061 | C | 4.853873 | 5.284185 | 4.433536 | H | 24.247390 | 14.406240 | 4.416036 |
| C | 9.990132 | 13.688560 | 4.334086 | C | 4.160970 | 4.084005 | 4.421661 | H | 21.769400 | 14.417340 | 4.452861 |
| C | 11.238700 | 13.037070 | 4.323686 | C | 2.753980 | 4.045650 | 4.388311 | H | 21.782600 | 18.735880 | 4.394686 |

**c** 0.00000 0.00000 6.81264 serrated armchair

## COF-10M

### Layer I AA, AB, inclined zigzag & armchair, and serrated zigzag & armchair

| | | | | | | | | | | | |
|---|---|---|---|---|---|---|---|---|---|---|---|
| C | 16.57332 | 12.16294 | 0.814356 | C | 20.62796 | 19.13068 | 0.902936 | C | 25.32753 | 18.59247 | 0.914636 |
| C | 17.77586 | 11.44018 | 0.814516 | C | 19.21583 | 19.08032 | 0.900016 | C | 24.09156 | 17.91935 | 0.918856 |
| C | 18.97789 | 12.16352 | 0.828336 | C | 18.46343 | 17.88479 | 0.892766 | C | 24.12646 | 16.51218 | 0.932736 |
| C | 18.97659 | 13.54880 | 0.844436 | C | 17.09066 | 17.88596 | 0.890156 | C | 25.32587 | 15.81852 | 0.942006 |
| C | 17.77513 | 14.28179 | 0.847666 | O | 7.671621 | 7.067490 | 0.936066 | C | 15.28373 | 7.113374 | 0.803886 |
| C | 16.57405 | 13.54829 | 0.830836 | B | 6.515409 | 7.841362 | 0.948236 | C | 14.05487 | 7.790636 | 0.807086 |
| C | 17.77726 | 9.968939 | 0.806816 | O | 5.356762 | 7.070887 | 0.939356 | C | 12.85693 | 7.094708 | 0.823616 |
| C | 16.57504 | 9.250811 | 0.798486 | O | 25.28426 | 5.565122 | 0.892136 | C | 12.82444 | 5.687621 | 0.836786 |
| C | 16.55731 | 7.850500 | 0.800456 | O | 14.00620 | 18.05991 | 0.898636 | C | 14.06156 | 5.016470 | 0.828516 |
| C | 17.78079 | 7.168805 | 0.808686 | B | 12.75822 | 18.67559 | 0.900236 | C | 15.26056 | 5.710602 | 0.812536 |
| C | 19.00276 | 7.853272 | 0.816356 | C | 11.46452 | 17.92673 | 0.907886 | H | 21.48687 | 0.175720 | 0.896186 |
| C | 18.98126 | 9.253584 | 0.815506 | C | 32.77322 | 18.59870 | 0.905736 | H | 30.61651 | 18.45701 | 0.913516 |
| C | 20.27827 | 7.119095 | 0.830066 | C | 31.57336 | 17.90601 | 0.915616 | H | 10.23199 | 14.72098 | 0.941416 |
| C | 21.50558 | 7.798915 | 0.841436 | C | 31.54857 | 16.50340 | 0.928956 | H | 12.37858 | 15.95767 | 0.923116 |
| C | 22.70539 | 7.105876 | 0.856886 | C | 10.23131 | 15.82520 | 0.931016 | H | 8.669429 | 13.81456 | 0.959056 |
| C | 22.74164 | 5.698829 | 0.861806 | C | 11.43031 | 16.51954 | 0.920556 | H | 26.89355 | 13.80858 | 0.967386 |
| C | 21.50576 | 5.025237 | 0.849186 | O | 1.575940 | 0.539410 | 0.897136 | H | 29.04920 | 17.55431 | 0.922016 |
| C | 20.30502 | 5.716323 | 0.833916 | O | 16.61842 | 16.54940 | 0.874986 | H | 27.48455 | 18.45185 | 0.919466 |
| B | 24.03574 | 4.950769 | 0.878316 | B | 17.77531 | 15.77627 | 0.867836 | H | 14.06743 | 0.169370 | 0.903936 |
| O | 24.12467 | 3.561997 | 0.879736 | O | 18.93337 | 16.54752 | 0.879716 | H | 23.17825 | 15.95051 | 0.936736 |
| C | 2.972248 | 3.299269 | 0.893536 | O | 21.54908 | 18.05305 | 0.909036 | H | 25.32609 | 14.71442 | 0.953266 |
| C | 3.631523 | 2.049202 | 0.896586 | B | 22.79765 | 18.66766 | 0.910306 | H | 26.89911 | 12.00369 | 0.978186 |

| | | | | | | | | | | | |
|---|---|---|---|---|---|---|---|---|---|---|---|
| C | 5.067356 | 2.047992 | 0.901166 | O | 11.43621 | 0.531490 | 0.904156 | H | 26.89981 | 9.524688 | 0.970436 |
| C | 5.788824 | 3.293977 | 0.906416 | O | 11.43922 | 3.551238 | 0.869056 | H | 8.677974 | 9.532068 | 0.965736 |
| C | 5.072328 | 4.537913 | 0.910486 | B | 11.53050 | 4.939757 | 0.860686 | H | 8.669823 | 12.01055 | 0.973466 |
| C | 3.660156 | 4.487441 | 0.901066 | O | 10.28333 | 5.556746 | 0.876836 | H | 23.65301 | 7.668761 | 0.865466 |
| C | 5.825573 | 5.733271 | 0.922416 | C | 30.27453 | 15.76684 | 0.940656 | H | 21.50498 | 8.902993 | 0.838316 |
| C | 7.198402 | 5.731416 | 0.920416 | C | 30.25672 | 14.36666 | 0.956036 | H | 19.34894 | 5.164138 | 0.824916 |
| C | 7.948356 | 4.534379 | 0.907216 | C | 29.05454 | 13.64834 | 0.965806 | H | 21.49355 | 3.923150 | 0.851736 |
| C | 7.228782 | 3.292298 | 0.905656 | C | 27.85025 | 14.36334 | 0.960666 | H | 17.78187 | 6.062794 | 0.812036 |
| C | 7.947905 | 2.044985 | 0.901736 | C | 27.82890 | 15.76352 | 0.945156 | H | 15.61671 | 9.803075 | 0.793766 |
| C | 9.383546 | 2.043364 | 0.897316 | C | 29.05074 | 16.44824 | 0.935286 | H | 19.93805 | 9.808386 | 0.824316 |
| C | 10.04565 | 3.291537 | 0.888756 | C | 29.05698 | 12.17717 | 0.974036 | H | 19.93378 | 11.61083 | 0.827966 |
| C | 9.360223 | 4.481037 | 0.893756 | C | 27.85581 | 11.45246 | 0.975886 | H | 19.93720 | 14.08908 | 0.856206 |
| C | 7.226426 | 0.799057 | 0.901336 | C | 27.85928 | 10.06717 | 0.971306 | H | 15.61331 | 14.08849 | 0.831566 |
| C | 5.786580 | 0.800659 | 0.900016 | C | 6.516085 | 9.335764 | 0.964886 | H | 15.61756 | 11.61013 | 0.802706 |
| C | 16.34050 | 19.08315 | 0.895956 | C | 7.716351 | 10.07074 | 0.968686 | H | 14.05293 | 8.894695 | 0.798816 |
| C | 14.92855 | 19.13630 | 0.895916 | C | 7.714987 | 11.45613 | 0.973236 | H | 11.90755 | 7.654508 | 0.828006 |
| C | 2.969599 | 0.800444 | 0.895316 | C | 26.55372 | 16.49787 | 0.937136 | H | 14.07635 | 3.914481 | 0.837066 |
| C | 10.04314 | 0.793766 | 0.900286 | C | 26.52789 | 17.90045 | 0.923286 | H | 16.21818 | 5.161092 | 0.808836 |

Transformation Matrix

| | | | | |
|---|---|---|---|---|
| **a** | 22.545500 | 0.000000 | 0.000000 | |
| **b** | 11.272750 | 19.524970 | 0.000000 | |
| **c** | 0.000000 | 0.000000 | 3.415248 | AA |
| **c** | 1.20129 | 0.693564 | 3.399478 | iclined zigzag |
| **c** | 1.408306 | 0.00000 | 3.39914 | iclined armchair |

## Layer II AB stacking

| | | | | | | | | | | | |
|---|---|---|---|---|---|---|---|---|---|---|---|
| C | 27.84607 | 18.67126 | 4.182771 | C | 31.90071 | 25.63901 | 4.271351 | C | 36.60028 | 25.10080 | 4.283051 |
| C | 29.04861 | 17.94851 | 4.182931 | C | 30.48858 | 25.58865 | 4.268431 | C | 35.36431 | 24.42768 | 4.287271 |
| C | 30.25064 | 18.67184 | 4.196751 | C | 29.73618 | 24.39312 | 4.261181 | C | 35.39921 | 23.02051 | 4.301151 |
| C | 30.24934 | 20.05713 | 4.212851 | C | 28.36341 | 24.39429 | 4.258571 | C | 36.59862 | 22.32685 | 4.310421 |
| C | 29.04788 | 20.79012 | 4.216081 | O | 18.94437 | 13.57582 | 4.304481 | C | 26.55648 | 13.62170 | 4.172301 |
| C | 27.84680 | 20.05662 | 4.199251 | B | 17.78816 | 14.34969 | 4.316651 | C | 25.32762 | 14.29896 | 4.175501 |
| C | 29.05001 | 16.47726 | 4.175231 | O | 16.62951 | 13.57921 | 4.307771 | C | 24.12968 | 13.60303 | 4.192031 |
| C | 27.84779 | 15.75914 | 4.166901 | O | 36.55701 | 12.07345 | 4.260551 | C | 24.09719 | 12.19595 | 4.205201 |
| C | 27.83006 | 14.35883 | 4.168871 | O | 25.27895 | 24.56824 | 4.267051 | C | 25.33431 | 11.52480 | 4.196931 |
| C | 29.05354 | 13.67713 | 4.177101 | B | 24.03097 | 25.18392 | 4.268651 | C | 26.53331 | 12.21893 | 4.180951 |
| C | 30.27551 | 14.36160 | 4.184771 | C | 22.73727 | 24.43505 | 4.276301 | H | 32.75962 | 6.684045 | 4.264601 |
| C | 30.25401 | 15.76191 | 4.183921 | C | 44.04597 | 25.10703 | 4.274151 | H | 41.88926 | 24.96534 | 4.281931 |
| C | 31.55102 | 13.62742 | 4.198481 | C | 42.84611 | 24.41434 | 4.284031 | H | 21.50474 | 21.22931 | 4.309831 |
| C | 32.77833 | 14.30724 | 4.209851 | C | 42.82132 | 23.01173 | 4.297371 | H | 23.65133 | 22.46600 | 4.291531 |
| C | 33.97814 | 13.61420 | 4.225301 | C | 21.50406 | 22.33352 | 4.299431 | H | 19.94218 | 20.32289 | 4.327471 |
| C | 34.01439 | 12.20715 | 4.230221 | C | 22.70306 | 23.02787 | 4.288971 | H | 38.16630 | 20.31691 | 4.335801 |
| C | 32.77851 | 11.53356 | 4.217601 | O | 12.84869 | 7.047735 | 4.265551 | H | 40.32195 | 24.06264 | 4.290431 |
| C | 31.57777 | 12.22465 | 4.202331 | O | 27.89117 | 23.05773 | 4.243401 | H | 38.75730 | 24.96018 | 4.287881 |
| B | 35.30849 | 11.45909 | 4.246731 | B | 29.04806 | 22.28460 | 4.236251 | H | 25.34018 | 6.677695 | 4.272351 |
| O | 35.39742 | 10.07032 | 4.248151 | O | 30.20612 | 23.05585 | 4.248131 | H | 34.45100 | 22.45884 | 4.305151 |
| C | 14.24500 | 9.807594 | 4.261951 | O | 32.82183 | 24.56137 | 4.277451 | H | 36.59884 | 21.22275 | 4.321681 |
| C | 14.90427 | 8.557528 | 4.265001 | B | 34.07040 | 25.17599 | 4.278721 | H | 38.17186 | 18.51201 | 4.346601 |
| C | 16.34011 | 8.556317 | 4.269581 | O | 22.70896 | 7.039815 | 4.272571 | H | 38.17256 | 16.03301 | 4.338851 |
| C | 17.06157 | 9.802302 | 4.274831 | O | 22.71197 | 10.05956 | 4.237471 | H | 19.95072 | 16.04039 | 4.334151 |
| C | 16.34508 | 11.04624 | 4.278901 | B | 22.80325 | 11.44808 | 4.229101 | H | 19.94257 | 18.51888 | 4.341881 |
| C | 14.93291 | 10.99577 | 4.269481 | O | 21.55608 | 12.06507 | 4.245251 | H | 34.92576 | 14.17709 | 4.233881 |
| C | 17.09832 | 12.24160 | 4.290831 | C | 41.54728 | 22.27517 | 4.309071 | H | 32.77773 | 15.41132 | 4.206731 |
| C | 18.47115 | 12.23974 | 4.288831 | C | 41.52947 | 20.87498 | 4.324451 | H | 30.62169 | 11.67246 | 4.193331 |
| C | 19.22111 | 11.04270 | 4.275631 | C | 40.32729 | 20.15667 | 4.334221 | H | 32.76630 | 10.43147 | 4.220151 |
| C | 18.50153 | 9.800623 | 4.274071 | C | 39.12300 | 20.87167 | 4.329081 | H | 29.05462 | 12.57112 | 4.180451 |
| C | 19.22066 | 8.553310 | 4.270151 | C | 39.10165 | 22.27184 | 4.313571 | H | 26.88946 | 16.31140 | 4.162181 |
| C | 20.65630 | 8.551689 | 4.265731 | C | 40.32349 | 22.95657 | 4.303701 | H | 31.21080 | 16.31671 | 4.192731 |
| C | 21.31840 | 9.799862 | 4.257171 | C | 40.32973 | 18.68550 | 4.342451 | H | 31.20653 | 18.11916 | 4.196381 |
| C | 20.63297 | 10.98936 | 4.262171 | C | 39.12856 | 17.96078 | 4.344301 | H | 31.20995 | 20.59740 | 4.224621 |
| C | 18.49918 | 7.307382 | 4.269751 | C | 39.13203 | 16.57549 | 4.339721 | H | 26.88606 | 20.59682 | 4.199981 |

| | | | | | | | | | | | |
|---|---|---|---|---|---|---|---|---|---|---|---|
| C | 17.05933 | 7.308984 | 4.268431 | C | 17.78884 | 15.84409 | 4.333301 | H | 26.89031 | 18.11846 | 4.171121 |
| C | 27.61325 | 25.59148 | 4.264371 | C | 18.98910 | 16.57907 | 4.337101 | H | 25.32568 | 15.40302 | 4.167231 |
| C | 26.20130 | 25.64462 | 4.264331 | C | 18.98774 | 17.96445 | 4.341651 | H | 23.18030 | 14.16283 | 4.196421 |
| C | 14.24235 | 7.308769 | 4.263731 | C | 37.82647 | 23.00620 | 4.305551 | H | 25.34910 | 10.42281 | 4.205481 |
| C | 21.31589 | 7.302091 | 4.268701 | C | 37.80064 | 24.40878 | 4.291701 | H | 27.49093 | 11.66942 | 4.177251 |
| **c** | 0.00000 | 0.00000 | 6.73683 | AB | | | | | | | |

## Layer II serrated zigzag

| | | | | | | | | | | | |
|---|---|---|---|---|---|---|---|---|---|---|---|
| C | 17.77461 | 12.85650 | 4.21071 | C | 10.55650 | 0.299270 | 4.299296 | C | 26.52882 | 19.28603 | 4.310996 |
| C | 18.97715 | 12.13374 | 4.210876 | C | 9.144370 | 0.248910 | 4.296376 | C | 25.29285 | 18.61291 | 4.315216 |
| C | 20.17918 | 12.85708 | 4.224696 | C | 19.66472 | 18.57835 | 4.289126 | C | 25.32775 | 17.20574 | 4.329096 |
| C | 20.17788 | 14.24236 | 4.240796 | C | 18.29195 | 18.57952 | 4.286516 | C | 26.52716 | 16.51208 | 4.338366 |
| C | 18.97642 | 14.97535 | 4.244026 | O | 8.872910 | 7.761054 | 4.332426 | C | 16.48502 | 7.806938 | 4.200246 |
| C | 17.77534 | 14.24185 | 4.227196 | B | 7.716699 | 8.534926 | 4.344596 | C | 15.25616 | 8.484200 | 4.203446 |
| C | 18.97855 | 10.66250 | 4.203176 | O | 6.558052 | 7.764451 | 4.335716 | C | 14.05822 | 7.788272 | 4.219976 |
| C | 17.77633 | 9.944375 | 4.194846 | O | 3.940050 | 6.258686 | 4.288496 | C | 14.02573 | 6.381185 | 4.233146 |
| C | 17.75860 | 8.544065 | 4.196816 | O | 15.20749 | 18.75347 | 4.294996 | C | 15.26285 | 5.710034 | 4.224876 |
| C | 18.98208 | 7.862369 | 4.205046 | B | 13.95951 | 19.36915 | 4.296596 | C | 16.46185 | 6.404166 | 4.208896 |
| C | 20.20405 | 8.546836 | 4.212716 | C | 12.66581 | 18.62029 | 4.304246 | H | 22.68816 | 0.869284 | 4.292546 |
| C | 20.18255 | 9.947148 | 4.211866 | C | 11.42901 | 19.29226 | 4.302096 | H | 31.81780 | 19.15057 | 4.309876 |
| C | 21.47956 | 7.812659 | 4.226426 | C | 32.77465 | 18.59957 | 4.311976 | H | 11.43328 | 15.41454 | 4.337776 |
| C | 22.70687 | 8.492479 | 4.237796 | C | 10.20436 | 17.19696 | 4.325316 | H | 13.57987 | 16.65123 | 4.319476 |
| C | 23.90668 | 7.799440 | 4.253246 | C | 11.43260 | 16.51876 | 4.327376 | H | 9.870719 | 14.50812 | 4.355416 |
| C | 23.94293 | 6.392393 | 4.258166 | C | 12.63160 | 17.21310 | 4.316916 | H | 28.09484 | 14.50214 | 4.363746 |
| C | 22.70705 | 5.718801 | 4.245546 | O | 2.777230 | 1.232974 | 4.293496 | H | 30.25049 | 18.24787 | 4.318376 |
| C | 21.50631 | 6.409887 | 4.230276 | O | 17.81971 | 17.24296 | 4.271346 | H | 28.68584 | 19.14541 | 4.315826 |
| B | 25.23703 | 5.644333 | 4.274676 | B | 18.97660 | 16.46983 | 4.264196 | H | 15.26872 | 0.862934 | 4.300296 |
| O | 2.780460 | 4.255561 | 4.276096 | O | 20.13466 | 17.24108 | 4.276076 | H | 24.37954 | 16.64407 | 4.333096 |
| C | 4.173538 | 3.992833 | 4.289896 | O | 22.75037 | 18.74661 | 4.305396 | H | 26.52738 | 15.40798 | 4.349626 |
| C | 4.832813 | 2.742766 | 4.292946 | B | 23.99894 | 19.36122 | 4.306666 | H | 28.10040 | 12.69725 | 4.374546 |
| C | 6.268646 | 2.741556 | 4.297526 | O | 12.63750 | 1.225054 | 4.300516 | H | 28.10110 | 10.21825 | 4.366796 |
| C | 6.990114 | 3.987541 | 4.302776 | O | 12.64051 | 4.244802 | 4.265416 | H | 9.879264 | 10.22563 | 4.362096 |
| C | 6.273618 | 5.231477 | 4.306846 | B | 12.73179 | 5.633321 | 4.257046 | H | 9.871113 | 12.70411 | 4.369826 |
| C | 4.861446 | 5.181005 | 4.297426 | O | 11.48462 | 6.250310 | 4.273196 | H | 24.85430 | 8.362325 | 4.261826 |
| C | 7.026863 | 6.426835 | 4.318776 | C | 31.47582 | 16.46040 | 4.337016 | H | 22.70627 | 9.596558 | 4.234676 |
| C | 8.399692 | 6.424980 | 4.316776 | C | 8.912510 | 15.06022 | 4.352396 | H | 20.55023 | 5.857702 | 4.221276 |
| C | 9.149646 | 5.227943 | 4.303576 | C | 30.25583 | 14.34190 | 4.362166 | H | 22.69484 | 4.616714 | 4.248096 |
| C | 8.430072 | 3.985862 | 4.302016 | C | 29.05154 | 15.05690 | 4.357026 | H | 18.98316 | 6.756358 | 4.208396 |
| C | 9.149195 | 2.738549 | 4.298096 | C | 29.03019 | 16.45708 | 4.341516 | H | 16.81800 | 10.49664 | 4.190126 |
| C | 10.58484 | 2.736928 | 4.293676 | C | 30.25203 | 17.14180 | 4.331646 | H | 21.13934 | 10.50195 | 4.220676 |
| C | 11.24694 | 3.985101 | 4.285116 | C | 7.712770 | 12.87073 | 4.370396 | H | 21.13507 | 12.30439 | 4.224326 |
| C | 10.56151 | 5.174601 | 4.290116 | C | 29.05710 | 12.14602 | 4.372246 | H | 21.13849 | 14.78264 | 4.252566 |
| C | 8.427716 | 1.492621 | 4.297696 | C | 6.515070 | 10.76073 | 4.367666 | H | 16.81460 | 14.78205 | 4.227926 |
| C | 6.987870 | 1.494223 | 4.296376 | C | 7.717375 | 10.02933 | 4.361246 | H | 16.81885 | 12.30369 | 4.199066 |
| C | 6.269040 | 0.251740 | 4.292316 | C | 8.917641 | 10.76430 | 4.365046 | H | 15.25422 | 9.588260 | 4.195176 |
| C | 4.857090 | 0.304890 | 4.292276 | C | 8.916277 | 12.14969 | 4.369596 | H | 13.10884 | 8.348072 | 4.224366 |
| C | 4.170889 | 1.494008 | 4.291676 | C | 27.75501 | 17.19143 | 4.333496 | H | 15.27764 | 4.608045 | 4.233426 |
| C | 11.24443 | 1.487330 | 4.296646 | C | 27.72918 | 18.59401 | 4.319646 | H | 17.41947 | 5.854656 | 4.205196 |
| **c** | 0.00000 | 0.00000 | 6.79272 | serrated zigzag | | | | | | | |

## Layer II serrated armchair

| | | | | | | | | | | | |
|---|---|---|---|---|---|---|---|---|---|---|---|
| C | 17.98163 | 12.16294 | 4.210716 | C | 22.03627 | 19.13068 | 4.299296 | C | 26.73584 | 18.59247 | 4.310996 |
| C | 19.18417 | 11.44018 | 4.210876 | C | 20.62414 | 19.08032 | 4.296376 | C | 25.49987 | 17.91935 | 4.315216 |
| C | 20.38620 | 12.16352 | 4.224696 | C | 19.87174 | 17.88479 | 4.289126 | C | 25.53477 | 16.51218 | 4.329096 |
| C | 20.38490 | 13.54880 | 4.240796 | C | 18.49897 | 17.88596 | 4.286516 | C | 26.73418 | 15.81852 | 4.338366 |
| C | 19.18344 | 14.28179 | 4.244026 | O | 9.079926 | 7.067490 | 4.332426 | C | 16.69204 | 7.113374 | 4.200246 |
| C | 17.98236 | 13.54829 | 4.227196 | B | 7.923715 | 7.841362 | 4.344596 | C | 15.46318 | 7.790636 | 4.203446 |
| C | 19.18557 | 9.968939 | 4.203176 | O | 6.765068 | 7.070887 | 4.335716 | C | 14.26524 | 7.094708 | 4.219976 |
| C | 17.98335 | 9.250811 | 4.194846 | O | 4.147070 | 5.565122 | 4.288496 | C | 14.23275 | 5.687621 | 4.233146 |
| C | 17.96562 | 7.850500 | 4.196816 | O | 15.41451 | 18.05991 | 4.294996 | C | 15.46987 | 5.016470 | 4.224876 |
| C | 19.18910 | 7.168805 | 4.205046 | B | 14.16653 | 18.67559 | 4.296596 | C | 16.66887 | 5.710602 | 4.208896 |

| | | | | | | | | | | | |
|---|---|---|---|---|---|---|---|---|---|---|---|
| C | 20.41107 | 7.853272 | 4.212716 | C | 12.87283 | 17.92673 | 4.304246 | H | 0.349680 | 0.175720 | 4.292546 |
| C | 20.38957 | 9.253584 | 4.211866 | C | 11.63603 | 18.59870 | 4.302096 | H | 32.02481 | 18.45701 | 4.309876 |
| C | 21.68658 | 7.119095 | 4.226426 | C | 10.43617 | 17.90601 | 4.311976 | H | 11.64030 | 14.72098 | 4.337776 |
| C | 22.91389 | 7.798915 | 4.237796 | C | 10.41137 | 16.50340 | 4.325316 | H | 13.78689 | 15.95767 | 4.319476 |
| C | 24.11370 | 7.105876 | 4.253246 | C | 11.63962 | 15.82520 | 4.327376 | H | 10.07773 | 13.81456 | 4.355416 |
| C | 24.14995 | 5.698829 | 4.258166 | C | 12.83862 | 16.51954 | 4.316916 | H | 28.30186 | 13.80858 | 4.363746 |
| C | 22.91407 | 5.025237 | 4.245546 | O | 2.984246 | 0.539410 | 4.293496 | H | 30.45751 | 17.55431 | 4.318376 |
| C | 21.71333 | 5.716323 | 4.230276 | O | 18.02673 | 16.54940 | 4.271346 | H | 28.89286 | 18.45185 | 4.315826 |
| B | 2.898550 | 4.950769 | 4.274676 | B | 19.18362 | 15.77627 | 4.264196 | H | 15.47574 | 0.169370 | 4.300296 |
| O | 2.987480 | 3.561997 | 4.276096 | O | 20.34168 | 16.54752 | 4.276076 | H | 24.58656 | 15.95051 | 4.333096 |
| C | 4.380554 | 3.299269 | 4.289896 | O | 22.95739 | 18.05305 | 4.305396 | H | 26.73440 | 14.71442 | 4.349626 |
| C | 5.039829 | 2.049202 | 4.292946 | B | 24.20596 | 18.66766 | 4.306666 | H | 28.30742 | 12.00369 | 4.374546 |
| C | 6.475662 | 2.047992 | 4.297526 | O | 12.84452 | 0.531490 | 4.300516 | H | 5.762620 | 9.524688 | 4.366796 |
| C | 7.197130 | 3.293977 | 4.302776 | O | 12.84753 | 3.551238 | 4.265416 | H | 10.08628 | 9.532068 | 4.362096 |
| C | 6.480634 | 4.537913 | 4.306846 | B | 12.93881 | 4.939757 | 4.257046 | H | 10.07813 | 12.01055 | 4.369826 |
| C | 5.068462 | 4.487441 | 4.297426 | O | 11.69164 | 5.556746 | 4.273196 | H | 25.06132 | 7.668761 | 4.261826 |
| C | 7.233879 | 5.733271 | 4.318776 | C | 9.137340 | 15.76684 | 4.337016 | H | 22.91329 | 8.902993 | 4.234676 |
| C | 8.606708 | 5.731416 | 4.316776 | C | 9.119530 | 14.36666 | 4.352396 | H | 20.75725 | 5.164138 | 4.221276 |
| C | 9.356662 | 4.534379 | 4.303576 | C | 7.917350 | 13.64834 | 4.362166 | H | 22.90186 | 3.923150 | 4.248096 |
| C | 8.637088 | 3.292298 | 4.302016 | C | 29.25856 | 14.36334 | 4.357026 | H | 19.19018 | 6.062794 | 4.208396 |
| C | 9.356211 | 2.044985 | 4.298096 | C | 29.23721 | 15.76352 | 4.341516 | H | 17.02501 | 9.803075 | 4.190126 |
| C | 10.79185 | 2.043364 | 4.293676 | C | 30.45905 | 16.44824 | 4.331646 | H | 21.34636 | 9.808386 | 4.220676 |
| C | 11.45396 | 3.291537 | 4.285116 | C | 7.919790 | 12.17717 | 4.370396 | H | 21.34209 | 11.61083 | 4.224326 |
| C | 10.76853 | 4.481037 | 4.290116 | C | 6.718620 | 11.45246 | 4.372246 | H | 21.34551 | 14.08908 | 4.252566 |
| C | 8.634732 | 0.799057 | 4.297696 | C | 6.722090 | 10.06717 | 4.367666 | H | 17.02162 | 14.08849 | 4.227926 |
| C | 7.194886 | 0.800659 | 4.296376 | C | 7.924391 | 9.335764 | 4.361246 | H | 17.02587 | 11.61013 | 4.199066 |
| C | 17.74881 | 19.08315 | 4.292316 | C | 9.124657 | 10.07074 | 4.365046 | H | 15.46124 | 8.894695 | 4.195176 |
| C | 16.33685 | 19.13630 | 4.292276 | C | 9.123293 | 11.45613 | 4.369596 | H | 13.31586 | 7.654508 | 4.224366 |
| C | 4.377905 | 0.800444 | 4.291676 | C | 27.96203 | 16.49787 | 4.333496 | H | 15.48466 | 3.914481 | 4.233426 |
| C | 11.45145 | 0.793766 | 4.296646 | C | 27.93620 | 17.90045 | 4.319646 | H | 17.62649 | 5.161092 | 4.205196 |
| **c** | 0.00000 | 0.00000 | 6.79272 | serrated armchair | | | | | | | |

## COF-11M

### Layer I AA, AB, inclined zigzag & armchair, serrated zigzag & armchair

| | | | | | | | |
|---|---|---|---|---|---|---|---|
| C | 6.029330 | 8.768199 | 7.743065 | C | 17.15021 | 6.996589 | 7.808554 |
| C | 7.277517 | 9.427519 | 7.752586 | C | 16.46681 | 8.179989 | 7.816382 |
| C | 7.252033 | 10.83956 | 7.750654 | O | 8.650606 | 7.269379 | 7.767845 |
| C | 21.17628 | 11.55042 | 7.740655 | O | 17.41270 | 9.284769 | 7.825348 |
| C | 19.95718 | 10.86990 | 7.732084 | B | 18.64921 | 8.651609 | 7.822211 |
| C | 19.95041 | 9.473769 | 7.733063 | O | 18.57954 | 7.264169 | 7.812064 |
| B | 8.567448 | 8.666579 | 7.765151 | O | 2.290272 | 2.200259 | 7.780051 |
| O | 9.823492 | 9.282629 | 7.775844 | B | 3.526822 | 2.833349 | 7.776802 |
| C | 10.76074 | 8.178919 | 7.784836 | O | 3.457262 | 4.220809 | 7.784103 |
| C | 12.17156 | 8.240099 | 7.796441 | O | 12.44407 | 10.80364 | 7.812343 |
| C | 12.89116 | 6.993119 | 7.797756 | B | 13.60999 | 11.55898 | 7.825169 |
| C | 12.16734 | 5.742769 | 7.793236 | O | 14.77729 | 10.80555 | 7.828099 |
| C | 10.72626 | 5.742849 | 7.786936 | O | 8.650353 | 4.216689 | 7.789758 |
| C | 10.07482 | 6.997209 | 7.780148 | B | 8.566852 | 2.819509 | 7.798276 |
| C | 14.33581 | 6.993719 | 7.804075 | C | 7.276627 | 2.059149 | 7.805703 |
| C | 15.05524 | 8.241649 | 7.813150 | C | 6.028722 | 2.719139 | 7.802707 |
| C | 14.29536 | 9.433119 | 7.816544 | C | 4.827250 | 2.014289 | 7.811697 |
| C | 12.92917 | 9.432439 | 7.807315 | C | 4.833230 | 0.618220 | 7.824137 |
| C | 15.05847 | 5.742629 | 7.800000 | C | 13.61305 | 13.03318 | 7.827328 |
| C | 14.33569 | 4.491589 | 7.796688 | C | 7.250310 | 0.647160 | 7.818232 |
| C | 12.89101 | 4.492319 | 7.796258 | O | 9.822869 | 2.203239 | 7.801608 |
| C | 12.17126 | 3.245409 | 7.798302 | O | 12.44368 | 0.681820 | 7.808698 |

| | | | | | | | |
|---|---|---|---|---|---|---|---|
| C | 10.76039 | 3.306769 | 7.796534 | B | 21.17066 | 13.02252 | 7.809775 |
| C | 10.07465 | 4.488579 | 7.789561 | O | 14.77694 | 0.679700 | 7.803260 |
| C | 12.92882 | 2.052999 | 7.801846 | H | 5.996126 | 3.823479 | 7.793185 |
| C | 14.29506 | 2.052179 | 7.798633 | H | 3.882250 | 0.057630 | 7.831465 |
| C | 15.05502 | 3.243589 | 7.794485 | H | 8.200110 | 0.082340 | 7.821305 |
| C | 16.46660 | 3.305119 | 7.789248 | H | 8.202126 | 11.40385 | 7.757337 |
| C | 17.15010 | 4.488489 | 7.791566 | H | 19.00654 | 11.43107 | 7.724606 |
| C | 16.49860 | 5.742569 | 7.799910 | H | 5.996109 | 7.663829 | 7.743672 |

Transformation Matrix

| | | | | |
|---|---|---|---|---|
| **a** | 15.122130 | 0.000000 | 0.000000 | |
| **b** | 7.561060 | 13.096150 | 0.000000 | |
| **c** | 0.000000 | 0.000000 | 3.465420 | AA |
| **c** | 1.22487 | 0.70718 | 3.40974 | inclined zigzag |
| **c** | 1.407205 | 0.000000 | 3.407183 | inclined armchair |

## Layer II AB stacking

| | | | | | | | |
|---|---|---|---|---|---|---|---|
| C | 13.59039 | 13.13358 | 11.12486 | C | 24.71128 | 11.36197 | 11.19035 |
| C | 14.83858 | 13.79290 | 11.13438 | C | 24.02788 | 12.54537 | 11.19818 |
| C | 14.81310 | 15.20494 | 11.13245 | O | 16.21167 | 11.63476 | 11.14964 |
| C | 28.73735 | 15.91580 | 11.12245 | O | 24.97377 | 13.65015 | 11.20714 |
| C | 27.51825 | 15.23528 | 11.11388 | B | 26.21028 | 13.01699 | 11.20401 |
| C | 27.51148 | 13.83915 | 11.11486 | O | 26.14061 | 11.62955 | 11.19386 |
| B | 16.12851 | 13.03196 | 11.14695 | O | 9.851337 | 6.565642 | 11.16185 |
| O | 17.38456 | 13.64801 | 11.15764 | B | 11.08789 | 7.198732 | 11.15860 |
| C | 18.32181 | 12.54430 | 11.16663 | O | 11.01833 | 8.586192 | 11.16590 |
| C | 19.73263 | 12.60548 | 11.17824 | O | 20.00513 | 15.16902 | 11.19414 |
| C | 20.45222 | 11.35850 | 11.17955 | B | 21.17105 | 15.92436 | 11.20696 |
| C | 19.72840 | 10.10815 | 11.17503 | O | 22.33836 | 15.17093 | 11.20989 |
| C | 18.28732 | 10.10823 | 11.16873 | O | 16.21142 | 8.582072 | 11.17155 |
| C | 17.63589 | 11.36259 | 11.16194 | B | 16.12792 | 7.184892 | 11.18007 |
| C | 21.89688 | 11.35910 | 11.18587 | C | 14.83769 | 6.424532 | 11.18750 |
| C | 22.61630 | 12.60703 | 11.19494 | C | 13.58979 | 7.084522 | 11.18450 |
| C | 21.85642 | 13.79850 | 11.19834 | C | 12.38832 | 6.379672 | 11.19349 |
| C | 20.49023 | 13.79782 | 11.18911 | C | 12.39429 | 4.983603 | 11.20593 |
| C | 22.61954 | 10.10801 | 11.18180 | C | 21.17412 | 17.39856 | 11.20912 |
| C | 21.89676 | 8.856972 | 11.17848 | C | 14.81137 | 5.012543 | 11.20003 |
| C | 20.45208 | 8.857702 | 11.17805 | O | 17.38393 | 6.568622 | 11.18340 |
| C | 19.73232 | 7.610792 | 11.18010 | O | 20.00475 | 5.047203 | 11.19049 |
| C | 18.32146 | 7.672152 | 11.17833 | B | 28.73173 | 17.38790 | 11.19157 |
| C | 17.63572 | 8.853962 | 11.17136 | O | 22.33801 | 5.045083 | 11.18505 |
| C | 20.48989 | 6.418382 | 11.18364 | H | 13.55719 | 8.188862 | 11.17498 |
| C | 21.85612 | 6.417562 | 11.18043 | H | 11.44332 | 4.423013 | 11.21326 |
| C | 22.61609 | 7.608972 | 11.17628 | H | 15.76118 | 4.447723 | 11.20310 |
| C | 24.02767 | 7.670502 | 11.17104 | H | 15.76319 | 15.76923 | 11.13913 |
| C | 24.71117 | 8.853872 | 11.17336 | H | 26.56761 | 15.79645 | 11.10640 |
| C | 24.05967 | 10.10795 | 11.18171 | H | 13.55717 | 12.02921 | 11.12547 |

| | | | | |
|---|---|---|---|---|
| **c** | 0.00000 | 0.00000 | 6.76359 | AB |

## Layer II serrated zigzag

| | | | | | | | |
|---|---|---|---|---|---|---|---|
| C | 7.254205 | 9.475381 | 11.1493 | C | 18.37508 | 7.703771 | 11.2148 |
| C | 8.502392 | 10.13470 | 11.1589 | C | 17.69168 | 8.887171 | 11.2227 |
| C | 8.476908 | 11.54674 | 11.1569 | O | 9.875481 | 7.976561 | 11.1741 |
| C | 22.40115 | 12.25760 | 11.1469 | O | 18.63758 | 9.991951 | 11.2316 |
| C | 21.18205 | 11.57708 | 11.1384 | B | 19.87408 | 9.358791 | 11.2285 |
| C | 21.17529 | 10.18095 | 11.1393 | O | 19.80441 | 7.971351 | 11.2183 |
| B | 9.792322 | 9.373761 | 11.1714 | O | 3.515147 | 2.907441 | 11.1863 |

| | | | | | | | |
|---|---|---|---|---|---|---|---|
| O | 11.04837 | 9.989811 | 11.1821 | B | 4.751697 | 3.540531 | 11.1831 |
| C | 11.98561 | 8.886101 | 11.1911 | O | 4.682137 | 4.927991 | 11.1904 |
| C | 13.39643 | 8.947281 | 11.2027 | O | 13.66895 | 11.51082 | 11.2186 |
| C | 14.11604 | 7.700301 | 11.2040 | B | 14.83487 | 12.26616 | 11.2314 |
| C | 13.39222 | 6.449951 | 11.1995 | O | 16.00216 | 11.51273 | 11.2344 |
| C | 11.95114 | 6.450031 | 11.1932 | O | 9.875229 | 4.923871 | 11.1960 |
| C | 11.29969 | 7.704391 | 11.1864 | B | 9.791727 | 3.526691 | 11.2046 |
| C | 15.56068 | 7.700901 | 11.2103 | C | 8.501502 | 2.766331 | 11.2120 |
| C | 16.28012 | 8.948831 | 11.2194 | C | 7.253597 | 3.426321 | 11.2090 |
| C | 15.52024 | 10.14030 | 11.2228 | C | 6.052125 | 2.721471 | 11.2180 |
| C | 14.15405 | 10.13962 | 11.2136 | C | 6.058105 | 1.325402 | 11.2304 |
| C | 16.28334 | 6.449811 | 11.2063 | C | 14.83792 | 13.74036 | 11.2336 |
| C | 15.56056 | 5.198771 | 11.2030 | C | 8.475185 | 1.354342 | 11.2245 |
| C | 14.11588 | 5.199501 | 11.2025 | O | 11.04774 | 2.910421 | 11.2079 |
| C | 13.39614 | 3.952591 | 11.2046 | O | 13.66855 | 1.389002 | 11.2150 |
| C | 11.98527 | 4.013951 | 11.2028 | B | 22.39553 | 13.72970 | 11.2160 |
| C | 11.29952 | 5.195761 | 11.1958 | O | 16.00182 | 1.386882 | 11.2095 |
| C | 14.15369 | 2.760181 | 11.2081 | H | 7.221001 | 4.530661 | 11.1995 |
| C | 15.51994 | 2.759361 | 11.2049 | H | 5.107125 | 0.764812 | 11.2377 |
| C | 16.27990 | 3.950771 | 11.2008 | H | 9.424986 | 0.789522 | 11.2276 |
| C | 17.69147 | 4.012301 | 11.1955 | H | 9.427000 | 12.11103 | 11.1636 |
| C | 18.37498 | 5.195671 | 11.1978 | H | 20.23141 | 12.13825 | 11.1309 |
| C | 17.72347 | 6.449751 | 11.2062 | H | 7.220984 | 8.371011 | 11.1499 |
| **c** | 0.00000 | 0.00000 | 6.81264 | serrated zigzag | | | |

## Layer II serrated armchair

| | | | | | | | |
|---|---|---|---|---|---|---|---|
| C | 7.436535 | 8.768199 | 11.14939 | C | 18.55742 | 6.996589 | 11.21487 |
| C | 8.684722 | 9.427519 | 11.15891 | C | 17.87402 | 8.179989 | 11.22270 |
| C | 8.659238 | 10.83956 | 11.15697 | O | 10.05781 | 7.269379 | 11.17417 |
| C | 22.58348 | 11.55042 | 11.14697 | O | 18.81991 | 9.284769 | 11.23167 |
| C | 21.36439 | 10.86990 | 11.13840 | B | 20.05642 | 8.651609 | 11.22853 |
| C | 21.35762 | 9.473769 | 11.13938 | O | 19.98675 | 7.264169 | 11.21838 |
| B | 9.974652 | 8.666579 | 11.17147 | O | 3.697477 | 2.200259 | 11.18637 |
| O | 11.23070 | 9.282629 | 11.18216 | B | 4.934027 | 2.833349 | 11.18312 |
| C | 12.16794 | 8.178919 | 11.19116 | O | 4.864467 | 4.220809 | 11.19042 |
| C | 13.57876 | 8.240099 | 11.20276 | O | 13.85127 | 10.80364 | 11.21866 |
| C | 14.29836 | 6.993119 | 11.20408 | B | 15.01719 | 11.55898 | 11.23149 |
| C | 13.57454 | 5.742769 | 11.19956 | O | 16.18450 | 10.80555 | 11.23442 |
| C | 12.13346 | 5.742849 | 11.19326 | O | 10.05756 | 4.216689 | 11.19608 |
| C | 11.48202 | 6.997209 | 11.18647 | B | 9.974056 | 2.819509 | 11.20460 |
| C | 15.74301 | 6.993719 | 11.21039 | C | 8.683832 | 2.059149 | 11.21202 |
| C | 16.46244 | 8.241649 | 11.21947 | C | 7.435927 | 2.719139 | 11.20903 |
| C | 15.70256 | 9.433119 | 11.22286 | C | 6.234455 | 2.014289 | 11.21802 |
| C | 14.33637 | 9.432439 | 11.21363 | C | 6.240435 | 0.618220 | 11.23046 |
| C | 16.46568 | 5.742629 | 11.20632 | C | 15.02026 | 13.03318 | 11.23365 |
| C | 15.74289 | 4.491589 | 11.20301 | C | 8.657515 | 0.647160 | 11.22455 |
| C | 14.29821 | 4.492319 | 11.20258 | O | 11.23007 | 2.203239 | 11.20793 |
| C | 13.57846 | 3.245409 | 11.20462 | O | 13.85088 | 0.681820 | 11.21502 |
| C | 12.16759 | 3.306769 | 11.20285 | B | 22.57787 | 13.02252 | 11.21609 |
| C | 11.48185 | 4.488579 | 11.19588 | O | 16.18414 | 0.679700 | 11.20958 |
| C | 14.33602 | 2.052999 | 11.20817 | H | 7.403331 | 3.823479 | 11.19950 |
| C | 15.70226 | 2.052179 | 11.20495 | H | 5.289455 | 0.057630 | 11.23779 |
| C | 16.46222 | 3.243589 | 11.20081 | H | 9.607315 | 0.082340 | 11.22762 |
| C | 17.87381 | 3.305119 | 11.19557 | H | 9.609330 | 11.40385 | 11.16366 |
| C | 18.55731 | 4.488489 | 11.19789 | H | 20.41375 | 11.43107 | 11.13093 |
| C | 17.90581 | 5.742569 | 11.20623 | H | 7.403314 | 7.663829 | 11.14999 |

**c** 0.00000 0.00000 6.81264 serrated armchair

# TP COF-2M

## Layer I AA, AB, inclined zigzag & armchair, serrated zigzag & armchair

| | | | | | | | | | | | |
|---|---|---|---|---|---|---|---|---|---|---|---|
| C | 11.53373 | 17.29940 | 7.897015 | C | 6.948308 | 5.495443 | 7.790462 | C | 15.51659 | 5.074529 | 7.910435 |
| C | 10.81540 | 16.08678 | 7.889568 | C | 6.963305 | 6.861492 | 7.807947 | C | 14.79675 | 6.309104 | 7.922283 |
| C | 9.386423 | 16.12945 | 7.891382 | C | 5.650118 | 3.343056 | 7.768093 | C | 15.52279 | 7.539138 | 7.913510 |
| C | 30.43892 | 17.38967 | 7.900753 | C | 4.452850 | 2.681089 | 7.770287 | C | 16.92713 | 7.509400 | 7.893935 |
| C | 31.20175 | 18.57367 | 7.907957 | O | 19.92867 | 5.149659 | 7.851935 | C | 13.36539 | 6.316542 | 7.942979 |
| C | 10.86680 | 18.52356 | 7.906059 | B | 19.15823 | 6.316537 | 7.864342 | C | 12.65723 | 7.558408 | 7.954891 |
| C | 28.99692 | 17.39786 | 7.902335 | O | 19.94037 | 7.478925 | 7.860467 | C | 13.41708 | 8.783940 | 7.945538 |
| C | 28.27953 | 16.23948 | 7.894841 | O | 21.52952 | 10.09981 | 7.846352 | C | 14.77946 | 8.775408 | 7.925808 |
| C | 28.93109 | 14.95246 | 7.884808 | B | 22.17415 | 11.34171 | 7.846267 | C | 14.75644 | 3.845973 | 7.919866 |
| C | 8.631410 | 14.91323 | 7.883423 | C | 21.44998 | 12.65246 | 7.848131 | C | 13.39303 | 3.851842 | 7.939608 |
| C | 9.294085 | 13.64705 | 7.873590 | C | 22.15148 | 13.87463 | 7.849526 | C | 12.64163 | 5.083708 | 7.951964 |
| C | 8.522690 | 12.47073 | 7.864807 | C | 21.49758 | 15.11753 | 7.853895 | C | 11.23282 | 5.120296 | 7.972434 |
| C | 28.83890 | 12.48731 | 7.865308 | C | 20.06886 | 15.15289 | 7.856809 | C | 10.55090 | 6.336157 | 7.983824 |
| C | 28.20938 | 13.74730 | 7.875818 | C | 19.33805 | 13.92472 | 7.854147 | C | 11.24899 | 7.542093 | 7.975286 |
| C | 11.45896 | 14.79528 | 7.880022 | C | 20.04195 | 12.70941 | 7.849787 | H | 27.10604 | 13.79462 | 7.876607 |
| C | 10.73877 | 13.63745 | 7.872524 | C | 19.37718 | 16.40523 | 7.862851 | H | 9.048549 | 11.49908 | 7.856876 |
| B | 28.03353 | 11.22390 | 7.854322 | C | 20.11559 | 17.62964 | 7.865990 | H | 23.25629 | 13.85647 | 7.847719 |
| O | 6.884225 | 9.950203 | 7.842186 | C | 21.55708 | 17.56425 | 7.862517 | H | 19.46911 | 11.76453 | 7.848138 |
| C | 5.755644 | 9.046624 | 7.834604 | C | 22.21482 | 16.37033 | 7.856641 | H | 17.47370 | 8.468995 | 7.887759 |
| C | 5.783925 | 7.635924 | 7.820360 | C | 17.94759 | 16.43221 | 7.866268 | H | 17.46608 | 4.140168 | 7.882244 |
| C | 4.526696 | 6.938115 | 7.817264 | C | 17.23538 | 15.17725 | 7.863058 | H | 12.63820 | 17.29018 | 7.895675 |
| C | 25.01942 | 7.688411 | 7.828087 | C | 17.89570 | 13.98475 | 7.857155 | H | 19.83174 | 0.738500 | 7.915137 |
| C | 25.05096 | 9.130285 | 7.837695 | C | 17.29024 | 17.67784 | 7.872745 | H | 16.18677 | 17.71970 | 7.875516 |
| C | 26.31880 | 9.760226 | 7.841638 | C | 7.153850 | 0.050300 | 7.875762 | H | 9.099760 | 0.989080 | 7.875019 |
| C | 23.75442 | 6.989074 | 7.829306 | C | 8.547570 | 0.032790 | 7.872471 | H | 10.65328 | 4.180081 | 7.979670 |
| C | 22.51656 | 7.729205 | 7.839743 | O | 23.56869 | 11.22691 | 7.844745 | H | 10.68723 | 8.492787 | 7.984669 |
| C | 22.61078 | 9.138580 | 7.843066 | O | 26.63275 | 11.17893 | 7.854360 | H | 23.31998 | 16.35819 | 7.854279 |
| C | 23.80739 | 9.800035 | 7.842160 | O | 21.36941 | 2.531600 | 7.825842 | H | 22.13660 | 18.50505 | 7.864892 |
| C | 23.73321 | 5.544355 | 7.820461 | B | 21.96362 | 1.276360 | 7.811249 | H | 17.31745 | 13.04286 | 7.854983 |
| C | 22.47369 | 4.850706 | 7.826912 | O | 1.626130 | 1.303490 | 7.794398 | H | 16.13047 | 15.18767 | 7.865653 |
| C | 21.29406 | 5.624925 | 7.841077 | O | 4.694230 | 1.246050 | 7.748926 | H | 15.32964 | 9.733315 | 7.919179 |
| C | 21.30888 | 6.990464 | 7.845919 | B | 6.080430 | 1.151040 | 7.734575 | H | 12.87823 | 9.748173 | 7.954705 |
| C | 4.505702 | 5.492987 | 7.802299 | O | 6.737047 | 2.375562 | 7.745324 | H | 15.29094 | 2.878303 | 7.911016 |
| C | 3.240720 | 4.793623 | 7.804294 | O | 8.320065 | 5.004504 | 7.780248 | H | 12.84489 | 2.892184 | 7.946462 |
| C | 3.208953 | 3.352280 | 7.790728 | B | 9.082167 | 6.167326 | 7.793336 | H | 12.56318 | 14.74916 | 7.878684 |
| C | 1.939740 | 2.726316 | 7.799774 | O | 8.335097 | 7.338007 | 7.810074 | H | 11.27392 | 12.67013 | 7.865308 |
| C | 22.50238 | 3.440037 | 7.818167 | C | 17.65861 | 6.306237 | 7.882397 | H | 28.46698 | 18.36703 | 7.909660 |
| C | 5.743215 | 4.753431 | 7.786190 | C | 16.92294 | 5.102267 | 7.890790 | H | 27.17570 | 16.28299 | 7.896270 |

Transformation Matrix

**a** 21.727490 0.000000 0.000000

**b** 10.863740 18.816550 0.000000

**c** 0.000000 0.000000 3.468760 AA

**c** 1.40699 0.00000 3.400518 inclined zigzag

**c** 1.22029 0.70453 3.404958 inclined armchair

## Layer II serrated zigzag

| | | | | | | | | | | | |
|---|---|---|---|---|---|---|---|---|---|---|---|
| C | 12.75401 | 18.00393 | 11.29992 | C | 8.168593 | 6.199975 | 11.19336 | C | 16.73688 | 5.779061 | 11.31334 |
| C | 12.03569 | 16.79131 | 11.29247 | C | 8.183590 | 7.566024 | 11.21085 | C | 16.01703 | 7.013636 | 11.32518 |
| C | 10.60671 | 16.83398 | 11.29428 | C | 6.870403 | 4.047588 | 11.17099 | C | 16.74307 | 8.243670 | 11.31641 |
| C | 31.65920 | 18.09420 | 11.30365 | C | 5.673135 | 3.385621 | 11.17319 | C | 18.14742 | 8.213932 | 11.29683 |
| C | 32.42204 | 19.27820 | 11.31086 | O | 21.14896 | 5.854191 | 11.25484 | C | 14.58568 | 7.021074 | 11.34588 |
| C | 12.08709 | 19.22809 | 11.30896 | B | 20.37852 | 7.021069 | 11.26724 | C | 13.87752 | 8.262939 | 11.35779 |
| C | 30.21721 | 18.10239 | 11.30523 | O | 21.16066 | 8.183457 | 11.26337 | C | 14.63737 | 9.488472 | 11.34844 |

| | | | | | | | | | | | |
|---|---|---|---|---|---|---|---|---|---|---|---|
| C | 29.49981 | 16.94401 | 11.29774 | O | 22.74981 | 10.80434 | 11.24925 | C | 15.99975 | 9.479939 | 11.32871 |
| C | 30.15137 | 15.65699 | 11.28771 | B | 23.39444 | 12.04624 | 11.24917 | C | 15.97673 | 4.550505 | 11.32277 |
| C | 9.851695 | 15.61776 | 11.28632 | C | 22.67027 | 13.35699 | 11.25103 | C | 14.61332 | 4.556374 | 11.34251 |
| C | 10.51437 | 14.35158 | 11.27649 | C | 23.37177 | 14.57916 | 11.25243 | C | 13.86192 | 5.788239 | 11.35486 |
| C | 9.742975 | 13.17526 | 11.26771 | C | 22.71786 | 15.82206 | 11.25679 | C | 12.45310 | 5.824828 | 11.37533 |
| C | 30.05919 | 13.19184 | 11.26821 | C | 21.28914 | 15.85742 | 11.25971 | C | 11.77119 | 7.040689 | 11.38672 |
| C | 29.42966 | 14.45183 | 11.27872 | C | 20.55834 | 14.62925 | 11.25705 | C | 12.46928 | 8.246625 | 11.37819 |
| C | 12.67924 | 15.49981 | 11.28292 | C | 21.26224 | 13.41394 | 11.25269 | H | 28.32632 | 14.49915 | 11.27951 |
| C | 11.95905 | 14.34198 | 11.27542 | C | 20.59747 | 17.10976 | 11.26575 | H | 10.26883 | 12.20361 | 11.25978 |
| B | 29.25381 | 11.92843 | 11.25722 | C | 21.33587 | 18.33417 | 11.26889 | H | 24.47658 | 14.56100 | 11.25062 |
| O | 8.104510 | 10.65473 | 11.24509 | C | 22.77736 | 18.26878 | 11.26542 | H | 20.68940 | 12.46906 | 11.25104 |
| C | 6.975929 | 9.751156 | 11.23750 | C | 23.43511 | 17.07486 | 11.25954 | H | 18.69398 | 9.173527 | 11.29066 |
| C | 7.004210 | 8.340456 | 11.22326 | C | 19.16788 | 17.13674 | 11.26917 | H | 18.68637 | 4.844700 | 11.28514 |
| C | 5.746981 | 7.642647 | 11.22016 | C | 18.45567 | 15.88178 | 11.26596 | H | 13.85849 | 17.99471 | 11.29858 |
| C | 26.23971 | 8.392943 | 11.23099 | C | 19.11599 | 14.68928 | 11.26005 | H | 21.05202 | 1.443032 | 11.31804 |
| C | 26.27125 | 9.834817 | 11.24059 | C | 18.51052 | 18.38237 | 11.27565 | H | 17.40705 | 18.42423 | 11.27842 |
| C | 27.53909 | 10.46476 | 11.24454 | C | 8.374135 | 0.7548319 | 11.27866 | H | 10.32005 | 1.693612 | 11.27792 |
| C | 24.97470 | 7.693606 | 11.23221 | C | 9.767856 | 0.7373219 | 11.27537 | H | 11.87357 | 4.884613 | 11.38257 |
| C | 23.73685 | 8.433737 | 11.24264 | O | 24.78897 | 11.93144 | 11.24765 | H | 11.90752 | 9.197319 | 11.38757 |
| C | 23.83107 | 9.843112 | 11.24597 | O | 27.85303 | 11.88346 | 11.25726 | H | 24.54027 | 17.06272 | 11.25718 |
| C | 25.02768 | 10.50457 | 11.24506 | O | 22.58969 | 3.236132 | 11.22874 | H | 23.35689 | 19.20958 | 11.26779 |
| C | 24.95350 | 6.248887 | 11.22336 | B | 23.18390 | 1.980892 | 11.21415 | H | 18.53773 | 13.74739 | 11.25788 |
| C | 23.69398 | 5.555238 | 11.22981 | O | 2.846415 | 2.008022 | 11.19730 | H | 17.35076 | 15.89220 | 11.26855 |
| C | 22.51435 | 6.329457 | 11.24398 | O | 5.914515 | 1.950582 | 11.15183 | H | 16.54992 | 10.43785 | 11.32208 |
| C | 22.52917 | 7.694996 | 11.24882 | B | 7.300715 | 1.855572 | 11.13748 | H | 14.09852 | 10.45270 | 11.35760 |
| C | 5.725987 | 6.197519 | 11.20520 | O | 7.957332 | 3.080094 | 11.14822 | H | 16.51122 | 3.582835 | 11.31392 |
| C | 4.461005 | 5.498155 | 11.20719 | O | 9.540350 | 5.709036 | 11.18315 | H | 14.06518 | 3.596716 | 11.34936 |
| C | 4.429238 | 4.056812 | 11.19363 | B | 10.30245 | 6.871858 | 11.19624 | H | 13.78347 | 15.45369 | 11.28158 |
| C | 3.160025 | 3.430848 | 11.20267 | O | 9.555383 | 8.042539 | 11.21297 | H | 12.49421 | 13.37466 | 11.26821 |
| C | 23.72267 | 4.144569 | 11.22107 | C | 18.87889 | 7.010769 | 11.28530 | H | 29.68727 | 19.07156 | 11.31256 |
| C | 6.963500 | 5.457963 | 11.18909 | C | 18.14322 | 5.806799 | 11.29369 | H | 28.39598 | 16.98752 | 11.29917 |

**c** 0.00000 0.00000 6.80580 serrated zigzag

## Layer II serrated armchair

| | | | | | | | | | | | |
|---|---|---|---|---|---|---|---|---|---|---|---|
| C | 12.94072 | 17.29940 | 11.29992 | C | 8.355298 | 5.495443 | 11.19336 | C | 16.92358 | 5.074529 | 11.31334 |
| C | 12.22239 | 16.08678 | 11.29247 | C | 8.370295 | 6.861492 | 11.21085 | C | 16.20374 | 6.309104 | 11.32518 |
| C | 10.79341 | 16.12945 | 11.29428 | C | 7.057108 | 3.343056 | 11.17099 | C | 16.92978 | 7.539138 | 11.31641 |
| C | 31.84591 | 17.38967 | 11.30365 | C | 5.859840 | 2.681089 | 11.17319 | C | 18.33412 | 7.509400 | 11.29683 |
| C | 32.60874 | 18.57367 | 11.31086 | O | 21.33566 | 5.149659 | 11.25484 | C | 14.77238 | 6.316542 | 11.34588 |
| C | 12.27379 | 18.52356 | 11.30896 | B | 20.56522 | 6.316537 | 11.26724 | C | 14.06422 | 7.558408 | 11.35779 |
| C | 30.40391 | 17.39786 | 11.30523 | O | 21.34736 | 7.478925 | 11.26337 | C | 14.82407 | 8.783940 | 11.34844 |
| C | 29.68652 | 16.23948 | 11.29774 | O | 22.93651 | 10.09981 | 11.24925 | C | 16.18645 | 8.775408 | 11.32871 |
| C | 30.33808 | 14.95246 | 11.28771 | B | 23.58114 | 11.34171 | 11.24917 | C | 16.16343 | 3.845973 | 11.32277 |
| C | 10.03840 | 14.91323 | 11.28632 | C | 22.85697 | 12.65246 | 11.25103 | C | 14.80002 | 3.851842 | 11.34251 |
| C | 10.70107 | 13.64705 | 11.27649 | C | 23.55847 | 13.87463 | 11.25243 | C | 14.04862 | 5.083708 | 11.35486 |
| C | 9.929680 | 12.47073 | 11.26771 | C | 22.90457 | 15.11753 | 11.25679 | C | 12.63981 | 5.120296 | 11.37533 |
| C | 30.24589 | 12.48731 | 11.26821 | C | 21.47585 | 15.15289 | 11.25971 | C | 11.95789 | 6.336157 | 11.38672 |
| C | 29.61637 | 13.74730 | 11.27872 | C | 20.74504 | 13.92472 | 11.25705 | C | 12.65598 | 7.542093 | 11.37819 |
| C | 12.86595 | 14.79528 | 11.28292 | C | 21.44894 | 12.70941 | 11.25269 | H | 28.51303 | 13.79462 | 11.27951 |
| C | 12.14576 | 13.63745 | 11.27542 | C | 20.78417 | 16.40523 | 11.26575 | H | 10.45554 | 11.49908 | 11.25978 |
| B | 29.44052 | 11.22390 | 11.25722 | C | 21.52258 | 17.62964 | 11.26889 | H | 24.66328 | 13.85647 | 11.25062 |
| O | 8.291215 | 9.950203 | 11.24509 | C | 22.96407 | 17.56425 | 11.26542 | H | 20.87610 | 11.76453 | 11.25104 |
| C | 7.162634 | 9.046624 | 11.23750 | C | 23.62181 | 16.37033 | 11.25954 | H | 18.88069 | 8.468995 | 11.29066 |
| C | 7.190915 | 7.635924 | 11.22326 | C | 19.35458 | 16.43221 | 11.26917 | H | 18.87307 | 4.140168 | 11.28514 |
| C | 5.933686 | 6.938115 | 11.22016 | C | 18.64237 | 15.17725 | 11.26596 | H | 14.04519 | 17.29018 | 11.29858 |
| C | 26.42641 | 7.688411 | 11.23099 | C | 19.30269 | 13.98475 | 11.26005 | H | 21.23873 | 0.738500 | 11.31804 |
| C | 26.45795 | 9.130285 | 11.24059 | C | 18.69723 | 17.67784 | 11.27565 | H | 17.59376 | 17.71970 | 11.27842 |
| C | 27.72579 | 9.760226 | 11.24454 | C | 8.560841 | 0.050300 | 11.27866 | H | 10.50675 | 0.989080 | 11.27792 |
| C | 25.16141 | 6.989074 | 11.23221 | C | 9.954560 | 0.032790 | 11.27537 | H | 12.06027 | 4.180081 | 11.38257 |
| C | 23.92355 | 7.729205 | 11.24264 | O | 24.97568 | 11.22691 | 11.24765 | H | 12.09422 | 8.492787 | 11.38757 |
| C | 24.01777 | 9.138580 | 11.24597 | O | 28.03974 | 11.17893 | 11.25726 | H | 24.72697 | 16.35819 | 11.25718 |
| C | 25.21438 | 9.800035 | 11.24506 | O | 22.77640 | 2.531600 | 11.22874 | H | 23.54359 | 18.50505 | 11.26779 |

| | | | | | | | | | | | |
|---|---|---|---|---|---|---|---|---|---|---|---|
| C | 25.14020 | 5.544355 | 11.22336 | B | 23.37061 | 1.276360 | 11.21415 | H | 18.72444 | 13.04286 | 11.25788 |
| C | 23.88068 | 4.850706 | 11.22981 | O | 3.033120 | 1.303490 | 11.19730 | H | 17.53746 | 15.18767 | 11.26855 |
| C | 22.70105 | 5.624925 | 11.24398 | O | 6.101220 | 1.246050 | 11.15183 | H | 16.73663 | 9.733315 | 11.32208 |
| C | 22.71587 | 6.990464 | 11.24882 | B | 7.487420 | 1.151040 | 11.13748 | H | 14.28522 | 9.748173 | 11.35760 |
| C | 5.912692 | 5.492987 | 11.20520 | O | 8.144037 | 2.375562 | 11.14822 | H | 16.69793 | 2.878303 | 11.31392 |
| C | 4.647710 | 4.793623 | 11.20719 | O | 9.727055 | 5.004504 | 11.18315 | H | 14.25188 | 2.892184 | 11.34936 |
| C | 4.615943 | 3.352280 | 11.19363 | B | 10.48916 | 6.167326 | 11.19624 | H | 13.97017 | 14.74916 | 11.28158 |
| C | 3.346730 | 2.726316 | 11.20267 | O | 9.742087 | 7.338007 | 11.21297 | H | 12.68091 | 12.67013 | 11.26821 |
| C | 23.90937 | 3.440037 | 11.22107 | C | 19.06560 | 6.306237 | 11.28530 | H | 29.87397 | 18.36703 | 11.31256 |
| C | 7.150205 | 4.753431 | 11.18909 | C | 18.32993 | 5.102267 | 11.29369 | H | 28.58269 | 16.28299 | 11.29917 |
| **c** | 0.00000 | 0.00000 | 6.80580 | serrated armchair | | | | | | | |

# REF-I

## Layer I AA, AB, inclined zigzag, and serrated zigzag

| | | | |
|---|---|---|---|
| O | 8.53482300 | 5.11449700 | 0.79376800 |
| B | 5.86856000 | 0.79392900 | 0.79376800 |
| B | 7.07711000 | 2.88729900 | 0.79376800 |
| O | 5.86856000 | 3.57514900 | 0.79376800 |
| B | 4.66000000 | 2.88729900 | 0.79376800 |
| O | 4.66869000 | 1.49697900 | 0.79376800 |
| B | 9.73479300 | 5.81749700 | 0.79376800 |
| B | 3.21081700 | 3.72373700 | 0.79376800 |
| O | 2.00231700 | 3.03588700 | 0.79376800 |
| O | 3.20228700 | 5.11449700 | 0.79376800 |
| B | 8.52630300 | 3.72373700 | 0.79376800 |
| O | 7.06842000 | 1.49697900 | 0.79376800 |

Transformation Matrix

| | | | | |
|---|---|---|---|---|
| **a** | 7.732466 | 0.000000 | 0.000000 | |
| **b** | 3.866233 | 6.696508 | 0.000000 | |
| **c** | 0.000000 | 0.000000 | 3.592001 | AA |
| **c** | 1.208490 | 0.697722 | 3.446160 | inclined zigzag |

## Layer II AB stacking

| | | | |
|---|---|---|---|
| O | 12.40106 | 7.346668 | 4.158838 |
| B | 9.734793 | 3.026100 | 4.158838 |
| B | 10.94334 | 5.119470 | 4.158838 |
| O | 9.734793 | 5.807320 | 4.158838 |
| B | 8.526233 | 5.119470 | 4.158838 |
| O | 8.534924 | 3.729150 | 4.158838 |
| B | 13.60103 | 8.049667 | 4.158838 |
| B | 7.077050 | 5.955908 | 4.158838 |
| O | 5.868550 | 5.268058 | 4.158838 |
| O | 7.068520 | 7.346668 | 4.158838 |
| B | 12.39254 | 5.955908 | 4.158838 |
| O | 10.93465 | 3.729150 | 4.158838 |
| **c** | 0.00000 | 0.00000 | 6.73014 AB |

## Layer II serrated zigzag

| | | | |
|---|---|---|---|
| O | 9.743313 | 5.812217 | 4.285588 |
| B | 7.077050 | 1.491659 | 4.285588 |
| B | 8.285600 | 3.585029 | 4.285588 |
| O | 7.077050 | 4.272869 | 4.285588 |
| B | 5.868490 | 3.585029 | 4.285588 |
| O | 5.877180 | 2.194699 | 4.285588 |

| | | | |
|---|---|---|---|
| B | 10.94328 | 6.515227 | 4.285588 |
| B | 4.419297 | 4.421457 | 4.285588 |
| O | 3.210807 | 3.733607 | 4.285588 |
| O | 4.410777 | 5.812217 | 4.285588 |
| B | 9.734793 | 4.421457 | 4.285588 |
| O | 8.276910 | 2.194699 | 4.285588 |

**c** 0.000000 0.000000 6.98364 serrated zigzag

# REF-II

## Layer I AA, AB, inclined armchair, and serrated armchair

| | | | | | | | |
|---|---|---|---|---|---|---|---|
| O | 15.328768 | 8.0298630 | 0.793768 | B | 15.243038 | 6.6372530 | 0.793768 |
| O | 16.974848 | 10.881153 | 0.793768 | O | 16.491958 | 6.0148430 | 0.793768 |
| C | 11.608732 | 2.9338220 | 0.793768 | C | 16.749358 | 8.3077030 | 0.793768 |
| C | 10.221312 | 2.9345120 | 0.793768 | C | 17.443178 | 7.1061530 | 0.793768 |
| C | 9.5268420 | 4.1387820 | 0.793768 | C | 4.3841160 | 7.1063330 | 0.793768 |
| C | 10.220672 | 5.3405120 | 0.793768 | C | 17.444578 | 9.5118230 | 0.793768 |
| C | 11.611122 | 5.3399020 | 0.793768 | C | 5.0784360 | 8.3079230 | 0.793768 |
| C | 12.304472 | 4.1378520 | 0.793768 | C | 18.832338 | 9.5120830 | 0.793768 |
| B | 10.913192 | 0.7940220 | 0.793768 | O | 5.3337660 | 6.0145530 | 0.793768 |
| O | 9.7503720 | 1.5653020 | 0.793768 | O | 6.4986960 | 8.0284530 | 0.793768 |
| O | 8.1061320 | 4.4158220 | 0.793768 | B | 6.5831160 | 6.6357230 | 0.793768 |
| B | 8.0199620 | 5.8084320 | 0.793768 | O | 19.301238 | 10.881833 | 0.793768 |
| O | 9.2685720 | 6.4313320 | 0.793768 | B | 18.137838 | 11.652123 | 0.793768 |
| O | 12.563232 | 6.4312020 | 0.793768 | O | 12.077002 | 1.5636820 | 0.793768 |
| B | 13.809412 | 5.8101220 | 0.793768 | O | 13.725892 | 4.4157220 | 0.793768 |

Transformation Matrix

| | | | | |
|---|---|---|---|---|
| **a** | 14.449272 | 0.000000 | 0.000000 | |
| **b** | 7.224636 | 12.513431 | 0.000000 | |
| **c** | 0.000000 | 0.000000 | 3.528001 | AA |
| **c** | 1.389180 | 0.000000 | 3.487678 | inclined armchair |

## Layer II AB stacking

| | | | | | | | |
|---|---|---|---|---|---|---|---|
| O | 22.55340 | 12.20101 | 4.103020 | B | 22.46767 | 10.80840 | 4.103020 |
| O | 24.19948 | 15.05230 | 4.103020 | O | 23.71659 | 10.18599 | 4.103020 |
| C | 18.83337 | 7.104967 | 4.103020 | C | 23.97399 | 12.47885 | 4.103020 |
| C | 17.44595 | 7.105658 | 4.103020 | C | 24.66781 | 11.27730 | 4.103020 |
| C | 16.75148 | 8.309927 | 4.103020 | C | 11.60875 | 11.27748 | 4.103020 |
| C | 17.44531 | 9.511658 | 4.103020 | C | 24.66921 | 13.68297 | 4.103020 |
| C | 18.83576 | 9.511047 | 4.103020 | C | 12.30307 | 12.47907 | 4.103020 |
| C | 19.52911 | 8.308998 | 4.103020 | C | 26.05697 | 13.68323 | 4.103020 |
| B | 18.13783 | 4.965168 | 4.103020 | O | 12.55840 | 10.18570 | 4.103020 |
| O | 16.97501 | 5.736447 | 4.103020 | O | 13.72333 | 12.19960 | 4.103020 |
| O | 15.33077 | 8.586967 | 4.103020 | B | 13.80775 | 10.80687 | 4.103020 |
| B | 15.24460 | 9.979578 | 4.103020 | O | 26.52587 | 15.05298 | 4.103020 |
| O | 16.49321 | 10.60248 | 4.103020 | B | 25.36247 | 15.82327 | 4.103020 |
| O | 19.78787 | 10.60235 | 4.103020 | O | 19.30164 | 5.734828 | 4.103020 |
| B | 21.03405 | 9.981268 | 4.103020 | O | 20.95053 | 8.586867 | 4.103020 |

**c** 0.00000 0.00000 6.618505 AB

## Layer II serrated armchair

| | | | | | | | |
|---|---|---|---|---|---|---|---|
| O | 16.717948 | 8.0298630 | 4.271908 | B | 16.632218 | 6.6372530 | 4.271908 |
| O | 18.364028 | 10.881153 | 4.271908 | O | 17.881138 | 6.0148430 | 4.271908 |

| | | | | | | | |
|---|---|---|---|---|---|---|---|
| C | 12.997912 | 2.9338220 | 4.271908 | C | 18.138538 | 8.3077030 | 4.271908 |
| C | 11.610492 | 2.9345120 | 4.271908 | C | 4.3830860 | 7.1061530 | 4.271908 |
| C | 10.916022 | 4.1387820 | 4.271908 | C | 5.7732960 | 7.1063330 | 4.271908 |
| C | 11.609842 | 5.3405120 | 4.271908 | C | 18.833758 | 9.5118230 | 4.271908 |
| C | 13.000302 | 5.3399020 | 4.271908 | C | 6.4676160 | 8.3079230 | 4.271908 |
| C | 13.693652 | 4.1378520 | 4.271908 | C | 5.7722460 | 9.5120830 | 4.271908 |
| B | 12.302372 | 0.7940220 | 4.271908 | O | 6.7229460 | 6.0145530 | 4.271908 |
| O | 11.139552 | 1.5653020 | 4.271908 | O | 7.8878760 | 8.0284530 | 4.271908 |
| O | 9.4953020 | 4.4158220 | 4.271908 | B | 7.9722960 | 6.6357230 | 4.271908 |
| B | 9.4091420 | 5.8084320 | 4.271908 | O | 20.690418 | 10.881833 | 4.271908 |
| O | 10.657752 | 6.4313320 | 4.271908 | B | 19.527018 | 11.652123 | 4.271908 |
| O | 13.952402 | 6.4312020 | 4.271908 | O | 13.466182 | 1.5636820 | 4.271908 |
| B | 15.198592 | 5.8101220 | 4.271908 | O | 15.115072 | 4.4157220 | 4.271908 |
| **c** | 0.00000 | 0.00000 | 6.95628 | serrated armchair | | | |
