# Supplementary material for: On the reticular construction concept of covalent organic frameworks
Source: Beilstein J Nanotechnol. 2010 Nov 22;1:60–70. doi: 10.3762/bjnano.1.8 (PMC3045923; doi:10.3762/bjnano.1.8)
Supplement: File 2 — CIFs for selected structures. [file Beilstein_J_Nanotechnol-01-60-s002.zip › S2.pdf]

Supporting Information

for

On the reticular construction concept of covalent organic frameworks

Binit Lukose[1], Agnieszka Kuc[1], Johannes Frenzel[2], and Thomas Heine*[1]

Address: [1]School of Engineering and Science, Jacobs University Bremen, Research III, Room 61, Campus Ring 1, Bremen 28759, Germany and [2]Lehrstuhl für Theoretische Chemie, Ruhr-Universität Bochum, Bochum 44780, Germany

Email: Thomas Heine - t.heine@jacobs-university.de

* Corresponding author

CIFs for selected structures

This ZIP file contains following crystallography information files (CIFs) of some of the studied
COF structures:

File name	COF
COF-10_inc-z_1.cif :	COF-10 inclined zigzag
COF-1M_AA_1.cif:	COF-1M AA
COF-1M_AB_1.cif:	COF-1M AB
COF-1M_inc-z_1.cif:	COF-1M inclined zigzag
COF-1M_ser-z_1.cif:	COF-1M serrated zigzag
COF-8M_AA_1.cif:	COF-8M AA
COF-8M_AB_1.cif:	COF-8M AB
COF-8M_inc-a_1.cif:	COF-8M inclined armchair
COF-8M_inc-z_1.cif:	COF-8M inclined zigzag
COF-8M_ser-a_1.cif:	COF-8M serrated armchair
Ppy-COF_AB_1.cif:	PPy-COF AB
Ppy-COF_inc-z_1.cif:	PPy-COF inclined zigzag
Ppy-COF_ser-z_1.cif:	PPy-COF serrated zigzag
TPCOF-1M_inc-z_1.cif:	TP COF-1M inclined zigzag
TPCOF_inc-z_1.cif:	TP COF inclined zigzag
